# Pharmacological Mechanisms Underlying the Hepatoprotective Effects of *Ecliptae herba* on Hepatocellular Carcinoma

**DOI:** 10.1155/2021/5591402

**Published:** 2021-07-16

**Authors:** Botao Pan, Wenxiu Pan, Zheng Lu, Chenglai Xia

**Affiliations:** ^1^Affiliated Foshan Maternity & Child Healthcare Hospital, Southern Medical University, Foshan 528000, China; ^2^Department of Laboratory, Fifth People's Hospital of Foshan, Foshan 528000, China; ^3^Wuzhou Maternal and Child Health-Care Hospital, Wuzhou 543000, China; ^4^School of Pharmaceutical Sciences, Southern Medical University, Guangzhou 510515, China

## Abstract

**Background:**

The number of hepatocellular carcinoma (HCC) cases worldwide has increased significantly. As a traditional Chinese medicine (TCM) with a long history, *Ecliptae herba* (EH) has been widely used in HCC patients in China, but its hepatoprotective mechanism is still unclear.

**Methods:**

In this study, we applied a network pharmacology-based strategy and experimental verification to systematically unravel the underlying mechanisms of EH against HCC. First, six active ingredients of EH were screened from the Traditional Chinese Medicine Systems Pharmacology Database and Analysis Platform (TCMSP) by the ADME method. Subsequently, 52 potential targets of 6 active ingredients acting on HCC were screened from various databases, including TCMSP, DGIdb, SwissTargetPrediction, CTD, and GeneCards. Then, by constructing protein-protein interaction (PPI) network from STRING, we displayed the intricate connections among these 52 targets through Cytoscape software. We also applied enrichment analysis, including Gene Ontology (GO) and Kyoto Encyclopedia of Genes and Genomes (KEGG) analyses, to provide an outline and set of concepts for describing gene functions and the advanced functions of biological systems of these 52 targets from genomic and molecular level information. Finally, molecular docking and biological experiments were used to reconfirm these results.

**Results:**

We hypothesized that EH might exert anti-HCC activity by acting on hub genes, including RELA, MMP9, PTGS2, ESR1, EGFR, AR, AKT1, HIF1A, AHR, CYP3A4, ABCG2, and MMP2. Moreover, based on GO and KEGG analysis, we speculated that EH may exert hepatoprotective effects on HCC through the following mechanisms: regulation of the PI3K-AKT signaling pathway to promote apoptosis and inhibit the abnormal proliferation of HCC, downregulation of HIF-1A expression by activating the HIF-1 signaling pathway, prevention of HCC by regulating lipid metabolism, and inhibition of nonalcoholic fatty liver disease (NAFLD) by the cytochrome P450 subfamily. Subsequent biological experiments verified that EH inhibits the PI3K-AKT signaling pathway through its active ingredients, quercetin, and wedelolactone, thereby inhibiting the proliferation of HCC cells and promoting the apoptosis of HCC cells.

**Conclusions:**

The network pharmacological strategy provides an efficient method to systematically explore the pharmacological mechanism of EH in HCC. Our study demonstrated that the anti-HCC proliferation activity of EH is mainly exerted by two active ingredients (quercetin and wedelolactone), which inhibit the proliferation of HCC cells (HepG2 and Huh-7) by inhibiting PI3K-AKT signaling.

## 1. Background

Cancer statistics show that liver cancer is the fourth most common cause of cancer-related death globally, with the World Health Organization estimating that more than 1 million people will die from liver cancer by 2030 [1]. More than 80%–90% of cases of liver cancer are classified as hepatocellular carcinoma (HCC), which is the most common primary liver cancer [2]. Hepatocellular carcinoma typically develops on the background of chronic liver disease, mostly as a result of hepatitis B (HBV) or hepatitis C (HBC) virus infection, alcohol abuse, and nonalcoholic fatty liver disease [3]. HCC is not caused by a single gene, but by an accumulation of multiple genetic defects and mutations. The pathogenesis of HCC is complex and includes multiple biological mechanisms such as epithelial-mesenchymal transition (EMT), the tumor microenvironment, tumor-stromal interactions, immune mechanisms, and well-known signaling pathways [4]. There are several treatments for HCC, including curative treatment options for early-stage HCC (surgical options, ablative electrochemical therapies, chemoembolization, radioembolization, and liver transplantation) and systemic therapy (targeted therapy and immunotherapy) for those patients who have failed locoregional therapy [1, 5–7]. However, the main drawbacks of curative therapies are recurrence of HCC and lower survival benefits than systemic chemotherapies. Although these therapeutics have greatly prolonged the survival of some patients with HCC, more effective medications and therapies are urgently needed.

Traditional Chinese medicine, which originated in China, has been practiced by Chinese people for thousands of years because of its satisfactory therapeutic effect and few side effects [8–10]. It has played an important role in the health care of Asians. *Ecliptae herba* (EH), known as “Mo-Han-Lian” in China, has been used as a “liver-nourishing” treatment in traditional Chinese medicine (TCM) for several thousand years [11]. EH has a broad range of pharmacological properties including antioxidant, antimicrobial, anti-inflammatory, immunomodulatory, hepatoprotective, and hypolipidemic effects [11–17]. Ethanolic extract of EH has been shown to have hepatoprotective effects on damaged livers in rats and mice [18, 19]. Its extracts include a variety of natural products, such as triterpenoid saponins, coumarins, and flavonoids [11]. Although several *in vitro* and *in vivo* experiments have shown that EH has hepatoprotective and antihepatotoxic effects in recent years, the detailed therapeutic targets and molecular mechanisms of EH against HCC have not been systematically elucidated.

Network pharmacology is an innovative method to investigate the interrelationship between drugs and diseases by integrating multiple sources of information which is an efficient method for understanding the effects of EH in HCC treatment. The strategy is based on the concept of “Medicine-Target-Gene-Disease,” which can illuminate intricate drug-disease interactions, and is considered to be very suitable for studying the molecular mechanisms of drugs and drug discovery [20–22].

In this study, we performed an integrated strategy, including network pharmacology-based analysis, molecular docking, and biological experimental verification, to identify the potential therapeutic targets and the associated molecular mechanisms of EH against HCC.

## 2. Methods

### 2.1. Data Preparation

#### 2.1.1. Screening the Active Ingredients of EH

We collected the ingredients of EH from the Traditional Chinese Medicine System Pharmacology Database (TCMSP, https://tcmspw.com/tcmsp.php). In this database, the Chinese characters of “Mo-Han-Lian” were input to identify related ingredients and their pharmacokinetic property data.

#### 2.1.2. Screening the Targets of EH Active Ingredients, HCC Disease Targets, and EH Treatment Targets in HCC

We collected relevant targets of EH active ingredients by screening different databases. These databases include the TCMSP, the Drug Gene Interaction Database (DGIdb, https://dgidb.genome.wustl.edu/), and the SwissTargetPrediction web server (http://www.swisstargetprediction.ch/).

Related targets of HCC were collected through the Comparative Toxicogenomics Database (CTD, http://ctdbase.org/) and the GeneCards web server (https://www.genecards.org/). We searched for the keyword “hepatocellular carcinoma” on these web servers, and the species was restricted to “Homo sapiens.” Then, we used an open web tool (http://bioinformatics.psb.ugent.be/webtools/Venn/) to filter the common targets of different databases to improve the reliability of the data obtained. We used this web tool to match the predicted target of EH active ingredients with the relevant target of HCC to obtain the overlapping targets. These overlapping targets are the targets that EH may play a role in the treatment of HCC.

### 2.2. Network Construction and Topology Analysis

In this process, a compound-target-disease network (C-T-D network), EH target-HCC target network (E-H network), compound-target-pathway network (C-T-P network), and hub gene network were constructed to reveal the pharmacological mechanism and therapeutic target of EH in the treatment of HCC. The graphical interactions in the C-T-D network were visualized by Cytoscape software (version 3.7.2). The E-H network was obtained by uploading the overlapping targets to the STRING (https://string-db.org/) web server. On this database, the species option was “Homo sapiens,” protein interactions had a confidence score >0.4, and the disconnected nodes were hidden in this network. The resulting data were imported into the visualization software Cytoscape to establish protein-protein interaction networks of EH against HCC. Based on this E-H network, the hub gene network was obtained through the CytoHubba plugin in Cytoscape software. In addition, we constructed the C-T-P network from the results of the Kyoto Encyclopedia of Genes and Genomes (KEGG) pathway enrichment analysis. For each network, we selected five parameters to evaluate its topological characteristics, which were calculated by the Cytoscape plugin, Network Analyzer. These topological parameters were “Degree,” “Betweenness Centrality,” “Closeness Centrality,” “Clustering Coefficient,” and “Topological Coefficient.”

### 2.3. Enrichment Analysis

In this study, we performed Gene Ontology (GO) analysis of the overlapping targets through the WEB-based Gene SeT AnaLysis Toolkit (WebGestalt, http://www.webgestalt.org/). The organism of interest was “Homo sapiens,” the method of interest was “Over-Representation Analysis,” and the functional database was “Gene Ontology.” Then, the overlapping gene list was uploaded to the database to obtain GO enrichment analysis results, which included three parts, biological process (BP), cellular component (CC), and molecular function (MF). Then, we constructed a network of enriched GO terms through WebGestalt analysis of the overlapping genes and TCGA RNASeq liver hepatocellular carcinoma (LIHC) database, with the option of method of interest selected as “Network Topology-based Analysis,” and the option of functional database selected as “TCGA RNASeq LIHC.”

In addition, we calculated and evaluated significant pathways using KEGG (http://www.kegg.jp/) data obtained from WebGestalt, with the option of method of interest being “Over-Representation Analysis,” and the functional database being “pathway.”

### 2.4. Molecular Docking

In this study, we performed molecular docking to further reveal the binding pattern of EH and hub genes. The X-ray crystal structures of hub genes were obtained from the Protein Data Bank (PDB, https://www.rcsb.org/). AutoDock Vina and PyMOL 1.8 software were utilized to perform docking studies for EH and selected genes. First, the 2D chemical structures of the active ingredients of EH were downloaded from PubChem (https://pubchem.ncbi.nlm.nih.gov/) with the format for SDF and converted to a .pdb format by PyMOL 1.8. For protein preparation, the protein was input into the PyMOL 1.8 to remove the water molecules and heteroatoms and saved in a .pdb format. Second, the .pdb format of the protein and active ingredients was converted to the .pdbqt format via AutoDockTools (version 1.5.6) and used to perform a series of operations, including adding hydrogen atoms and calculating and adding Gasteiger charges. Third, we defined the specific pocket for active ingredients of EH binding with the hub genes using the grid box function of AutoDockTools. We used the command prompt for molecule docking analysis and the output results were displayed by PyMOL.

### 2.5. Cell Culture and Reagents

Hepatocellular carcinoma cell lines (HepG2 and Huh-7) were obtained from South Medical University Affiliated Maternal & Child Health Hospital of Foshan (Foshan, China). HepG2 cells were cultured with DMEM (Gibco, NY, USA) containing 1% penicillin-streptomycin (Sigma, MO, USA) and 10% fetal bovine serum (FBS; Gibco, NY, USA) under 5% CO_2_ at 37°C. Huh-7 cells were cultured with RPMI 1640 (Gibco, NY, USA) medium containing 1% penicillin-streptomycin (Sigma, MO, USA) and 10% fetal bovine serum (FBS; Gibco, NY, USA) under 5% CO_2_ at 37°C.

Wedelolactone was purchased from MedChemExpress (Monmouth Junction, USA), and quercetin was purchased from Shanghai Fushen Biotechnology Co., Ltd. (Shanghai, China). Raw EH was purchased from Foshan Zhongtian Chinese Medicine Pieces Co., Ltd. (Foshan, China). The raw EH was authenticated by Deng Dongmei from the Department of TCM of the South Medical University Affiliated Maternal and Child Health Hospital of Foshan. The extraction step of EH was carried out using a previously reported protocol [23]. The final extract of EH was redissolved at a concentration of 0.15 g/mL, filtered through a 0.22*μ* membrane, and then stored at −20°C.

### 2.6. Cell Viability Assay

HepG2 or Huh-7 cells (3,500) were transplanted into each well of a 96-well plate and treated with different concentrations of EH, wedelolactone, and quercetin for 48 hours. Then, the proliferation of HCC cells was measured by CCK-8 assays (Beyotime, Shanghai, China). The cell viability of the sample was calculated by the OD value.

### 2.7. Flow Cytometry Analysis for Detection of Cell Apoptosis

HepG2 or Huh-7 cells were treated with 150 *μ*g/L EH, 8 *μ*g/mL wedelolactone, and 8 *μ*g/mL quercetin for 48 hours, according to the Annexin FITC/propidium iodide (PI) cell apoptosis kit (KeyGEN BioTECH, Nanjing, China) instructions for the experimental procedures. FACSCanto II (Becton, Dickinson and Company, New Jersey, USA) was used for the analysis, and the results were analyzed by FlowJo V10 software.

### 2.8. Western Blot Analysis

After treatment of HepG2 or Huh-7, cells were treated with 150 *μ*g/mL EH, 8 *μ*g/mL wedelolactone, and 8 *μ*g/mL quercetin for 48 hours, western blotting was performed using previously reported protocol standards [24]. The antibodies used in this study included the following: PI3K, Akt, p-Akt (Ser473), and beta-actin, and horseradish peroxidase-conjugated secondary antibodies were purchased from CST (Cell Signaling Technology, Danvers, MA, USA). The p-PI3K p85 (Tyr467)/p55 (Tyr199) antibody was purchased from Affinity Biosciences Ltd. (Jiangsu, China). Band intensity was analyzed by ImageJ software (National Institutes of Health, Bethesda, MD, USA).

### 2.9. Statistical Analysis

Each experiment was repeated three times independently. Data are presented as the mean ± standard error of the mean (SEM) and analyzed using GraphPad Prism 7 (GraphPad Software, Inc., La Jolla, CA, USA). One-way analysis of variance (ANOVA) was used, and the difference was considered significant when *P* value < 0.05.

## 3. Results

### 3.1. Active Ingredients of Ecliptae herba

**Figure 1:**
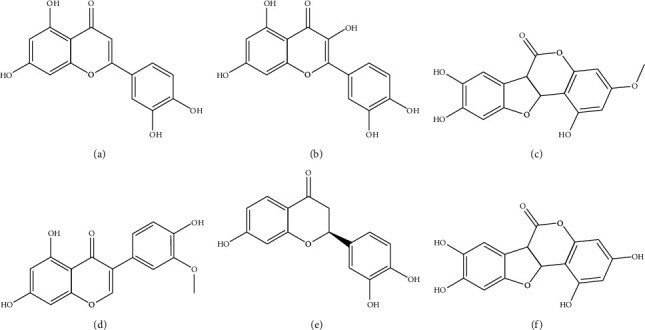
The chemical structures of 6 active ingredients of *Ecliptae herba*. (a) Luteolin. (b) Quercetin. (c) Wedelolactone. (d) 3′-O-Methylorobol. (e) Butin. (f) Demethylwedelolactone.

A total of 48 chemical ingredients of EH were obtained from TCMSP (Supplementary File S1). ADME-related properties are an important component of pharmaceutical R&D, including Lipinski's “Rule of Five,” which is often referred to as a guideline for drug lead optimization [25–27]. These properties are oral bioavailability (OB), drug-likeness (DL), half-life (HL), blood-brain barrier (BBB) permeability, Caco-2 permeability, molecular weight (MW), logarithm of the 1-octanol/water partition coefficient (Log *P*), hydrogen-bond donors (Hdon), and hydrogen-bond acceptors (Hacc). According to the ADME thresholds of DL > 0.18, OB > 30%, Caco-2 > 0, BBB permeability < −0.3, MW < 500 Da, Log *P* < 3, Hdon < 5, and Hacc < 10, 6 active ingredients were selected out [28, 29]. These active ingredients were luteolin, quercetin, wedelolactone, 3′-O-methylorobol, demethylwedelolactone, and butin. Their pharmacokinetic parameters are shown in Table 1 and their chemical structures are shown in Figure 1.

### 3.2. Target Screening and Analysis

**Figure 2:**
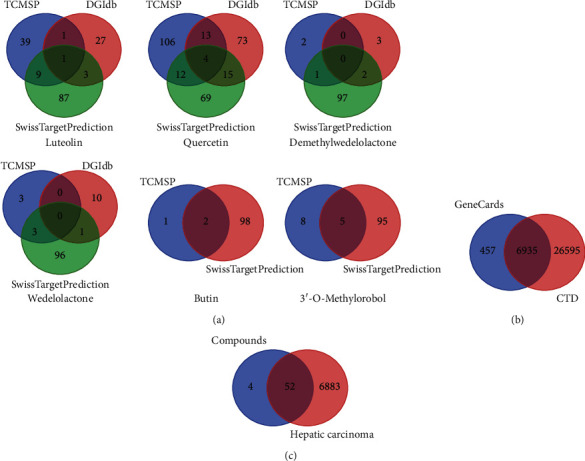
Screening the potential targets of EH in the treatment of HCC. (a) Venn diagram of targets of 6 active ingredients of EH collected from different databases. (b) Venn diagram of targets of HCC collected from GeneCards and CTD databases. (c) Venn diagram of targets of EH against HCC.

In this study, we identified 426 related targets of 6 active ingredients of EH from 3 different databases, TCMSP, DGIdb, and SwissTargetPrediction (Figure 2(a), and Supplementary File S2). Luteolin had 50 targets from TCMSP, 32 targets from DGIdb, and 100 targets from SwissTargetPrediction. Quercetin had 135 targets from TCMSP, 105 targets from DGIdb, and 100 targets from SwissTargetPrediction. Demethylwedelolactone had 3 targets from TCMSP, 5 targets from DGIdb, and 100 targets from SwissTargetPrediction. Wedelolactone had 6 targets from TCMSP, 11 targets from DGIdb, and 100 targets from SwissTargetPrediction, and 3′-O-methylorobol had 13 targets from TCMSP, 0 targets from DGIdb, and 100 targets from SwissTargetPrediction. Butin obtained 3 targets from TCMSP, 0 targets from DGIdb, and 100 targets from SwissTargetPrediction. To improve the credibility of the potential targets of these 6 active ingredients of EH, we selected the overlapping targets in these databases as the objects of the next study. A total of 56 overlapping targets were collected, including 14 overlapping targets of luteolin, 44 overlapping targets of quercetin, 3 overlapping targets of demethylwedelolactone, 4 overlapping targets of wedelolactone, 5 overlapping targets of 3′-O-methylorobol, and 2 overlapping targets of butin.

For the pathogenetic targets of HCC, 33530 and 7392 were obtained from CTD and GeneCards, respectively, with 6935 overlapping targets screened from these two databases (Figure 2(b), and Supplementary File S3). By calculating the overlapping targets of 56 targets of 6 active ingredients and 6935 overlapping HCC targets, we obtained 52 therapeutic targets for EH in the treatment of HCC, and the detailed information is shown in Figure 2(c) and Table 2.

### 3.3. Network Construction and Topology Analysis

**Figure 3:**
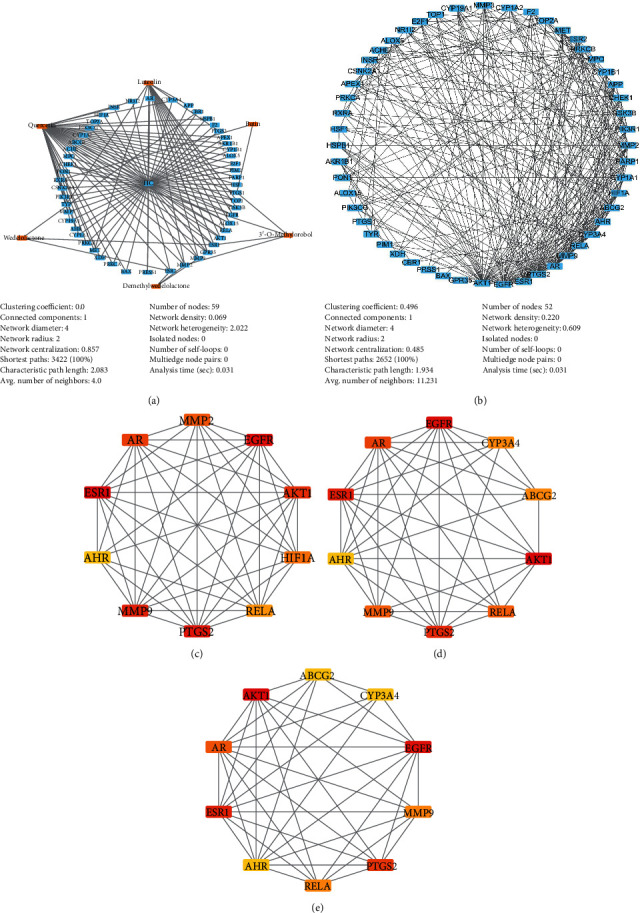
The network was constructed based on 52 therapeutic targets of EH in the treatment of HCC and the top 10 hub genes were analyzed in this network. (a) Compound-target-disease (C-T-D) network. (b) EH target-HCC target (E-H) network. (c) Top 10 hub genes from the maximum neighborhood component (MNC) method. (d) Top 10 hub genes from the maximal clique centrality (MCC) method. (e) Top 10 hub genes from the degree method.

To clarify the potential mechanism of EH on HCC, we constructed the C-T-D network, E-H network, and hub gene network based on these 52 therapeutic targets of EH in the treatment of HCC. The C-T-D network was composed of 59 nodes and 118 edges, and quercetin had more edges, which suggests that it plays a more complex biological role in the anti-HCC function of EH (Figure 3(a)).

The PPI network can expand the understanding of protein function, so we generated an E-H network to systematically study the biological function and mechanism of these 52 potential therapeutic targets of EH against HCC (Supplementary File S4). There were a total of 52 nodes and 292 edges in this E-H network, and as shown in Figure 3(b), AKT1, EGFR, and ESR1 had more edges among these genes. To further reveal the interaction of this E-H network, we adopted the plugin of Cytoscape, Network Analyzer, to calculate the topological parameters of these hub genes. The main topological parameters used in this study were “Degree,” “Betweenness Centrality,” “Closeness Centrality,” “Clustering Coefficient,” and “Topological Coefficient.” As shown in Table 3, AKT1 (degree = 35), EGFR (degree = 29), ESR1 (degree = 27), PTGS2 (degree = 25), AR (degree = 22), MMP9 (degree = 19), RELA (degree = 19), ABCG2 (degree = 16), AHR (degree = 16), and CYP3A4 (degree = 16) have high degrees, indicating that they play a core role in the E-H network (Supplementary File S5). Moreover, the genes with higher values of “Betweenness Centrality,” “Closeness Centrality,” “Clustering Coefficient,” and “Topological Coefficient” were identified as essential proteins with significant centrality values based on the network topology analysis of 52 genes.

Furthermore, the top 10 hub genes in the E-H network were calculated using different methods through the plugin CytoHubba, and the networks were displayed by Cytoscape. These calculation methods include the degree method, maximum neighborhood component (MNC), and maximal clique centrality (MCC). As shown in Figure 3(c), the top 10 hub genes of the MCC calculation were RELA, MMP9, PTGS2, ESR1, HIF1A, EGFR, AHR, AKT1, AR, and MMP2. For the MNC method, RELA, MMP9, PTGS2, ESR1, HIF1A, EGFR, AKT1, ABCG2, AR, and CYP3A4 were obtained as the top 10 hub genes in this network (Figure 3(d)). For the degree method, RELA, MMP9, PTGS2, ESR1, EGFR, AHR, AKT1, ABCG2, CYP3A4, and AR were collected (Figure 3(e)). These results suggest that these genes, such as RELA, MMP9, PTGS2, ESR1, EGFR, AR, AKT1, HIF1A, AHR, CYP3A4, ABCG2, and MMP2, play a very important role in this E-H network.

### 3.4. GO and KEGG Pathway Enrichment Analysis

**Figure 4:**
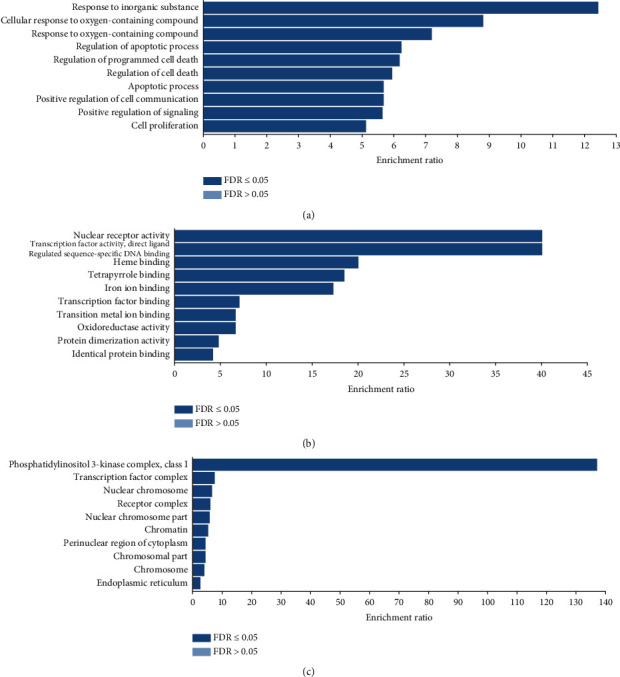
Gene Ontology (GO) enrichment analysis of 52 targets of EH in the treatment of HCC analyzed through WebGestalt. (a) The top 10 biological processes. (b) The top 10 molecular functions. (c) The top 10 cellular components.

After uploading 52 therapeutic targets of EH in the treatment of HCC in WebGestalt, we obtained the results of the top 10 GO enrichment analyses, which were divided into biological processes, molecular functions, and cellular components (Figures 4(a)–4(c), and Supplementary File S6). For this study, only processes or pathways with a *P* value < 0.05 were considered significant pathways. The top 10 biological processes were mainly involved in proliferation, cell death, cell communication, and cell apoptosis. The biological processes included the apoptotic process (GO:0006915), regulation of the apoptotic process (GO:0042981), regulation of cell death (GO:0010941), regulation of programmed cell death (GO:0043067), positive regulation of cell communication (GO:0010647), and cell proliferation (GO:0008283). These results suggest that EH may induce anti-HCC activity by regulating cell proliferation, death, and apoptosis. In molecular function analysis, EH exerted anti-HCC activity mainly through the following processes: transition metal ion binding (GO:0046914), identical protein-binding (GO:0042802), protein dimerization activity (GO:0046983), oxidoreductase activity (GO:0016491), heme binding (GO:0020037), and transcription factor binding (GO:0004879). In addition, the cellular components of the 52 targets of EH in the treatment of HCC were mainly the nuclear chromosome (GO:0000228), transcription factor complex (GO:0005667), nuclear chromosome part (GO:0044454), chromosome (GO:0005694), and chromosomal part (GO:0044427).

**Figure 5:**
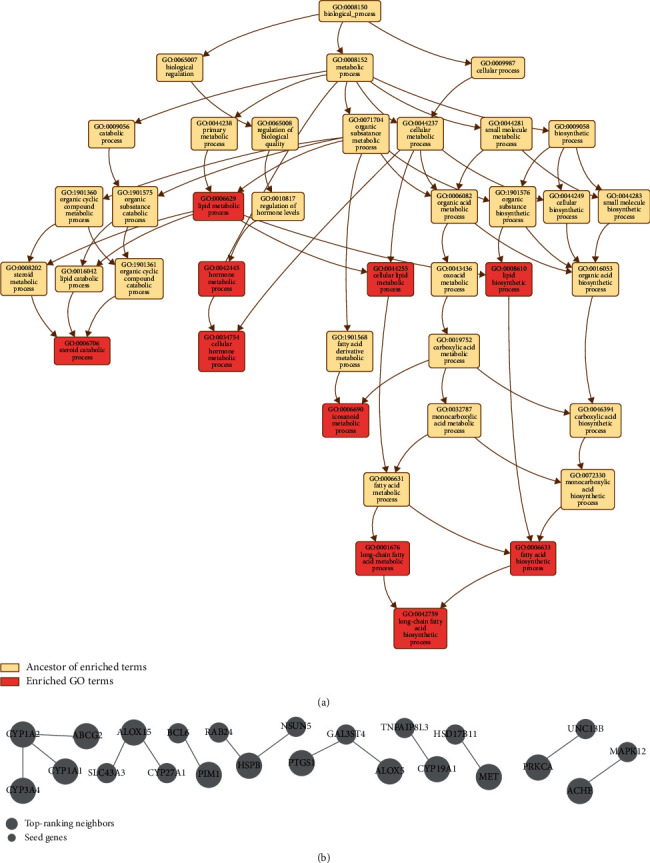
Gene Ontology (GO) network. (a) GO network in the TCGA RNASeq LIHC database through the Network Topology-based Analysis (NTA) method in WebGestalt. (b) The subnetwork of the GO network.

To further reveal the mechanism of action of EH in the treatment of HCC, we built a GO network in the TCGA RNASeq LIHC database through the Network Topology-based Analysis (NTA) method on the WebGestalt web server (Supplementary File S7). As shown in Figure 5(a), the top 10 enriched GO terms were mainly involved in metabolic process, biosynthetic process, and catabolic process. For metabolic process, the GO network mainly included long-chain fatty acid metabolic process (GO:0001676), lipid metabolic process (GO:0006629), cellular lipid metabolic process (GO:0044255), hormone metabolic process (GO:0042445), eicosanoid metabolic process (GO:0006690), and cellular hormone metabolic process (GO:0034754). Moreover, through TCGA RNASeq LIHC database analysis, we obtained key genes from the subnetwork showing the anti-HCC activity of EH, such as CYP1A2, CYP3A4, ABCG2, CYP19A1, ALOX5, PTGS1, PRKCA, CYP1A1, ALOX15, HSPB1, MET, ACHE, and PIM1 (Figure 5(b)).

**Figure 6:**
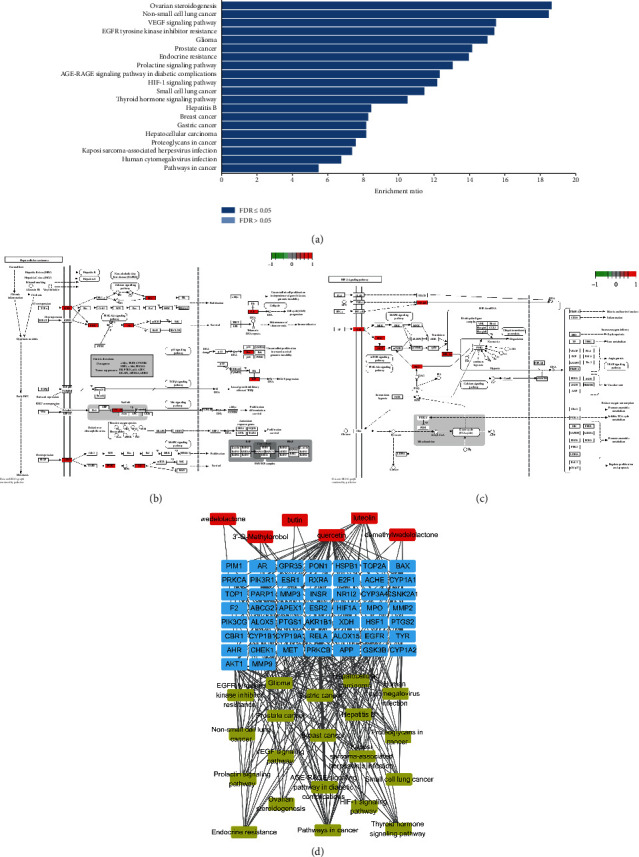
Kyoto Encyclopedia of Genes and Genomes (KEGG) pathway enrichment analysis and C-T-P network analysis. (a) Bar chart of the top 20 KEGG pathways. (b) Pathway diagram of EH acting on HCC through the hepatocellular carcinoma pathway. The nodes in red represent targets that EH may regulate in this pathway. (c) Pathway diagram of EH acting on HCC through the HIF-1 signaling pathway. The nodes in red represent targets that EH may regulate in this pathway. (d) C-T-P network. Nodes represent the compounds (red), the targets (blue), and the top 20 KEGG pathways (yellow). The edges represent compound-target and target-pathway interactions.

Then, we performed KEGG pathway enrichment analysis to further determine the functions and signaling pathways involved in the 52 therapeutic targets of EH against HCC. As shown in Figure 6(a), the molecular signaling pathway of EH against HCC was related to various pathways, including “pathways in cancer,” “non-small cell lung cancer,” “prostate cancer,” “endocrine resistance,” “EGFR tyrosine kinase inhibitor resistance,” “AGE-RAGE signaling pathway in diabetic complications,” “HIF-1 signaling pathway,” “glioma,” “proteoglycans in cancer,” “ovarian steroidogenesis,” “thyroid hormone signaling pathway,” “hepatocellular carcinoma,” “human cytomegalovirus infection,” “VEGF signaling pathway,” “small cell lung cancer,” “Kaposi sarcoma-associated herpesvirus infection,” “hepatitis B,” “breast cancer,” “gastric cancer,” and “prolactin signaling pathway.” The details of the KEGG pathway enrichment information are shown in Supplementary File S8.

### 3.5. Compound-Target-Pathway (C-T-P) Network Analysis

In this study, we tried to further analyze the interactions among 6 active components, 52 targets, and the top 20 KEGG enrichment pathways in EH treatment of HCC, and the compound-target-pathway network could be used to analyze the relationships among these factors. As shown in Figure 6(d), the C-T-P network was comprised of 77 nodes (20 pathways, 6 compounds, and 51 targets) and 384 edges, indicating that EH plays an anti-HCC role through a complex mechanism. There were 51 targets involved in the top 20 KEGG pathways associated with HCC, and each pathway contained more than 6 targets. Among these 20 pathways, the pathways in cancer were enriched by 19 proteins, with the highest degree of enrichment. For the targets, AKT1 was enriched by 19 pathways and 2 compounds, and the enrichment was the highest among the targets. In addition, quercetin was enriched by 40 targets in this network, and these targets were widely enriched in the top 20 KEGG pathways. We speculated that EH was effective for the treatment of HCC. Detailed information is provided in Supplementary File S9.

### 3.6. Molecular Docking Verification

In this study, a molecular docking strategy was used to reveal the binding pattern between the active ingredients of EH and the hub genes. According to the prediction results of molecular docking, quercetin, luteolin, wedelolactone, demethylwedelolactone, and 3′-O-methylorobol bind these hub targets well with low binding energy (Table 4). This result may be because these active ingredients have multiple hydroxyl groups, making them good hydrogen-bond donors or acceptors.

**Figure 7:**
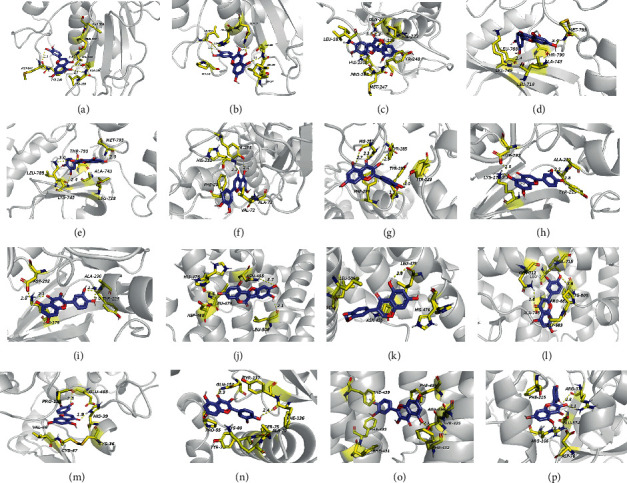
The 3D interaction diagram of the compound and the target was analyzed by the molecular docking method and displayed by PyMoL 1.8. Compounds (purple), residue (yellow), hydrogen bonding (dotted line). (a) Luteolin-MMP9. (b) Quercetin-MMP9. (c) Wedelolactone-MMP9. (d) Luteolin-EGFR. (e) Quercetin-EGFR. (f) Luteolin-RELA. (g) Quercetin-RELA. (h) Luteolin-AKT1. (i) Quercetin-AKT1. (j) Demethylwedelolactone-ESR1. (k) 3′-O-Methylorobol-ESR1. (l) Luteolin-AR. (m) Luteolin-PTGS2. (n) Quercetin-AHR. (o) Quercetin-ABCG2. (p) Quercetin-CYP3A4.

As shown in Figure 7, docking results predicted that active ingredients of EH can form stable noncovalent interactions with the top 10 hub genes. In the protein-binding pocket of MMP9, luteolin can form 5 H-bond interactions (VAL-223, ALA-189, MET-247), quercetin forms 5 H-bond interactions (LEU-243, GLN-227, ALA-189), and wedelolactone forms 2 H-bond interactions (GLN-227). In the ATP-binding pocket of EGFR, luteolin can form 2 H-bond interactions (LYS-745, MET-793), and quercetin can form 3 H-bond interactions (LEU-788, LYS-745, MET-793). The predicted binding patterns of luteolin and quercetin with RELA showed that they formed 2 H-bonds (TYR-285, HIS-252) and 5 H-bonds (HIS-252, TYR-285, TYR-223, TYR-297), respectively. Similarly, luteolin and quercetin could form 4 H-bonds (ASP-292, LYS-179, ALA-230) and 4 H-bonds (ASP-292, LYS-179, ALA-230) with the binding pocket of AKT1, respectively. In addition, demethylwedelolactone and 3′-O-methylorobol could bind with ESR1 to form 3 H-bonds (HIS-476, ASN-455, LEU-508) and 1 H-bonds (LEU-479), respectively. Luteolin can bind to AR (GLN-711, HIS-808) and PTGS2 (HIS-39, GLU-465) at very low binding energies, forming two H bonds. Quercetin can bind to the protein pockets of AHR (GLN-150, PHE-136), ABCG2 (THR-435), and CYP3A4 (ARG-372, ARG-106) to form 2, 1, and 3 H-bonds with very low binding energies, respectively. Analysis of the interaction mode between protein and ligand indicates that active ingredients may bind to these hub targets well and have lower binding energy primarily by forming multiple hydrogen bonds. Furthermore, the model of molecular docking provides evidence for how EH acts on these targets to inhibit HCC.

### 3.7. Ecliptae herba, Wedelolactone, and Quercetin Inhibit the Growth of HepG2 and Huh-7 Cells

**Figure 8:**
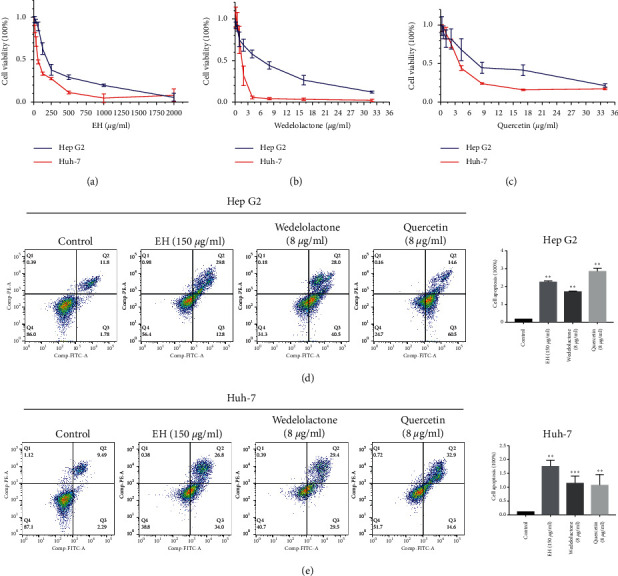
*Ecliptae herba*, wedelolactone, and quercetin inhibited proliferation and promoted apoptosis in hepatocellular carcinoma cell lines. (a) The CCK-8 assay detected the proliferation of HepG2 and Huh-7 cells treated with different concentrations of Ecliptae herba for 48 hours. (b) The CCK-8 assay detected the proliferation of HepG2 and Huh-7 cells treated with different concentrations of wedelolactone for 48 hours. (c) The CCK-8 assay detected the proliferation of HepG2 and Huh-7 cells treated with different concentrations of quercetin for 48 hours. (d) Flow cytometry detected the apoptosis of HepG2 cells with 150 *μ*g/mL EH, 8 *μ*g/mL wedelolactone, and 8 *μ*g/mL quercetin treatment after Annexin-V-FITC and PI staining. (e) Flow cytometry detected the apoptosis of Huh-7 cells with 150 *μ*g/mL EH, 8 *μ*g/mL wedelolactone, and 8 *μ*g/mL quercetin treatment after Annexin-V-FITC and PI staining. The data are expressed as the mean ± standard deviation values; *n* = 3. ^*∗∗*^ indicates *P* < 0.01 and ^*∗∗∗*^ indicates *P* < 0.001 compared to the control group.

Although network pharmacology results indicate that EH could inhibit HCC through multiple active ingredients and pathway mechanisms, biological experiments are still needed to further verify the current results. The foregoing C-T-D network results indicate that quercetin plays a complex biological role in the anti-HCC function of EH. In addition, the aforementioned study showed that wedelolactone may be an important compound for EH to exert biological activity. Therefore, we used these two active ingredients for subsequent biological research. First, the results of the CCK-8 assay showed that EH, wedelolactone, and quercetin could effectively inhibit the proliferation of the HCC cell lines HepG2 and Huh-7 (Figures 8(a)–8(c)). Treatment with the same concentrations of EH, wedelolactone, and quercetin more effectively inhibited the proliferation of Huh-7 cells than HepG2 cells.

Next, to investigate whether EH, wedelolactone, and quercetin decrease the viability of HCC cell lines by inducing apoptosis, we used Annexin V and PI staining to calculate apoptosis by flow cytometry. As shown in Figures 8(d) and 8(e), treatment of HepG2 and Huh-7 cells with 150 *μ*g/mL EH, 8 *μ*g/mL wedelolactone, and 8 *μ*g/mL quercetin for 48 hours significantly promoted HCC cell apoptosis.

### 3.8. Wedelolactone and Quercetin Inhibit the PI3K/Akt Signaling Pathway in HepG2 and Huh-7 Cells

**Figure 9:**
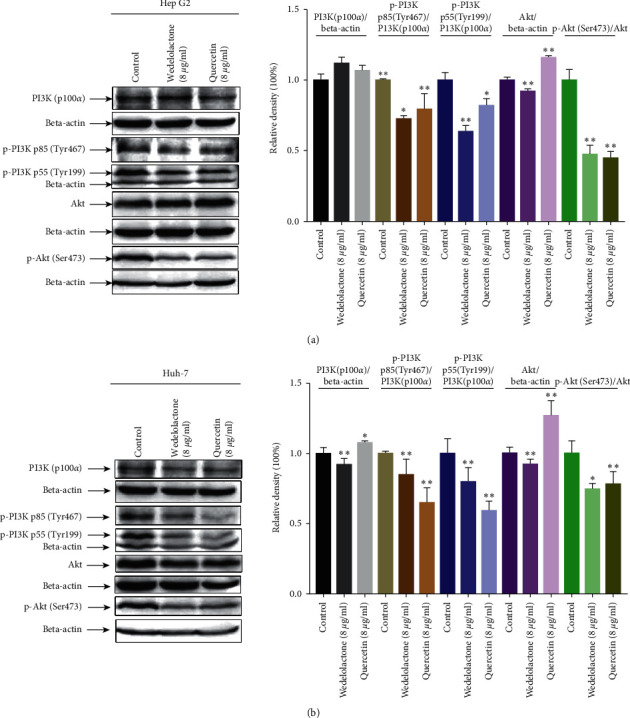
Wedelolactone and quercetin inhibited the PI3K/AKT pathway in hepatocellular carcinoma cell lines. (a) Western blot analysis showed that wedelolactone and quercetin inhibited the protein expression of p-PI3K p85 (Tyr467), p-PI3K p55 (Tyr199), and p-Akt (Ser473) in HepG2 cells. (b) Western blot analysis showed that wedelolactone and quercetin inhibited the protein expression of p-PI3K p85 (Tyr467), p-PI3K p55 (Tyr199), and p-Akt (Ser473) in Huh-7 cells. The data are expressed as the mean ± standard deviation values; *n* = 3. ^*∗*^indicates *P* < 0.05 and ^*∗∗*^ indicates *P* < 0.01 compared to the control group.

The results of the aforementioned KEGG enrichment analysis showed that 19 of the top 20 KEGG pathways were involved in PI3K-AKT signal transduction. Combined with the GO results, these findings indicated that EH can inhibit the abnormal proliferation of HCC and promote cell apoptosis by acting on the PI3K-AKT signaling pathway. Further western blot assays confirmed that 8 *μ*g/mL wedelolactone and 8 *μ*g/mL quercetin significantly inhibited the expression of p-PI3K p85 (Tyr467), p-PI3K p55 (Tyr199), and p-Akt (Ser473) in HepG2 (Figure 9(a)) and Huh-7 cells (Figure 9(b)).

## 4. Discussion

As an empirical herbal medicine widely used in China, *Ecliptae herba* has been proven to have hepatoprotective effects on damaged livers in rats and mice, but the mechanism of action and potential therapeutic targets of EH in the treatment of HCC have not been thoroughly studied [11]. Additionally, the pathological mechanism of HCC is very complicated and involves multiple targets and signaling pathways during progression. Therefore, we attempted to explore the potential mechanism of EH in the treatment of HCC through a network pharmacology-based strategy. In this study, we screened 6 active ingredients of EH: luteolin, quercetin, wedelolactone, 3′-O-methylorobol, demethylwedelolactone, and butin. These 6 active ingredients inhibit HCC by acting on 52 targets. Among these targets, some targets with high topological parameters were defined as hub genes, including RELA, MMP9, PTGS2, ESR1, EGFR, AR, AKT1, HIF1A, AHR, CYP3A4, ABCG2, and MMP2, obtained by the EH-HCC network. Additionally, by analyzing the C-T-D network, we found that quercetin plays a more complex biological function in the anti-HCC function of EH among the 6 active ingredients. Moreover, as the major component of EH, wedelolactone has been reported to have hepatoprotective effects [11]. In addition, through UPLC analysis, the content of wedelolactone in EH was shown to be 25.65% [23]. These results indicate that EH mainly exerts anti-HCC effects through quercetin and wedelolactone.

To better reveal the molecular mechanism of EH treatment of HCC, we performed enrichment analysis in this study. Through GO enrichment analysis, we found that EH produced anti-HCC activity by regulating cell proliferation (GO:0008283) and cell apoptosis (GO:0006915, GO:0042981). We concluded that the regulation of cell proliferation and apoptosis plays a vital role in the development of HCC. The effect of EH on HCC through cell proliferation and apoptotic processes involves 29 targets and 31 targets in these biological processes, respectively. These targets include AKT1, EGFR, PI3K, GSK3B, and MET. Additionally, KEGG results showed that 19 of the top 20 KEGG pathways were involved in the PI3K-AKT signaling pathway, including “hepatocellular carcinoma (hsa05225)” (Figure 6(b)). This finding suggests that EH may inhibit the abnormal proliferation of HCC and promote apoptosis by acting on the PI3K-AKT signal transduction axis. PI3K-AKT signaling is one of the key pathways in the occurrence and development of HCC, and dysregulation of this signaling pathway can lead to reduced cell proliferation and apoptosis and inhibition of tumor development [30–32]. In this pathway, EH may activate the PI3K-AKT pathway by acting on some upstream receptors, such as EGFR and MET. The PI3K-AKT pathway can affect the biological functions of HCC by mediating RELA, GSK3B, MMP, and other downstream factors. In addition, KEGG results showed that EH may inhibit the development of HCC by regulating the HIF-1 signaling pathway (hsa04066) (Figure 6(c)). Many studies have reported that inhibition of HIF1A can significantly inhibit the growth of tumors, including HCC [33, 34]. Quercetin, as an effective flavonoid component in EH, combined with specific anticancer drugs can increase the expression of p53 by downregulating the expression of HIF1A, thus inducing an increase in the proapoptotic process of liver cancer cells [35]. In addition, the expression of HIF1A can be regulated by activation of the PI3K-AKT pathway, which can be activated by RTK [36, 37].

The liver plays an important role in lipid metabolism, and excessive lipid accumulation in hepatocytes can lead to nonalcoholic fatty liver disease, which is a chronic liver disease that has emerged as a major cause of liver cancer in Western countries in recent years [38, 39]. Cytochrome P450 of the liver plays a pivotal role in the process of lipid metabolism and lipid biosynthesis [40]. Several studies have shown that regulating the activity of cytochrome P450 can affect the development of NAFLD through different mechanisms, such as oxidative stress [41–43]. By analyzing the TCGA RNASeq LIHC database and 52 targets of EH against HCC, we obtained an enriched GO term network. This GO network reveals that EH may play a role in HCC by regulating lipid metabolic process (GO:0006629), lipid biosynthetic process (GO:0008610), and cellular lipid metabolic process (GO:0044255), involving the CYP1A1, CYP1A2, and CYP3A4 gene subnetworks. These results suggest that EH may inhibit the occurrence of NAFLD by regulating the lipid metabolism process and produce hepatoprotective activity in the liver to prevent HCC.

In addition, we simulated the interaction pattern of these 6 active ingredients of EH with the hub genes by molecular docking technology, and the results showed that these molecules effectively bind to the binding pocket of these genes by forming several hydrogen bonds. However, this phenomenon does not allow them to selectively interact with specific proteins. Therefore, it is necessary to improve the selectivity of these compounds through subsequent structural modification.

Although network pharmacology has revealed the potential mechanism by which EH inhibits HCC, validation experiments are still needed to verify the above results. CCK-8 and cell apoptosis experiments confirmed that EH and its main active ingredients, quercetin, and wedelolactone, can significantly inhibit the proliferation of HCC and promote apoptosis, which is consistent with the GO analysis. To further clarify the molecular mechanism by which EH inhibits HCC, we confirmed through western blot assay that the active components of EH, quercetin, and wedelolactone can inhibit the PI3K-AKT signaling pathway in the HCC cell line. These results suggest that the pharmacological effects of EH on the inhibition of the abnormal proliferation of HCC and the promotion of HCC cell apoptosis are achieved by regulating the PI3K-AKT signaling pathway.

## 5. Conclusions

Overall, this study explored the underlying mechanism of EH against HCC based on the strategies of network pharmacology and experimental verification. These mechanisms of network pharmacology prediction mainly involve the following aspects: regulation of the PI3K-AKT signaling pathway promotes apoptosis and inhibits abnormal proliferation of HCC cells, downregulation of HIF-1A expression is mediated by activating the HIF-1 signaling pathway, and prevention of HCC is induced by regulating lipid metabolism and inhibiting NAFLD by the cytochrome P450 subfamily. Moreover, we verified through experiments that EH inhibits the PI3K-AKT signaling pathway through its active ingredients, quercetin, and wedelolactone, thereby inhibiting the proliferation of HCC cells and promoting apoptosis of HCC cells. Although the network pharmacology method can be a good solution to this issue, further experimental verification is necessary.

## Figures and Tables

**Table 1:** Pharmacokinetic parameters of 6 active ingredients of *Ecliptae herba*.

Molecule name	MW	ALog P	Hdon	Hacc	OB (%)	Caco-2	BBB	DL	HL
Luteolin	286.25	2.07	4	6	36.16	0.19	−0.84	0.25	15.94
Quercetin	302.25	1.50	5	7	46.43	0.05	−0.77	0.28	14.40
Wedelolactone	314.26	2.73	3	7	49.60	0.32	−0.45	0.48	9.61
3′-O-Methylorobol	300.28	2.05	3	6	57.41	0.45	−0.38	0.27	17.31
Butin	272.27	2.30	3	5	69.94	0.30	−0.40	0.21	16.80
Demethylwedelolactone	302.25	1.10	4	7	72.13	0.04	−0.69	0.43	9.17

**Table 2:** Detailed information on 52 targets of 6 active ingredients of EH in the treatment of HCC.

Name	Count	Gene symbol	Total
Luteolin, quercetin, wedelolactone, and HC	2	MMP2\MMP9	52
Quercetin, demethylwedelolactone, 3′-O-methylorobol, and HC	1	ESR2	
Luteolin, quercetin, and HC	6	EGFR\ALOX15\RELA\AKT1\GPR35\TOP1	
Demethylwedelolactone, wedelolactone, and HC	1	GSK3B	
Demethylwedelolactone, 3′-O-methylorobol, and HC	1	ESR1	
Butin, 3′-O-methylorobol, and HC	1	PTGS1	
Luteolin and HC	5	MET\TYR\AR\APP\PTGS2	
Quercetin and HC	31	XDH\PRKCB\CYP1A1\AHR\CYP19A1\MMP3\PIK3R1\CSNK2A1\PON1\MPO\ACHE\ABCG2CYP1A2\PIK3CG\TOP2A\HIF1A\INSR\NR1I2\YP3A4\HSPB1\F2\APEX1\AKR1B1\CYP1B1\ALOX5\E2F1\PIM1\PARP1\HSF1\BAX\PRKCA	
Demethylwedelolactone and HC	1	CBR1	
Butin and HC	1	RXRA	
3′-O-Methylorobol and HC	2	CHEK1\PRSS1	

**Table 3:** Topological parameters of the top 10 hub genes of the E-H network ranked by degree.

Gene symbol	Betweenness Centrality	Closeness Centrality	Clustering Coefficient	Topological Coefficient	Degree
AKT1	0.206016	0.761194	0.258824	0.240336	35
EGFR	0.089246	0.69863	0.337438	0.270453	29
ESR1	0.067784	0.68	0.39886	0.292665	27
PTGS2	0.085964	0.662338	0.346667	0.276078	25
AR	0.041309	0.62963	0.424242	0.313636	22
MMP9	0.040523	0.607143	0.45614	0.308421	19
RELA	0.027075	0.6	0.450292	0.330827	19
ABCG2	0.028045	0.579545	0.433333	0.306122	16
AHR	0.048945	0.593023	0.55	0.34	16
CYP3A4	0.038392	0.566667	0.358333	0.271277	16

**Table 4:** Energy and RMSD values of the compounds binding to the target obtained in molecular docking analysis.

Targets (PDB ID)	Compound	Binding energy (kcal/mol)	RMSD/LB	RMSD/UB
MMP9 (6ESM)	Luteolin	−10.6	0	0
MMP9 (6ESM)	Quercetin	−10.4	0	0
MMP9 (6ESM)	Wedelolactone	−8.8	0	0
EGFR (3W2S)	Luteolin	−8.9	0	0
EGFR (3W2S)	Quercetin	−8.8	0	0
RELA (3QXY)	Luteolin	−8.9	0	0
RELA (3QXY)	Quercetin	−8.9	0	0
AKT1 (4EKL)	Luteolin	−8.5	0	0
AKT1 (4EKL)	Quercetin	−8.7	0	0
ESR1 (4XI3)	Demethylwedelolactone	−6.8	0	0
ESR1 (4XI3)	3′-O-Methylorobol	−6.6	0	0
AR (1XJ7)	Luteolin	−8.3	0	0
PTGS2 (5IKT)	Luteolin	−9.5	0	0
AHR (5NJ8)	Quercetin	−7.4	0	0
ABCG2 (6ETI)	Quercetin	−9.1	0	0
CYP3A4 (4D75)	Quercetin	−8.9	0	0

## Data Availability

The data used in the article comes from public databases, all of which are described in the Methods section of the article. Besides, the rest of them used to support the findings of this study are included within the supplementary information files.

## Abbreviations

EH: *Ecliptae herba*
HCC: Hepatocellular carcinoma
TCM: Traditional Chinese medicine
TCMSP: Traditional Chinese Medicine Systems Pharmacology Database and Analysis Platform
ADME: Absorption, distribution, metabolism, and excretion
DGIdb: The Drug Gene Interaction Database
CTD: Comparative Toxicogenomics Database
PPI: Protein-protein interaction
NAFLD: Nonalcoholic fatty liver disease
GO: Gene Ontology
KEGG: Kyoto Encyclopedia of Genes and Genomes
HBV: Hepatitis B
HCV: Hepatitis C
EMT: Epithelial-mesenchymal transition
OB: Oral bioavailability
MW: Molecular weight
Log *P*: The logarithm of 1-octanol/water partition coefficient
Hdon: Hydrogen-bond donors
Hacc: Hydrogen-bond acceptors
DL: Drug similarity
HL: Half-life
BBB: Blood-brain barrier permeability
C-T-D: Compound-target-disease
E-H: EH target-HCC target
C-T-P: Compound-target-pathway
BP: Biological process
CC: Cellular component
MF: Molecular function
LIHC: Liver hepatocellular carcinoma
TCGA: The Cancer Genome Atlas
PDB: Protein Data Bank.

## Supplementary Materials

Supplementary Materials Supplementary File S1: a total of 48 chemical ingredients of EH were obtained from TCMSP. Supplementary File S2: detailed information of the targets of 6 active ingredients in EH was extracted from three databases, TCMSP, DGIDB, and SwissTargetPrediction. Supplementary File S3: detailed information on HCC-related targets was extracted from GeneCards and CTD. Supplementary File S4: detailed information on the PPI network of 52 potential therapeutic targets for HCC was obtained from the STRING platform. Supplementary File S5: topological parameters of nodes in the E-H network obtained from Cytoscape. Supplementary File S6: detailed information on GO enrichment analysis obtained from WebGestalt. Supplementary File S7: detailed information on the top 10 GO terms of the GO network in the TCGA RNASeq LIHC database through Network Topology-based Analysis obtained from WebGestalt. Supplementary File S8: detailed information on the top 20 KEGG enrichment pathways obtained from the WebGestalt. Supplementary File S9: detailed information on the C-T-P network obtained from Cytoscape.
