# Supplementary material for: Pharmacological Mechanisms Underlying the Hepatoprotective Effects of Ecliptae herba on Hepatocellular Carcinoma
Source: Evid Based Complement Alternat Med. 2021 Jul 16;2021:5591402. doi: 10.1155/2021/5591402 (PMC8302389; doi:10.1155/2021/5591402)
Supplement: Supplementary Materials — Supplementary File S1: a total of 48 chemical ingredients of EH were obtained from TCMSP. Supplementary File S2: detailed information of the targets of 6 active ingredients in EH was extracted from three databases, TCMSP, DGIDB, and SwissTargetPrediction. Supplementary File S3: detailed information on HCC-related targets was extracted from GeneCards and CTD. Supplementary File S4: detailed information on the PPI network of 52 potential therapeutic targets for HCC was obtained from the STRING platform. Supplementary File S5: topological parameters of nodes in the E-H network obtained from Cytoscape. Supplementary File S6: detailed information on GO enrichment analysis obtained from WebGestalt. Supplementary File S7: detailed information on the top 10 GO terms of the GO network in the TCGA RNASeq LIHC database through Network Topology-based Analysis obtained from WebGestalt. Supplementary File S8: detailed information on the top 20 KEGG enrichment pathways obtained from the WebGestalt. Supplementary File S9: detailed information on the C-T-P network obtained from Cytoscape. [file 5591402.f1.zip › 5591402.f1/Supplementary File S1.pdf]

Supplementary File S1. A total of 48 chemical ingredients of EH were collected from TCMSP

MOL ID	NAME	MW	ALogP	Hdon	Hacc	OB(%)	Caco-2	BBB	DL	FASA	HL
MOL000103	PHB	138.13	1.17	2	3	30.15	0.39	0.21	0.03	0.45	11.77
MOL000105	protocatechuic acid	154.13	0.9	3	4	25.37	0.1	-0.17	0.04	0.43	NA
MOL001790	Linarin	592.6	-0.18	7	14	39.84	-1.68	-2.77	0.71	0.27	16.07
MOL001689	acacetin	284.28	2.59	2	5	34.97	0.67	-0.05	0.24	0.35	17.25
MOL001899	ZINC02169908	268.54	6.2	0	1	23.3	1.42	1.24	0.1	0.23	NA
MOL000009	luteolin-7-o-glucoside	448.41	0.16	7	11	7.29	-1.23	-2.49	0.78	0.32	NA
MOL002854	(1R,2R,4R)-Dihydrocarveol	154.28	2.58	1	1	51.17	1.32	1.63	0.03	0.26	11.33
MOL002975	butin	272.27	2.3	3	5	69.94	0.3	-0.4	0.21	0.4	16.8
MOL003375	.alpha.-T COOH deriv.	292.42	4.91	1	2	24.52	0.82	0.5	0.13	0.12	NA
MOL003376	2-(Buta-1,3-diynyl)-5-(4-chloro-3-hydroxybut-1-ynyl) thiophene	234.71	4.47	1	1	29.61	1.3	0.49	0.06	0.46	NA
MOL003377	Demethylwedelolactone-7-glucoside	462.39	0.57	7	12	14.45	-1.5	-2.76	0.92	0.3	NA
MOL003378	1,3,8,9-tetrahydroxybenzofurano[3,2-c]chromen-6-one	300.23	2.48	4	7	33.94	0.01	-0.65	0.43	0.34	9.62
MOL003379	Demissine	1,018.34	-0.95	12	21	10.71	-3.46	-4.29	0.02	0.2	NA
MOL003380	Demissine_qt	399.73	5.52	1	2	16.02	1.25	0.89	0.75	0.17	NA
MOL003381	Ecliptasaponin B	959.26	-0.08	12	19	1.64	-3.4	-4.38	0.06	0.25	NA
MOL003382	Ecliptasaponin B_qt	472.78	5.32	3	4	17.26	0.18	-0.43	0.74	0.25	NA
MOL003383	Ecliptasaponin D	634.94	3.57	6	9	5.81	-1.03	-1.74	0.34	0.26	NA
MOL003384	Ecliptasaponin D_qt	472.78	5.32	3	4	16.43	0.14	-0.65	0.74	0.25	NA
MOL003385	Ecliptasaponin	634.94	3.57	6	9	17.72	-0.97	-1.8	0.34	0.23	NA
MOL003386	Ecliptasaponin_qt	472.78	5.32	3	4	17.47	0.22	-0.4	0.74	0.25	NA
MOL003387	3-[(2S)-2,3-dihydroxy-3-methyl-butyl]-4-methoxy-1-methyl-carbostyril	291.38	0.71	2	5	15.4	0.25	-0.01	0.19	0.27	NA
MOL003388	Isodesacetyluvaricin	396.58	3.43	2	6	28.16	-0.25	-0.64	0.48	0.24	NA
MOL003389	3'-O-Methylorobol	300.28	2.05	3	6	57.41	0.45	-0.38	0.27	0.32	17.31
MOL001232	TES	288.47	3.33	1	2	12.93	0.68	0.29	0.35	0.26	NA
MOL003391	alpha-Terthienyl methyl acetate	320.48	5.08	0	2	33.61	1.09	0.8	0.17	0.06	-1.88
MOL003392	.alpha.-T OHMe deriv.	278.44	4.71	1	1	29.97	0.99	0.52	0.11	0.03	NA
MOL003393	(1S,4S)-7-isopropylidene-1,4-dimethyl-2,3,4,5,6,8-hexahydro-1H-azulene	204.39	5.13	0	0	24.38	1.86	2.07	0.07	0.23	NA
MOL003394	Benzofurano(3',2':3,4)coumarin	236.23	3.55	0	3	29.11	1.22	0.73	0.25	0.41	NA
MOL003395	(3S,8S,9S,10R,13R,14S,17R)-17-[(1R,4R)-1,4-dimethylhexyl]-10,13-dimethyl-2,	386.73	7.38	1	1	11.33	1.45	1.19	0.67	0.21	NA
MOL003396	echinocystic acid	472.78	5.32	3	4	24.43	0.09	-0.43	0.74	0.24	NA
MOL003397	(3S,8S,9S,10R,13R,14S,17R)-17-[(E,1R,4R)-1,4-dimethylhex-2-enyl]-10,13-dim	384.71	6.93	1	1	15.33	1.43	1.06	0.68	0.22	NA
MOL003398	Pratensein	299.27	1.37	2	6	39.06	0.39	-0.09	0.28	0.06	17.13
MOL003399	Caulophyllogenin	488.78	4.23	4	5	29.74	-0.37	-0.9	0.72	0.23	NA
MOL003400	Ecliptasaponin A	634.94	3.57	6	9	8.62	-1.11	-1.84	0.34	0	NA
MOL003401	Ecliptasaponin A_qt	472.78	5.32	3	4	15.58	0.18	-0.44	0.74	0	NA
MOL003402	demethylwedelolactone	302.25	1.1	4	7	72.13	0.04	-0.69	0.43	0.34	9.17
MOL003403	nicotine	162.26	1.24	0	2	77.67	1.38	1.53	0.04	0.25	3.99
MOL003404	wedelolactone	314.26	2.73	3	7	49.6	0.32	-0.45	0.48	0.29	9.61
MOL003405	Chloromaloside	1,345.61	-5.6	18	32	7.59	-5.42	-6.94	0.01	0.23	NA
MOL003406	Chloromaloside_qt	448.71	2.98	3	5	11.87	-0.27	-1.1	0.86	0.24	NA
MOL000361	Amyrin	426.8	7.3	1	1	17.6	1.45	1.29	0.76	0.22	NA
MOL000421	nicotinic acid	123.12	0.28	1	3	47.65	0.34	0.21	0.02	0	11.98
MOL000460	(Z)-1-(2,4-dihydroxyphenyl)-3-(3,4-dihydroxyphenyl)prop-2-en-1-one	272.27	2.63	4	5	83.78	0.1	-0.46	0.17	0	19.88
MOL002003	(-)-Caryophyllene oxide	220.39	3.52	0	1	32.67	1.58	1.76	0.13	0.28	6.51
MOL000006	luteolin	286.25	2.07	4	6	36.16	0.19	-0.84	0.25	0.39	15.94
MOL000007	Cosmetin	432.41	0.43	6	10	9.68	-1.08	-2.26	0.74	0.32	NA
MOL000008	apigenin	270.25	2.33	3	5	23.06	0.43	-0.61	0.21	0.41	NA
MOL000098	quercetin	302.25	1.5	5	7	46.43	0.05	-0.77	0.28	0.38	14.4
