# Supplementary material for: Pharmacological Mechanisms Underlying the Hepatoprotective Effects of Ecliptae herba on Hepatocellular Carcinoma
Source: Evid Based Complement Alternat Med. 2021 Jul 16;2021:5591402. doi: 10.1155/2021/5591402 (PMC8302389; doi:10.1155/2021/5591402)
Supplement: Supplementary Materials — Supplementary File S1: a total of 48 chemical ingredients of EH were obtained from TCMSP. Supplementary File S2: detailed information of the targets of 6 active ingredients in EH was extracted from three databases, TCMSP, DGIDB, and SwissTargetPrediction. Supplementary File S3: detailed information on HCC-related targets was extracted from GeneCards and CTD. Supplementary File S4: detailed information on the PPI network of 52 potential therapeutic targets for HCC was obtained from the STRING platform. Supplementary File S5: topological parameters of nodes in the E-H network obtained from Cytoscape. Supplementary File S6: detailed information on GO enrichment analysis obtained from WebGestalt. Supplementary File S7: detailed information on the top 10 GO terms of the GO network in the TCGA RNASeq LIHC database through Network Topology-based Analysis obtained from WebGestalt. Supplementary File S8: detailed information on the top 20 KEGG enrichment pathways obtained from the WebGestalt. Supplementary File S9: detailed information on the C-T-P network obtained from Cytoscape. [file 5591402.f1.zip › 5591402.f1/Supplementary File S2.pdf]

Luteolin				Quercetin				Demethylwedelolactone		
TCMSP	DGIdb	SwissTargetPrediction		TCMSP	DGIdb	SwissTargetPrediction		TCMSP	DGIdb	SwissTargetPrediction
MET	HPGD	NOX4		PTGS1	ESR2	NOX4		PTGS2	MAPK3	ESR2
PTGS1	KDM4E	AKR1B1		AR	HSF1	AVPR2		GSK3B	MMP9	ESR1
AR	CYP3A4	CDK5R1 CDK5		PPARG	PIK3R5	AKR1B1		HSP90	NFKBIA	ALOX5
PTGS2	GAA	XDH		PTGS2	NR1I2	XDH			MAPK1	CBR1
PRSS1	FEN1	MAOA		NCOA2	PIK3CG	MAOA			MMP2	GSR
NCOA2	RAPGEF3	FLT3		AKR1B1	APEX1	IGF1R				SRC
RELA	POLI	CA2		PRSS1	HSPB1	FLT3				CCND1
EGFR	TNFRSF10B	CCNB3		KCNH2	FEN1	CYP19A1				PDGFRB
AKT1	POLB	ALOX5		SCN5A	F2	EGFR				FLT4
VEGFA	NFKB1	ADORA1		ADRB2	PRKCA	F2				CDK2
CCND1	SMN1	CA7		MMP3	GABPA	CA2				PLK4
BCL2L1	RECQL	GLO1		F7	PRKD1	PIM1				TEK
CDKN1A	CYP1A2	APP		RXRA	CYP2C19	ALOX5				AURKA
CASP9	SMN2	SYK		ACHE	PIK3CB	AURKB				MAP3K8
MMP2	ALOX15	GSK3B		GABRA1	ESR1	DRD4				BRAF
MMP9	BAZ2B	PARP1		MAOB	NFKB2	ADORA1				EPHB4
MAPK1	BLM	TTR		RELA	E2F1	CA7				SQLE
IL10	NFKB2	MMP9		EGFR	CYP3A4	GLO1				FGR
RB1	WRN	CA12		AKT1	NFKB1	MPO				LYN
TNFSF15	EHMT2	MMP2		VEGFA	PRKCQ	PIK3R1				AKR1B1
JUN	MAPT	CA4		CCND1	HCK	ADORA2A				HSPA1A
IL6	RELA	MMP12		BCL2	PIK3CA	DAPK1				FASN
CASP3	HSD17B10	CD38		BCL2L1	APOB	PYGL				CSNK2A1
TP63	KDM4A	CYP1B1		FOS	BAX	CA1				GPR35
NFKBIA	GPR35	ABCG2		CDKN1A	DRD1	GSK3B				EGFR
TOP1	TYR	AKR1B10		EIF6	CEBPB	SRC				TNNC1
MDM2	POLK	TNKS2		BAX	GALK1	PTK2				IGF1R

APP	ALOX15B	TNKS		CASP9	ATP5C1	HSD17B2				CA2
MMP1	ALDH1A1	TOP1		PLAU	CDC25C	KDR				AURKB
PCNA	APEX1	ARG1		MMP2	AHR	MMP13				CA7
ERBB2	CYBB	PTPRS		MMP9	PRKCI	MMP3				CA1
PPARG	POLH	ABCC1		MAPK1	PIK3R3	CA3				GSK3B
HMOX1		HSD17B1		IL10	GSR	ALOX15				PTK2
CASP7		ACHE		EGF	PRKCZ	ABCC1				KDR
ICAM1		CDK6		RB1	PRKD2	PLK1				PLK1
MCL1		ABCB1		TNFSF15	STK17B	CA6				CA6
BIRC5		HSD17B2		JUN	CYP1A1	CDK1				CA12
IL2		ALOX15		IL6	HSP90AA1	MMP9				CA14
CCNB1		ALOX12		AHSA1	ATP5B	CA12				CA9
TYR		ESR2		CASP3	KDM4E	MMP2				MET
IFNG		CYP19A1		TP63	ALOX15	PKN1				CA4
IL4		ADORA2A		ELK1	PIK3CB	CA14				CA13
TOP2A		CSNK2A1		NFKBIA	XDH	CA9				AKT1
GSTP1		ESR1		POR	PRKCG	CSNK2A1				CA5A
SLC2A4		PTGS2		ODC1	POLK	ALOX12				BACE1
INSR		CFTR		CASP8	ATAD5	MET				NUAK1
CD40LG		AMY1A		TOP1	PIK3C2G	CA4				XDH
PTGES		GRK6		RAF1	EHMT2	NEK2				INSR
NUF2		CDK2		SOD1	AVPR2	CXCR1				KCNMA1
ADCY2		TERT		PRKCA	UGT3A1	CAMK2B				ERBB2
		CA1		MMP1	CSNK2B	ALK				FNTA FNTB
		CA9		HIF1A	PIK3R2	AKT1				NFKB1
		CDK1		STAT1	PRKD3	ABCB1				MAOA
		TYR		RUNX1T1	RECQL	NEK6				FEN1
		AHR		ERBB2	CBR1	PLA2G1B				TERT
		ESRRA		PPARG	HPGD	CA5A				EIF4H
		GPR35		ACACA	HIF1A	BACE1				PABPC1
		DAPK1		HMOX1	PRKCB	CYP1B1				HSPA8
		AVPR2		CYP3A4	EIF3F	AXL				CDC25A

		IGF1R		CYP1A2	PKN3	ABCG2				CDC25B
		EGFR		CAV1	POLB	NUAK1				SNCA
		F2		MYC	PRKCD	AKR1C2				CA5B
		PIM1		F3	CYP19A1	AKR1C1				HSD17B3
		AURKB		GJA1	NQO2	AKR1C3				KCNA5
		DRD4		CYP1A1	ACTB	AKR1C4				KCNA3
		MPO		ICAM1	CSNK2A1	CA13				BCL2L1
		PIK3R1		IL1B	PKN2	AKR1A1				DAO
		PYGL		CCL2	TOP1	GPR35				DUSP3
		SRC		SELE	PIK3C2A	SYK				DAPK1
		PTK2		VCAM1	MAPK3	MAPT				PIK3CG
		KDR		PTGER3	CD34	KDM4E				MPG
		MMP13		CXCL8	XDH	TOP2A				PIK3CA
		MMP3		PRKCB	POLH	INSR				SLC22A12
		CA3		BIRC5	MAPT	ACHE				IGFBP6
		PLK1		DUOX2	PKN1	MYLK				IGFBP4
		CA6		NOS3	MBP	PIK3CG				IGFBP5
		PKN1		HSPB1	PIK3CA	APEX1				IGFBP2
		CA14		SULT1E1	PIK3CD	CDK5R1				IGFBP3
		MET		MGAM	PIK3R1	CCNB3				IGFBP1
		NEK2		IL2	CYP2C9	ARG1				CYP1A2
		CXCR1		NR1I2	SF3B3	PTPRS				AVPR2
		CAMK2B		CYP1B1	PIK3CG	ESR2				F2
		ALK		CCNB1	GPR35	MPG				DRD4
		AKT1		PLAT	CYP1B1	SLC22A12				GLO1
		NEK6		THBD	UBA1	CDK6				MPO
		PLA2G1B		SERPINE1	PRKCE	CDK2				PIK3R1
		CA5A		COL1A1	HSPA2	TYR				PYGL
		BACE1		IFNG	TOP2A	HSD17B1				MMP13
		AXL		ALOX5	ALDH1A1	AHR				MMP3
		NUAK1		IL1A	CYP1A2	ESRRA				MMP9

		AKR1C2		MPO	ABL1	APP				MMP2
		AKR1C1		TOP2A	PIK3C3	PARP1				PKN1
		AKR1C3		NCF1	HIBCH	TTR				NEK2
		AKR1C4		ABCG2	HSD17B10	MMP12				HSP90AA1
		CA13		HAS2	PRKCH	CD38				CXCR1
		AKR1A1		GSTP1	POLI	AKR1B10				CAMK2B
		PFKFB3		NFE2L2	PON1	TNKS2				NEK6
		KDM4E		NQO1	PIK3C2B	TNKS				PLA2G1B
		PLG		PARP1	ALOX15B	TOP1				AKR1C2
		AR		AHR	MPHOSPH8	TERT				AKR1C4
				PSMD3	PIK3CA					
				SLC2A4	PIK3R4					
				COL3A1	BLM					
				CXCL11	RELA					
				CXCL2	YES1					
				DCAF5	PIK3R6					
				NR1I3	ATP5A1					
				CHEK2	RUVBL2					
				INSR	SHBG					
				CLDN4	PIM1					
				PPARA						
				PPARD						
				HSF1						
				CRP						
				CXCL10						
				CHUK						
				SPP1						
				RUNX2						
				RASSF1						
				E2F1						
				E2F2						

				ACPP						
				CTSD						
				IGFBP3						
				IGF2						
				CD40LG						
				IRF1						
				ERBB3						
				PON1						
				DIO1						
				PCOLCE						
				NPEPPS						
				HK2						
				RASA1						
				GSTM1						
				GSTM2						

Wedelolactone				Butin				3'-O-Methylorobol		
TCMSP	DGIdb	SwissTargetPrediction		TCMSP	DGIdb	SwissTargetPrediction		TCMSP	DGIdb	SwissTargetPrediction
ESR1	CBR1	ALOX5		PTGS1	/	CYP19A1		NOS2	/	ABCB1
PPARG	THRB	CBR1		PTGS2		CA7		PTGS1		CA7
ESR2	APEX1	ESR1		RXRA		CA12		ESR1		CA12
GSK3B	BLM	ESR2				CA4		AR		CA4
IKBKB	CBFB	CA12				CYP1B1		PPARG		CYP19A1
AR	MAPK3	CA9				ABCC1		PTGS2		HSD17B2
	NFKBIA	CA13				HSD17B1		ESR2		CBR1
	MAPK1	CA5B				SHBG		MAPK14		EGFR
	RECQL	GSR				CBR1		GSK3B		PTPRS
	RUNX1	AKR1B1				ESR1		CHEK1		TYR
	POLK	KCNA5				ESR2		PRSS1		MIF
		KCNA3				MAOB		CCNA2		CA2
		CA7				PTGS1		NCOA1		XDH
		CA1				TAS2R31				ESR1

		ERBB2				MMP13				ESR2
		CCND1				ABCG2				IL2
		PDGFRB				MMP12				PPARA
		FLT4				ADORA1				ALOX12
		CDK2				ADORA3				TLR9
		PLK4				AKR1C3				HSD17B1
		TEK				GRM5				TBXAS1
		AURKA				PLA2G1B				MAOA
		MAP3K8				KLK1				MGAM
		BRAF				KLK2				HTR2A
		EPHB4				CES1				HTR2C
		SQLE				PPARG				ADORA1
		FGR				CES2				ADORA2A
		LYN				KIT				ESRRA
		MAOA				SRC				ESRRB
		KCNMA1				KDR				ABCG2
		IGF1R				FGFR1				ALDH2
		AURKB				MET				MAOB
		SRC				ALOX12				NOX4
		PTK2				SLC5A2				PTGS1
		KDR				CA2				SLC6A2
		PLK1				CA1				ALOX15
		MET				POLB				CA1
		AKT1				CA3				PFKFB3
		NUAK1				CA6				ABCC1
		FEN1				CA13				CYP1B1
		EGFR				CA5B				CDC7
		CA6				CA5A				ACHE
		CA14				PLA2G2A				IGFBP3
		CA4				PLA2G5				MCL1
		CA5A				PLA2G10				PON1
		PFKFB3				DYRK1A				PTPN1

		PIK3CG				CA9				AKR1B1
		PIK3CA				CHRNA7				SNCA
		DUSP3				APP				PLAT
		TERT				ACHE				F10
		CYP1A2				BACE1				PLAU
		SRD5A1				MMP2				IGFBP6
		GPR35				RXRA				IGFBP4
		HDAC3				HSD17B2				IGFBP5
		HDAC6				BCL2				IGFBP2
		HDAC2				ESRRA				IGFBP1
		HSD17B1				ESRRB				STS
		HDAC8				SERPINE1				DUSP3
		HDAC1				YWHAG				CDC25B
		PKN1				NOX4				HSP90AB1
		NEK2				GSK3B				PTK6
		NEK6				HSD17B14				TBXA2R
		HDAC10				IGFBP3				CA5B
		PRKCA				CLK1				BAD
		PRKCB				DYRK1B				ALOX5
		PRKCE				DNMT1				CHEK1
		XDH				ST3GAL3				WEE1
		AMY1A				FUT7				TNF
		CA2				FUT4				PLG
		GSK3B				STAT1				BACE1
		CSNK2A1				SQLE				CA3
		BACE1				MMP9				CA6
		DHODH				MAPT				CA14
		F2				ERN1				CA9
		FNTA				IGF1R				CA13
		PRSS1				INSR				CA5A
		F10				TERT				GCGR
		PLAU				LCK				CRHR1

		NOS2				SYK				PRMT1
		NOX4				NQO2				PRSS1
		FASN				MAOA				IGF1R
		HSPA1A				FFAR1				CXCR2
		PTGS2				HIF1A				TNNC1
		SLC22A12				CDK5R1				CDK5R1
		INSR				AKT1				CCNB3
		PTPN1				VCP				ARG1
		COMT				AKR1B1				HSP90B1
		KDM4E				ABCB1				KLKB1
		CDK1				CDK2				CCR4
		APP				CDC7				PLA2G2A
		SNCA				CTSB				FNTA
		DAPK1				PTPN1				SORT1
		MPG				F3				EGLN1
		EIF4H				ADCY5				APP
		PABPC1				ODC1				HSP90AA1
		EGLN1				PDPK1				PARP1
		MAOB				WEE1				TNKS2
		MMP9				EDNRA				TNKS
		MMP2				PARP1				CDK6
		IGFBP3				SNCA				CHEK2
