# Supplementary material for: Pharmacological Mechanisms Underlying the Hepatoprotective Effects of Ecliptae herba on Hepatocellular Carcinoma
Source: Evid Based Complement Alternat Med. 2021 Jul 16;2021:5591402. doi: 10.1155/2021/5591402 (PMC8302389; doi:10.1155/2021/5591402)
Supplement: Supplementary Materials — Supplementary File S1: a total of 48 chemical ingredients of EH were obtained from TCMSP. Supplementary File S2: detailed information of the targets of 6 active ingredients in EH was extracted from three databases, TCMSP, DGIDB, and SwissTargetPrediction. Supplementary File S3: detailed information on HCC-related targets was extracted from GeneCards and CTD. Supplementary File S4: detailed information on the PPI network of 52 potential therapeutic targets for HCC was obtained from the STRING platform. Supplementary File S5: topological parameters of nodes in the E-H network obtained from Cytoscape. Supplementary File S6: detailed information on GO enrichment analysis obtained from WebGestalt. Supplementary File S7: detailed information on the top 10 GO terms of the GO network in the TCGA RNASeq LIHC database through Network Topology-based Analysis obtained from WebGestalt. Supplementary File S8: detailed information on the top 20 KEGG enrichment pathways obtained from the WebGestalt. Supplementary File S9: detailed information on the C-T-P network obtained from Cytoscape. [file 5591402.f1.zip › 5591402.f1/Supplementary File S3.pdf]

Hepatocellular carcinoma	
**GeneCards**	**CTD**
TP53	CCND1
MET	CASP8
CTNNB1	NFE2L2
PIK3CA	JUN
APC	PARP1
CDKN2A	MYC
EGFR	TNF
HRAS	CCNB1
PTEN	MKI67
AKT1	CYP1A1
KRAS	CYP1A2
CDH1	IL6
ERBB2	PCK1
CCND1	TGFB1
TERT	MMP2
BRAF	EGR1
TGFBR2	EGFR
MSH2	GSTM1
CDKN1A	CTNNB1
SMAD4	HSPB1
NRAS	SOD2
EPCAM	PTGS2
MTOR	CDK1
BRCA1	VCAM1
CASP8	RB1
NFE2L2	IGF1
HNF1A	EPHX1
FAS	FOS
CDKN1B	TOP2A
AXIN1	TRP53
H19	CCNE1
RB1	EGF
MLH1	SREBF1
BAX	AFP
FASLG	CDKN1B
EGF	CXCL12
FHIT	CDK4
KIT	PTEN
RET	CYP2E1
FGFR1	GDF15
ESR1	ESR1
MIR21	CEBPB
CDKN3	HSPA5
MDM2	MMP9
STK11	CEBPA
IGF2	RRM2
PTCH1	SLC2A2
VEGFA	GSTP1
MMP1	ACACA
IGF2R	FASN
RAF1	CDC20
MSH6	HMGCR
STAT1	STAT1
MIR145	CCNB2
MIR17	MAD2L1

IDH1	E2F1
MIR31	PDK4
BRCA2	DCN
GNAS	IGF1R
MYC	SLC2A1
MIR221	AURKA
MIR34A	CTSD
PPARG	HGF
CDKN2B	IRS1
MIR143	MET
SRC	HRAS
FGFR2	MPO
TNFRSF10B	BTG2
MIR200A	CBR1
MIR195	RPS6
MIR155	UBE2C
TGFBR1	NR1H4
FGFR4	ACOX1
HIF1A	BID
MIR141	ME1
STAT3	IGF2
KRT7	MCM2
SMARCB1	CCN1
MAP2K1	HMGB2
HNF4A	NUSAP1
MIR126	CDKN3
AR	ISG15
TP73	UBD
MIR27A	ABCB4
MIR214	GHR
SMARCA4	TLR4
MIR146A	PKM
IDH2	CCL3
MIR200C	NR0B2
AFP	ABCB1
AURKA	ANXA4
IL1B	HSPA9
MIR125A	MTOR
TWIST1	SCD
MTUS1	KRAS
JAG1	PDIA3
HFE	PTTG1
PTGS2	CDC25C
KEAP1	RRM1
KRT19	APOA1
TERC	DPYD
BIRC5	MT1A
MIR29C	CDKN2A
MIR150	CEBPD
MIR200B	TYMS
CHEK2	ACTB
MIR205	CP
MIR204	RARA
TGFB1	ENO1
OGG1	GJB1
MIR122	TGFA
MIR193A	CENPE
MIR140	GLUL

MIR22	KIF2C
MIR106B	PLK1
MMP9	CENPF
MIR182	CDC6
MIR483	USP2
MIR148A	PHLDA1
CD44	F2
MIR222	IL1RN
MIR203A	UHRF1
MIR185	SOCS3
MIR224	LIFR
MIR30E	PTK2
IL6	CYP17A1
BCL2	STMN1
ATM	PRDX2
MIRLET7A1	TK1
CASP3	CENPA
MIR10B	KIF20A
MUC1	DLGAP5
MIR93	PGD
MIR15A	ACLY
AXIN2	PBK
PMS2	IGFALS
PTPN11	PZP
SERPINA1	AKR1B10
KCNQ1OT1	PRDX1
MIR183	BUB1
MMP2	LCAT
MIR223	FABP5
NFKBIA	MAT1A
SPRTN	NEK2
TSC2	APEX1
MIR18A	SFN
MIR23A	MT2A
KLF6	APCS
MIR181A1	A2M
MIR192	CRP
MIR486-1	RAC1
MIR23B	PRC1
MIR29A	SOCS2
MAPK1	GAPDH
TSC1	EZH2
MIR26A1	FST
MIR191	NME1
IFNG	RCAN1
GSTP1	FDFT1
MIR20A	MAPT
FAH	CXCL14
MIR15B	AURKB
JUN	CDCA8
BCL2L1	ATG7
MIR96	ECM1
HGF	ADH4
TCF4	SLC22A1
HNF1B	TPX2
CXCL8	ZWINT
MIR127	SPC25
CD274	EGR2

KDR	TTK
MIRLET7G	IRS2
MIRLET7D	INMT
MIR34C	GMNN
MKI67	ATM
TNF	NCAPH
MIR19A	MCM10
NOTCH1	RACGAP1
MIR107	TRIM24
ABCB4	ACO2
PDGFRL	GNMT
CDKN1C	TERT
MIR25	BUB1B
TGFA	GLI1
RASSF1	SKP2
MIR16-1	FOXM1
NR1H4	CDCA5
CASP9	CCNF
MIR199B	HAO2
CEACAM5	NDC80
G6PC	CHAF1B
KRT18	IL1RAP
KRT8	CDCA3
CDK4	AADAT
MIRLET7B	MYBL2
GPC3	CDKN2C
MAPK3	APC
MIR324	NUF2
CREBBP	ERP29
IGF1R	THY1
SDHB	ESM1
MEN1	FBP1
GSTM1	PRDX6
MUTYH	ECT2
MIRLET7E	IDH1
MAPK8	TRIP13
DLC1	ETFA
NME1	TH
PEG10	ASF1B
FGF2	CD34
MIRLET7C	TAGLN2
EP300	NNMT
MIR128-2	FOSB
ABCB1	DTL
NFKB1	HMMR
ITGB4	KIF23
TNFSF10	UCHL1
VHL	PYGL
MIR130A	MVK
VIM	E2F8
MIR137	CD163
MIR24-2	CEP55
IL2	ANLN
HULC	NCAPG
MIR196A2	PDGFB
VEGFC	ASPM
ALB	CDH13
ERBB3	ACSL4

SPP1	TFPI2
CYP1A1	MECP2
SLC2A1	PIK3CA
EZH2	CD5L
MIR181B1	CYP2B6
MIRLET7A3	EHD3
IL12RB1	NAT2
TYMP	MELK
IFNA1	TCF19
INS	RND3
FGFR3	PLAC8
CXCR4	COMT
JAK2	C9
RARB	CDC45
FLT1	KIF11
BMPR1A	CA2
YAP1	CYP39A1
BMP2	ADAMTS1
TLR2	TALDO1
MIR181A2	PHB
ABCB11	HAMP
MIR152	LRAT
RAC1	CCNA1
PTK2	GTSE1
SYP	KMT2A
MAGEA3	UBE2T
WRAP53	OLFML2B
HTATIP2	STAT4
MAGEA1	RPS6KA3
PRKACA	CDCA2
IL10	CFP
SMAD2	COL15A1
TIMP2	SERPINA1
MMP7	IRF2
MLH3	PGK1
PLAU	AKR1C2
CCNA2	ITIH3
HOTAIR	SFRP1
RHOA	PCLAF
SMAD3	KIF15
MALAT1	KIF14
PARP1	CCR1
MIR199A1	TACC3
DNMT1	TST
CYCS	ROBO1
MIR215	MLXIPL
NF1	FANCI
PALB2	IQGAP1
CDH2	ZFP36
CDK2	HHIP
CTTN	IGF2R
IFNL3	ARHGAP11A
PTPRC	BMPER
MAPK14	SREBF2
NKX2-1	VASN
MIR181C	PDGFRL
CAV1	KIFC1
CCNB1	CSPG4

HEPN1	UMPS
CDK6	CENPU
CXCL12	SRPX
SNAI1	GPC3
NBN	BRAF
IGF1	YAP1
SERPINB3	KIF4A
PLAUR	NR1H2
HSPB1	HGFAC
PMS1	SHH
E2F1	SLC25A47
ITGB1	MED1
CHGA	PTH1R
MGMT	GYS2
XRCC3	YTHDF2
MMP14	FANCD2
MME	RAD54L
BSG	BCO2
TIMP1	CELSR3
NQO1	SLIT2
ABCC1	IQGAP3
TYR	LYVE1
XIAP	UBE2E2
PRKAR1A	MFSD2A
PDGFRA	CYLD
MCL1	ARID1B
HSP90AA1	PAMR1
SERPINA3	PYCARD
IFI27	KMT2C
RXRA	CDT1
CFLAR	IQGAP2
MIR30A	INPP4B
NOS2	TROAP
WRN	TSC1
PIK3R1	TCIM
DNMT3B	HSD3B2
ARID1A	DEPDC1B
ANXA5	CENPM
CDK1	KCNN2
TIMP3	APLN
XRCC1	SKA1
JAK1	TLR9
MEG3	TSLP
UCA1	IGF2BP3
SNAI2	ASPG
MIR139	KBTBD11
PDGFRB	APOF
IFNA2	NOTCH3
MAGEC2	EXO1
GSK3B	KIF18A
MSH3	CSRNP1
ABCG2	CDK14
AREG	CENPW
STARD13	ORC1
PROM1	PLVAP
PVT1	ARID2
SP1	ADD1
ZEB1	EAF2

CALCA	LRRC59
PIK3CG	PKMYT1
TNFRSF10A	PPP1R1A
BAK1	MBTPS1
GRB2	CYP2C8
HPSE	MARCO
PRKCA	FGF19
AKT2	GINS1
TUG1	TRAIP
PCNA	ANGPTL6
F2	RTP3
SDHD	FGF4
LRRC56	GPM6A
MIR181B2	CKAP2L
LEF1	MT1E
ANXA2	UROC1
GGT1	FAM83D
GAPDH	SCAP
IL6R	DEPDC1
GAS5	AXIN1
PRKCD	CPEB3
SDHC	NEIL3
CTAG1B	RUNX3
PKM	CD274
URGCP	OLFML2A
AHCY	TMEM70
E2F2	MIR122
MIR149	COLEC10
H2AC18	PKP1
SPARC	STAB2
CCNE1	GNAO1
POU5F1	COPS5
MIR9-1	PNPT1
FGL1	KMT2B
BAD	HERC5
CCR6	CYP4A11
SKP2	TLR7
ENG	HJURP
CDKN2B-AS1	JDP2
EPO	VIPR1
BIRC3	WDR62
CASP10	PITX1
SOD2	GPR182
RPS6KB1	CAP2
KRT5	PARK7
WNT5A	MBTPS2
MIR142	CNDP1
CASP7	RSPO3
TCF7L2	EPS8L3
CCAT1	HOXA10
MIR144	VSIG4
NEAT1	IGF2BP1
SIRT1	ZIC2
CYP2E1	MRO
CTLA4	ARID1A
MIR101-1	KDM8
ABCC2	CEP131
GPT	MICA

TJP2	CCBE1
AKT3	DNAJC6
MIR451A	WDR76
SOS1	GDF2
PIK3CB	MT1F
WNT1	SLC26A6
BMP6	EIF3H
SHC1	OIT3
SETD2	TTC36
SPRY4-IT1	ARHGEF39
SFRP1	CLEC4G
FOS	FAM111B
AKR1B10	LRRC1
MIR24-1	HOTAIR
EPAS1	DNASE1L3
FOXM1	MOGAT2
LINC-ROR	ATP5PD
E2F3	CETP
TLR4	CLEC1B
DPYD	ADRA1A
CYP17A1	FBXL18
RUNX3	ADRA2B
PDCD1	SKA3
ZFAS1	TIMD4
ERCC6	HOXD9
UROD	MTBP
ZEB2	RNF157
ICAM1	KIF18B
CD82	FATE1
ZC4H2	CRHBP
HEIH	MSH5
EZR	FLVCR1
CASC2	HOXA13
MSR1	DIRAS3
CLTC	GBA3
MIR196A1	ADAMTS13
EGR1	DEPDC5
CDX2	GNAZ
ANGPT2	ZNF23
POLE	CNTNAP4
TGFB2	TONSL
ALDH2	IGBP1
MIR99A	TGM3
XIST	PITPNM3
PHF20	PLXDC1
NTRK1	ZIC5
SMARCA2	MAGEA1
CD34	CLEC4M
CYTOR	DBH
HSPA5	CLTRN
CCAT2	MAGEA3
MIR424	GPR158
MIR338	SPRTN
PCAT1	SLC22A10
ETS1	GABRD
FASN	C14ORF180
BECN1	FGF3
TGFB3	NLRC3

CCL2	ZIC4
RNASEL	MAGEA6
DNAJB1	XAGE1B
SQSTM1	CSMD1
KRT14	ADGRG7
UCHL1	COX7B2
MIR423	LPA
HOTTIP	TEDC2
SOCS1	TICRR
MAGEA4	LILRB5
PIK3R3	FAM180A
MTA1	CCL14
CHP2	FCN2
MIR490	SSX1
MIR429	FAM72B
HSPA4	FCN3
HDAC1	MAGEC2
SMAD7	MIR615
PIK3R2	DIPK2B
PTK2B	FAM65C
FOXP3	TATDN1
NANOG	DCAF4L2
ENPP2	MIR520B
PXN	PPP4R3C
FGF3	FAM134B
SF3B2	RBMY
MVP	INS-IGF2
WNT3A	LINC00221
CCN2	NKILA
MIR138-1	SPRTN
CD80	TP53
IFNB1	BCL2L1
DKK1	BIRC5
HMOX1	PPARG
ATP7B	TNFSF10
BARD1	NFKBIA
HAMP	AR
ENO1	CXCL8
IRS1	HTATIP2
MIR133B	SERPINA4
ACTB	ACE
WNT3	IFNA1
SOX2	SLC5A5
FZD7	CD276
TP63	THEM4
SULF1	LETM1
STMN1	MIR885
GREM1	MIR539
PLCG1	CDKN1A
PTPRH	PCNA
HEPACAM	BAX
POLH	CASP3
IKBKB	NFKB1
DANCR	BCL2
ARID2	FAS
MIR124-1	ABCC3
PANDAR	MDM2
MIR29B1	RELA

KRT20	CASP9
MIR30D	IL1B
HMBS	CAT
PNPLA3	H2AX
MIR296	CDH1
CRNDE	ALDH1A1
GAST	NQO1
RSF1	CD14
ABCC3	ABCC2
MIR197	GADD45A
SSTR2	COL1A1
EPHX1	GPX1
RRM2	KRT8
MRE11	GSR
TPX2	DNMT1
MIR212	IFNG
LARP1	CD36
IGF2BP2	MAPK1
UBD	GSTA2
MIR375	VIM
PRKCB	GPT
PAX8	CCNG1
RECK	GSK3B
HK2	GSTM3
PGR	COL1A2
GLUL	VEGFA
PTHLH	DDIT3
SLC22A18	TIMP1
PBRM1	ATF3
DIABLO	TRIB3
CEACAM1	PPARA
ERCC2	SCD1
GHET1	ACTA2
PODXL	AKT1
IGFBP3	CDK2
SNHG1	SOD1
TFE3	POR
LINC00261	NR3C1
MIR455	ABCG2
SOCS3	STAT3
WNT2	MAPK8
AFAP1-AS1	CASP7
MIR125B1	MGMT
RAD51C	HMOX1
CXCR1	GCLC
HNF1A-AS1	CCNA2
HMGA2	MAPK3
PTENP1	GADD45B
SST	IGFBP3
AURKB	ICAM1
GJB1	SPP1
MDK	CCN2
RHOC	APAF1
CNDP2	IGFBP1
SLC25A13	IL10
CYP1A2	CYCS
TNFRSF6B	CDH2
GOLM1	UGT1A1

EPHA2	ODC1
LRP6	SRXN1
GSTM3	ID1
SERPINE1	EHHADH
MIR132	NOS2
IFNAR2	FASLG
FBXW7	TFRC
MIR133A1	GJA1
PIGR	GSTA4
PRDM2	NPM1
RBM39	AHR
ARG1	XIAP
ATP8B1	SERPINE1
NDUFA13	TGFBR2
MIR26B	FN1
VPS37A	SLC3A2
SET	TNFRSF10B
URI1	BIRC3
ITGA5	GSTM2
SEMA4A	MAPK14
MIR590	ASS1
CPQ	S100A9
NOTCH4	NR1I2
CDC25A	LPL
ANGPT1	FABP4
CASC15	ABCC4
NUS1	APP
SOX2-OT	CCND2
PSMD10	CXCL1
MIR301A	PPARGC1A
BANCR	PIM1
HSP90B1	BAD
CRP	ABCA1
MIR503	SGK1
MIR33A	GPAM
MIR491	CXCL10
GAB1	ALAS1
TG	RAD51
IL4	HSP90AB1
MIR148B	SQLE
SLC37A4	SPARC
GADD45B	CXCR4
DDIT3	VLDLR
TCF7	IER3
DNMT3A	IL4
WNT7A	HIF1A
ZEB2-AS1	CYP3A11
RAD51D	GPX2
SDHA	CYP2B10
DYNLRB1	HSPE1
LIN28B	IL1A
GADD45G	CTSB
MTHFR	CCL2
LNCRNA-ATB	ANGPTL4
PIK3CD	GSTA1
NPTN-IT1	CFLAR
FRZB	ABCC1
CREB3L3	ECH1

HOXA11-AS	TSC22D1
COPS5	ABCB1A
ALK	CYP7A1
FN1	FGF2
MIR186	TPM1
ELK1	EPCAM
ESR2	TGFBI
POLD1	CPT1B
CBR3-AS1	G0S2
ERCC1	DDIT4
BATF2	CYP4A14
RBP5	CCNG2
ZNRD1ASP	GSTT1
MIR129-1	HPRT1
SREBF1	HSPD1
ACTG1	ID2
MST1R	PDGFRB
FZD2	S100A8
MAD2L1	CALR
YY1AP1	MAPK9
NAA40	ENC1
GRAMD1A	XDH
NORAD	SQSTM1
MIR130B	ALB
CYP19A1	DUSP1
PINX1	SDC2
LOX	SMAD3
TLR3	ACOT1
TCP10L	CCND3
CCKBR	CTH
NOTUM	MMP3
MIR363	CAV1
HDGF	LEP
SMYD3	ANXA5
WNT4	SRC
ITGAV	NUPR1
TP53COR1	SULT1A1
CTNNA1	INSIG1
SLCO1B3	DNMT3A
SNHG15	DECR1
MIR218-1	BDNF
CLDN10	IGFBP2
STEAP3	KRT18
NPM1	LGALS3
TUSC7	PTCH1
DEPDC5	EIF4EBP1
WNT7B	IHH
NOD2	PLIN2
CYP3A4	BAK1
B2M	C1S
RCHY1	ASNS
FLT4	GAS6
LGALS1	ANXA3
WNT11	CTSL
FTX	MAP2K1
ZDHHC2	SP1
DVL3	MT1
CSNK1A1	ACSL1

GPX3	CFB
CCL5	CYP7B1
CA9	LUM
HMGB1	FOSL1
EGFR-AS1	THBD
HLA-A	HMGCS1
RNF43	MAT2A
DVL1	OAT
MGAT5	NAT8
SLC4A2	DLC1
WNT5B	PKLR
ING1	PTGS1
ASS1	FKBP5
BAP1	HES1
BRD7	FADS2
NTHL1	MFGE8
SNHG12	GADD45G
FABP1	KDR
GSTA1	ACADL
DACT1	LDHA
TFR2	HSPH1
MIR219A1	NR1H3
ARID1B	PLK2
TXNRD1	TIMP2
PLAG1	KRT19
TIFA	PRKCB
PLPP5	CXCL2
PCAT29	IGFBP5
GSTO2	ACADM
MIR374A	CDKN1C
DGCR5	ALPL
KLRK1	NRP1
MIR485	PDK1
DVL2	EFNA1
SARNP	COL3A1
SNHG20	CYP3A23-3A1
NTS	MMP7
RIOX2	CYP4A10
MIR675	MSH2
CDR1-AS	S100A4
GNMT	NRG1
MAP2K2	FSTL1
MIR30C1	ABCG5
MIR151A	ENPP2
GLS2	RXRA
CDH17	GSTA3
LAPTM4B	SLCO1A1
KDM4C	ANXA2
MIR92A1	NDRG1
RAD51	CPT1A
MYCT1	UGT1A6
NKILA	PLSCR1
CCEPR	CCL5
ECI2	ID3
ZEB1-AS1	GOT1
RASGRP1	WEE1
CYP1B1	CHEK1
VDR	EDN1

GSTO1	RARRES1
ANGPTL8	BHLHE40
IL17A	MCM7
SMO	IRF7
MIR125B2	MAP2K6
EHD4	GCLM
WWOX	SAT1
HJV	ABCC5
HAGLR	LY6E
MIR210	BCL6
DRAIC	SESN2
HOXA-AS2	HSPA1A
MECOM	BRCA1
INSR	IL18
HINT2	XRCC5
IGF2-AS	RBP1
DYNLRB2	XRCC1
OTUD7A	MAP1LC3B
CD81	ITGB1
GSTM2	TNFRSF21
MIR216A	SERPINE2
WT1-AS	ADD3
SRA1	CKB
MT-CO1	PSAT1
MIR500A	RGS16
TP73-AS1	DNMT3B
CCL20	SELENOP
AZIN1	SLCO2A1
MAT1A	CYP1B1
PIM2	SMAD2
RAB11FIP4	GGT1
IL36A	ADM
SAMD9L	NAP1L1
TTC36	AK4
FER1L4	SULF2
RAD50	IL2
LINC00473	GSN
SLC25A47	CYBA
SAPCD2	PHGDH
PCBP2-OT1	ALAD
PTPN3	PAPSS2
CLDN7	ATP2A2
FBP1	CASP4
MGST1	RGS2
ARHGEF39	CDKN2B
DNAJA3	IGFBP4
GSTA4	DUSP6
ALDOB	GPX3
ENO2	JAK1
GLYAT	MCL1
HOXA13	UGDH
HDAC9	CD44
CASC11	BMP2
XRCC2	NEDD9
PSD3	PMP22
SLC40A1	NCL
TFDP3	KIT
NR0B2	ATF5

TFDP1	ARG1
MIRLET7A2	HSP90B1
LEP	ALDOA
PDX1	CITED2
ASPH	LPIN1
CPE	S100A6
MIR22HG	PPP2CA
SNHG6	CASP12
TMEM176A	CAR3
CERNA2	DUSP10
DICER1	SLC40A1
NNT-AS1	PROM1
F5	RUNX2
ACTL6A	FABP1
TYMS	THBS1
UGT1A7	GRB10
IL12A	MCM6
LECT2	ACOT2
PRAP1	HSPA2
FRAT1	PECAM1
GDF15	PTP4A1
CFAP52	APOE
CPOX	ABCB1B
CAT	CSF2
ACSL4	GSS
HBB	IKBKB
ATF6	BHMT
NUTM1	LY6D
CHUK	CLU
TGS1	AREG
MIR9-2	EEF1A1
C9orf78	TGFB3
EIF2D	ITGAV
CPS1-IT1	PAICS
ALDH3A1	CASP6
IRF5	RHOB
FALEC	TF
PDE11A	TNFRSF1A
NTRK3	NFIL3
GAS5-AS1	MCM4
MYB	TJP1
PRAL	MAP2K2
RASA1	AQP7
MIR136	KRT23
CD8A	TYROBP
BCL2L11	GSTM5
CLDN1	ANXA1
CREB1	RAF1
ARAF	CYP26A1
BHMT	PPP1R15A
PRKAA1	HSPA8
FAN1	ZFP36L1
MIR103A2	HSD17B4
PRECSIT	HSPB8
CD4	SLCO2B1
SNHG3	PLTP
RPS20	UBC
FABP4	XBP1

MIR124-2	LITAF
GSTT1	LHB
LINC00210	PHLDA2
C20orf204	CYP3A4
FOXO1	PRKCA
LINC01138	TAGLN
SF3B1	GULO
MIR26A2	HSDL2
CD68	F3
ADIPOQ	TXNRD1
IL2RA	LEPR
NOTCH3	IL1R1
CXCR3	PLAT
SRGAP1	HSPA4L
MIR1-2	IFITM1
MIR106A	COL6A3
DDR2	PTGES
LAMC2	CD9
ATP7A	INHBA
FGF19	PNRC1
MIR10A	ATP8B1
MIR100	CDC34
MIR532	NOX4
CHKA	RARB
MT-CYB	NT5E
ZNF350-AS1	RPS6KB1
MST1	CAPN2
NOTCH2	TXNIP
ARMC5	AGT
AOC4P	CYP2B1
DDB2	FAT1
MITF	FMO5
MIRLET7F2	GBP2
LGALS3	SDHA
C14orf132	STAT5B
EPHB2	GDF10
ODC1	HMGCS2
DGUOK	MGLL
HLA-DRB1	MMP1
ALDOA	ACO1
BCAS3	PRLR
KLK3	SLC22A5
MIR346	STAR
CHEK1	NOS3
RELA	PRMT1
CTCF	INS1
TF	ERBB2
PCNA-AS1	PGR
MIR335	SLC9A3R1
ICOSLG	CYP3A13
DBH-AS1	GCK
NR1H2	GSTO1
MAP3K20	SOX9
COX5A	GPNMB
PRC1-AS1	PON2
CEACAM3	POSTN
GIHCG	RAD51AP1
GZMB	HSP90AA1

LINC02055	SLC7A11
CARD8-AS1	LDHB
WT1	BBC3
SOX9	PDLIM1
CDH4	IL12B
LINC00974	LXN
BMI1	MSMO1
ENSG00000278769	PPARD
MIR7-3HG	SMC2
FOXE1	APOA4
HSPA6	F2R
CIB1	PLAU
EPOR	DIO2
TFRC	MT2
HLA-B	OGG1
AXL	SELE
PTPRG	PMAIP1
ENSG00000250899	S100A11
ERCC5	BMF
IRF1	EIF5
MIR518D	PPP1R3C
JPX	NR1I3
NR2F1-AS1	NTRK2
LINC01018	EIF2S1
ACTA2-AS1	IFIT1
ZNF674-AS1	CD68
LINC00665	COL4A1
PDIA3P1	ESR2
GPC3-AS1	FHL2
EGILA	ENTPD5
LDC1P	MGST3
DLEC1	MAFF
STEAP4	BIRC2
LINC00941	THRSP
GLI1	SPON2
CYLD	C3
PRL	TMSB10
HMGCR	MME
LZTS1	CLDN1
RAB4B-EGLN2	CREBBP
MT-CO2	MBP
ALPP	ACAT1
MIR30B	JUNB
LOXL2	EGLN3
SDHAF2	DSC2
SLC7A6	BDH1
MMP3	RTN4
FADD	PDGFA
IL7	TLR2
HLA-DQB1	CBS
CD36	DGAT2
SPINK1	SLC20A1
BRIP1	MTHFD1L
TMEM51-AS1	SMOX
LINC02027	TNFRSF12A
VLDLR-AS1	SCARB1
CYP2D6	CD74
THBS1	CTSC

LINC00589	SRD5A1
MIR373	MMP13
NARF-AS1	LCN2
SLC17A5	OCLN
UFC1	PER2
MIR146B	SLC16A1
MT1DP	CST3
CCR5	CYB5R3
IATPR	PIR
ENSG00000266990	PCTP
TFEB	RBBP4
SUFU	LOX
CTSD	BNIP3
IL1A	PLA2G4A
MIR100HG	S100A10
MCU	RBP4
POMC	SMOC1
IL18	SRM
DPP4	SOX4
SLC10A2	TPM3
XPC	DNAJB4
ITGA1	NFIA
SIRT1-AS	TNC
GRP	CD38
FOXA1	KLF5
NCAM1	ACAA2
HSPA1A	COL18A1
GPR35	FADS1
HLA-G	FUBP1
LINC00554	ISYNA1
SERHL	SOD3
BAIAP2-DT	ACSL5
BUB1B	ECI1
PYGL	CASP1
IGFBP2	BCL2L11
MIR499A	SLC2A4
CAMTA1	RCAN2
CD86	GRN
PDPN	ATF4
KCTD13	CROT
CDH3	ZBTB20
MIR34B	VNN1
CXCL9	HPGD
SLC25A27	ERCC1
PRPSAP1	SLC25A4
KRTAP5-AS1	FOXO1
FAM83A-AS1	AHCY
LINC00383	SERPINA7
CCND3P1	CHUK
ENSG00000258798	RHOU
CNC2	SLC10A1
MIR27B	DST
TOP2A	B2M
CDR1	HERPUD1
ACTC1	DBI
CALR	AIFM1
MIR494	ALDH2
ARC	SFRP2

SOD1	NUCB2
XPA	PSMB9
PPARA	HSD3B1
IL2RB	CTSS
FOXO3	ALCAM
TNFSF15	DDAH1
BCL10	KNG1
MUC16	FGF7
POU2AF1	PON1
CECR7	BECN1
TNPO3	POLA1
HAVCR2	TXNL1
DAPK1	ZEB2
RNF6	DNAJB1
NCOA4	SULT2A1
AMACR	KLF6
LINC00173	PALLD
NPC1	TUBB2A
SIRT3	FABP3
CEL	TNFRSF11B
SLC10A1	ALDH1A7
DMD	PIK3R1
VEGFD	ACACB
PFKFB1	MBD1
GLT1D1	ATP6V1A
URB2	ARNTL
DIP2C-AS1	NFKB2
FABP5P3	RAMP1
MAPKAPK5-AS1	SNAI1
LINC00926	ADGRE1
OVCH1-AS1	BIK
LINC00601	TUBA1C
HTR2A-AS1	KEAP1
SEMA6A-AS1	UPP1
CA3-AS1	CSRP3
LINC01419	HACL1
PERCC1	KCTD12
ULK4P2	TMSB4X
ENSG00000228363	CTSH
ENSG00000258711	RSAD2
ENSG00000272953	TAP1
ENSG00000265778	GPX4
ENSG00000225544	ATP5F1B
ENSG00000145063	BCL3
ENSG00000202542	HDAC1
CXCL1P1	HNF4A
ENSG00000279159	NCF1
LOC105369388	INHBB
THY1-AS1	TAT
ERBB4	SDC4
SERPINB5	SPHK1
MUC5AC	PLK4
CSF3	RRM2B
EIF2AK2	HAL
PLK1	SLC25A30
FANCC	MCM5
DDX3X	CKS2
MSLN	GSTA5

LCN2	PLCB1
SLCO1B1	NID1
CD40	SPON1
CLU	CDO1
TNFSF11	IKBKG
ST14	SAA1
BRD4	SERPINA6
MPO	ABCC6
SPIB	FBLN1
MMP12	FMO1
MIR98	EIF5A
CP	TGM2
PHB	G6PD
PCLAF	CPEB2
TFF1	DAPK1
ACOX2	CYP2C29
OTC	MAP2K3
TOP1	PLK3
ACCS	RNASE4
RUNX2	EDNRB
MMEL1	MGST1
CEBPA	LGALS1
NAT2	FDPS
MMP13	MAP3K6
EXO1	TCF7
S100A1	CLDN4
CXCL10	FBXO32
MUC2	CES2
APOA1	CYB5A
MMP11	GLRX
CD151	ORM1
LINC01307	HSD11B1
MLANA	SLC6A9
MIR331	ADIPOQ
MIR187	CREB1
SHH	PEMT
EIF4E	BACE1
CALB2	NCF2
ELN	SMAD7
LMNA	ADH1
CSF2	LGALS3BP
CDKN2C	NFIX
TLR9	THRA
TCN1	CKS1B
KCNQ1	KYNU
NONO	NRIP1
MYCN	SLC27A2
MIR378A	SLC6A6
BUB1	FTH1
XBP1	BTG1
DLK1	MGP
EIF2AK3	TGFBR3
APOB	CYP2C39
ABCC4	INSIG2
CTNND1	PALMD
TRIM24	TUBB6
S100A4	AQP3
JUP	RFC3

PIP	ABCB11
SALL4	PFKP
LEPR	TNFSF11
CDH13	ITGA1
PML	SCD2
TNFRSF1A	TIMP3
ATR	VTN
KLF4	CYP26B1
GADD45A	PTPRG
CTSB	SLPI
CD40LG	RPSA
MTDH	SERPINF1
SFPQ	PLD1
CD24	APOC1
MIR320A	DDC
RINT1	MAP3K5
LAMB3	WNT4
ITGA6	FCER1G
MIR299	MASP1
LNCARSR	PSRC1
HSPD1	SPTBN1
PSMB8	TRIB1
PPM1D	PRTN3
KIF1B	CHKA
PDCD4	SLC27A1
ALAD	ERBB3
FBN1	TMPO
BGLAP	TOP1
RACK1	FAM107B
HOXB13	CYP11A1
PRKN	TRAF2
MIR372	WSB1
CXCR5	ABCG1
BPIFA1	MAP2K4
ERCC3	PEX11A
SART1	ALDH4A1
SLPI	DNAJB9
FSCN1	PDGFRA
HP	COL6A1
IREB2	PRKCZ
GATA6	SERPINB2
MAD1L1	CYBB
ROCK1	PIGR
RAD18	SH3BGRL3
AMFR	COL6A2
AHR	GLB1
MIR95	HNRNPA1
MYO5B	NUDT7
CFTR	PPL
PRKAR2B	TGFB2
MIRLET7I	APOB
EIF4EBP1	STIP1
IL6ST	CFI
ELAVL1	FOXQ1
CYP7A1	INS
RARA	THYN1
HES1	CDK6
PPP2R1A	FLT1

MIR342	C6
MXI1	DLK1
PTER	COL4A2
MIR154	CYP27A1
CHRNA3	GAMT
GHRH	ABHD2
MIR361	CLDN7
CCND3	METTL7A
CA2	PSMA1
HLA-DQA1	PRKCD
GCG	TUBB5
MIR193B	S1PR1
GCNA	CYP19A1
DMBT1	CADM1
APOE	IFI27
ETV6	SORT1
RSPO1	CYP2B2
DLL4	ROCK2
DDR1	SLC19A1
PECAM1	DSP
POSTN	SNCA
HLA-C	MARCKS
EPHA3	NFKBIZ
F3	JAG1
NRP1	BMP7
SLC7A5	CSRP1
SEPSECS	CYP2B9
CSF1R	IFRD1
HNRNPK	KPNB1
PPP2R1B	UNG
PTPN1	CHEK2
FST	SCARB2
HPX	CREM
TAP1	HADH
NRG1	ITPR3
IGF2BP3	PROCR
WNT10A	TFAP2A
BMP4	FGF1
PDE8B	KLF13
TEK	PHYH
GJB2	WFDC2
ATG5	UCP2
MICA	GUSB
TACC2	SLC22A7
ATP11A	LDLR
ELOC	MYBL1
KITLG	SLC7A5
MIR206	PDCD4
POLD3	IL1RL1
TKT	CRYAB
HBEGF	DHCR24
MIA2	ELOVL6
IL3	CPE
GH1	KLF10
SERPINC1	BAAT
ILK	CLMN
ING3	SLC10A2
TPO	GNE

ELANE	TP53INP1
ASCC1	PRKAA1
CYP2A6	IFIT2
APPL1	SLC39A8
INHBA	ISOC1
CCNL1	BAG3
TDP2	BTG3
TNFRSF11B	ALAS2
AGR2	MYH10
GATA3	CAR2
CDC42	FNDC3B
MT-ND4L	QPCT
PHKA2	ETS2
JAK3	KLHL24
NDRG1	TUBA4A
TDP1	NR1D2
LOC110806263	POU5F1
ALDH1A1	RORA
CLDN4	SERPINH1
USP53	ECE1
MAX	ENPP1
MICB	MAPKAPK2
OSMR	GALM
PRSS1	ST3GAL1
GJA1	FOXA1
MIR326	ACSS2
DOCK8	LRP1
ANXA1	RAN
ACTA2	TFF1
DKC1	GPAT3
AGER	GPC1
LDHA	PSMB8
GHR	MTHFD2
CCND2	PLPP3
TRAIP	CDC25A
CEACAM6	ARPC1B
SELENBP1	CCN4
POLK	DFFB
TFAP2A	HIPK2
C7orf50	LHX6
NAMPT	NGF
CSF1	AOX1
TEX264	ADHFE1
C11orf95	PSIP1
TSHR	PDIA4
REG1A	IFIT3
PTGS1	PHLDA3
ZNRF3	BGN
GALT	E2F3
U2AF1	GSTK1
AFAP1	PNPLA2
NAPSA	AXL
NEUROD1	HMGN2
KRT17	PPAT
APEX1	RPA3
ERN1	TNFRSF10A
ATF4	COL5A2
S100B	CYP2A4

MUC6	ETFB
LPCAT1	MOGAT1
CXCR2	ZMYND8
NF2	BGLAP
BAG1	TSC22D3
VDAC1	PLAUR
CNOT9	ADAM17
FOSL1	COTL1
IQGAP1	PTPRD
BID	TEK
IL1RN	ALDH1B1
UTP6	ALDH3A2
NCOA3	FGF21
HPRT1	OSGIN1
UGT1A1	DHRS3
CS	EZR
NR1I2	MIF
ASL	ATP1B1
MIR328	GSTM4
PEBP1	LTF
FECH	SMAD1
ETV4	PLA2G2A
AIFM1	IFI44
MIR376A1	SLC13A3
NTRK2	SELENBP1
IL11	MYD88
KISS1	GCH1
CUL2	CLIC4
IDO1	MYO1B
GLI2	SERTAD1
H19-ICR	CRIP2
IKBKG	DKK3
MSMB	CSE1L
HLA-DPB1	SLC19A2
CPS1	CDC25B
LRP5	NFKBIB
CCR7	SIRT1
MAP3K5	NR2F2
SREBF2	RGS4
BCS1L	ROCK1
COL4A2	ACAT2
PLG	CXCL9
KRT13	HNRNPAB
APOBEC3G	SYNPO
MIR584	NREP
MIR497	LGR5
ID1	BCAR1
ABCC8	PA2G4
MAP2K4	PRSS8
STAT4	PSMC4
MAPK10	BLVRB
KCNJ11	GPD1
TTN	NES
ERCC4	COMMD3
HPD	GATA3
RPS3A	BMP4
GSN	FUS
PRKDC	G6PC

CD59	TRADD
MCM2	FOXO3
PIN1	CYP8B1
MOK	KLF4
PAK1	CAPRIN1
MIR377	GLS2
S100A7	HBA1
YBX1	CD24A
MC2R	ISG20
CCN1	SERPING1
BIRC7	SESN1
DNAH8	LMNA
LAMB1	DPP4
CADM1	EPHX2
MIR502	CFH
PGF	CACYBP
FGF1	NT5DC2
GRN	PNPLA3
TNFRSF11A	PSMB3
ALAS2	FYN
TSG101	NOTCH1
VEGFB	FSHB
KRT10	DCLK1
MBL2	RXRG
ADAM10	DHFR
IBSP	PRNP
HMMR	CRIM1
DES	FGL1
NNMT	SPARCL1
MMP10	NCOA1
TAT	TCF4
IVL	TUBB4B
TH	UGT2B1
MAP3K1	ALOX5
SOX4	HMGB1
PSG2	MARVELD3
VTN	MYLK
SIL1	NR1D1
EPHB4	PSMD12
TDGF1	SOAT1
EWSR1	SULT1C2
SERPINB4	ICA1
WNT10B	LTBP4
ABCG5	TLE1
PSMB9	TKT
CYP2C9	MATN2
HCCAT5	QSOX1
FGF7	SCPEP1
MIR370	HELLS
FOXA2	PCSK9
SFN	CES2C
PTH	ANXA6
LINC01554	TNS1
RAD23B	TMEM97
XRCC6	CCT3
MMP8	CREG1
SLC51A	FRZB
ROS1	INHBE

TUBB	VAT1
PTTG1	ATPIF1
INHA	TLE2
PPARGC1A	EIF2AK3
DHFR	CDC73
LDLR	DES
LTA	GPD2
APAF1	NOLC1
TCIM	FHL1
IFITM1	OGDH
TLR6	ARPP19
PNMT	ATG12
HHCM	ERN1
UHRF1	GATM
MIF	NR4A1
IRS2	ETS1
CEACAM7	HSPA4
CASC9	LEF1
RARS1	VDAC1
SGO1	AP1S2
RRAS2	SACS
SPINT2	LGMN
LAMA3	SLC4A4
NR3C1	FGFR2
CCNG1	MX1
NCOR1	OTC
MACC1	PAK1
CLDN3	FADD
SERPINA7	ATF2
PTGER4	SLC38A2
SERPINA6	CKM
TNFRSF10D	SDF2L1
FAP	JAK2
EDN1	ACKR3
ADH1B	C1QB
PTPN13	DNAJC3
PTPRN	HNRNPD
SRD5A2	MAF
ABL1	P4HA1
ZFHX3	SPAG5
ELOB	FGG
ANPEP	G6PC1
H2AX	KLF9
XRCC5	NTN1
EBAG9	SERINC3
S100A6	EIF4E
RBP1	EIF4A1
CD5	MX2
BIRC2	PSMC5
SDC1	SEPHS2
RBX1	AQP1
ROBO1	HSF1
ALAS1	ITGAM
MAPK9	HSPA1B
SCTR	DLAT
MIR3658	ETHE1
FZD1	GCNT2
MIR128-1	DHRS4

THY1	LASP1
TFF3	ORC6
USP8	SDS
EGLN3	XAF1
MIR7-1	CDC42
TLR5	RHOC
SLC11A2	ZBTB16
ERG	TSPAN3
HDAC2	MDH1
VTCN1	P4HB
MATK	RELB
MIR135A1	FKBP1A
MIR9-3	GBE1
ADH1C	NOL3
YY1	TUBB2B
KDM1A	CCNE2
BNIP3	TTR
LINC01139	JUND
STC2	HSD17B2
WIF1	IDE
EGLN1	ARHGDIB
ECT2	LBP
GRPR	SHC1
KISS1R	SMPD1
FZD4	DIABLO
RPSA	SULT1E1
SCARB1	GPM6B
MPL	ILK
PLIN2	CSF1
ROCK2	ACADVL
SERPINB2	CASP2
ITGA3	GFRA1
MAP3K8	TJP2
NAT1	HADHA
PKP3	COL5A1
FATE1	CSF1R
CLDN6	SELL
G6PD	TES
MIR28	BZW1
CKS1B	HSD3B5
NAFLD1	SRGN
NAFLD2	TPP1
GFER	RBPMS
RB1CC1	TGFBR1
MIR196B	TSPO
MLXIPL	AKAP12
ARNT	PER1
CLIC1	DCXR
NGFR	LIG1
AVP	RND1
CDC25C	DDX21
DMPK	EPS8
MYO18B	GAS1
GSTZ1	PSMB10
PRC1	SHCBP1
SHBG	ANP32E
PRTN3	DDIT4L
PTPN12	HPCAL1

AIMP2	LPIN2
WT2	MMD
PDGFB	MMP14
NSD1	NANOG
CCS	OLFM1
FCHSD1	PABPC1
SELENOI	RPL10A
SNORD42B	TCF7L2
MIR4698	BCO1
ALPG	NDUFS3
NES	SLCO1A4
HUWE1	TFPI
IL24	GTPBP4
CYP11B1	MBD2
PPOX	PCCA
SNHG16	NDRG2
TNC	RPS3A
APOC3	SLC2A3
OPCML	PRKCE
HSF1	ITGA6
FGF21	MDK
DDX58	PRDX5
CSNK2A1	CHORDC1
PCSK9	POLE2
HMGA1	WARS1
DUSP1	FMO3
PPIA	VDR
IGFBP1	COL27A1
ADAM12	HMGN3
FABP12	MYO1D
MIR330	TMEM237
CXCL1	SDHC
IL15	TPI1
GSR	NCOR1
TXN	CXXC5
ALOX5	DACT2
CLPTM1L	LGALS8
CCN4	MYBBP1A
TIAM1	CAV2
ACVR2A	PPARGC1B
GNAS-AS1	SMPD3
FDPS	DDB2
SASH1	CIDEC
PLAGL1	IMPACT
IRF3	FSCN1
LPL	GZMA
NCOA1	LRP5
LOC111832670	STOM
ASGR2	IL1R2
FGF4	DUSP5
PDCD1LG2	LAMB3
HERC2	SLC2A5
TLR1	PLIN4
RIPK1	HNMT
DCUN1D1	MVP
TP53BP2	RAB31
RXRB	ACOT4
NQO2	CYP2A1

PDPK1	INSR
F2R	HMGN1
BCYRN1	MAN2B1
TM6SF2	PLIN5
MIR501	ACP5
MCC	MKNK2
CIP2A	PSMA4
TPD52	RAB30
STAT5A	CD40
UCP2	RHOA
CD69	DIO1
TNFAIP3	PEBP1
MIR542	HK2
GAS8-AS1	CNN1
SAT1	OLFML3
DAXX	ARRDC2
LASP1	CPQ
MAD2L2	ELOVL3
NR1H3	NFE2L1
SELE	ENPP3
SSTR5	PLAA
ANXA10	SATB1
NEDD9	TIPIN
SURF1	TMBIM1
LYVE1	CSAD
STK4	ENPEP
MIR124-3	LRG1
CDC6	AKR7A3
CFH	ALOX5AP
UMPS	PTPRF
MIR135B	BMP2K
S100A2	TMED3
CDC5L	TRP53INP1
MDC1	PLG
ENSG00000266919	ARHGDIA
BRMS1	FASL
MYH8	KAT2B
KLF5	IDI1
ADIPOR1	DRAM1
GPX1	PSMC3
BMP7	SLC1A5
SLC39A1	MSH6
MIR449A	ST6GAL1
RHOH	STEAP4
F10	IL6RA
SERPINB1	MEG3
STC1	SERPINF2
ASGR1	ITGB2
STAT5B	NCOA2
TGIF1	PPP1R3B
KDM5C	ALDOC
LGALS3BP	TNFRSF1B
NOS3	BRCA2
SIX1	CKAP2
MIR199A2	CCL8
FLT3	FZD8
HHEX	IFI6
B3GAT1	FBXO30

PRKCI	KCNJ8
ANG	PDLIM7
TINCR	RRBP1
TCF7L1	DNAJA1
COL18A1	EFNB2
CTCFL	PSEN2
NEK3	PSMA7
GAL3ST1	GFAP
SLC5A5	PTK2B
TNK2	MMP12
GNRH1	MVD
LAMTOR5	HDC
HPN	PAWR
CTAG2	AKAP8
LBR	DYNLL1
SSTR1	SGMS1
MIR885	APLP2
LATS1	DMBT1
PON1	SYNJ2
KRT15	TAX1BP3
JAG2	CRAT
EFNA1	AGTR1A
ACVR1B	CYP2C11
GRB7	ARL4A
HCP5	PITPNC1
MIR31HG	REEP5
UROS	RHBDF1
AHSG	ASPH
KDM6A	HIGD1A
CSK	PSMB5
PDGFA	RAPGEF4
NAGS	RIDA
MIR574	SLC25A10
CDC25B	CPT2
DLL1	PPP3CA
GPER1	MAOA
ATP8B2	ANG
ATP8A1	HADHB
ANXA6	CES3
F2RL1	EIF3A
FER	LGALS4
TGFBR3	MTHFD1
COMT	MYO5A
SPHK1	NDRG4
MIR202	PCX
PFKFB3	IRF1
MTAP	ADAMTS2
REEP5	COL12A1
MEST	NFIB
AIP	TXN1
KL	CFL1
PPY	MAP2
TYK2	ACHE
TACSTD2	HBEGF
PLCE1	ANPEP
SLC31A1	NFATC1
RBPJ	CPS1
CD79A	DDX5

ITGA2	FBXO5
HEPH	FOXA2
TNXA	GLO1
MIR572	LAMC1
SLC15A2	LRRFIP1
MYOF	MAL
CCN3	PGRMC1
DBH	SMO
LIG4	YWHAB
ST13	AKR1B1
FOXD2-AS1	ELK1
GPR101	FZD1
IL4R	IMPDH2
ITGB3	KIF22
MSH5	SAA3
PRKD1	TIMELESS
CASP5	HBB
MCAM	PTPN1
MAGED2	SLC11A2
MAN1B1	BCL6B
YWHAE	TCEAL8
PLA2G2A	FLNB
MIR744	CLTB
KLK10	RAD18
TOR1A	RDH11
HGD	ELF3
SATB1	NOP56
MIR32	NRAS
THBD	DGAT1
MIR16-2	HNRNPA2B1
CLDN8	TYMP
BTC	ACOT3
FURIN	EREG
AKR1C1	RUNX1
PNOC	CD86
CD28	GAS7
TINF2	HDAC6
MFN2	PRKCH
TFPI2	UGT1A9
EREG	PCK2
AIRE	CX3CL1
CD247	HSPA1L
SGCE	HPX
IGFBP5	NQO2
PRRT2	TLR3
H4-16	TNFAIP3
MIR29B2	YWHAZ
ALOX12	CLDN2
ACKR3	LSP1
VIP	PTMA
SLC2A4	AHNAK
RPS6KB2	FAM107A
TMEM30A	HEXB
PRLR	HPD
THRB	MPP1
ETS2	UBE2L6
PTPA	POLG2
CUL1	PDE4B

CASR	FILIP1L
MRPL58	P4HA2
DLAT	AIF1
CASP2	CCL6
CD276	SDC1
CYP8B1	VASP
SULT1A1	ANKRD1
KRT1	PRIM1
EPHA1	SLC7A2
IL5	TNFRSF19
SHC3	USP18
MIR365A	MMP10
MIR509-1	VWF
CD9	CD37
NGF	XRN2
MIR103A1	PAM
ACO1	SMARCA2
GFRA1	CHAC1
FGF5	EEF2
PRF1	GLS
TNFRSF10C	TFAM
CCL26	CYP3A2
ITGAL	ALDH9A1
S100A8	ARHGAP24
CXCL14	CD52
GLOD4	PAQR9
CDK14	SCARA5
IFNAR1	UBA7
ATP8A2	ANTXR1
HSD17B1	FEN1
ACTG2	GK
IL7R	KLF2
CD63	MAP3K14
MDM4	TBL1XR1
RABL3	TRAP1
ADIPOR2	HMGA2
APP	IGFBP7
HOXB9	PROS1
TMPRSS6	TPM4
RETN	WNT5A
PRKX	COX8A
CGB3	SEC63
BDNF	SLC48A1
ATAD2	GPT2
SMAD6	ADA
BCAR1	LAMC2
AGO2	ANGPT1
WNT2B	CAPG
PNLIP	FABP7
ATP11C	HAT1
TIMELESS	RBL2
ZNF217	TCEA3
ADAM9	IL5
ONECUT1	CARHSP1
ATF3	CD84
DROSHA	CYP2C37
ACE	DDX39A
CCNE2	LIPC

TRAF6	CTSE
ABHD16A	GRINA
SLC16A4	CCL7
TGM2	ATAD2
CCAR2	PIK3R3
DRD2	MYH6
ALCAM	ATP6V0E2
PCSK1	HIP1R
CCNH	CD83
FOXC1	TGM1
ABCG8	NAMPT
ACY1	BDH2
MCM7	COMTD1
ARID4B	GPCPD1
TLR7	LAPTM5
HBB-LCR	NFYB
CTSC	RAD23B
CASP1	RFC5
IGF2BP1	SET
NET1	TOMM20
MT-ND6	HES6
GLI3	TNFAIP8
RBP4	ABCG8
PSMA7	MAP3K8
EEF1A1	VCL
IL21	ABCD2
TTK	GJA4
CTSL	RPL32
TAP2	UOX
CTSG	ELOVL5
MAP2K7	TCP1
CD58	LY6A
CCK	SERPINA12
PTPRJ	ATL2
HIF1AN	MUG1
WEE1	RCBTB2
CUL3	TMEM47
SEMA3B	IVNS1ABP
FZD8	ADGRG1
SAA1	AMIGO2
MSN	C8B
PSMG2	CAVIN3
CTHRC1	CKAP4
CDK7	CMBL
VWF	CYP2A5
SLC51B	FABP2
PSC	MMP8
PBC2	MYOF
PBC3	PTPN3
PBC4	RRAS
PBC5	TLN1
DSP	EPAS1
HNRNPA2B1	ALDH7A1
S100A9	ZMAT3
NR1I3	CELA1
FTH1	GPC4
FANCD2	GPLD1
PAX5	IRF5

MTTP	KPNA2
USP7	MAP3K1
UBE3A	AEN
ECM1	ANP32A
SLC39A6	MANF
RAP1A	IFITM3
SYK	SOX17
AMH	TRAF6
LIFR	RPL3
RRM1	YBX3
SOS2	ECHDC2
LGR5	POLD4
HSPA8	TMEM176B
SMARCE1	PRKCI
FABP6	TNNI2
EPHA7	SMC4
EPB41L4A-DT	BBOX1
FERMT1	LIMA1
KAT2B	TFDP1
MIR218-2	CFD
TSPAN8	C1QA
RALA	IL4R
ST6GAL1	MRC1
TK1	TPR
FYN	SLC1A2
FGF8	FMNL2
LDHB	LACTB2
AGL	MCM3
LRPPRC	PCCB
HHIP	PTGFRN
NPRL2	RASGRP2
STIM1	S100A16
ARTN	SKI
ESRRA	TM6SF1
ADAR	CSRP2
MIR425	MAP4K4
SULF2	F7
GALE	NR5A2
CEMIP	CCNC
MIR184	PPIF
IGFBP7	SPSB1
ADA	ST3GAL5
CLDND1	MAFB
ITGA9	PDIA6
PRDX2	ADH7
WNT16	GM2A
ZBTB7A	MBL2
INTS6	RPL23
PRDX3	S100G
LGALS9	H19
FKSG49	PLS3
DCK	DBP
EDNRB	BSG
MAPK7	PRDX4
LTF	RPS19
TXNRD2	REPS1
MRPS27	LAP3
POLG	BLNK

SNCG	IL17A
RIPK3	PAK2
HBA1	SIPA1L2
PRAME	TACC1
DKK3	TLR5
AKR1C3	ITIH4
TTR	LIF
MAP3K14	ORM2
BMX	PCSK6
SERPINA2	PTMS
TTC33	RABGAP1L
CCR4	APOC3
DLL3	ID4
GLS	MUC1
CDCP1	UGCG
RAD54B	ZFAND2A
CDK5	PLCG1
ZMYND10	DDX58
TFAM	RETSAT
WNT6	MRPL49
HBA2	PPIL3
PLAT	AHSG
NCL	DEPTOR
SLC9A3R1	PDXK
HSPA1B	PSAP
MIR615	ARRDC3
MVK	MYB
PDGFC	ARHGAP5
LRRFIP2	KHK
TP53BP1	PGM3
MIR452	PRPS1
SPINT1	SFPQ
ROR2	SH3BGRL
ROR1	SPTLC2
CEBPB	SSR3
VAV2	ZEB1
HAVCR1	DAD1
HDAC6	FOXA3
SRD5A1	MFAP2
NCOR2	PLPP2
MIR302A	PPM1K
SMG1	RCN2
RPL6	SALL1
HLA-E	SYT12
FAF1	NOCT
MTSS1	GATA4
MIR4435-2HG	CLOCK
LINC01194	DCT
KDM5B	FAM43A
FUT2	SERPIND1
ABCC11	ATG5
CCNA1	ORM3
GNRHR	CDCA7
KHK	GDA
ZFP36	LBH
TGM1	OPTN
FTO	GSTT2
CDH6	SORD

LOXL4	SOX2
BLACAT1	ADRB3
RBL1	LAMA5
CRYAA	SLC6A8
ATP11B	CADPS2
FXYD2	CD53
FTL	EMP2
AREL1	PSMC3IP
PYY	AMACR
AOPEP	IRF6
TMEM8B	NID2
SIAH1	BTF3
OIT3	CYP2D22
LAMC1	EPB41L2
PRDX1	IPO4
CDH5	LSS
PARK7	OPLAH
PROX1	PXMP2
GBA	TM4SF4
CYP3A5	ITPR1
TRIM33	GINS2
SULT2A1	RPL6
SLC25A37	SLCO1B2
ACHE	DIO3
ADAM17	RAD50
ZFP57	ADIPOR2
CRYAB	NIBAN1
ALPL	HCFC1
TAGLN	LYSMD2
ACP1	MPP7
FLG	SMPDL3B
NKX2-8	RASD1
CYP21A2	APOC2
DSE	CHPT1
HPGD	EEF1G
ARMC10	IRF3
WNT8B	PCOLCE
DSG3	TLR1
CYBRD1	TNNC1
FOLR1	AXIN2
CXADR	DECR2
SLC29A1	EHD4
UBE2D1	ETFDH
TPM3	NECTIN3
VCAM1	PFKFB1
TMPRSS4	RBMS1
SNCA	ENO2
DEK	PSMB1
SUGP2	RBL1
RBL2	C8A
LAMA5	HYAL1
PRKAA2	KITL
PKP1	SPRR1A
CRABP1	TPD52L1
PYGM	ISLR
DNAJC6	IL33
TBPL2	PTX3
ZNF606	F2RL1

HEY1	PER3
RHOB	APOM
PON3	NET1
SCARA5	TENT5C
PDIA3	TFF3
MCM3	ATP5F1A
RAB25	ARF1
MIR345	MIR34A
PTK6	SLK
RAD52	AKT2
GPR87	LIPE
SERPINI1	TNFAIP2
MIR362	ABHD6
FXN	ACSL3
MBD4	ECI2
FUT4	HSPA12A
E2F4	POLD3
CD27	GSTT3
PTCSC1	HSD17B6
ITPR2	LYZ
MVD	RBM3
IL32	SLC37A4
WWTR1	CELF2
LOC100505549	ETV4
CD46	IREB2
L1CAM	PRKACA
FZD10	SERPINB5
CDX1	HNRNPH1
FTCD	JUP
ANXA3	PC
HDAC4	PROC
ADM	PRKAG1
CASC8	CPD
ACTN4	MACF1
DDX5	MSRA
FEZF1-AS1	DAB2
SPOP	HTRA1
PC	SLC31A1
AQP1	MAP3K4
GATA4	TUSC3
GATA2	CCR2
PTP4A1	CAPN1
ARSH	DTX4
FZD3	RDH10
PNKD	VIL1
FBXO11	FBN2
PPP2CA	AVPR1A
SESN2	DUT
SRPK1	KRT5
DGUOK-AS1	IMPA2
HIC1	TPD52
DAB2	DUSP4
LGALS7	PLA2G7
PRKACB	PTGDS
SFRP2	SLC16A3
PRKCE	ATP6AP2
SPAG9	CSTA
CYP24A1	SESN3

SLC25A15	SNX10
CAP1	TPMT
TJP1	SLC30A1
ATF1	CYP2C9
CDIPT	ABCD3
LIF	E2F4
F7	ESRRA
HEY2	FAM171A1
ELF3	SMAD4
TNFRSF9	TGIF1
MIR92B	ACADS
GFAP	GRB2
PPARD	MLH1
ATOX1	PSMA2
MIR432	DNM1L
PDGFD	TSHB
AGR3	AKR1A1
DUSP6	BCHE
RARG	AASS
NISCH	ABI2
FLVCR1	HCK
GALNT12	PKP2
CCL4	PTN
UBE2C	RPS3
RCC2	ADGRE5
FZD5	CDV3
CHI3L1	EVI2A
PKLR	RNF125
HSPB2	TENM3
VCL	AIFM2
RYBP	CCL11
MIR198	F8
TRIM25	KLK3
WNT9A	ACTN1
YWHAZ	ENO3
TSPY1	PLOD2
MIR17HG	SLC25A20
LINC00963	CSTB
MLKL	OGA
ZNF148	RRAS2
API5	EP300
NR3C2	FGFR1
MMP26	CORO1A
S100P	DUSP8
IFITM3	FBLN2
RAN	OXCT1
ANXA4	PPFIBP1
NKX3-1	TXN
SLC9A5	EBNA1BP2
OR10S1	NKTR
ZNF99	PBX3
HLA-DRA	SLBP
FGFBP1	THNSL2
SEMA3A	VAV2
SOX8	RIN2
HLA-DPA1	HRG
NEDD4	MAGED1
SNHG7	MCCC1

SRRT	CALD1
PHLDA2	NFAT5
PPP1R13L	EMP1
MARCHF6	DHRS9
DLD	GNL3
TPI1	NCAPG2
SCD	RAD51C
FBLN1	BASP1
BCL3	DCK
PIWIL1	MERTK
IL13RA2	PLA2G12A
ATRX	PLEC
ALPI	SEMA3C
PRNP	FBN1
LINC01133	IL13RA1
PI4KA	IL7R
RICTOR	NDUFS1
DAB2IP	CYP4F14
RPA1	NCAM1
ACER3	OSMR
CD1D	RGS5
CUL4A	CES2A
SSTR4	MCCC2
SOX10	MPDZ
SBF2-AS1	RPS20
NCOA2	SLC12A4
DCLK1	PRL
WTAP	CLYBL
GGT2	CYB561
GGTLC3	RNF4
GMNN	TCF21
PAX6	AKAP1
ACOX1	ATP1B3
PIM1	CXCL16
PEA15	ILF3
PCCA	NME2
RRM2B	BUB3
RING1	GRPEL2
MAGT1	HACD2
MIR340	SOX13
CCR9	HNF1A
SLC39A4	PDLIM5
FCGBP	SULF1
ABCB7	TEF
REG3A	XPC
TRIM28	CCL4
MIR217	PGF
MAP1LC3A	UNC5B
AQP3	RGN
TBCD	SLC29A1
RASSF5	CXCL5
THBS4	SNAI2
CDC20	ABHD5
NEUROG1	BMI1
SCT	CABLES1
VCP	DIAPH3
MIR663A	DKC1
PLEC	FAM13A

ACADVL	GRB14
CRABP2	KDELR3
HDAC5	LGR4
ULK1	PAPSS1
PSEN2	RPL17
PRKCG	WLS
IAPP	CIDEA
PSMD4	CISH
CSMD1	RGCC
APOD	SPINK1
RAD51L3-RFFL	CCT2
KIFC1	DAPK2
SLC39A14	IFI30
GYS2	MTAP
TRAF4	PPM1D
USF2	TMEM98
MYH11	ARNT
IL33	ATP2B1
HOXA1	SRSF7
HAND2-AS1	NR4A3
HSP90AB1	LRRC28
GCKR	USPL1
MIR519D	CBR3
HIPK2	COX4I1
LAMA4	LRRC39
PLA2G4A	SLC22A8
ATF2	PERP
TRIP13	SEC61A1
MIR625	SMAD6
MIR371A	TRAF3
EPHB6	CDA
SYNE1	ELOVL2
GPRC5A	FZD2
ABI1	PPRC1
MT-CO3	COX6A2
GPBAR1	SH3PXD2A
LATS2	XRCC3
SLC3A2	FGB
TNFRSF25	ABAT
INS-IGF2	ITGA5
YES1	PRSS23
CYP11A1	VCAN
ESM1	YWHAE
FOXP2	COX6A1
PHF20L1	MASTL
MIR129-2	NFE2
DEPDC1	PLEK2
ENAH	RNF13
IMMT	TLCD4
JUND	UBA52
GHRL	CASP10
PVR	FDXR
DDB1	NGEF
MIR608	RRS1
NR5A2	SYNE1
MIR92A2	AGTPBP1
GLB1	ARIH2
MIR511	SUSD2

TRIM37	USP10
F2RL3	CLDN3
GPNMB	DLD
BBC3	RASSF1
PER1	ADAM10
SLC5A8	CCT8
RGS22	IL10RB
ANXA7	KMO
GBE1	MRPS18B
CRH	NUDT1
VCAN	SLC25A13
KRT37	STAU2
FGB	STK39
DST	LYN
ABCD4	VEGFC
GGT3P	ABCA3
NUAK2	NRF1
ADGRE5	PSMD4
FEN1	BHLHE41
IFNL4	NPC2
MACROD1	STAT2
ECHS1	BAMBI
KRT16	CD47
BRD2	PF4
HELLS	DHCR7
SULT1E1	ABCC8
SIRT6	ITGAX
HOXA10	PLAAT3
INPPL1	SUCLG2
ZDBF2	TPM2
COL4A3	VEGFB
MIR498	ARL4C
ACKR2	PPM1A
MIR637	SUCLG1
APOH	BCL2A1
PTK7	GOLM1
S100A14	TIAL1
CSE1L	UBA5
PSMA1	APOD
CD99	PNP
LRP1	RAC2
FUCA1	CTNNA1
PRMT5	LOXL2
EFNB2	FAAH
MT-ND1	ADAMTS4
AGK	CTSA
ST3GAL6	NUDT4
MIR708	SLC46A3
KLK6	CYP4B1
PLPBP	EBP
STK33	SORL1
PENK	VCP
TRADD	DLG1
MIR411	MTUS1
TARS1	TNFRSF25
BRINP1	DVL1
MED19	POLD2
AGTR1	IL15RA

MIR383	CORO6
MIR1202	ERCC4
TNS4	HSD17B11
CD109	UCP1
MIR506	WWOX
NAP1L5	ADGRL2
DYNC1I2	ELF1
GPX2	MPEG1
BIN1	MYL2
DSCR8	NDUFB6
LCAT	POLR1B
TBX3	RAB3D
ERCC8	MGST2
KCNK12	CIRBP
PRSS58	FCGR2B
EEF1A2	LTB
CSNK2B	TSC2
EIF3A	FCGR3
ARFGAP1	PSMD7
COA8	TTC3
COL1A1	CTTN
MCRS1	IER5
TRAF2	PLAGL1
MYD88	PLXNA2
PTN	TSKU
SLIT2	ZFP36L2
GNA11	FOSL2
MELK	ANKRD12
COX10	HOPX
KPNA2	IRF8
SATB2	RPS27
PTGER2	SLC23A2
FZD6	IGFBP6
TRAP1	FOXP1
JUNB	MALAT1
CTBP1	ACAA1A
CASP6	FPGS
RAB23	PRMT3
BTD	FITM2
FAM3C	ASGR1
LAG3	NUP153
NNAT	PRKD3
SIRT7	PRPF4B
LYPD3	RPF2
TIMP4	RUVBL1
SLC25A11	ESPL1
EPHA4	CCPG1
NFKB2	COQ8A
KLF11	DGKA
HSD11B2	GSDME
SLC25A22	H4C3
SCG5	MPST
AGMAT	MRPS6
LGALS7B	PCP4L1
RAB27A	SFXN1
MIR339	CYP3A25
VPS53	PFKFB3
MAT2A	PML

CCR3	EPOR
EIF5A2	GRK5
DNASE1	REL
ADPRH	MAOB
NTN1	KLF15
ZBTB20	CD55
NPY	DKK1
VKORC1	ACY1
RBM38	ANGPTL2
ANKRD11	BMP5
HJURP	BORA
HTRA2	EPB41
TET2	FGF12
FGA	MYCBP2
TSPAN32	NMT2
PAH	PSMD14
DNMT3L	RGS10
CCL18	ASGR2
UBC	ERMP1
PROS1	MLPH
PRDX4	NDUFS5
SGK3	NRBP2
MIR188	S100A13
BMPR2	TTC39C
HBS1L	CRY2
PRSS8	MYH11
ARHGAP5	PLA2G6
PBOV1	PRDM2
TNFAIP8L2	LAMA3
EPHA5	MSN
RHOD	COX6B2
MDH2	CYFIP2
PRMT1	MRPL17
PRKG1	PPM1G
CLDN9	PPP1R10
PTPN6	STK17B
RUNX1	SURF4
LINC00052	HP
USF1	FGA
ID2	AP3S1
DNTT	PGS1
MADCAM1	CYP3A7
PHLPP2	SULT1B1
KLF9	ACOT7
NUMB	ACOT9
RPS6	GGH
CXCL5	GNA14
BLCAP	ITSN1
MTHFD1	PTBP1
FOXP1	RAI14
FOXN3	RCN1
PGD	S1PR3
PGK1	SH3BP5
TLN1	ST3GAL6
TMEFF2	TSPAN8
SAR1A	YPEL5
PAGR1	ACOX2
MAGI2	ANK3

MTR	CLEC10A
OGFR	EPN2
SELP	LAD1
LIMD1	LIMK2
EEF2	RCL1
PER2	RPL28
MR1	SLC28A2
SCO2	TUBA8
TWNK	WDR26
KAT5	WSB2
CYP27B1	PDE5A
PANTR1	HBP1
KLF17	HYOU1
P2RX7	BBS2
CCR1	CBFA2T3
CLDN5	CTSF
CISH	EIF2B3
RALBP1	RBM19
NOX4	SSRP1
DCTN1	COX1
MAML1	STC2
PTPN9	POMC
FGF23	PRKDC
NEK2	AACS
FOXK2	AKAP9
SLC2A2	ATP6V0D2
G6PC3	CAD
TUBB2A	CD81
CCR2	EIF2S2
FLI1	FETUB
LGALS4	LBR
DCN	PCSK5
TEP1	PSMB2
MIR873	PTPRO
CCT3	SLC7A8
MIR99B	TOB1
VIPR1	TRIB2
PTPN22	CPN1
MIR1468	FADS3
KCNH1	KRT4
AOC3	PKIB
MYLK	PTER
TRIM26	RAD51B
AGAP2-AS1	RALGDS
NHLRC2	SLC38A3
BCR	BCAT1
MIR1285-1	CISD1
PAWR	CLEC2D
PAK2	FMO4
TAGLN2	HNRNPF
KMT2B	PPP3CC
ELP3	TCOF1
HDAC3	C1QC
SEPTIN9	MAPK13
UNC13D	STARD4
TAB1	STK11
RCVRN	GLDC
IGBP1	PTPRC

SPAG5	AGXT
CLCA4	FANCA
FUBP1	PBX1
APOBEC3B	PSMC2
SETX	SLC27A5
RGMB-AS1	IDH3A
MIR505	IL12A
PRKAB1	PNRC2
NDRG2	SCCPDH
CCL25	APOH
TPMT	FUCA1
IRAK1	TRA2A
TUBB3	TRIM16
CD55	CHST11
PLA2G12B	DYNLT3
POTEKP	MAD1L1
RAC2	PGPEP1
HPR	PPA2
ANGPTL4	REV3L
GATA1	TFCP2L1
COMMD1	XPOT
SP100	AKR1D1
VILL	PRPF6
RAP1B	SLC22A4
SRF	SYNCRIP
POU5F1B	TWIST1
MBD2	CBLB
EMP2	GEM
NLRP2	NPPA
NUP210	SCNN1A
DMTF1	SLC47A1
RARRES2	CMPK2
H4C1	FXYD5
CD22	FZD4
ADAM15	GPX7
DIRAS3	MID1IP1
C1RL	PELI1
MIR512-1	CCNDBP1
FAM215A	TPK1
GNLY	ERRFI1
BCL6	APRT
NUPR1	CES1D
MIR33B	SELP
SPAAR	LRP11
FHL2	RPL7A
APRT	RTKN
EFEMP1	SAR1A
SPHK2	TMEM43
CDK5RAP3	CCL9
TRIM31	ACTG1
WDR20	GOT2
TUSC1	IFIH1
HPS3	RIPK2
CHIT1	TIA1
NOVA1	UQCRC1
TACC3	HSD11B2
RSAD2	ABI1
SPTB	ANAPC1

USP48	CENPK
TUSC3	CLIC1
MYOG	ECHDC1
PDHX	FOLR2
SLC25A12	GNG11
TGM3	PLA1A
SMURF2	SOX6
NTSR1	TP53I3
UFL1	AQP9
TAF1B	CYGB
UGT2B7	FKBP4
NRIP1	LTBP1
MIR671	CLTC
SLC30A5	CTBP2
ESD	EIF3C
DDX39B	FLI1
ISL1	NDUFAB1
CCNB2	PLP2
OSM	ANGPT2
RPS27A	FZD7
UCK2	BYSL
MAPK12	CREB3
XRCC4	FEM1C
TMEM30B	FGFR1OP2
TRPM7	IL18BP
GRIK1	RASA2
IST1	C8G
ERBIN	CHDH
GCK	PLXNB1
STAT2	SAC3D1
MAP3K2	SPEG
POLR2E	ARHGAP18
LINC01116	CCN5
FLNB	DHX9
NDUFAF5	KLF11
MBD6	PIGA
SLC25A2	PRKCQ
ERFE	RBBP8
PTX3	SORBS2
MAGEA9	SPRY1
GPSM2	XRCC6
ITIH4	GJB2
NDC80	PRDX3
CYP2C19	ATR
MIR630	EFEMP1
PTMA	NEDD4L
EPHX2	OLR1
STK10	EIF2A
AKIP1	GREB1
WASF2	TMPRSS2
RPS6KA3	AIG1
MAP1LC3B	HEXA
TCF19	PKDCC
CCL3	RPL4
BHLHA15	YPEL3
CANX	CES1
PRKCQ	TDO2
DDIAS	AGL

DDC	GCAT
ARFGAP3	RALA
SSTR3	RETREG1
MAP2K3	RIF1
MYH9	SHISA2
PDP1	TCF12
NEO1	ABL1
NFATC1	FLNA
PFKP	MT3
TRIP11	STC1
RELB	CCDC80
VPS52	CRYZ
GTF2H4	FIGNL1
MIR1-1	PAFAH1B1
NUAK1	RGS3
AMBP	SLC13A2
AHNAK	CAMK2D
NUP133	FGFR4
NUP85	ARSA
XDH	BRD3
SETDB1	CD63
IL16	DKK2
MAGI1	EIF1
ARF1	FBXO31
OSCP1	LECT2
CLEC4M	NASP
RXRG	SCHIP1
ASNS	SRSF1
CETP	TRAF1
DSG2	GSTM6
FUT8	ZFPM1
SSB	ANGPTL3
TMSB4X	CSTF3
THRA	DMGDH
GAL	FTL1
CGA	MAPRE1
RPS27	PARP12
APC2	CARS2
UPF1	ARHGEF3
CORO1C	TJP3
SMAD1	EIF2AK2
PCK1	GLUD1
CUL5	ATP1A1
ABCF1	EIF4B
MIR211	HMBS
GRB10	LAMA1
KMT2D	ZYX
PRKD3	GTF2E2
OSBPL5	ADRA1B
NME2	BNIP3L
FUT6	IL7
ATP8B4	IRF9
TMEM30CP	IFNGR1
MIR134	EIF4EBP2
NR0B1	JPT1
MIR582	SRSF11
TXNIP	DBT
AGGF1	DYRK3

CHD1L	ERCC3
SOX1	KLF12
NLRP7	MEOX1
CFL1	MXI1
CD19	PTPRE
HMOX2	CA3
MAP1S	CBX5
PAK6	COX17
PEG3	PFN1
MT-ND5	PSME1
MTA2	SIAH2
XAF1	B4GALNT1
MIR874	BLMH
KIF11	EIF4H
PMVK	INHBC
EFNB3	PES1
EPHB1	SH3YL1
EEF2K	ITGB6
NPPC	MBNL2
CLOCK	TRIM2
IGFBP6	CEACAM1
FOXG1	EGR3
FZR1	LY96
GP5	MFN2
NT5C	RRAD
ORMDL1	PI4KB
RTL1	NR3C2
PIAS1	CEMIP2
NR4A3	CITED4
SMARCAD1	FUT8
SMC4	LYZ2
CENPE	NEB
DSG1	STEAP3
TSPO	SYTL2
GCLC	ATP2A1
MIR489	ESD
RAG1	XPO1
SYNE2	AGPAT2
RARRES1	EIF4G2
ID4	FXN
RTN4	LPGAT1
MIR302B	RNPEP
SLIT3	RPL12
ENTPD1	SEC23B
GUSB	SNRPD1
ESRRG	ABLIM1
HSPA9	EEF2K
DIO2	GC
LHX1	HPSE
BLK	ITGA3
PRDX5	MSR1
KNG1	THBS2
FERMT2	MYL9
HK1	ABHD1
HNRNPH1	CIDEB
CMKLR1	MDFIC
CNR1	PTPN18
SERPINF1	SPINT1

PCK2	NTRK1
FUT3	ACNAT2
PTGER1	FCNA
GGPS1	CXCL3
MIR448	SIGMAR1
BLZF1	ETNK2
HSPB8	MTMR6
COL1A2	ALDH18A1
UBE4B	DACT1
NR4A1	MTR
ARHGEF2	PDHA1
CSTA	SDHB
TERF2	EIF4G1
ADAMTSL1	HEY1
RAD21	ACAA1
SND1	CGREF1
PAK4	EI24
KDM6B	IL17RB
MTF1	NCOA4
EIF3H	RAMP2
SOAT1	RHBDF2
PSMD9	SKAP2
STUB1	CYP4A1
FLNA	CAPNS1
POTEF	CYC1
MIR194-1	DAAM1
PELP1	EEF1B2
SERPINH1	FGFBP1
RASSF2	HIVEP3
AATF	MPZL1
TERF1	N4BP2L1
SOX30	RPS14
TBL1XR1	TRIM28
TIA1	UBE3A
TEAD1	TWF2
MIR520D	EIF2B4
IQGAP3	FGD6
NOX1	LYAR
C10orf99	NDUFS8
TLL1	PAFAH1B3
PPP5C	TCIRG1
AGTR2	ALDH1L1
GDF2	CD59
ATP1A1	FMO2
SEMA3F	HDAC2
RECQL	TM7SF2
IL12B	HAS2
NUP160	MAP1B
SLC4A1	ASL
PNLIPRP3	GART
SLC25A5	HNRNPDL
DSC3	SLC16A10
PTP4A3	YWHAG
FABP5	STAT5A
BAG6	ABCE1
SPTBN1	H6PD
MT1G	IFI35
LLGL1	NAB2

HDGFL2	NOG
GOLPH3	PPP2CB
EHMT2	VHL
RLIM	SC5D
ELL2	ABCA6
PLIN1	ADAMTS7
MIR181D	BLCAP
CDC27	FARS2
MELTF	PHKA2
SPRY2	SMPD2
PRKACG	CACNB3
NUP107	EEPD1
AHCTF1	ETNPPL
NUP43	KANK1
UBE2T	RAB14
EGLN2	TIMM10B
HOXB7	APOA2
XAGE1A	AKR1B8
RASAL2	ATP7B
IFIH1	PSMA5
FGF17	RFC4
MSI1	FERMT2
CCKAR	GALE
LCK	HILPDA
FZD9	NPAS2
LPAR2	RAP1GAP
LINC01433	RPA1
COPG2	UBE2I
CBR3	BCKDHA
VRK1	CYB5R1
TIE1	DDX10
HOXA11	EPDR1
RBBP4	GSE1
PSAP	NMB
ATXN2	PDCD5
CLPS	AZIN1
EPM2AIP1	CD82
OGDH	SARDH
BACH1	SLCO3A1
PITPNM3	RASA4
FBXO6	SULT5A1
LPAR1	F10
ASIP	MB
AKR1A1	SLC1A3
FGL2	ASAH1
ADRB2	AKR1C1
E2F5	AKR1C3
ANLN	RPS27L
FBLN5	CHCHD10
PAM16	DUSP14
NELFE	ELOVL1
MEF2D	FRMD6
SRSF1	PAPOLA
KANSL1	RASGRP3
SUMO1P3	SLC22A2
MACROH2A1	ADAMTS15
MZF1	COL8A1
TIMM8A	EXTL1

PNPT1	HAND2
GET1	LMO7
ATP9A	NPDC1
LIVAR	SENP7
MSI2	SULT1D1
CGB5	RORC
GABRA3	ADAMTS9
STMN2	IL15
FOXQ1	PDGFC
BCL11A	STS
SH2D1A	CBX1
GSDME	CSNK2B
SKA1	CUX2
REN	FAM102A
EGFL7	H1-0
CRKL	SERPINA10
CBX4	SMYD3
PLEK	STK38
SCAP	SYT7
TAPBP	THRB
STYK1	AKR1C14
CCL15	ULK1
CBR1	GEMIN2
PRKAR1B	GRTP1
EIF2B2	EIF4A2
SNRPN	NINJ1
LRBA	SVIL
SLC30A7	KLF3
NLRP5	ME3
MSH4	MPZL2
RAB3IL1	NAV1
CAB39L	NFYA
ARFGAP2	RPL22L1
ANKRD49	SMTN
MAS1L	RELN
KHDC3L	ADAM23
FAM50B	COQ9
ASTE1	NEU2
MT-TF	WT1
MT-TH	IDH2
GPR1-AS	ECHS1
DGAT1	EMP3
AKAP13	LTBR
REV3L	NDUFS4
PPP1R10	PSMD1
BCAM	RHEB
BTRC	RPA2
SLC1A5	ADAM11
CACNA1G-AS1	BEX4
WDR77	IMPDH1
NEFH	MDM1
EIF6	SETD4
CBL	IL6ST
APOM	KITLG
RDX	TNFAIP6
CKS2	CTPS2
CLEC3B	H1F0
MT-ND4	PDZRN3

PAK5	PRELP
MIR367	PRG4
IRS4	SGO2
AKR1C2	CRYL1
LIME1	KCNN4
MUS81	PYGM
NEU1	ASRGL1
KLF10	CCNH
TXNRD3	EFHD2
PHF10	FXYD6
POLR1H	RAB8B
COX6B1	SERINC2
KIF7	SSX2IP
CT83	TAP2
BAGE	CTSK
C2	RET
MMP15	ACADSB
CYP4B1	ADAMTS5
ACADL	CSF2RB
HBG2	CSNK1A1
H4C13	LIPG
PLIN3	RPS15A
TET1	SDCBP
RERG	LAMP2
APEH	LAMP1
DPF3	OGT
SIRT2	PSMB4
USP22	TUBA1A
E4F1	CHFR
CTNNBIP1	HLA-DRA
BTG2	MRPL24
NDUFA4L2	MRPL46
CREB3	MRPS18A
TCTE3	CYP2B3
FCGR3A	CYP4A12A
BAG3	MPHOSPH10
SECISBP2	NUDCD2
TUBA1B	TESK2
TBK1	AFM
MIR101-2	AGPS
SIRPA	AOX3
DDIT4	ASAH2
PCDH9	DENND2D
EFCAB11	MRPL33
MAPRE1	PSMD5
GLUD1	STAB1
NSD2	UQCR11
MIR381	ACP1
GC	CYP2F2
ARRB1	HDLBP
SETD6	IER2
GLO1	LARP6
CSTB	PLEKHA5
LOC113687175	RFC1
LIN28A	SLC43A3
PLA2G1B	TDG
MIR138-2	TSPAN5
MINCR	BMP6

MIR622	FKBPL
P4HB	TMEM115
AGRN	ARHGAP26
MT1M	NUAK1
QKI	SLC13A5
PRDX6	BMP1
SP3	CRK
SUN2	EMB
GMPR	MR1
KLHDC2	MSLN
RPA2	PSMB7
LEPQTL1	BARD1
MIR422A	C4B
PSAT1	KIF20B
SPA17	XPA
GPI	NOS1
IKZF3	HSD17B7
LPXN	CYP51
CEBPD	APCDD1
DEPTOR	EEF1E1
LTBP1	MYO1E
IFI16	OXR1
PIK3C2A	PACSIN2
AKAP12	PRRG4
TRPC1	SUCLA2
PTGES	TREM2
ING5	CD4
H4C14	FBP2
AGO1	FRAS1
MIR454	GAB1
RPL18A	PID1
TUBA1A	POU2F1
PREX2	RAPH1
SMARCC1	STARD13
RIT1	ZFHX3
HM13	BANF1
DOT1L	COPB2
GPX4	GBP3
CCN6	GLTP
CBFB	PREB
FBXO30	RNF14
UGT1A9	RPS4X
GPS2	SCAPER
LPAR3	SERPINA3K
GIMAP6	BCL2L2
PRKAR2A	SPDL1
POGLUT1	STX3
KLF2	FAH
UGT1A6	GLIPR1
MYCBP	RPS6KA1
HCG11	CHCHD2
PLAC1	SHISA5
RAB5A	UGT1A2
KIF20B	PRKAA2
MB	PINK1
SYNM	ARHGAP8
LINC00668	CYP2C38
WASF3	EIF2B2

MYBL2	TMEM184C
LFNG	ZNHIT3
SPG11	NLRP3
SKP1	CDH11
SQLE	CLGN
MAEL	DBN1
SFRP4	HNRNPA3
LOC108251797	JAK3
CXCL13	SERPINA3N
ADCY10	SIRPA
GPSM3	TRIO
PIEZO1	AGRN
ADCY1	FGD4
ZNF251	FKBP1B
EIF4G1	HDAC9
DCST1-AS1	LPXN
KLF14	NEBL
SLC30A1	OSBPL1A
SMARCD2	PEA15
H3-3A	POLD1
ALOX15	NOTCH2
KANK1	RACK1
S100A11	KEG1
COX8A	DPYS
RPLP0	ENAH
MIR20B	ENTPD2
MAP2	H2BC5
PSMC4	LPCAT3
FUS	MADD
ZFX	MAPK7
AQP9	PPP1R2
ADORA3	SERPINB1A
STK19	TET1
PLK4	GPER1
GRAMD4	ITGA2
MAP3K7	TNFRSF9
YWHAB	GDPD5
VIL1	GFI1
OLA1	NIFK
BEX2	AATK
MAGI2-AS3	ARHGAP10
DND1	FMN2
SAFB	NOC2L
NID1	PPP2R2D
CCDC88A	ZDHHC23
STAT6	C1R
MRC1	GLI2
EFNA3	TNFSF9
STK3	TNNI3
DHX9	ADRB2
AGT	CDKN2D
STIP1	LIN7A
SLC34A1	SYN1
NKX6-2	TLR6
HES7	ALDH3A1
CFC1	SYP
MESP2	AK2
MYHAS	CDC14B

SSBP1	LMAN1
THORLNC	METAP2
CD47	PANK1
WSPAR	PDX1
SLC9A1	PXDN
CASK	SAMD4A
XAGE1B	SMOC2
TGFB1I1	TMED2
ORMDL3	TNFRSF11A
ANKLE1	TRAF4
MECP2	UBE2H
SLC12A2	WIPF1
CPD	WNT3A
TGFBI	MUP2
HLA-F	ACER2
MTHFD1L	ADSSL1
OPTN	CD151
SPNS1	CDS2
CREB3L4	ERO1B
BMP10	EXOSC8
MAP3K6	FAM114A1
ASPRV1	FLOT1
MIR302C	HSPB7
SOST	LGALS9
NT5E	NKAIN1
VWCE	PABPC4
UAP1	RPS16
IRF7	TMEM245
POLD2	CHAT
APOA4	GATA6
H4C11	CALM1
H4C12	BOK
H4C15	MCAM
MARCKS	PDK2
OPRM1	AAAS
TPTE2	MIR30A
TNFSF13	NDUFA4
GSDMB	PDZK1IP1
PTS	PPP2R5A
MMP16	RNF6
DPF1	SMS
RUVBL2	SPSB4
AIF1	UCHL5
NLRP3	WDR61
RND3	CXADR
CELF1	SAMHD1
TRRAP	NR4A2
HCK	ABCA5
DNM2	CUX1
DCXR	LPP
CLEC16A	TKFC
EIF4B	USP7
CX3CR1	ALDH1A2
TCF3	HAVCR1
FLOT1	SPTAN1
VAPB	BBS4
FBXO5	CYP2C54
BHLHE40	METTL9

TTF2	MRPS28
CDH15	PPP1R18
PSME3	RDH16
GYS1	VRK2
QDPR	ADORA2B
SLC30A2	ANO1
SLC39A8	BCAT2
RHD	CDC27
CD163	DLGAP4
KLK11	GNPDA1
SPINK7	GPHN
SOX17	MEIS1
PON2	NR2F6
ANTXR1	PIAS1
MICA-AS1	PPA1
COMMD7	RASSF5
CBS	SLC25A22
GART	BCLAF1
WWP1	FBLN5
SLIT1	CRYM
XPO1	PTPRK
RPA3	SLIT3
DPP6	TSHR
DBT	VDAC2
TRPV4	ALDH6A1
AHI1	PTHLH
TRIM44	BTC
DDX53	GIPC2
DACH1	MTMR11
CALD1	POPDC2
HLA-DQB2	TIE1
CCL22	ALDOB
MIR30C2	PPIA
NKD1	ADCY6
PFN1	ERCC6L
H3C1	FAM20C
SELL	HDAC3
DHRS9	OSBPL3
CR1	RUNX1T1
CAMK4	TAF1D
FOXO4	YBX1
TCF21	DPY30
MAGEB2	EIF3J
CHD1	MMRN2
SF3B4	ALDH1L2
SLC39A13	ARG2
ATP9B	ABHD4
ATP10A	DDX1
TEX14	FKBP11
GTPBP1	GCA
MOB3B	PTPN12
ZPBP2	PTPN21
COBL	PXMP4
SVOPL	TMEM176A
ZIM2-AS1	TRAF5
TAT-AS1	ALG14
FAT1	CYP4F15
OAT	IRAK1

PSEN1	LRATD1
SOX5	MBD3
HGFAC	MTMR7
ABCA1	NDUFA10
SP140	PGLYRP1
HLA-DMA	PODXL
MAL2	SLC16A6
B3GALT5	SLC22A17
CHL1	AMBP
ACIN1	MTSS1
SOCS6	NABP1
CDKL2	PIK3CD
CTSK	RPL13
ETV7	SYK
CD14	CD48
NCOA6	DNAJA4
IL37	ENTPD1
GNA15	MEIS2
FGGY	PDE7B
SLC16A1	SMPDL3A
SLC22A1	TCP11L2
BTK	CAPZB
TSPAN1	CRYAA
HNRNPD	NPTN
CCT8	ODF2
NBPF12	PEX19
NKX2-2	PLEKHA1
F13A1	PSMD13
DLGAP5	SAT2
GYG2	TMEM86A
SLC25A28	TNS2
PLCG2	TRIM22
POU5F1P4	USP3
CENPF	CYP2B13
FLOT2	C5
MUCL1	HMGA1
TFPI	IFITM2
MBOAT7	PRODH
IL1RAPL2	PTPN6
TNFRSF12A	SERPINC1
CA1	SLC4A7
MAGEE1	ATF6
MAGEE2	CLCN4
MAGEC3	CLK4
NSMCE3	FKBP14
THBS2	PELO
HOTAIRM1	PHIP
PIAS3	PPFIBP2
MX1	PSME3
ING4	PUS1
NDUFAF2	SLC43A1
MAFB	TYRO3
GALNT3	CC2D1B
BCL2L2	TRIP10
CAV2	ACTG2
MGAT3	DDX17
SNHG8	PDPK1
ANXA2P2	IL13

RDH11	APOA5
CNTF	ASPA
CXCL2	CD8A
CAPN2	DNAJC12
CYB5A	GNA12
RGN	H2AZ1
TMC2	OIP5
PTPRU	RASL11B
GBA3	RHOQ
UIMC1	SRSF3
IL17F	DMTN
QSOX1	LIN7C
ACTR3	LSM4
TMSB10	NFS1
MAP4K3	NLRP12
DDAH2	OGFR
FXYD5	PRMT7
PHLPP1	SEM1
MIR19B1	CD80
CORO1A	C1QBP
BAZ2A	CCT5
IL1R1	ENDOG
SERBP1	HDAC4
MIR7-3	INCENP
CREB5	MFAP4
TATDN1	NBN
NR2F2	RPS2
ATF6B	TGFB1I1
PTPRD	TXN2
SOAT2	AQP2
HLA-DRB5	DDB1
MTCO3P1	DHTKD1
LMNB1	EVA1A
IL12RB2	HERC6
LIPC	KPNA4
IL23R	MAN2B2
PYCARD	MXRA7
PPP1R13B	MYO1C
SLC46A1	PITPNM1
PTPRT	POLR1H
BCAT1	PTPRB
EPHB3	TCEAL9
MIR508	TOB2
IL23A	CRISPLD2
SAR1B	SLC39A14
RELN	CARD10
PTPRS	COBL
PSMB4	GARS1
TRIM29	GPR155
GNL3	NFATC3
TOP2B	OTUB2
MIR492	PHLDB1
PLA2G6	PIK3CB
MAP4K4	RRP12
CDK10	SGK3
SMC1A	SMIM14
PRPF19	SRI
SLC8A1	SSBP2

POMGNT2	MXD1
CDK9	PDE4D
FCGRT	AMD1
ERP29	ARHGEF2
FOXC2	BAZ1A
CDK8	CHAF1A
PCAT14	EBF1
IRF9	MAP2K5
ILF3	MAP4
CFD	PHEX
BPI	PLEKHF1
FIP1L1	CNN3
OBSL1	FBH1
DENND1B	VAV3
RIPK4	VNN3
EPRS1	SLC51B
H1-3	FGFR3
TUBB1	EMC9
MARK3	ERCC8
FAT4	ETFBKMT
PKD1	NOB1
ARHGAP35	PFDN4
ALKBH1	TXLNA
PPFIBP2	CAB39
RPS6KA6	CBX2
FKBP4	ILF2
PDSS2	LAPTM4B
CARD16	MAPRE3
UBAP2L	MYOM1
PES1	SAA4
FNDC5	APH1B
CX3CL1	B4GALT6
PDK1	CDC42SE1
FBL	MCOLN2
TRPV1	RERG
FLNC	RFFL
CXCR6	RPL30
HLA-DQA2	SLC35G1
HLA-DRB6	F5
CYP21A1P	B4GALT5
PBX2	CAMK2G
SLC13A5	DNAJC2
PTH1R	ITIH2
EIF2AK1	NXF1
CENPB	ONECUT1
GAS6	PCYT2
IFT88	RHPN2
DUSP19	TMTC2
AHSP	TSHZ1
CNN1	HFE
KIF5B	MSX1
MAGEA2	TRA2B
PBK	ADCY7
MIR190A	F13A1
ANXA8	GPRC5A
HNF4G	PDE4DIP
TRIM65	TOP2B
SGK1	CCL20

DTL	CAMK2A
VASH1	ITGB3
RPH3AL	ARHGAP22
PTGES2	ASAP1
ACACA	CD3D
PRPF8	CLCF1
CRISP3	DAP
RLN2	DPEP1
HBE1	DTYMK
ATPAF1	ELK3
FBXL2	FMR1
SPCS1	GCGR
EGID-105180392	GZMB
APOBEC3A	IP6K2
ATG7	KNL1
GSTM4	NRXN1
DACT2	PPP1CA
TNFRSF1B	PRIM2
TM4SF1	PTPRN
TFCP2	RBBP6
LOC111589215	RECK
CMTR2	RPL15
DSC2	SAA2
KLF8	SLC22A18
MIR7-2	SLC23A1
PGM1	SOX18
YWHAH	TBXAS1
CLCN3	AGO2
GSTA2	AMPD3
LNCNEF	ATP1A2
KDM3A	LCK
PDLIM7	ACOT13
TICAM1	BEX1
GYG1	FXYD3
ALS2	HAAO
KLF1	KHDRBS1
FIG4	LACTB
PANK2	LEAP2
SLC33A1	MECR
HBG1	MYO6
EIF2B3	PTGR2
DNAL1	SLC39A11
PANK1	STK4
UNC13A	WDFY3
ACY3	GNAI2
ATP8B3	CBLL1
ATP10D	CDK18
HBD	CLPP
SHOX	CLPTM1
NPRL3	DERL1
PGM5	HUNK
TBC1D20	PRKAR1B
KLHL12	SYCP3
ATP10B	TWIST2
SLC37A1	WDR36
SLC37A3	COL2A1
ZNF597	SBDS
PATL1	CYP2C40

INSL6	DHX33
RILPL2	EPO
PPP1R3D	OTUD5
DDX60L	CLK1
PPP1R3E	MIR21
C4orf3	MYCN
MESTIT1	NPC1
COPG2IT1	AK1
LOC108783645	E2F7
LOC109951029	SLC34A2
MAPK11	SLC5A3
ABCB5	ABCF1
DPP9	DDR1
BTG1	CRABP1
CCHCR1	DAXX
CST3	GSPT1
HSPA1L	NBL1
EIF3D	NDUFA5
UGT1A	PROX1
CUEDC2	RPS24
E2F7	ADI1
KAT8	CCAR1
UBE3C	CLPX
EIF3C	CLSTN2
PRRX1	E2F5
AQP5	FABP6
BABAM2	GOLIM4
OCIAD2	ITPA
RBBP6	MAMDC2
SMURF1	NIPSNAP1
HLA-DMB	NME7
HLA-DOA	OSBPL6
HLA-DOB	PI4K2B
HLA-DQB1-AS1	SLFN5
BCL9	TIMM8B
CEP55	TPST1
ZKSCAN1	PPP1R1B
EIF2S1	ACOT12
RPN2	C4A
TUFM	CHD1
ARHGDIA	DMTF1
CHD5	IGSF6
SUMO1	MPHOSPH6
NR2C2	RNPS1
EEF1G	SNHG11
PTBP1	TAF9
HINT1	UTP20
KCNJ5	DUSP2
SAA4	HSD17B10
CKAP4	RASSF4
DUOX1	VKORC1
PDHA1	CSF3
LPA	A1CF
H1-1	AMOTL1
CDK12	DBNDD2
ELOVL6	NADK
SELENOP	NRG4
LAMP2	NUTF2

H1-5	PLSCR2
BCL2L12	SULT1C1
GTF2B	TBCB
ACSL5	ANKRD11
EIF3I	CYP2S1
WASHC4	DNAJB11
SOCS2	ELAVL1
WNT8A	NEAT1
MIR619	YWHAH
H1-4	DYRK2
PHB2	PARP9
FNDC3B	PPIC
POLE2	SPINT2
TUBG1	SLC8A1
UTS2	ATP5G1
CSN2	EXT2
SLC6A2	UBQLN4
SPON2	ABHD14B
SRSF2	ARF6
MAGEA12	ARMCX3
FDFT1	CYP2U1
CSNK1D	FANCC
ADAM8	IFNAR2
HIBCH	MAGED2
TLCD3A	NLN
HOXD10	NPEPPS
NOG	NPNT
IL18R1	NUMB
BCL2A1	PDZD2
RAB38	PPP1R15B
NEK7	SERTAD2
WNT9B	ATP6V1D
ACVR1	COL14A1
RPTOR	IKBKE
MAPK6	SLC25A5
GPANK1	APBB3
CXCL11	APLP1
COX4I1	EDA2R
TXNDC5	NPPB
MEF2A	PLSCR4
CCL21	RAB43
SULT1A3	RFX4
GSK3A	S1PR5
KTN1	SLC25A19
CDKN2D	SMARCD2
MIR1307	ST7
RETREG1	TRAM2
ATF5	VPS13D
TRERNA1	MIR16
MIR513A1	NAT8F1
ACLY	DNM2
HDLBP	ITIH1
ASH2L	LPAR6
HTRA1	PLXDC2
EYA4	PSMA6
PSORS1C2	SLC44A1
HCG22	SLC6A12
HLA-J	TFR2

ENSG00000272221	YY1
MN298114-200	CEBPG
ERRFI1	HDAC5
SELPLG	MAP1LC3A
AIM2	MFN1
AKR1C4	WIPI1
PCBP2	ABHD15
EHHADH	MMD2
PROK1	NCAM2
CCL1	PHC2
ABO	PORCN
ABCC5	TMEM53
TRIM21	MTTP
SKIL	BEX3
PCDH10	CDK9
ACTN1	CETN3
CCT2	PLOD3
NR6A1	PTPRS
HSPB6	RAB1A
CAPNS1	RBMS3
BPTF	SLC16A12
SDC4	SORBS3
PAXIP1	SRPK2
TRIM39	UPP2
PIWIL4	FHIT
GSTA3	MCF2L
GRK6	PMVK
RLF	YWHAQ
APOC2	LMNB1
UBE2I	NPR3
GAB2	PNLIPRP1
USP15	ACSF2
MIR613	ACSS1
LRIG3	CCT7
CRK	CORO1C
TSTD1	FNDC5
UGCG	G2E3
USP10	LGALSL
TRIM11	MLEC
USP14	PRF1
IL22	RPS15
ALDH9A1	STRBP
KHDRBS1	SYNGR1
FRS2	USP14
SOX11	ZWILCH
YBX3	ADAM15
GLIS3	ATP11C
SRRD	CRYBG1
ITPA	CTPS
HILPDA	DYNLL2
SIRT5	GRAMD1B
TLR8	MATK
PNN	SLC14A2
ICAM2	TMEM33
ISG15	TOX
SFRP5	TTF2
FASTKD2	UNC13B
MIR657	USP47

MRC2	SCP2
SVEP1	CDK5
CEACAM4	NEK6
AZGP1	PCDH7
HSPG2	SGK2
RNF11	ABCA8A
COL11A2	ABI3BP
NKX6-1	ANXA8
HRH2	ATF7IP
DSC1	BAIAP2L1
CHD4	CHTF18
TPT1	COQ3
LMTK2	CYP2C55
LOC110283621	EFNA5
ST3GAL4	ESPN
SPRED2	GTF2H1
HNRNPDL	MUTYH
CUX1	NRTN
BIRC6	PANX1
MIR493	PPID
SOX21	RTN4RL1
ERO1A	SELENOM
FBLIM1	STK10
PNP	TPP2
CHSY1	WWC1
PABPC1	HK1
CBX7	SLC1A4
DHX16	ADORA2A
NFKBIL1	ATP2B4
NRM	BTBD3
LY6G5B	CRADD
HCG4	IFI27L2B
RPL3P2	PDZK1
ENSG00000272501	RBBP9
ENSG00000199332	SIK2
ENSG00000271581	SIRT3
MGLL	ANXA7
ZHX2	CNDP2
DUOX2	EHBP1
KIF4A	HC
TBXT	LZIC
SLC5A1	PARD3
CAMK1	SEMA3B
SCYL1	SERPINB1
CRAT	TRIT1
PHGDH	E2F2
FOSL2	SLC5A1
SYCP1	EDEM1
HDAC8	NFKBIE
RAB1A	NSD2
SLCO2B1	RGS1
CD83	RPLP0
JAZF1	TPT1
CUL7	BICC1
POU2F1	GALNT15
ABCD3	GATA2
NDN	GEMIN6
MUC15	NARF

GPC1	NR2C1
MIR379	PRODH2
HOXA9	RAPGEF5
NPPA	RPS13
WDR46	SMARCA5
ITGB5	STT3A
NAP1L4	TNK2
TLK1	VGLL4
CRHR2	CYP27B1
PARD3	LMCD1
EGFL8	RSRP1
PPM1A	SERPINB9
LSM2	CTDSPL
DRG2	LIMK1
RPS18	LONRF3
ESRRB	MMP16
P2RY2	NDUFB2
S1PR1	PARVA
MIR638	PCOLCE2
SOX12	SUV39H2
MMP21	TIMM9
MYOD1	TOMM40
SIX2	DOK1
CENPH	PARP16
PGRMC1	RBP2
MYO6	RNF186
ETFDH	TMPRSS6
CBX8	AQP4
ADH4	MYH9
PHKG2	RIPK1
PSG9	CORO2A
CSF3R	EIF3B
CSNK2A2	FGR
GPLD1	FJX1
HNRNPA1	FOXN3
SHC4	PRKAB1
USP4	SF1
JDP2	ST13
LRG1	TANK
RPS6KA1	TRPV2
EFNB1	WWTR1
GKN1	BLM
USB1	CA12
NLRC4	CAMK2N1
WFDC1	CAVIN2
NCBP1	CSK
CORT	GBP1
DIO3	HOXA1
NID2	LAMB2
IL27	MDH2
KIF14	PRKAR1A
RUVBL1	SLC51A
TPM2	BHMT2
RP1	CABYR
NLK	KHDRBS3
POR	MLLT6
TUBA1C	MMP17
NDUFS4	PFDN2

CTSS	PKN2
HAX1	SART1
PSORS1C3	TSPYL4
HLA-K	VSIR
HLA-W	ZCCHC14
ENSG00000272540	KRT17
ENSG00000237669	LRP2
RPL5	NRP2
MIR1204	PPP2R2B
CFHR1	SPRY4
MIR421	NTF3
THPO	ATP6V0B
ADAM28	FAIM
ANAPC11	FN3K
CYP3A7	KPNA3
ILF2	NDUFA9
ATG3	PCYT1A
RORC	RPS18
PNPLA2	SNTG2
CAMP	SPG7
PYGO2	ZBP1
CDO1	UGT2B7
CAPG	ARSB
YWHAQ	DNAJC9
COL3A1	GLG1
FOXF2	MANBA
KCNN4	MDN1
CD74	PKP4
TCP1	SERP1
CCL17	AGR2
STK17B	GRPEL1
NAP1L1	HIBCH
TNFSF9	MATR3
OSGIN1	RAB27B
UHMK1	RNF144B
TTLL12	RNF19B
LIMS1	SCARA3
UBB	SEC24D
KRT81	SP3
ARHGAP1	DHRS7
SHC2	EEF1A2
DHDH	UQCRC2
RACGAP1	TMEM86B
SH3PXD2A	ALKBH7
GEMIN4	COQ5
PHF8	DDX27
PER3	LAG3
PRICKLE4	MTERF2
MIR384	NCBP2
ST7	POGLUT3
CDCA7L	TBC1D10A
KPNB1	PSTPIP1
SMARCD1	SP110
MIR765	YIPF5
PSMC1	ACAA1B
SERPINA4	ANXA13
B3GALT4	ATXN2
MIB1	CMTM3

IER3	CYP4A2
UBE2Q1	EIF4E2
SRSF3	LRATD2
SKIV2L	MEGF6
FKBPL	NETO2
MIR194-2	PAK1IP1
MIR940	PCBP4
MRPL45	PNKD
SMOC2	PRPF19
GNA12	PTBP2
FOXD3	SRPRB
UCHL5	SRPX2
CCDC137	ST3GAL2
SPRY1	UBE2D2
CSNK2A3	ADAM12
GSTT2B	CASZ1
ZYX	CDH17
CCL14	CLIC3
ABCC6	CRTAP
PIM3	ELN
MIR1297	EWSR1
NCAPG	MBNL3
TTF1	PCM1
YTHDF2	PSPC1
AKAP3	ABCC9
POLR2L	ACTA1
BCORL1	DOCK8
KLKB1	FGL2
SYMPK	GTPBP2
MIR1269A	LIPA
PITX1	NGFR
ANAPC1	SON
USP9X	ST8SIA4
SMARCC2	TUBB3
HADHA	AGTR1
HSPE1	GSTZ1
APOBEC1	HNRNPK
ZNF384	LCP1
CERS2	SHMT1
ISG20L2	REEP6
CCT4	RRAGD
NDRG3	SLC38A4
E2F8	CLIP1
C5AR1	GNA13
KRT6B	MEST
RNF8	PRKAB2
TRIM39-RPP21	RPL10
EED	SRRM2
ZNF592	SYT1
SIAH2	UGP2
CDC37	UTRN
SSX2	ABTB2
LTBP2	AKR1B7
GATA5	AQP5
UNC119	ATP6V0D1
MUC17	BIN1
CTBP2	DDX6
SMTN	LATS2

GZMA	MIA2
SOX7	RAD54B
PROM2	REV1
TRIM35	RTP4
SUOX	SEC31A
LUC7L2	TLE3
P2RX4	TMED10
SMARCD3	TOPBP1
NOB1	XRCC4
UGT2B15	SNORD22
PPT2	C4BPA
SAPCD1	CPEB1
SNHG32	ERP27
MICD	FPR1
ENSG00000288587	GALNT7
HSALNG0049430	GTF2H5
NONHSAG045982.2	MTHFR
LOC107986589	NDUFA13
ADAMTS5	PPP5C
PGAM1	PTGR1
LINC00882	SETD7
BOK	UQCRFS1
TBP	VPS13B
HSD17B8	WDR12
NEDD8	CYP24A1
MT-ND2	AKIRIN1
PDCD5	ANAPC5
LAP3	CERT1
BCLAF1	DEDD
PTPRO	HAMP2
HNRNPU	KRTCAP2
FOSB	LAMTOR2
TFAP4	MIR200B
PDLIM5	MYO18A
MAGED4B	NFX1
H4C8	OBP2A
MIR539	ORMDL3
MACF1	RSU1
MYCBP2	SAP30
ZMYND8	SLC25A34
HNRNPH2	STRN
PPIG	TOR1B
TNFRSF14	TUBE1
USP28	CHID1
TNKS2	CYP2C7
SUV39H1	TCP11L1
MEF2C	PGAM1
KRT12	CD24
MT-ATP6	CD69
MAOA	PAH
P4HA2	SLC7A7
PPP1R3B	BAG2
FGF18	CAMKK2
ENPP7	GSTP2
CXCL17	KLHL21
CCT6A	OAF
CETN2	P2RX4
PARVB	PFN2

SOX18	PTPN11
APLNR	S100P
TAF11	AJUBA
SPTBN2	CYP2C23
PTAFR	GRHL1
ADAMTS1	KDM1A
FSTL1	MMP11
MT2A	MRPL12
ZNF382	NELL2
EIF4A1	PHF20L1
HECTD1	PURA
CAMK2G	SPRED1
IQCE	PMEPA1
SEPTIN4	TUBB
SPOCK1	TIPARP
FOXK1	ADIPOR1
ACTL6B	BSCL2
C5	CDK8
PPP6C	CHCHD3
CRNKL1	DDOST
BAIAP2L1	EPS15
HNRNPF	GREM2
VMP1	H3F3B
CAPZA1	MACROH2A1
HNRNPAB	NMT1
TERF2IP	PGAP2
SOD3	PLEK
LRAT	PLPP1
MIR133A2	PPP2R2C
GRK2	PTP4A3
GAGE1	SIN3A
KRT78	TMEM14A
GSTT2	MUC5AC
RNF14	BAG4
SPI1	COPG1
SLC29A2	FKBP3
MIR1271	GGTA1
PCDH20	KRIT1
BLVRB	LIG3
PRDM5	MOK
EXT1	RASAL1
FAM189B	REEP3
AMY1A	SMC6
MIR589	SNED1
MIR766	SNRPA1
SLC25A17	SUCNR1
OCLN	TMC7
ZKSCAN4	VEZF1
EIF2S2	ZMYND11
NR4A2	HSD17B3
TAF1	ADAM19
NAT10	CHN2
RNF2	OGN
MORF4L1	OXTR
FKBP5	PSMB6
CARD9	RANGAP1
CCL19	SLC25A33
SETD7	UBE2D3

FOXH1	WDHD1
KHSRP	CASKIN1
TKTL1	CDC16
PSMD2	GABPB2
NEIL1	HOXB3
DNM3	KCTD3
LETMD1	LILRB3
PLOD2	LST1
CYP20A1	MRE11A
CNR2	PAQR7
RAB35	PCBP3
HUS1	ROGDI
LAMP1	TSTD1
LAMA2	USP8
ZIC1	AGER
APLN	ATOX1
TNFRSF18	EHD2
EIF2AK4	ENDOD1
CPT2	ITIH5
ALYREF	JCHAIN
ACSS2	MAGI1
GPA33	PCDH17
LTBR	SLC30A10
DXO	TOR3A
HCG25	WIF1
RPL23AP1	PHF12
RPL32P1	PTPRT
MIR520A	TRP53INP2
SOX6	GCHFR
PPP1R12A	KNSTRN
G3BP1	ITGA4
MTA3	AKTIP
PSPH	ATP11A
RORA	DDX46
BCL6B	DERL3
XPO5	FKBP10
POU2F3	G6PDX
CSNK1A1L	MICAL1
HOXD3	OLFML1
GARS1	RPL27
WEE2	TAOK3
MAP3K3	ACTR3
COPS3	AGTR2
PTOV1	BCAR3
ORM2	CREBRF
RPL38	EPB41L1
MGST3	GCNT3
FRAT2	LAMA2
PUF60	OAS1
ZC3H14	RPS6KA2
ADAMTS14	ARID4A
MIR302D	ATP10D
ADORA1	CD27
DKK4	CERS5
MIR1246	MIA
DDX39A	OSBPL5
RPLP2	PELI2
FSTL5	REEP2

RITA1	SMG1
HSD17B13	ZHX3
TLE3	IFNB1
RASSF10	TM4SF1
DKK2	ATOH8
SRY	COX6C
RGS17	HDGF
DNAJB4	HMCN1
H4C3	LPCAT1
TCIRG1	LSR
DCD	PEG10
CD2BP2	PHB2
PIK3IP1	PLXNC1
SLC19A1	SNAP23
KIR3DS1	CRY1
ITCH	ACTR1A
CD226	ACTR6
UCN	ASH2L
CYP2B6	ATN1
INTS5	CCDC28B
IRAK4	GET1
AARS1	PCSK4
AVPR1A	SLC25A42
CD2AP	COLEC12
MGST2	ERBB4
GSTM5	GFRA2
GSTA5	GLA
NFATC2	LPO
SLC39A7	MSX2
CASP14	NAV3
H2BC14	RAD23A
KDM5A	RANBP1
NFIC	SKIL
HIF3A	ABCB9
GGCX	AHCYL1
KLF12	APLNR
MT-ND3	BST1
CXXC5	CGN
ALX4	DOCK10
TROAP	GFOD1
PLAAT4	HKDC1
EPHA10	ITGB7
CYP2J2	PILRA
PTPRA	PIM2
NODAL	QDPR
BTBD7	SAP18
MIR545	SCLY
PI4K2A	SLC16A5
SHPRH	SLC24A3
CARD10	SLC38A5
PRR11	TBX3
SKI	TFB1M
CYP26B1	UBE2K
FOXP4	WDR4
MCM6	CRABP2
EDN3	IL11
RXFP2	DHX38
EIF5A	HAPLN2

SHMT2	STK11IP
HYOU1	TBP
DNM1L	ATP6V0A1
METTL21A	DDT
VPS54	EVL
EEF1AKMT3	HSD17B12
LINC01451	MBNL1
ENSG00000216663	MEOX2
FADS2	CLEC4D
COMP	COMMD10
ANP32A	EGFL6
POLI	ESYT1
ATP5F1A	GLT8D1
CLDN2	MYH1
OGT	NCOA6
NACA	PNPO
ZNF418	RNF144A
MCM3AP	SLC35B1
BNC2	THRAP3
CCNL2	TRIP12
MIR300	WDFY1
TP53INP1	ZNRF1
IRAK3	B4GALT1
EVA1A	COL7A1
FUT7	CTNNAL1
GFUS	FAM162A
PIK3R5	FAM214A
HSPH1	IL2RB
HNRNPL	ITGA7
KLB	LRP8
MTRR	PPP1CB
TUBB4B	PRICKLE1
SSRP1	TTPA
ATP5IF1	TRAPPC4
ZNHIT3	ADGRL1
ISX	AGPAT4
MBNL3	AQP8
BCL2L10	COX7A2
CTPS1	HABP2
CENPA	HAUS4
IL11RA	LAT
MRTFA	PGRMC2
PAPPA	PMM1
MIG7	RFC2
PEX1	SLC9A3
SGTA	SMURF2
SYNJ2BP	ST3GAL4
UBR5	ULK2
ACP5	CCR7
TNIP1	BHLHB9
DUXAP10	DMXL1
DLX4	OGFOD1
EDIL3	QTRT1
EGR2	SLC49A4
ATG4B	STAM
NUDT1	SULT2A2
SERPINA5	TERF2IP
PPP1R15A	WBP4

B4GALT1	IL6R
MIR216B	ACAD9
GLDC	ADAM9
RBBP5	CIT
H2BC4	DHX58
GABPA	GASK1B
RFX1	PYCR1
MT1X	RFTN1
GCNT3	TRIM25
SUPT3H	TSPAN4
MIR718	ATP2B2
AMER1	CCT6A
AKR1B1	CHD2
CCT7	DLL1
RAB11B	PARP14
TSIX	PFKM
NEFL	PTGIS
AICDA	STEAP1
MIR570	GUCY1B1
BTG3	GUCY1A1
IRF2	KRT10
F2RL2	AGXT2
ING2	CES2E
RNASE3	LIMD2
PCDH17	MRAP
STRAP	MRPL18
NCK1	NDUFS2
ADAMTS13	NELFE
KRT9	PPP1R14C
MRPS34	PSTPIP2
ARG2	RPS25
UBE2S	SALL4
TRIM31-AS1	STARD5
RNY4P10	USP53
HLA-DPA3	AZIN2
ENSG00000232080	H4C11
ENSG00000259053	MEIS3
PKHD1	MUP5
MYO1B	NAT8F5
SLC35A3	PRUNE1
UBR4	TBX6
PDCD6	TMEM199
PIK3C3	AIMP1
PFAS	IFRD2
ITGAM	IGSF3
H2BC21	NDUFAF4
TARBP2	NOC3L
GOT1	PTPRU
OTUD7B	RNF128
MT1F	TBC1D8
TEX10	TRAM1
TUBB2B	ALOX12
CD5L	CLEC7A
KIF20A	CTSZ
UTS2R	DNAJC10
MIR301B	DUSP3
LOC111832671	HTT
SMYD4	LY86

FRK	MACROD1
CTSH	NAA15
FAIM2	NKX3-1
FKBP10	PRPS2
TIGAR	SEL1L3
PET117	TACC2
STING1	UAP1L1
ENTPD2	WNT11
UCP1	ATP7A
H4C2	CCNL1
H4C4	DBF4
MFF	ERCC2
DHX33	LFNG
CDC42EP3	METRNL
DMWD	RPL31
ALDH1L1	SLC46A1
CLDN11	SOCS1
ZNF350	DYNC1H1
HOXC8	GREM1
MIR1301	SLC25A25
DLG2	ACP6
CSPG4	ADGRF1
ZBTB16	CDC123
C1GALT1	CUL5
MIR612	H2BC14
BNIP3L	MMADHC
NPR1	NFYC
PKP2	PEX5
VASH2	TTL
CDK3	ZNF608
CCT5	AGTRAP
ST7L	ARPC4
NPAS2	ATAD3A
ACSS1	ATP5MC1
BST2	CHD1L
IGFBP4	CHST14
EHD2	DEGS1
STK38	EXOC4
SLC34A2	FAM222A
CKS1BP7	FRK
ZRANB1	KYAT3
AFM	LIMS1
SETD1B	LLGL2
FBXO22	MYOM2
MERTK	PRPF8
SPRED1	SEZ6
SNAPIN	SLC25A29
GALNT14	TRMT1
SSBP2	UBE2S
GRHL2	WDR75
KMT5A	ZC2HC1A
RUBCN	CRYBB3
HOXC6	ALS2
BAMBI	CD302
ENDOG	CLSTN3
RPL39L	FCGRT
BMF	FLT4
WARS1	GPR146

PPP1R9B	MIR22
HOXB-AS3	MST1
TAB3	NDST1
BCL2L15	RBX1
FOXR2	S100A14
UBA1	SMC3
MIR484	ARID5B
CADM2	FNBP1
SHOX2	IVD
LOC110594336	KRT15
MAPKAPK5	PYGB
PADI4	SLC16A7
MSBP1	MYO5B
PTP4A2	NEXN
MIR135A2	PDLIM3
ARL6IP5	PLXNB2
MAGED1	PRKAR2A
ARHGDIB	RASA1
BTF3	RHOBTB1
MAT2B	RHOBTB3
TSPYL5	SLC12A3
MIR504	SLC6A13
SLC38A1	TNFSF14
GALR2	VAMP8
FUT1	ARL8A
KDM4A	KATNAL1
GABBR1	KLRK1
TRIM66	MRPL57
MIR548C	RPE
RCAN1	TMEM147
EP400	EDNRA
SCAMP3	ACTC1
PHETA1	ETV5
CBX5	LOXL1
HLF	NEDD4
CPEB3	EPB41L3
SALL3	NCOA7
MIR9-1HG	AGFG2
NDUFS1	CACNB1
VANGL1	FCHO2
TGFBRAP1	FOXP4
MIR365B	GTF2A1
GLG1	HM13
SRGN	ILDR2
MPG	MAGEH1
SENP7	MYLPF
PCBD1	PATL1
GSS	RBM34
PIWIL2	ROS1
CHRNA7	UBR1
ATIC	DYSF
SIGLEC1	EPHA1
NFAT5	H2BC21
PFKFB4	ITGA8
INCENP	MYADM
MUC13	NCALD
PIGU	OAS2
VPS35	PPP2R2A

HNRNPC	SEMA6D
NRF1	SFXN2
RASSF6	SIVA1
TRPC6	TNPO1
UNG	UBE2N
HERC5	ACSM1
VSIR	ADSL
MCPH1	AP2A1
PTPRF	ASAP2
EFNA5	BPNT2
GDI2	CHSY1
DHCR24	CKMT2
APOBEC3H	FOXO4
CDCA5	FXYD1
NCOA5	HR
FAM83D	IER5L
DFFB	KAT5
SERPIND1	LHX1
CES1	MAP4K1
CPEB4	NT5C2
NLRC5	PKD1
ARHGEF5	PLEKHG3
SCYL2	SCGB1A1
IRX3	SIPA1L1
SLC26A5	SLA
GINS2	SMAD5
ATOH8	SNRPE
NASP	SRRM1
MASP2	SURF1
RTCB	TMEM45B
MTBP	TTPAL
PPP1CC	UBE4B
FBXO4	ZFPM2
LCP1	KLK1B4
NSUN2	LPAR1
HMGB3	PRKACB
RPRD1B	RYR1
MIR650	MITF
YWHAG	ANTXR2
P2RX3	AS3MT
MAGEC1	CREB5
INSL3	AHRR
TRO	AZGP1
CCL8	FRMD4A
AQP8	HIVEP2
HACE1	RBPJ
PFKFB2	TBX21
RGS5	TFDP2
BLVRA	B3GALNT1
MCUR1	C2CD2
PDZK1	CAPN5
ARMC8	CBX7
NCR3	CD70
C1QTNF6	COL4A5
ITPR3	GMDS
CD200R1	HAO1
PDK2	HEATR1
MAP2K6	HEBP2

PRSS3	HNRNPR
IL10RB	ITPKA
GLP1R	JARID2
UGDH	PIPOX
RTKN	PREX1
PLAC8	RAB38
ADAMTS4	RPL8
AJAP1	SCN8A
PPP1R11	TAX1BP1
RNF39	TMEM140
VWA7	XPNPEP2
HCG17	PVR
lnc-HLA-DPA1-2	SLC1A1
piR-38257-014	CMTM6
CTNNA3	DROSHA
CCNC	DYNC2LI1
SSX5	ELP4
ABCB6	EML2
CSN1S1	FITM1
TRPM2	GBA
RPL4	H1F2
ZIC2	H3C15
CTNND2	HMGN5
CHST11	IFT172
PCDH19	LZTFL1
CAB39	MAGOH
EDARADD	MANEA
KRT28	MOCS1
KRT73	NCK2
HEG1	NEK4
GFPT1	NUS1
PPIL3	PHKG2
MBNL2	PPDPF
TNFAIP8	PRSS2
TRIM14	RPL36
TRDMT1	RPS21
FOXJ2	RREB1
RND1	SLC35D1
MIR644A	SLC38A6
DARS1	SOCS5
CMTM3	SSBP3
UBE2L3	SSH1
KIF18A	TMEM132A
ITGB8	AKR1C18
LTB	ASB13
CYP2W1	LARP4B
CKAP5	LMBRD1
IL9	LYPLAL1
GANAB	MTF2
ZGPAT	NSD3
PSMC5	SLC45A4
DHX15	TMLHE
SPANXC	CXCL13
POLR2A	GABARAPL1
RPS15A	SLC22A3
ASAP3	CXCR1
NDUFV1	GNAI3
COA3	GULP1

PRDM1	IFNGR2
MT1H	SCG5
CAMKK2	SGO1
MIR577	SLC41A2
KIR3DL1	SQOR
SSX1	ALDH5A1
TREM1	BST2
MIR433	NRCAM
LOC112529895	CSF3R
MIR1207	GNAT1
RBMY1A1	HIF3A
MIR203B	HMGCL
EFTUD2	PKIA
MGAT4A	PPAN
UPP1	PRXL2A
HSPA2	RAD21
PSMC3	SLC2A9
GORAB	SLC44A4
POFUT1	SPATA13
SENP2	TMOD1
ATG12	CDK5R1
WWP2	COX5A
CISD2	DFFA
ASIC1	KLKB1
UQCC1	PEPD
GLYATL1	APBB1IP
XCL1	APPL2
RNF38	ARSG
LRIG2	BTD
BNC1	CITED1
S100A10	GBP6
PDCD6IP	GPC6
NUDT21	HLTF
XCR1	IGF2BP2
PDIA6	MAPK8IP3
CBLB	MBD4
DUSP10	MIR222
PSIP1	PAN2
TNS2	RPL34
ASRGL1	SLC45A3
MIR153-2	TCEA2
MIR3127	TIMM10
SIK2	TNNT3
CDK13	ALOX12B
SLC22A17	AP2A2
PSMD1	ARHGAP1
PRKCSH	CAR14
ME1	CIZ1
H2BS1	CRTC2
TMOD1	CTLA2A
AMPD3	CYP2J2
SUZ12	FBXO21
DYNC1H1	GALK1
HAO2	GIPC1
FCN3	GPI
SMARCA5	JADE1
SLC22A2	LRIG1
ACVRL1	LSAMP

FGFRL1	MSC
SUMO3	MYL1
SDC2	NDUFS6
DUT	NUCKS1
H4C6	OBSCN
RAB11A	RAB24
C1D	RPS7
IQGAP2	SETD1B
PHF19	SNX24
MIR876	TMED7
PCNT	ADGRA3
STOML2	DOK2
GCNT2	FOXRED2
ALDH7A1	HOXD3
LPAR6	LSM2
CRYL1	MAD2L2
MIR592	NDFIP2
EMP3	PSMG2
GIT1	RIC8A
GNAO1	SSH2
TWIST2	TBC1D17
RSPO2	MUP1
MPV17	SHROOM1
NOD1	SLC17A4
USP18	NAT1
CTH	ELL2
SEC16B	INPP5D
HNRNPM	ARHGEF7
FPR2	B3GNT2
PCBP1	CDS1
MT1A	GSTO2
ANKHD1	IMPA1
CMTM5	MTARC1
LXN	NDUFA1
LOC107982234	NIN
H3-4	RHBG
PDE4D	SH3GLB1
MIR655	SLC5A6
PATZ1	STAMBPL1
SNW1	TICAM1
PIDD1	TSPAN13
FBXO31	WNT10B
ASCL2	CYP2A6
SDCBP	CACNA1G
ADAMTS9	ITGB4
IPO7	ABCA2
PKMYT1	ADAMTSL4
SUMO2	AHSA1
NUF2	ANP32B
H4C9	ATG4B
H4C5	CAR8
CIZ1	CLEC11A
BCL2L14	DHX30
FMNL2	GINS3
MRTFB	HDHD3
THOP1	JAM2
KDELR2	KYAT1
SEC62-AS1	LTBP2

GLRX	NAGK
CFAP45	NDUFV1
KRT75	NDUFV3
SEPTIN2	NXN
ACKR1	PDCD11
GTF2I	UGT2A3
VPS4B	VAV1
DHPS	ADK
POLR2C	BCL10
HOXA7	NCEH1
C11orf65	P2RY2
LOC108942766	SMARCA4
SNHG18	TXNRD2
MIR552	ABCB10
RAB18	ACSM5
ENTPD7	ATE1
GTSE1	ATP13A3
SETD3	EBPL
CCNY	FPR2
ATP6V1C1	GAPDHS
TNKS	HTATSF1
RBM3	MRPL45
NUP62	MS4A1
RNF181	P2RY14
LAMP3	PARP3
CAPN1	PIGP
CLEC12A	POLE3
CDC34	RAI2
EVI5	RP2
LRPAP1	SF3B3
SORBS3	SLC17A8
RAB10	SNRPD3
USP39	SPOCK2
NKD2	TM7SF3
RAB7A	UBE2G2
SLC6A3	AVPI1
IFIT1	BCKDHB
EBI3	DUSP16
CEP57	FXYD2
BRD1	OASL
CNBP	PRSS12
COPS6	SYVN1
PROCR	TIMM17A
TFCP2L1	CFTR
SRXN1	MARCKSL1
MIR1236	PRKD1
CCN5	PSMA3
TNFAIP8L1	TBC1D4
ANP32B	TCN2
NRSN2	TRPM4
TGIF2	APOOL
CALM2	CYP2D9
CALML3	FRRS1
INTS3	MED27
PPA1	MRPL22
MARK4	NUP133
UQCRH	PHF8
PDZD7	RUFY1

MTHFD2	SLAMF9
DUSP13	TAF5
PDIA4	TEX261
PRSS23	BMPR2
BTBD3	CRELD2
H1-2	F11R
SAGE1	LTA
MIR942	VEGFD
KHDRBS3	APOBEC1
H2BC15	ART3
SYNCRIP	DEXI
FADS1	IRF4
CSAD	KCNA2
MATR3	MPP6
TIGIT	NEO1
ERAP2	NIPAL2
CDC42BPA	NUP155
DYSF	OPA1
IDE	PHKB
USP21	SCRN1
MIR634	WNT3
RBM8A	CHRNA4
FBXW8	CXCR2
TRIM52	MEF2C
SELENOM	MIR17
MIR298	SDR9C7
MIR23C	TATDN2
CORO6	CDCA7L
SENP1	CREB3L3
SARDH	FXR1
SEC62	GCKR
MYDGF	INTS6
UBE2V2	MMP15
AGA	PGM2L1
LOC109113863	PPIB
MASP1	RBM24
OVOL2	STXBP6
FUCA2	USP15
PRKCH	GAD1
TOLLIP	H4C12
CAPRIN1	LRTM1
NEK6	CDK7
DAD1	DCBLD2
CITED2	EHMT2
MKNK1	FAM111A
SCARA3	HIVEP1
CYP27A1	AIMP2
UMOD	CCDC50
OTUB1	CDK16
HGS	ERBIN
SORBS2	FRYL
DOK1	GLOD4
TUBA8	GMPR2
PHF6	GOLPH3
WSB1	MARK2
ACTR1B	MGAT5
PPM1F	NELFA
RAB17	RANBP2

BASP1	RHOBTB2
HRC	RIOK2
ARHGAP18	SUSD4
SH2D4A	TUB
VASN	ZFP91
RPL36A	SMC1A
ZNF689	SRSF5
SOGA1	ASB2
PTTG3P	BOP1
MCM3AP-AS1	CYP20A1
MIR1180	DCAF11
MIR487A	DNAJB5
C1QTNF1-AS1	EZH1
MIR105-1	GGCT
MIR5692A2	HECTD1
MRPS11	MAPK4
CUL4B	NMI
MGA	NOVA1
PLCD4	RPS11
LOC110386951	TMEM106C
CCNDBP1	ZMYM2
ORM1	CARM1
TRIP12	CES1G
PRR5	CRYZL1
TEC	DYNLT1
DGCR8	E2F6
SHKBP1	FBXL3
NOMO1	HSD3B6
NOMO3	MAN1A
INPP5D	MRPL47
BLMH	POLR1F
NUCKS1	PWP2
UVRAG	RPP40
BUB3	RPS6KC1
RPL24	SERPINA11
ZNF207	TNIP2
SULT2B1	TSPAN14
CDC16	ZFP62
MEFV	ACAD11
GPX7	ADGRG2
PSMD5	COX6B1
LOC108961161	GALNT3
RAP2B	KANK4
FABP2	KLF7
CIITA	MPG
H2BC13	OGFRL1
NEK8	RAB32
BRF1	RNASEH2A
MBP	RPS8
MSRB3	WNK1
MAS1	AKAP13
CSNK1E	CYB5B
GCSH	PLCE1
GPC5	SH3KBP1
RPL11	SLCO4A1
TRIM59	VMP1
FBP2	CS
TYRO3	AP3M2

DNAH2	ATP5PO
IL1RL1	CDADC1
INTS6P1	CHP1
MAPKAP1	CLCN6
NCKAP1	CYP2C
GHSR	EPB41L4A
HSPA12A	GPAT4
ACE2	GSAP
ULBP1	MAP4K5
ADH5	MFNG
EIF3L	NIBAN2
CDSN	NKX2-1
TEFM	P3H3
NAB1	PDCD10
SAMM50	PIP5K1B
VEPH1	RNF138
OSMR-AS1	TMEM14C
LINC01265	BACH1
MIR3166	FOXS1
HLA-DPA2	RAB4B
LOC102723899	TRMT2A
ENSG00000255471	AK3
RNU6-959P	RDX
RNU6-828P	YARS1
ENSG00000223969	C3AR1
ENSG00000173867	COBLL1
ENSG00000249631	COL17A1
RN7SL731P	FAR1
ENSG00000235292	FEZ2
LOC107985896	LHFPL2
RNU6-37P	OAS3
ENSG00000250131	PGM5
LOC100420680	RPRM
RPS20P10	SH3D19
ENSG00000248262	ST6GALNAC2
RF00026-601	TAF9B
RF00017-6427	TMC5
ENSG00000285588	TRERF1
lnc-RNF39-5	TSPAN33
lnc-RNF39-8	EFHD1
RF00017-4589	POLE
piR-48222-068	COL4A6
lnc-FOXN3-6	DAO
NONHSAG009279.2	DNAJA2
lnc-PGD-4	PPP1R14B
ENSG00000280339	ZMIZ1
lnc-TDP1-2	APOL3
lnc-CCNL1-7	FTSJ3
piR-41141-006	PITRM1
HSALNG0015894	PLCL1
piR-56759-534	SNX1
piR-61240-151	ANK
piR-38351-255	ANKRD37
LOC107984428	DPYSL3
lnc-BRD2-5	PRDM1
RF00017-4590	RARG
lnc-FOXN3-7	RIOK3
piR-58308-062	TNIP1

piR-37045	TTN
piR-36588-346	AGFG1
NONHSAG046509.2	AMOT
lnc-PNPLA5-2	CARMIL1
RF00017-054	CDC37
LOC105376725	CNMD
SEPTIN7	CUTC
MPZL2	EML1
KARS1	ETFRF1
EPHA8	HLX
SENP3	KLHL7
LOXL1	LCORL
NOP53	LDB2
MPZL1	MYEF2
ORAI1	PDCD2
EGR3	PLEKHH1
VDAC3	PMS2
PBX3	RND2
POLG2	RSRC1
EIF4A2	SLC8B1
CHD3	TBC1D31
UBE2J2	TCEA1
HERC4	TULP4
PPAT	ANK1
SNRPG	CALML4
ARHGAP42	CSNK1E
SLC2A4RG	FARP1
TMEM99	GALNT1
LRCOL1	GNG7
PROX1-AS1	GPSM2
LINC02484	HPN
LOC101928858	IL2RG
MIR519A1	KCNB1
WLS	NDUFV2
MAZ	PDE1A
HSD11B1	PLEKHB1
PSMC6	THBS4
TNIK	TMEM158
POTEE	CAV3
SSBP3	DDHD1
ARRB2	EMILIN2
HRH3	EXOSC5
RPGRIP1L	RPIA
UQCRC2	SCN3B
TSPAN4	SNTB1
CYP2R1	SSB
TUBA4A	TSC22D2
PPBP	VEPH1
ATXN7	IL2RA
H2BC5	MAPK10
H2BC17	ERCC6
FMR1	PGM1
CASP4	ACOT5
EPB41	LRRC47
PPIB	STX4
DENND2D	TMEM11
COPA	VBP1
MIR1247	AFF4

ERAP1	POLA2
CYYR1	ST14
ARAP1	VSNL1
ICOS	LAMB1
CYTH3	RETN
SLC25A6	ARL6IP5
OPRD1	COQ10B
PRPF3	CSNK2A2
MT-TS1	EPB41L5
CYB5R3	NPR2
TMEM98	PDP1
RASAL1	TET2
ID3	UAP1
HAT1	SLC12A2
DDX6	ABCB7
AZU1	COQ10A
EIF3E	DNAI1
NR2E3	EFR3B
TNFSF12	ENTPD3
HOOK1	KIFC2
CIAPIN1	KRT6A
CDC123	MNAT1
SEPTIN11	NAP1L4
CD3D	NEDD1
PAQR3	NGLY1
TSN	NUBP1
CAVIN1	PNMA2
UGT1A10	SCYL1
MT4	SLC66A1
PCLO	SURF6
ASPM	ACP3
UBA52	AP1B1
TFDP2	CHCHD6
KNL1	DCUN1D4
ZMYM2	DPP3
ARL5A	FRMD4B
C19orf48	GYPC
BCAP31	MASP2
GALNT10	MLF1
TK2	PLOD1
MIR18B	RGS19
UNC5B	RNF19A
LPIN1	TTC39A
ACVR1C	APPBP2
FBXO8	ATRNL1
BDKRB2	GAK
INA	KIFAP3
RPS6KA2	MYH2
UHRF2	OS9
TRPV2	OSTC
DDX17	PLA2G1B
LOC110467515	RAB11B
BBS2	RAP2B
PARVA	RLIM
LINC00328	SGCB
RPL3	SNN
CAP2	SPEN
USP16	TMEM135

NAV3	TRMT61A
SIRT4	WDR3
LMO4	WWC2
MAST4	ALDH1A3
TENT5C	EPHA2
RPL13AP20	ARNT2
DHRS4	CACNA1A
CHMP4B	MAPK11
SRSF7	PFKL
PRKAB2	SLC7A14
PTCD1	ZFP52
NPY1R	AOC3
CDHR2	ASF1A
H2AZ1	AUH
CARM1	FLCN
VPS4A	FOXP3
MIR3619	HGD
SH3KBP1	LDHD
TRIP6	NCAPD2
CDK20	PIM3
CPT1A	PPT1
MYH14	TFE3
PGLYRP2	ZFAND5
APOC1P1	NCOA3
TMOD3	ARAF
TRIM3	BMPR1A
LY9	DYRK1A
CD3G	GBP4
AP2M1	IKZF1
VASP	KCNE3
CBLL1	KIF3A
MATN2	MAP3K12
NECTIN2	MYH14
IFNGR1	PABPN1
CDT1	PBXIP1
M6PR	SLTM
SNTA1	STIL
MSRA	TECR
RFC4	UQCRB
NAA11	ADAM8
NOLC1	FLNC
SRSF5	FTCD
PPL	GAA
PTCD3	PRKAR2B
SLC28A1	RARRES2
CFI	CHCHD1
EEF1B2	EMC8
PSME1	MBL1
FHL1	NRD1
LRP10	NSMCE2
ZNF274	TARS
DELE1	TEX9
NFYA	ACBD5
NHEJ1	ADH1C
HOXD9	ARMCX2
MIR466	CELSR1
HNRNPR	CEP85
ZNF23	LRP12

SLC22A5	NIT2
SLC22A7	NLRC5
PBXIP1	NNAT
BIK	PBX2
CIDEC	PJA2
TMEM88	SERINC1
UBE2D2	SLC22A15
MIA3	TRAK1
PGAM5	UPF1
RASGRF1	AGMO
SLC7A2	ANAPC11
JARID2	BICD2
POU3F1	CCL21
MIR642A	CFDP1
CDK11A	KIF4
BRCC3	MRPS33
GALNT2	MTREX
P2RY11	NME6
GSC	STK24
LAMB2	VARS2
NUP88	FSHR
ICMT	TARDBP
MIR3928	ALDH8A1
RAPGEF1	ATP2A3
PEPD	CENPI
CLVS1	CNN2
CFB	CPNE8
CUBN	EFNB1
EOMES	GRK3
CFAP20	MOXD1
H3C15	PRDM16
MAPK13	RPL22
NXF1	SELENOW
AP2B1	SFTPB
PTTG1IP	TNNC2
SPINK6	LIMCH1
SCLY	C4BPB
FBXL15	CBX3
CA3	CCDC86
ATF7IP	CCR3
BVES	CYP3A9
RRAD	DCAKD
HSD17B2	FNBP1L
CDYL	GMPPB
GPATCH2	HOXB9
FCN2	KTN1
SLCO1A2	NDUFA7
SSX4	NSG1
MBD1	P2RY6
GTF2H2	PLD3
KIF3B	PSME2
GRAP2	RPL14
ATG16L1	S100A1
IFN1@	SEPTIN11
UGT1A4	SLC25A15
DUSP14	TNFRSF14
RTKN2	UROD
MIR770	USP5

RNASE4	ARRB1
TNFSF14	HHEX
HES5	MYLIP
TUBB4A	ADORA1
PHF2	GH1
PDK4	LHCGR
KIF23	ACYP1
MYL2	AHCTF1
COA5	APMAP
GNA13	APPL1
HMGB2	ARHGEF19
AFAP1L2	CALN1
DLX2	DAGLB
TAMALIN	DGKH
SHROOM3	FAM3C
ASH1L	FCGR3A
PSMD14	FCMR
CDKAL1	FGFRL1
GJC1	IRF2BPL
MAP4K2	NLE1
PSMC2	P2RX5
HLA-S	PLEKHF2
TNRC6A	PPIL1
A1CF	RAB25
IGHG1	RRP9
TM4SF5	SLC25A24
TFPT	SNCB
HMGCS2	TRIM35
IL17RB	ATIC
GORASP1	DPP7
PPM1B	ERCC5
ANOS1	FLRT2
CEP135	GFPT2
IPO4	PPP1R9A
IPO9	RGL1
UBE2N	SLC3A1
KDM4B	STBD1
HAPLN1	MYH7
ACTN3	ANKS4B
HIVEP2	CCN3
DDX47	EIF1A
PARP12	GBA2
SAMSN1	IPMK
PUSL1	KRT79
IL34	LRMP
FAM160B2	MFSD1
ATPAF2	NEFL
OIP5	NUDT6
CREM	ORMDL2
MAOB	OSBPL10
TBXAS1	OSBPL7
CDK11B	RTN2
CABIN1	TMEM63A
KDM8	UGT3A1
RFC3	UNC5CL
GIPR	ZBTB7A
SLC7A11	CCDC120
MIR645	MTERF3

TSPYL2	QTRT2
EIF1	NPY
MAD2L1BP	ACIN1
CIRBP	ARPC2
S100A16	CEP57
CNPY2	CKLF
CAVIN2	CREB3L2
MIR758	HSD3B7
YRDC	MMUT
SERPINA11	MN1
CALU	PLBD1
SETD1A	PLCD1
LGMN	RPL9
FUT5	TMCC1
H2BC3	ABLIM3
PYM1	ADARB1
ULK2	ADCY9
INTS4	ARL6IP1
TRIM16	COX7C
MIR153-1	GAL
PKD2	LRP4
GMPR2	LURAP1L
LIPA	MYO10
LOC112081413	NFE2L3
IMPA1	STRA6
CLIC5	YPEL2
GNPDA1	ZFHX4
MAFF	ITGB5
DNAH5	NSDHL
SGSM3	FGF9
FMN1	ASCC3
GIPC2	BCAP29
SIGLEC10	CDK2AP2
UCKL1	CEBPZ
TRPC4AP	CSNK1G1
CBX6	FCHSD2
BTG4	MARCHF6
GOLGA3	MFHAS1
CDYL2	PIF1
SAFB2	PRPF40A
KCNRG	PXK
THOC5	RBBP7
ZBTB12	RDH5
CEP131	SRGAP3
IER2	SRPK1
HSF2BP	ZFYVE26
SPATA9	AP1G1
UBAP2	CCDC92
LCTL	COX7A1
ZNF233	DNM1
ZNF26	EEA1
NXPE3	EFEMP2
YJEFN3	INF2
CCDC178	LYPD1
MAP11	PASK
ZNRD2	PSMC1
H2BC18	RASSF2
INSYN2B	RPN2

LMCD1-AS1	UPF3B
TMEM92-AS1	ARF5
ID2-AS1	ATG2B
EPS15	CDKL2
AGXT	CENPX
LOC112679202	CLTA
DIAPH3	CPED1
TXLNA	DUSP13
CKM	EIF3F
SPDYA	ESRP2
IKZF1	FBXL7
OPRK1	FES
RPN1	GTPBP1
DDX46	HSPBAP1
MLN	LUC7L2
ZBTB17	NUDT12
DGKQ	ONECUT2
ARPP19	PIGQ
MIR1275	PIP4P2
MIR329-1	POGLUT2
TNFRSF4	PPP1R13B
SMAD5	RRN3
MIR520F	TBCEL
MPP3	TPRKB
RNF111	WIPF3
EEF1D	RXRB
ACACB	COL5A3
SIVA1	GFPT1
NINJ1	INHA
IRF8	MXD4
FBXO7	SLC39A4
SLC7A1	CDH3
ZNF32	ESCO2
STRN	CASKIN2
IARS2	CELA2A
TNR	CYP2C70
OXCT1	DAPL1
PFKL	DCAF5
FOXO6	DCLK3
ZFYVE9	GGA3
RRBP1	LONRF2
PLAA	MAK16
PMPCA	PTPN23
MEIS2	RNASEH1
SERPINE2	RRP1
NEDD4L	APOL7A
KIR2DS2	CD8B1
ST3GAL5	LTB4DH
C7	RBM13
NEIL3	REN1
ZHX1	CANX
MIR610	ACSM2
DBI	ADPGK
GIT2	AKR1C6
TRIO	CACNA2D2
TXN2	CES1E
DNAJA1	CLEC4F
GRAP	CNKSR3

CDC14B	HIGD2A
NLRX1	IL12RB1
TLN2	KLHL4
XPOT	LCP2
DLG5	LRP10
SLU7	ME2
UNC5C	MORF4L2
C1QBP	NIP7
ITPR1	PIAS3
H3C14	PRPSAP1
CIAO2A	PRR13
SLC1A2	SCAMP5
MAVS	SEPHS1
CABYR	SPI1
JTB	UBAP2L
SNORA24	UBE2J2
MIR761	DLG4
FSTL3	CNR1
RAB21	ACSM3
HSD17B10	CASP8AP2
SRPX2	CGNL1
GALNT4	IRX3
CKAP2	KRT13
CHAF1B	LTC4S
MXD1	MGAT2
NDUFA9	NUMA1
SGK2	PPP1R14A
KPNA1	PSMD8
PSMD3	SLC7A6
TIAM2	SMAD9
DRAM1	TBC1D1
H2BC12	TNNT1
MIR520E	TNNT2
UBTF	CD200
ZNF667	NEU1
COP1	PSMC6
PAFAH1B1	SLC25A1
TARDBP	TFAP2C
NAE1	ABCG4
MIR208A	ARL15
MIR604	CDC5L
MIR593	CILP
RBPMS	CLIC6
DGAT2	CMTM7
PPP1R8	CTNNBIP1
OSBPL1A	CYFIP1
LOC111216273	DEF6
FLAD1	FKBP7
USP5	GDF11
SLC25A3	HYKK
DGKA	LCMT1
ITPKA	OBP3
RCN2	OPN3
SSTR5-AS1	PAFAH1B2
SSR3	REEP4
FBXL5	SEC61G
DCAF13	SLC25A51
SSX4B	SNTA1

SSX7	STX8
TRIB1	TTC27
RASA2	UBE2A
PRKRA	CSNK2A1
TARBP1	FOLR1
F11R	PPP1R12A
NR2F1	PTGER3
CAST	SHMT2
C4A	CLCN3
TPP1	GNB1
RAB34	H2AC6
LOC110599567	NR2F1
NDUFA10	AFF1
PARP2	CLDN14
LITAF	CLP1
UTRN	COPE
ATP1A3	CREBZF
DHCR7	DUSP9
EYA1	IPO7
HTR1D	PCDH1
RPS10	PSMD2
PAPSS1	RAB11A
DYNC1I1	RNASEH2B
ATAD3A	SH3BP2
BTLA	SRRT
CKMT1B	TLK2
HMCN1	CD93
CCAR1	DICER1
EPB41L5	FOXP2
SPINK13	RABGGTB
MIR519C	ADCY2
MIR646	ATXN3
CD244	BCOR
TOMM34	GTF2IRD1
PDE4A	HACD4
NDUFA12	LHX2
TRIM32	PPP2R1B
INHBB	PTPN13
LILRB2	RBM5
GCLM	RUVBL2
CCL23	SRR
TMX3	TIFA
DSEL	ZAP70
NMI	CCDC47
CD209	CEP135
PDE4DIP	COG8
KCNN3	CYP3A44
TRAK1	DUS2
CTSZ	HAUS6
RNF20	LAMTOR5
MAP3K12	MSI1
PTPN2	OXNAD1
TMPO	PACC1
TP53AIP1	SPATA2L
MIR325	CALM2
ARHGAP9	PDE2A
XPO4	UCP3
GUCD1	ARMC6

LSS	MRPL53
FBXW5	TM4SF19
CHD6	TMEM9B
PARP14	USP20
SPART	ASB9
DCX	BLOC1S2
GPAA1	CCDC90B
PRPF31	CR1L
FARSA	DLL4
MYH1	FAM20A
CMBL	HAUS1
RPP30	HBS1L
ACOT12	HSPB9
IGDCC4	LMOD1
ONECUT2	MYO1F
PASD1	NUDT21
ODR4	POLE4
SPINDOC	RBM17
LINC00461	RCHY1
MIR602	SGTB
PROC	SLC5A9
LINC01615	TBCE
DHRS2	TMEM44
GNAI3	C2
SRPRB	KISS1
DESI2	PPBP
SHCBP1	RPL27A
UTP14A	UPB1
UGT2B4	ZDHHC2
LNPEP	AMOTL2
CTAG1A	CDCP1
EIF4G2	DNER
NR2F6	DSCC1
VSIG4	DSG2
SPC24	H2AZ2
DARS2	MTA1
S1PR2	PPP2R5C
BDH1	SLC12A7
RPS26	SOX11
ACSM5	ACBD4
ANAPC13	KDM5A
ABHD3	KLC1
APOC4	MOGS
CDNF	MRPL50
GOLGA7	MTFR1
MS4A7	MXRA8
UBL5	NADK2
RAB24	PPP2R5E
SLC22A25	RALGPS2
MED8	RASL11A
C12orf66	SELENOS
CDADC1	SGPL1
CCDC158	SLC2A6
C2orf72	SLC39A1
CFAP221	SP100
C3P1	SYTL4
REELD1	TMEM106A
LOC100288798	DZIP1L

TCEAL3-AS1	LTA4H
LOC101927269	AOC1
PCCA-DT	CD300LF
LINC01595	CDK20
LINC01348	CLEC4A3
ACTBL2	GNGT2
C12orf43	H2-EB1
IDO2	HEPH
PLOD1	MBOAT2
LIN7A	MDM4
CASC3	MRPL30
HRG	PARN
SLC1A4	SART3
IL31	SEC22A
GINS3	TMEM25
SRCAP	UCK1
SYF2	UCK2
SERPING1	UPK1A
SNAP23	USP4
CPA6	ANXA11
TRIM22	APBB2
PSMD11	C7
ADCYAP1R1	CELF1
TUBB6	EPS8L2
UBE4A	EXOSC2
CHCHD2	FGF10
PRIM1	GALNT10
MALT1	GPRC5C
HDAC7	OMD
ITLN1	PCYOX1
PRPF4B	PDP2
SYTL1	SEMA3E
DPP3	SLC22A12
PEX2	AGMAT
IL10RA	CENPP
GPR37	FBXO22
CRY2	GAPVD1
SYVN1	HERC1
TPTEP1	HMOX2
MIR548L	HOOK3
GINS1	KSR1
LAPTM5	MYRIP
SUMF2	PHF5A
NAXE	RAB5B
IL26	SDSL
SLC30A8	SIL1
RFX6	SLC37A2
ARF6	THSD7A
MIR509-3	TSPYL2
ITGB7	ZFP354A
ACVR2B	AEBP1
AS3MT	BCL2L13
RPS3	CAMTA1
AIMP1	CDH4
CDK5R2	CENPN
CHST4	CRKL
RSU1	CXCL6
PRMT6	DUOX2

PCBP4	FNBP4
TAB2	GTF2B
MEOX2	KAT14
H2BC1	KCTD1
LAS1L	KIF5B
ABT1	LARP1
LEFTY2	LDLRAD4
ROBO3	MAS1
PSMB2	MDC1
TRAF7	MUC4
HNRNPH3	NEIL1
KRT80	NPHP4
MAGEH1	PRMT2
LCT	PWWP3A
ADRA2C	RAB4A
LBP	SLC16A4
CYP3A43	TANC1
ATG14	TCERG1
ANXA8L1	TMX1
MARVELD1	TRAFD1
RPS19BP1	TRIM27
ADRM1	TRIM6
HOXD8	ZFYVE16
IL25	TRH
DIXDC1	P2RX7
MIR760	PTGER4
MIR1260B	ANK2
ANGPTL3	BCL11A
HERC1	CEMIP
C6	DEFB1
ACOT8	HLF
IRGM	MLKL
OSBPL11	PDCD6IP
ACRBP	PPM1B
NABP2	C2CD3
LARP4	CYP2C50
LEPROT	LRWD1
SLC25A48	NUDT13
LOC111216272	PARL
CEP164	PXN
ATP5ME	ACTN4
XAB2	ADGRV1
MDH1	ADORA3
GALNT1	EXT1
HRNR	F12
TTC28	GLIPR2
GAD1	MECOM
NDUFB8	PICALM
SLIRP	PSD3
ZNF267	STMN2
NDUFB3	CDH5
SERPINB9	BMP8B
DSN1	CAPN15
PTPN18	CDR2
TASP1	CHAC2
BOP1	COG3
ISG20	CUL1
PTBP3	CYP21A1

NT5C2	DNAJC1
JMJD6	HPS5
FOXL1	INPP5A
MIR3662	KLF1
PDHB	MAX
CPXM2	MIDN
CILK1	MORC4
TCN2	MYL4
KIR3DL3	NIT1
KCTD10	NRBF2
MIR4458	PRKX
ENPP7P13	RAB23
TBX21	RABEP2
ACAD9	RBM8A
C12orf65	RNMT
COA6	RTEL1
ENO3	SBNO1
AQP7	SPIB
RPL35A	ST8SIA3
USP11	TAB3
FBXW2	TAF5L
THADA	TM9SF2
GLRX3	TMEM150A
ETFB	VRK1
LOC110806306	AP3M1
AGPS	C5AR1
RPS6KA5	CABIN1
IL18BP	CDC42EP5
H6PD	CKMT1
APOA2	CNTFR
DYRK2	CORIN
SMPD2	FDX1
MGP	FUCA2
SSX2IP	GALT
AK3	HGS
CLN3	HIF1AN
GLCE	HNRNPC
B3GNT2	ITGA9
DNAJB11	MAGI3
KANK2	NAT8L
PLXDC1	NDUFB4
SUPT6H	OAS1A
ERGIC3	PCNT
KANK3	PTRH2
PATJ	RASSF6
LSINCT5	RSF1
DPYSL3	SERPINI1
AGBL2	SLC38A1
MZB1	SPP2
CCDC85B	SUGP2
TMEM234	TBC1D9
EGFL6	TDRD3
LOC111255642	TLE5
FGG	TTC23
MAP3K21	WDR47
SEMA3B-AS1	ZDHHC14
SUCLG1	GDNF
MRPS22	CTSG

NEUROG3	RDH9
COQ8A	ABCB6
SPARCL1	GNAI1
KMT2A	SLC39A10
KIF13A	AADAC
BMPR1B	AKT1S1
GNB1	CBX4
SLC2A9	CLIP4
SGPL1	DAPK3
PROK2	HPGDS
MED23	MND1
CSRP1	NME4
LAIR1	NRXN3
NCAPH	NTN4
TNPO1	PMS1
DLX6	RPL18
LMO3	SLC25A23
DDX24	ZCCHC7
POF1B	ZFR
MLF2	ADH5
RAD51AP1	DAG1
LUZP1	ITGA2B
VPS13D	PLIN3
ZG16	SYNM
DYNC2I2	AP2B1
H2BC11	COX2
MIR3194	CSGALNACT1
RAET1G	ELMO1
GFI1	FA2H
GABRB3	HOXA5
LHX3	ITPK1
MARCO	KIF1B
RNF41	LARP7
AQP4	PAK3
LUM	PLCD4
NPEPPS	RPL36A
NOM1	RPN1
DIS3L2	RRAGC
GEM	SIDT2
GLIPR2	SKP1
CCDC40	TERF1
TMPRSS13	USP1
EAF2	HSP110
TXNDC9	TEGT
CROCC	CTDP1
C1orf43	GTF2F2
GPR78	KLHL25
TMUB1	MOB3A
GLYATL2	PIGT
FAM9C	SCAMP4
LINC01287	SGSM1
PHACTR2-AS1	SPRR2A
LINC01374	TRABD
MIR2392	WDR81
MIR892A	ZNRF2
UC.134	UGT1A7
LDB1	ACYP2
ARNTL	AGA

ATP5F1E	ARMC9
DDX20	BAG6
MRPL44	BCL7A
NENF	CACHD1
ANXA11	CD2BP2
NELFB	CDK5RAP3
UTP4	DNTTIP2
CCL28	ESAM
SPTAN1	FAM110B
FBN2	GAL3ST1
KCNH2	HIPK1
GCN1	HPS1
EFNA4	HYI
HTR2B	JPH3
EIF2S3	MRPL38
GABARAPL1	NOD1
NCAN	NUDT5
SLC38A2	PCMTD2
ARHGEF19	QSER1
NOSTRIN	RT1-S3
CCDC8	SAMM50
YTHDF1	SEMA5B
ZIC5	STX12
GINS4	TBRG4
FSIP1	TMX4
PARPBP	TSPAN1
CCNJ	UGGT2
RNF187	AAMP
H2BC9	ADCK2
PLPPR5	ARHGAP25
LINC00467	CNOT1
MIR520G	ERH
MIR575	MPPE1
MIR877	MRPL16
MIR922	PRPF18
MIR769	PUM1
MIR920	ST6GALNAC3
RAD51-AS1	STXBP5
MIR329-2	TMEM41B
MIR194-2HG	ZYG11B
MIR924	ARAP2
MIR4443	DDX39B
MIR4782	FANCG
MIR4262	HNRNPL
LOC106014249	MAG
POLA1	MUC2
NDUFS3	TUFM
INSIG2	KCNK1
FLII	APOL9B
PCSK7	ACBD3
LOC106096416	BDKRB2
CYC1	CAMP
HEXA	FBXO3
AASS	IGTP
ART3	KDM7A
TNIP2	MKNK1
LIN9	NDUFA3
FOXJ1	PAIP1

SERPINB12	PARM1
RTP3	PGP
RASSF7	POLI
ARL4C	PTBP3
PPT1	RNF103
DUSP28	ST3GAL3
NFE2L1	YES1
KCNJ3	PNN
ADRB1	REST
CNTNAP2	TRIM59
LOC111365141	CDC7
GGA3	CX3CR1
B4GALNT1	FBXO34
AMPD2	KDM3A
GRHPR	MAL2
OPA1	PLN
OLR1	SPRR2D
DDAH1	ATG10
CYB5B	BOC
IL21R	CCNO
GSTK1	CD2AP
HLX	CIAPIN1
BZW2	DDX56
STK26	DPAGT1
P3H1	IFI16
BYSL	IK
PLXNA3	LACC1
TMX1	MAP4K2
TXNDC12	PDRG1
ABHD10	PRRX2
MYO18A	PYROXD2
PROKR1	RGS7
IL9R	RHOD
STX2	RRP1B
ZC3HAV1	SERPINA3M
ARHGAP44	STRN3
MICALL2	TMEM123
DUOXA1	ZDHHC21
SYPL1	ZPR1
KIF27	SLC7A1
COA7	ARHGEF5
SGTB	CCDC3
SPIDR	CDIP1
MIR374B	CYP4A8
MIR601	DMD
KTN1-AS1	EYA3
MIR767	FBXO8
MIR522	FECH
MIR4516	GRID1
MIR591	MIR30E
LOC117204001	POLR3B
ACADS	PRELID2
SLC5A3	RTCA
PEX6	RUNDC3A
G6PC2	SMYD5
DDRGK1	SNIP1
MRPL28	SPIRE2
ENPP1	STX17

BBS9	UNC119
PRPF40A	WAS
INHBC	ANKRD13B
ADK	APBA3
PLOD3	EXTL3
ERVMER34-1	FBF1
NPPB	NVL
POLL	ARIH1
CAPN10	ATG3
NPTX2	B3GNT5
NDUFV2	CENPH
NDUFA4	CYTIP
COQ9	EPHB6
TRIP10	F11
RANBP1	FIGN
LHX6	GAS2L3
LOC105274310	HACD3
C1QA	MFAP5
RPS14	MPZ
IL3RA	NAB1
EXOSC4	NME3
AMPD1	PLLP
CIT	SOWAHC
GMDS	SRD5A3
BAAT	TSPAN12
CNDP1	YRDC
CLDN14	MAP3K7
DGKG	ARHGAP29
KMO	ATP6V1B2
RHOT1	CUL3
CRY1	FARSB
NR2E1	GTF2I
SPTA1	HSPB6
PSMD7	PNPLA8
RAPGEF4	SEL1L
ARHGAP4	SLC39A6
MGAT4B	UBE2B
HPCAL1	VAMP2
RFX5	ZC3H12A
CYTH2	ABHD11
NCS1	ART4
PEMT	DNA2
PSMD12	ECHDC3
KCNE2	GP1BB
PSMB3	HERC3
B3GALNT2	KLHL2
RPL30	MIR29C
SCD5	MLLT10
SLC35C1	MPC2
SLAMF6	NKD2
TPPP	RCAN3
RAB31	RUFY2
STXBP3	SEC11C
FXYD6	SEC24B
ART4	SH3BGRL2
CRLS1	SLC35D2
CD48	SLMAP
CHST12	VASH1

CFHR3	VOPP1
DNER	ZDHHC13
IGSF3	ANO2
PPP1R12B	IDO1
RPL36	NFATC2
OPN3	TP73
TPTE	ACVR1
STAB2	BAZ2B
TUFT1	BLVRA
LMCD1	CHD7
BMS1	FAM126B
SH3RF1	HTRA3
NFE2L3	LUC7L
TOR1B	NRN1
TOB2	PCBP2
UGT2B28	PIK3AP1
UNC5A	PTPN14
ZNF24	SAMD9
VAT1	THOC2
AKR7A3	WASL
GABRQ	ZMYM3
APOBEC3F	GAS2
FAM83H	HSPG2
BUD31	RAB20
GUF1	SERPINB8
COG6	TCF3
EHD3	TIAM1
PEF1	TUFT1
OXGR1	ARPC5
SCPEP1	ARSJ
TRIM55	ATP6V1F
PTPMT1	BTAF1
NCAPG2	CUL4B
GSPT2	DYM
SF3A3	GOLGB1
PBLD	HDAC11
KHDRBS2	HMBOX1
MANEA	KLC4
LRRC1	LRRC58
CMTM6	MAP4K3
CHST13	MRPL9
IGSF11	NAPA
TONSL	NUDT19
KIZ	PDE1B
TMEM9	PKN1
PRDM8	PRR16
DNAH17	RASL12
FKBP11	SAMD8
ADAM21	SUFU
DNAH10	TERF2
CCDC47	WDR19
GPR155	NPHS1
DBX2	SLC4A1
SARS1	ARL6
SPRYD4	DENN2B
TMED3	FAM47E
PCDH11X	GCN1
WWC2	SPICE1

F8A1	TMIE
ANP32E	ZW10
EPPK1	CHI3L1
CDKN2AIP	ALG2
DENND5B	BCCIP
CEP85	CERS6
SPAG7	DAPP1
RRS1	FASTK
NLRC3	HDDC3
UBQLN3	HECA
TRMT6	IPO9
SYAP1	ITM2C
KLHL21	NDUFA8
CMTM2	NUBPL
CMYA5	POLR1C
DNAJC14	PPIG
RSBN1L	RFWD3
SLC44A5	SIRT5
TRIM60	SLC17A2
TRIM7	SLC39A5
SVOP	STK38L
TMEM100	TFF2
IFI44L	TIMM23
KRT26	TMEM144
VNN3	TMEM64
CCDC174	WFDC1
FBXO34	FBLIM1
FBXO36	KIF5C
CLDN17	MICAL2
EFCAB2	PDE3B
ICE2	PHLDB2
SAC3D1	PNISR
L3MBTL4	PON3
KRI1	TBK1
ZNF479	TENT5A
RMDN1	TRPV6
DUSP21	UBB
PRMT9	WDR1
ZC3H12B	ABHD3
ZNF717	CHD3
DTHD1	DLST
SH3D21	INPP1
FAM43B	LRPAP1
SVBP	MSI2
MFSD4A	MTCH2
PCDH11Y	NPHS2
BBOF1	SEMA6A
RMC1	SLC43A2
GRAMD2A	TMEFF2
OR2T29	TMEM171
OR2T5	TOR1A
MIR1224	CYP3A5
MIR1306	COL11A1
HOXA-AS3	PAX6
LINC00161	PSEN1
MIR1296	PRKN
MIR663B	NFIC
MIR636	ABCA7

EIF2S3B	AKR1B3
LINCMD1	ATF6B
MIR3117	DCAF13
FAM99B	FIG4
MIR632	FRAT1
DUX4L1	GOLGA8A
LINC00462	HINT2
LINC00488	HOXB6
LINC00707	IL18RAP
MIR1231	IL3RA
FLVCR1-DT	KLHDC2
EEF1A1P5	LTV1
MIR3178	METTL5
MIR512-2	MUC13
MIR513C	MYL12B
LINC01080	OSBPL11
MIR1181	PGAM5
MIR376A2	POLR2G
MIR3126	POLR2H
SNORD76	POMP
SNORD113-1	PQLC3
LINC00682	SCAND1
MIR1199	SYMPK
MIR3131	TMCO1
MIR764	TRIM11
LINC01391	TSPAN17
MIR1268A	SLC9A1
MIR5581	HIBADH
MIR3910-1	PDIA5
RB1-DT	UBQLN1
EIF3J-DT	MT1X
MIR3910-2	AGO4
MIR103B2	CALCRL
MIR4641	CDC42BPA
LINC01703	CMTM8
MIR6875	DMAC2L
MIR103B1	FBXW7
KRT8P3	GALNT2
MIR5692A1	GLRX3
UBE2CP3	H2BC12
MIR1843	HBA-A1
MIR9986	IGHM
MIR548BC	JPH1
LOC105369748	L3MBTL3
HVBS8	LIG4
MIR9985	LRRC20
LOC110599568	MAP1A
DPY30	MAPK8IP1
BCHE	MICALL1
KIR2DL3	MMACHC
RSL1D1	MRPL19
ACSL3	NFASC
ITGA7	NRARP
NDUFS8	P2RX2
GALNS	PCDH9
MT1E	PHF14
IMPDH2	PLEKHG5
MNX1	PRAG1

GDF9	RBFOX2
NARS2	RHOJ
PDSS1	SLC25A36
GALK2	SLC26A2
COQ2	SLC4A11
NBAS	SNRPG
WDR83	THOP1
FAM167A	THPO
FAM124B	TIMM13
TANC1	TNXB
DENND4B	TRIM13
PLUT	TUBG1
TDG	UBL3
INPP4B	WNT2B
IWS1	WNT5B
SPG7	ADAMDEC1
OSBPL8	AMY1
BNIPL	CEP192
SLC12A6	CMAH
NEIL2	DGUOK
ATP1B1	FUT7
ADH1A	H2-K1
ABCE1	MAST3
ETFA	OCRL
CTNNBL1	PLXNA1
DERL1	PSD4
RASA4	SEC11A
TP53I11	SERPINA9
EPM2A	SPG21
DR1	TAF1A
PRDM4	ZSCAN12
FAAP20	ATXN1
NECTIN1	DEK
SOX9-AS1	SLC16A2
MSC-AS1	SYNE2
SYTL2	CYP1A
CCNF	HBB-B2
WFDC21P	HSD3B4
TKTL2	CHSY3
BMP8A	FBXO42
FMNL3	UPK3A
CANT1	KLK1
PDE3B	PRKAG2
APOC1	BCAP31
BMP3	BTBD7
CLIP2	EED
ADNP	ERLIN2
HNRNPUL1	HOOK1
ABCG1	KMT5A
NDUFA1	LIN37
MDH1B	LZTS3
CLTRN	MAGT1
NDUFAF4	MARCHF5
TIMMDC1	MRPL23
RANGAP1	NCKAP1L
HSD17B12	NEDD8
REPIN1	NINJ2
A2M	OAZ1

SDF4	PCDH10
SEC14L2	PDCL3
TNFSF13B	PKIG
IL1RAP	PTPA
RBM14	PTPN9
CHN1	RABAC1
GRID2	STOML2
SERAC1	TEX2
REL	TNKS1BP1
COL15A1	TRIM37
U2AF2	VPS37A
USP25	AP2M1
CACUL1	BACH2
KLRG1	BCKDK
UQCRC1	COX15
PCSK6	CUL4A
SRI	EP400
TMEM165	IFI47
MIR1299	LRBA
MARVELD2	SEMA7A
UBE2L6	SIX1
NUP98	SNRK
MNT	TINF2
MOB2	TRPS1
IL2RG	UCHL3
DAPK2	CAMK2B
LOC110366354	BUD31
ARHGEF10L	CCDC34
VLDLR	COL11A2
HADHB	CREB3L1
ATP5MD	CTDSP2
INPP4A	EPHB4
EIF3B	GABRP
JADE3	GALNT11
IPO5	GNG5
ITIH5	HEBP1
LOC111162621	HOGA1
DPT	KIF2A
DDX41	MAN1A1
NUBPL	MICB
TOM1L2	MRPL4
BCCIP	MYCBP
MIR935	NANOS1
DIAPH1	NDUFC2
MOGS	OSBP2
COPB2	SCAF11
FOXL2	SMYD2
LIMA1	STMN3
GAD2	STX2
MYL9	TBC1D5
LTA4H	TRIAP1
SIN3A	TRIM47
RAET1E	ZC3H13
RNPEP	CCS
FMNL1	COX5B
DERL2	NPTX1
KIR2DL1	NTRK3
TMBIM6	STAT6

NPC2	CD40LG
CYP2C8	ABI3
ULBP2	ANKRD40
GATD3A	CHIC1
VAV1	DENND2B
CTF1	EMC6
ELP4	HARS2
SH3BP1	HPS4
NXF3	IFITM6
SGF29	LRSAM1
MIR517A	LYZ1
MIR517C	MICALCL
A2MP1	MORF4L1
MIR6508	NUDT18
CXCL6	PLA2G2D
INTS11	POLDIP2
UGT1A8	PTTG1IP
HBP1	SHLD2
CYP2C18	SYDE2
FFAR4	TMEM203
CAD	TMEM40
OS9	DEDD2
KIR2DS5	FMOD
HHAT	GJC1
ATG10	ITM2A
RAB22A	KLHL5
TANK	NUP50
SMOX	RPL37
MYBBP1A	RPS5
CXCL3	SEC62
ASAP1	TMTC4
CAMK1D	TPCN1
SAMHD1	TUBB4A
EXOC7	ALYREF
CPB1	AMPH
LSM4	ANGPTL1
TAX1BP1	ANKRD33B
DHX30	ARL8B
BZW1	ARPC3
WFS1	ATG16L2
UGP2	C1QTNF5
MT-ATP8	CCDC126
F11-AS1	CLN8
SCARB2	CYP2C12
CCNK	DNAJC19
RBBP9	DUSP11
INSIG1	EDN2
GLYCTK	F2RL2
RNF103	FMN1
CHAT	GOLT1B
AFMID	HIPK3
TST	ING4
OTOA	ISCA1
PPM1H	LDLRAD3
MACIR	MRPL20
LRRC53	NDN
PTF1A	NDUFS7
CELA3B	NRAP

PSME2	NUP93
COL16A1	PANX2
LZTS2	PCBP1
PIR	RAD52
PTGDS	RALGAPA2
SNRPD2	RALGPS1
EPG5	RCOR1
ROMO1	RFNG
SCRIB	RLF
MAP3K11	SEC22B
SRCIN1	SELENOI
AP2A1	SESTD1
SERPINF2	SLC16A11
PABPC4	SLC26A1
ELF2	SPIN1
POTEI	SRPRA
POTEJ	STXBP4
USP27X	SUGT1
KIR2DS1	SUMO2
IFNGR2	TANC2
LOC108281177	TFAP4
PPP1CA	TK2
ABHD5	TOR1AIP1
GALK1	TRMT10A
MTNR1B	TXNRD3
SEC13	UTP3
MAFA	VDAC3
GTPBP3	ZFAND6
ZNF746	ZP3
FGR	GAS5
GDF11	KNTC1
POTEM	HNRNPU
KPNA4	SORBS1
ZNF423	INVS
SUB1	PPY
ABR	RNF169
JKAMP	SOWAHB
MIR520B	BCR
ACSL1	EHD1
SS18	GPRC5B
NETO2	KIFC3
ITGAE	LYPLA1
LOC106560211	SIK1
NAGLU	SLC29A3
PPARGC1B	SPRED2
PUM1	ZFP385A
MIR802	ACOT10
RPS2	AKT3
ARNT2	UGT2B17
LOC111255645	CAST
SNIP1	CGB3
SLC19A3	ETNK1
PCCB	GABBR1
NDUFS2	GABBR2
SEC24C	ITPR2
NDUFAF3	KCNQ1
UFM1	PFKFB4
NDUFAF7	PLCG2

POTEG	BTBD11
PDPK2P	CNTNAP1
ACTG1P1	DNMBP
ACTG1P14	EYA4
ACTG1P19	GTF3C2
LOC402221	HOMER2
SF3B3	HUWE1
LRP2	KAZN
DLX6-AS1	KLHL13
MIR543	NSD1
ENTPD5	OCIAD2
PHACTR3	OSGIN2
RAMP3	PALM2AKAP2
SOCS5	RBCK1
RIPK2	RNH1
USP42	SMARCC1
ACADM	SNRPB
HADH	TMEM163
NDUFS7	TRAPPC6A
SLC25A20	UQCRQ
MCCC1	ZRANB2
NDUFB9	ACTR10
NDUFA6	ANKRD24
MRPS16	APOL9A
NDUFS6	ASB7
TSFM	CENPO
NDUFA2	CYP4A31
DECR1	EXOC8
POLRMT	FUBP3
UQCRQ	GPR1
COX7B	HOXA11
GFM2	KLHDC7A
NDUFB11	MAPKBP1
MRPL12	METTL16
NDUFA11	MGA
MRPS26	NCAPH2
SDHAF1	NDUFAF2
COA4	NGRN
EIF3F	PWP1
RILP	SYTL1
NAA10	TAF12
TSTD2	TPPP
ELK3	TRMT12
ST6GALNAC2	VPS16
TACR2	UGT1A8
MIR320B2	BCL2L14
CSH1	BRI3BP
GPC4	CHST8
TM9SF2	CRELD1
ST8SIA1	DDX23
MIR616	EID1
POLR2K	GLYAT
RBP2	KCNA3
GIMAP5	MLF2
EIF4A3	MLX
SACS-AS1	NECTIN1
LOC105378976	PLA2G15
HSD17B4	PLAAT4

ADSL	PPM1L
KDM3B	PRSS1
PF4	PTPMT1
EVC2	RAB34
AFDN	RNF170
PLCD3	SCN7A
AP1G1	TET3
IFIT2	TRIM32
ABCF2	ZFYVE1
IL17D	GRIA3
LOC111162620	SST
SEMA3C	ARHGEF26
TINAGL1	CD99L2
RNF123	CFL2
PLA2G7	EPPK1
LOC110596866	NUAK2
WDR48	PIK3R5
RNASE1	RIPK4
NPTXR	RSL1D1
EPN3	SND1
CR1L	SRP9
LINC01551	SSPN
DLGAP1-AS1	STAG2
NCBP2AS2	RASGRP1
LINC00364	AP1S1
LINC01224	CALU
LINC02882	CTNND1
AURKAP1	MIR155
MIR888	RSRC2
LINC02476	UBA1
ZKSCAN2-DT	ARF3
LINC02835	CHRNB1
LOC111216291	CPPED1
TSGA10	CPZ
GCFC2	CRLS1
PDXK	DNAJC15
AP1B1	HDAC8
NELFCD	HSPA13
HIPK3	LRIG3
PLA2G4B	LRRC8D
GPM6B	LYNX1
CNTN1	NANS
PDE3A	PCMT1
SPRR1A	PTPRM
SIGMAR1	PTPRR
SCG3	RECQL
C4BPA	RIPOR2
ZWINT	SHB
LRRC59	TDRD7
PACRG	TNFAIP8L1
CPSF7	ATP5D
STX17	BOLA2
ATG101	WNT16
UBQLN2	ATRX
PLSCR1	MAFK
NRARP	ATP6V0C
PXN-AS1	CHD9
FAM172A	CHRDL1

PPP2CB	CTHRC1
TEAD4	DOCK4
CDK5R1	ETV6
C1S	FOXC2
TALDO1	GRK6
FARSB	NDUFB8
PSMA3	RBMX
VPS33B	SPTBN2
GATAD1	TLN2
KIR3DL2	TSPAN7
PSPC1	ARRDC4
YARS1	G3BP1
ADH7	HCAR2
WDR76	PDPN
CCR10	AP2S1
NR1D1	APBB1
RBM4	ATP6AP1
ESRP2	BCS1L
SUPT20H	CASD1
RNMT	CCDC117
PHF5A	CD58
TENT5A	EIF2B5
CNN2	ERC2
KRT23	FLII
TTPA	FNDC3A
KDM2A	KCNIP3
CATSPERZ	LRRCC1
ABITRAM	MANSC1
P2RY6	NDUFC1
ABCA2	NEK7
SNX16	NUP54
MAF	PRTG
MIR486-2	RBM26
APOBEC2	RNF24
RAP1GDS1	RNF26
CLDN12	SCFD2
DUSP11	SPAG4
F9	THNSL1
CBFA2T3	TSHZ2
PARP4	TTC7B
B4GALT7	UGGT1
ARFGEF2	ANAPC4
UBE2D4	ANKMY2
ACBD3	CYP2B15
RANBP6	DHRS7B
ZBTB10	GSPT2
VDAC2	HPS3
APOL1	KCTD17
MGAM	KLHL12
LGALS2	LSM14A
H3-3B	MTIF2
BRD8	NSUN5
ADRA1B	NUDT16
VARS2	PEX11B
PMPCB	PUS10
MYLIP	RESP18
ARHGAP27	RHOT1
RGS3	SERPINB6A

MAP3K13	SLC35F5
MRPL13	SMNDC1
RPA4	SUSD1
TM4SF4	SZRD1
TIPRL	TM2D2
INTS2	TMPRSS4
USP13	TXNDC9
CMTM7	KCNMA1
NRCAM	BDKRB1
UXT	ABHD12
LARGE2	ACP2
IFNL1	ETF1
CMTM4	FZD3
RANBP9	GAB2
PACSIN2	GANAB
AFF1	HNF1B
CLIC4	LHPP
GRHL3	NBR1
SLC22A4	NCK1
FAM3B	PMM2
PYCR2	RAB5C
GPRC6A	SPHK2
SLC44A3	TRAK2
CSRNP1	WNT2
PLTP	DDX3X
UBE2G1	HHLA3
NANS	NOL5
ETNPPL	PDHB
SH3GLB2	SCNM1
CARD11	SNX19
SLC27A4	ZFP704
KLF15	CHRNA2
CHMP3	DLX5
PLEKHO2	FURIN
FOXN2	KCTD15
TNFRSF17	RBM47
CMPK1	RERE
PNPO	SMIM3
IGHMBP2	SSBP1
CASP12	IL23A
PPP1R15B	EPHA4
CERS6	CHCHD7
PEX5	CSGALNACT2
PITHD1	DDX24
GPD2	DPF3
PRICKLE1	EAF1
KLF3	EHBP1L1
BTBD10	FRMD8
ASCC2	FUT1
ANKRD16	G3BP2
ANKRD23	GNL2
MIR6809	HAP1
HSD17B7	ITGB1BP1
ABCD1	OSBPL2
MAP3K10	RAB13
RNF168	RAB26
ERLIN2	SLC25A32
SAP130	SLC25A45

ZNF362	SPAST
MAFIP	TBC1D24
IL1R2	TCEAL1
RACGAP1P1	TLR8
STIM2	TRDMT1
SLC35B3	TSPAN6
ADARB1	UBL4A
LOC110599585	VTI1A
SRPK2	FTL
SNX1	LAMP3
KLRC1	TACSTD2
MYH10	CFHR1
STK38L	DNAJC17
PIGK	HOXC5
STAG1	TJAP1
RGMA	TMEM192
CD8B	ZC3H3
NFIL3	ZFP148
DNAJC7	ADIRF
SNRNP70	CAPRIN2
CHPF	CNNM4
ZNF510	CRCP
MAP7D1	CWC27
ZNF395	CYHR1
ABRAXAS2	DCLRE1C
MIR431	FAM120B
MFSD2A	HEATR5A
EEF1AKNMT	KCTD20
APOA5	LEO1
UGT1A3	LRRC49
DPAGT1	MIR183
GNE	MRPS2
GNPTG	MYOM3
ERVV-1	NUDT14
AOX1	RBM28
ERLIN1	RFX2
EDC3	RNF10
UBQLN4	SEC14L4
DUSP22	SECISBP2
PLCL2	SLC25A28
ZNF341	SLC41A3
C15orf39	TDH
AP5B1	TFCP2
ZNF83	THUMPD1
PPP3CA	TMEM165
GAS2	TRIM9
SMAD9	TUBD1
GATAD2B	ZDHHC9
APCDD1	BHLHA15
BAZ1A	CAB39L
TENM3	LPCAT2
FNBP4	NPC1L1
C9	PARP2
MARCKSL1	PARPBP
PIAS2	PGM2
HCFC1	SERPINA5
LMO2	SH3RF1
DBP	ADCY1

PPIAL4A	ADGRG6
KRIT1	GPATCH4
OSBPL2	GRB7
NFE2	GYS1
HSP90AA2P	LGALS2
MIR664A	MKLN1
F8	PRRX1
PIAS4	RASA3
ACAA2	SUOX
ALDH1B1	TOX3
IL20	AKR7A2
ARL13B	ANKRD17
TCTN1	CDX2
GRIP1	FER
PROZ	GNG4
MTMR4	GPX6
PCDH1	IFT88
COL21A1	KDSR
ALDH1A3	LIAS
TRIB3	MCEE
PPP1R1C	MORC3
GPR182	MS4A7
GNPAT	MTRR
EDEM1	NDUFB9
CEP41	NHLRC2
SRP72	PUF60
ZC3H15	TEP1
ASB8	THOC1
WAPL	TPST2
CCDC86	TTC30B
KLRB1	VCPIP1
MIR939	CLCA1
MIR320B1	ESRRG
MIR1285-2	FGF8
LIPE	GOSR2
CYP4F12	L1CAM
LPGAT1	MALT1
PAMR1	MAN2A1
CYGB	MLLT11
RPL32	NISCH
ANKRD13A	SEPTIN4
MBTPS1	STX1A
MAN2B1	CACNA1C
FKBP8	PRKG1
IQSEC1	ABCF2
IL17B	ACMSD
PUS1	AMPD2
MYO1A	ANKH
SNRPA	ARHGAP45
SLC15A4	AUTS2
HIVEP1	BNC2
DNAJB12	CDKAL1
SLC23A2	CTDSP1
METAP2	GDPD1
RBM24	GMPR
ZFAND4	HIC1
MIR889	KHSRP
LOC109286563	PDHX

CSDE1	RFK
CDC23	SNRPA
RGS2	SNRPF
KYAT1	SPCS3
THOC1	SRP72
TBX4	SUZ12
ISYNA1	SYBU
ACER2	TRIM36
MRI1	UBFD1
XXYLT1	AHSA2
CLIP3	CES4A
UBE2B	HPR
MKKS	LOR
TMEM67	LY6C1
ROBO4	NELFCD
IGHA1	REG3A
TRIM71	ALG3
ITIH3	AMHR2
SUPT16H	DOCK3
MARS1	GDF3
POLDIP2	HIC2
KIR2DS4	ORC2
ANKRD1	PLD4
KIR2DL2	RC3H1
SORD	RRAGA
CES2	ST6GALNAC6
NFATC3	TMEM208
PCTP	UBAP2
SNRPD1	VIT
LOC110013312	BRD2
SLC12A4	NECTIN2
PDIA2	SPC24
GAS8	ADSS
UBQLN1	ANKZF1
GLDN	GREB1L
F12	INTS6L
GK	KCTD6
PPP1CB	METTL27
OAS1	ODAD3
NUP155	TNFAIP8L2
MGAT1	ZNHIT2
GGA1	BACE2
RAB4B	DAB1
IER5	DDAH2
NIBAN2	FZD6
QARS1	GMFB
OSBP2	KCNK5
SOX13	TAPBP
B3GNT3	A1BG
CA11	ADGRD1
PPIP5K1	ANKRD44
ETHE1	CERS4
ANGPTL1	CGRRF1
COG8	DENND3
TMED6	DTNB
DTYMK	EPC2
FUT9	GFER
PKIA	GFM1

CEP250	IRAG1
HIPK1	KLB
PITPNB	LILRB4
ASXL2	MAP7D1
ANKLE2	MAVS
ELMOD3	PROK2
SH3TC2	PRXL2B
PHYKPL	RSL24D1
CLINT1	RSPH1
VPS41	SERTAD4
WDR59	TM6SF2
WIPF2	TTC17
CEP192	USP43
CFDP1	ZNF395
DRAP1	BZW2
CDC42EP2	CCR4
DCP1B	CDK12
LRRC41	CLCN5
SPATS2L	HNRNPM
IRF2BP1	IARS1
RBM23	KDELR2
SUSD1	MPC1
THSD7A	MYO5C
ZNF512B	NDUFB3
ZBTB11	NOX1
GIGYF1	PFAS
ECHDC3	PHACTR2
OSBPL10	PIEZO2
SLC38A10	RCC2
NT5DC1	REEP1
MFSD6L	SERINC5
CCDC127	SOX5
MTMR11	TCF7L1
LDLRAD4	TRIM63
PILRB	TYK2
THAP4	TRPV4
FAM43A	DNAJB6
ARRDC2	RYR2
ZNF443	CALCA
GPBP1L1	TAC1
XAGE3	CCR6
LPAL2	CST6
C22orf46	KALRN
HSPA7	PADI2
BAALC-AS1	PRRC2C
CRYZL2P-SEC16B	AP3S2
CENPJ	APOL6
MAFG	ATXN10
P4HA1	AVEN
KIR2DL4	B3GALT1
TTC21A	CAPN8
H2AW	CCR5
MORF4L2	CD1D1
GABRG2	CLUH
NPC1L1	EIF3M
LHPP	ESRRB
GRHL1	FEM1B
GMPS	IPP

UGT2B10	IWS1
GPX5	KLHL9
MKS1	LIN54
TTC8	LPAR2
EVC	MRPL40
TMEM237	MXRA5
PSRC1	SPRR1B
DCTD	TADA2A
MRPL3	TM2D1
LIMK2	TMEM69
NM	VPS36
FHL3	VWA1
CNKSR1	APOL7C
INVS	EXD1
TEAD2	SERPINA4-PS1
IGHM	JMJD6
MIR544A	MT1H
MIR1258	PDE4A
RPL10A	TBXA2R
HELZ	VGF
RHBDD1	ATRN
HDGFL3	CCDC68
SPNS2	CLIC5
PSG5	DDX60
CNTFR	GCDH
POLR1A	GRIK5
TOP1MT	NOL8
SNX14	PCP4
JCAD	PLA2G4C
MIR1269B	PRMT5
SELENOS	RIT1
LOC111188164	RMDN2
LOC111242783	SLC27A3
STAM2	SPOCK1
CDC40	TP53BP1
RSPO3	AMMECR1
RYR2	ARHGAP20
ECSCR	ARL3
CYP7B1	ATG16L1
ADI1	BANP
ATF7	BRINP1
TAF9	CBLC
STAB1	CES1C
PRSS22	CES1F
CENPM	COQ7
LYPD6B	GABARAP
SELENOK	IKZF2
PIP4P2	JPH2
SNHG10	MED10
LINC00205	MYH3
ROR1-AS1	NDUFA6
ZNF385D-AS2	PPP1R13L
RTE1	RAB2A
ADAMTSL3	RMND5A
CYP51A1	SCFD1
LOC110467516	SHOX2
GNA14	SMN1
RPL27A	SNRNP70

SIK1	SOX3
CPNE3	TRF
RDH10	TUBGCP3
ZBTB24	TUG1
PTGIR	NEFH
LMAN1	SLC22A6
RTN3	IFI27L1
SLC30A9	IPO13
LRRFIP1	MRPS25
ADAMTSL4	NOP14
H2AC19	PKNOX1
MIR1228	TUBGCP2
NAGK	ZKSCAN3
FBXW11	DPT
OAZ1	PIK3C2A
RPS25	SLC4A2
TCTA	SP5
BMP8B	ASB11
CDCA3	HMCN2
IL20RA	IZUMO4
RNPC3	STFA3
HCAR1	CRIP1
PRKD2	NNT
PTGR1	RIPK3
L1TD1	NTS
SRM	CAP1
IL19	CBFB
SLC23A1	CIITA
MEP1A	CLSPN
UBE2V1	CPB2
ARCN1	CYP46A1
RAB5C	DEPDC7
APBB1IP	GLRA1
HKDC1	MCM8
RNF114	MLC1
SSR1	MNS1
STAU1	NAA38
MTREX	NPR1
LOC109286556	SASH1
SMPD3	ARMC8
POLR1C	BOLA1
HDAC10	CFAP20
EXOSC8	CSPG5
ZMYND11	CTDSPL2
ZCCHC8	DDX25
CALCOCO2	GMPPA
JPH4	GNPTAB
INPP5E	GORASP2
PCSK5	HIRA
DGKE	HSPBP1
AKR1D1	MICALL2
IGFALS	MIR214
ARHGEF18	MIX23
RPIA	NDRG3
PTPN5	NDUFB7
UBE3B	NXT1
P2RX2	POU4F2
GNPTAB	PUM2

AP1S3	QKI
ARHGAP29	RASSF7
FN3K	RNF11
MOB1A	RNF7
PDLIM1	SCEL
SMOC1	SIDT1
GGA2	SLC25A46
B4GALT3	SPATA6
ARHGAP10	SPG20
MAF1	TINAG
ACSM3	TMEM119
EPS8L2	TMOD4
EPS8L3	YLPM1
RHBG	ACTR2
MYL6B	ADAR
PLCH2	ARHGEF9
USP30	BCAM
LMAN2	CAPN3
FLG2	CD3E
MEX3B	CEP70
CHML	CLDN23
EIF5B	CLIP2
PARP10	DIAPH2
CCL24	DSE
FKBP7	EIF2AK1
FRMD5	GALK2
COPG1	HSPB2
USP29	LARGE1
ANAPC4	MAP3K3
BRI3	MEN1
FUNDC1	PPTC7
FCRL1	RAP1GAP2
RBFOX3	SDHD
TBRG4	SLC44A3
TMBIM1	SMARCA1
IFNLR1	SNX9
YTHDC2	SULT2A6
MIF4GD	SUSD3
UNC50	SYTL5
GPR137	TMEM107
SH2D5	TRIM38
WDR70	VWCE
ZCCHC13	ATP1B2
TRIM50	FLG
UAP1L1	KCNQ2
ANKRD34B	MRAS
IGFL3	PDE7A
ELSPBP1	GAD2
RBMY1F	HJV
ACP7	MXD3
RBMY1J	RNASET2
KIR2DS3	SBK1
RBMY1B	SF3B1
RBMY1D	STEAP2
RBMY1E	TFEC
KIR2DL5A	UBR4
NRON	USP36
SNORD126	ABRACL

FAM86B3P	BIRC7
CAMTA1-DT	CLDN9
H3P16	DCLRE1B
LOC112543491	FCRLS
LIMS2	LHFP
OSBP	NAA16
STARD7	NCDN
SLCO6A1	NDUFA4L2
SCP2	PAPPA2
PPP1R3A	PHF20
TOPORS	PICK1
ATP6V0C	PIGH
NAIP	SCRN2
TMEM231	STRN4
CD84	THAP4
ANTXR2	UBAC1
MIP	USP6NL
HMG20A	CACNA2D1
TAF10	CALB2
MORC4	CASK
ISCU	HTRA2
MBD3	PDLIM2
ITIH1	CHRM2
TRA2B	CHRM3
GGT7	ARHGEF16
SNAI3	CKAP5
SOCS7	COX11
MRM3	DDX18
APLP2	DNAJC21
STRA6	DYNC2I2
SON	EMG1
AP2A2	FAM171B
MMUT	HSD17B13
PLEKHG6	KCNJ16
GOLIM4	LSM6
SLC25A4	MLYCD
UNC45A	MRTO4
MAN1A2	MUSTN1
STAM	PITPNA
ACOX3	PKNOX2
C4BPB	PPM1H
GOLGB1	RHEBL1
SNX17	RPL35A
UBXN11	SDAD1
ANKRD27	SLC6A14
ABTB1	TAB2
STBD1	TRPM6
LOC110599575	TRRAP
LOC110673974	TTC9
HEYL	VAPA
GSDMD	ABHD14A
LTBP3	ACVR2A
TXK	ANKRD46
STK25	ATXN7L1
PFDN5	BAZ2A
AIG1	CD79A
PI15	CEP170
DEPDC7	CHRD

LCORL	MIR182
SECISBP2L	MIR27A
MIR1203	MRPS30
ANK3	NBEAL1
ANAPC2	NCOA5
CD7	TIMM8A1
SUPT5H	TMEM109
ASCC3	TMEM62
MTF2	TTC14
FBXO38	ERO1A
COPB1	CCL21B
NIT1	CES3A
TRIM62	CIB3
HTATSF1	COL24A1
CAMSAP1	FOXI2
PPIL4	PNPLA5
CNTROB	SPIN2B
ANKRD28	ENG
CECR2	HEXIM1
NKRF	IL32
LAMTOR1	ITGB3BP
BLNK	KMT5B
HRH1	PIK3IP1
RAB4A	PIP4K2A
RPL28	PLXND1
HMBOX1	PTPRN2
LGSN	RAB6A
RNF4	SNRPN
TMBIM4	SPA17
SLC6A6	BBX
ZSCAN4	SLC22A23
LOC107372315	ADRB1
MSTN	DPYSL2
SPR	ARPP21
CYP4A11	ATP9A
AIFM3	CBX6
PDLIM2	CCRL2
BTBD2	COLQ
PDCD7	CSPP1
ZBTB3	DMXL2
SLC6A4	EYA2
ITK	FAM110C
RNASE2	GATAD2A
ATRN	GLCE
ZAR1	INS2
CNTN2	LNX1
PNKP	MARVELD1
SLC27A2	MTMR4
RLBP1	ND4
VAPA	NUP88
PIGQ	PIN1
PAPOLA	PRKG2
CAPN6	PRRC2A
SGMS1	RAMP3
PITPNA	RPL39
DSTN	SGPP1
UCK1	SH2D4A
MX2	TRAF3IP2

ORAI2	ZFP37
GAL3ST4	SHANK2
MRPL19	BRMS1
OSBPL3	CCDC107
OSBPL6	CUZD1
OSBPL7	CYP4F11
ABHD11	DTD1
HSPBP1	FASTKD1
ITGAD	GOLT1A
RBM19	GSTCD
SPZ1	HBG1
TXLNG	INKA2
CSH2	KBTBD7
EMC2	MED18
JMJD8	NHLRC3
KMT5C	NOL7
DENND2B	RBBP5
SLC49A3	SUPT3H
IGKV2D-29	TAFA5
LOC106707173	TENT4A
NPHP4	TYW3
SLC5A2	USP42
IDI1	CXCL11
PDE6D	ABLIM2
TRHR	ADH1A
KCNN2	AGPAT3
RABGEF1	ALPK2
TIRAP	BTRC
MYLK3	CLPTM1L
EPN1	CNBP
PPP2R5E	CPXM1
DBNL	ETV1
SCRN1	FICD
KIF3C	FLRT3
TRIB2	G6PC3
SERINC2	GRIN1
ZNF451	HEY2
GMEB1	HLCS
CHTOP	HOMER3
BUD23	IPO5
WDCP	IVL
MT1JP	PRG2
MAN2A1	PXYLP1
GPD1	RAP1B
H2BU1	RNF43
TRIM63	SAP30BP
CEP290	SAR1B
BBS1	SPPL2A
DDX3Y	SSR1
MIR4484	STYX
DLGAP1	TMEM263
LARS1	USP39
RNF17	WASF1
CYP2D7	YAF2
KCNQ2	ZBED5
FMO3	AARS1
C1R	ALPP
SLC19A2	C1QTNF4

KYNU	CDH6
B3GAT3	CPSF6
TLK2	CSDE1
EIF2B4	FAF1
SEC24D	FARSA
KCNJ4	GALNT12
PTDSS1	GHRH
CLPB	GYG1
CLCN4	HINT3
DNAJB2	KCNQ3
EIF2B5	MARCHF2
PEX19	MARCHF3
TREH	MRPL13
CCM2	NCKAP1
HIRA	NKX2-5
RNF216	NSFL1C
SEC31A	PCBD1
ITSN1	RELL1
STX16	RGS20
ENTPD3	RNF145
MLC1	RNF152
DAAM1	SAPCD2
TOM1	SCUBE2
LEMD3	SH3GL3
SORBS1	UBTF
IL36RN	VPS41
VPS11	CPEB4
F13B	CYP2R1
EPN2	GSK3A
METAP1	MTF1
OAS3	CLEC2H
UBA6	CYP4F16
TSEN15	FUZ
TBC1D1	HSD3B3
RPS28	TGS1
ZNF41	ACTN2
MAN1C1	DNAJB2
ARFGEF1	ERAP1
ALG12	GPX8
ACMSD	LRP6
RHOT2	MAP2K7
DGCR2	NPTX2
RFK	PGAM2
OGDHL	PHF19
SLC43A2	POLK
PSMD8	RAB5A
TRAM1	RPS27A
TTC3	SLC25A12
TTC37	TMTC1
TCERG1	TNRC18
ZFYVE16	UFD1
CCL16	ARC
ASB2	NR5A1
EXPH5	TP63
LRTOMT	ACOT8
MED4	ATAD2B
ABHD6	BNC1
DCP1A	CD2

RNF144A	DLX4
PPME1	HOXA2
SLC38A5	IFI204
LAPTM4A	LSM10
KIAA0319L	NAE1
KLHDC8B	PREX2
LDB2	SCOC
AUP1	SLC35E3
BATF3	STRAP
APOF	TBC1D30
ESPN	TIMM21
CTR9	TMEM120A
MPHOSPH10	UTP6
MOB1B	XPO7
DDX27	AIDA
DAZAP2	ALG9
MPPE1	APIP
NEU4	ARHGAP6
PSG3	ATP13A1
PAPPA2	CYB561D2
OSBPL9	ELP5
UGGT1	FAM184A
ZDHHC3	GLB1L
ZNF76	HCCS
AZIN2	ISY1
GDPD5	LIMD1
CCDC65	MED4
CUEDC1	RELT
RMND5A	SETD3
EPS8L1	SHPK
MTFR1	SLC4A3
EFHD1	TIAM2
CALCOCO1	TMEM258
CREBZF	VPS39
SF3A2	VTI1B
IFT20	FBXW9
SNRPD3	SLC24A5
SAA2	CNP
SDK1	PRKCG
OAZ2	PSME4
TRIM69	ATP5B
UBAC1	ATP8B2
REEP4	BATF
TMED1	CCNL2
CNTRL	DMKN
THAP11	DVL2
THAP5	DYNC1LI2
ZFYVE1	EMX2
ZCCHC7	GLIS2
YIPF5	KIF21A
VPS8	NDUFB5
UBXN1	NPHP1
AVPI1	RALBP1
DOHH	RGMA
GPR107	SH3BP4
PLEKHB2	TXNDC16
NSMCE4A	CACNA1D
PID1	COX3

TOX4	DDIAS
TMUB2	EBI3
TRIM41	EIF6
TSKU	IL16
QRICH1	POLR3G
TMEM214	PTGFR
KIF18B	RPTOR
RNF167	SLC31A2
VCPIP1	SOBP
ZNRF2	SVEP1
VPS51	AMFR
RMND5B	COL4A4
CLEC14A	CPT1C
CSRNP2	CYB5R2
NRBP2	DENND2C
THAP2	DENND5B
ZMYM5	EGFLAM
ZNF106	FKBP8
ZBTB44	GABRB1
FAM161B	IL3
MTMR10	KCTD9
MSL2	LMF1
MRFAP1L1	MIB1
SPATS2	NAALAD2
PLGLB2	NDUFA11
NUDT22	NUP58
TPRN	POLR1A
TBC1D10B	PUS3
ZFP2	RIPPLY3
ZNF484	RNF141
ZNF557	ROPN1L
ZNHIT6	SLC15A4
TPRA1	SLF1
UGT1A5	BARX2
YIF1B	CHD4
YLPM1	DNPH1
C11orf24	GPR3
C11orf54	HINT1
CCDC181	HOXD8
FADS6	KRT86
METTL22	MEX3B
C9orf64	MIR148A
CLEC4C	NDE1
PPP1R26	OST4
TMEM139	PDK3
ZNF518A	SELENBP2
ZNF329	SYAP1
ZNF107	TAOK1
ZNF800	UBXN6
ZBED5	AFAP1L2
ZBTB6	AOPEP
ZZEF1	ASTN2
NCKAP5L	CD79B
PPP1R37	CHST1
LBX2	CLCN7
RUNDC1	EHF
ZNF587	EMCN
ZNF675	EXOC3

CNMD	GHITM
UBALD1	GRHPR
PUM3	HIRIP3
OR4F6	KRCC1
MACO1	NEGR1
CEMIP2	OSBPL8
ZNF587B	RASD2
MYG1	SEC13
TLCD3B	SFXN4
DEPP1	SH3GL2
DHRS4L1	SLC12A1
DIPK1B	STUB1
PLAC4	TMOD2
DHRS4-AS1	TTC39B
GOLGA2P5	TTLL7
PLGLA	WWP1
HSD17B7P2	AKR1C20
MIR525	CBARP
LINC00273	CD180
TEKT4P2	CHPF
GSTA7P	CPNE7
GPRACR	CPSF1
B3GAT3P1	DZIP1
LOC100134317	ECEL1
IL6RP1	FAN1
HVBS7	GET3
LPAL1	LRRC42
AIR	LRRFIP2
LOC110599569	MIR193A
LOC110631417	MRPS24
LOC110740340	NARS2
LOC111216288	POMGNT1
LOC111242785	PROZ
EGID-106632268	RANGRF
LOC107963949	REG3B
IFRD2	SH3GLB2
KCNH6	SHROOM4
OAS2	SLC41A1
PPP4R1	SNRPB2
FETUB	TMEM159
TAS2R38	TMEM65
RCOR3	TMEM71
LRRC47	TOMM34
MIR320C1	TRMT5
LOC110599580	UNC50
GDA	ZMYM6
ABCD2	FEZ1
EXOSC5	KRT14
HS3ST3B1	RAB3B
PRELID1	ADRM1
ZNF300	BPGM
ADGRF1	CPOX
MIR1908	EML4
MIR573	MARS1
MIR921	NDUFA2
EPHA6	SLC27A4
MOAP1	TNFRSF10D
ANGPTL6	AFAP1L1

C3orf33	ARVCF
CMTM8	CBR4
MTCH2	CPXM2
B9D2	CSTF2
FGF20	DHRS1
GZMH	DOK4
ZBP1	H2-AB1
GNG3	HK3
WDR19	IFT74
ADAMDEC1	IMMP2L
F11	KDM2A
MMRN1	M6PR
DYNLL2	MARVELD2
PZP	MIR93
IFI6	MNT
EXOC8	PFDN5
JCHAIN	PRRC2B
ZBTB4	RABGGTA
IGHG2	RMDN1
IGHG4	SCTR
IGHA2	SCX
MIR658	SENP2
NPHP1	SF3A2
ARL6	SGSM2
BBS4	SNTB2
OFD1	UMOD
IQCB1	UTP15
GLIS2	BSPRY
BBS10	CENPQ
IFT122	CTBP1
TCTN3	CYP4A3
BBS5	DEPP1
CC2D2A	DSN1
NPHP3	EIF4E3
TCTN2	GALNT16
BBS7	OTUD1
ANKS6	PARG
TTC21B	PFKFB2
IFT80	RFLNB
B9D1	SLC15A3
TMEM216	SLC17A1
TMEM138	SNCG
BBS12	WNK4
CPLANE1	YKT6
GP2	EEF1D
SDF2L1	ICE1
UBP1	MAPKAPK3
SERTAD2	SUMO1
NME2P1	SUPT4H1
MIR19B2	ABHD17A
MIR190B	ACAP2
LINC01137	ANAPC16
PCNAP1	ANGEL2
PFN1P2	CDKL3
PFN1P3	CHMP4B
POMT1	CMPK1
MID1	CYP2T1
DHDDS	EIF3L

CFP	GGNBP2
INPP5K	GRAMD1A
POMT2	HOXD10
KIFAP3	ISG20L2
LHX4	KDM5C
ADAMTSL2	MAF1
SP110	MYBPC2
AKAP10	NICN1
DRAM2	NOL11
COPE	OASL1
VPS28	OBI1
C1QTNF3	OLR59
CISD1	PHOSPHO2
SPPL2A	PPP2R5D
APOBEC3C	PTPDC1
FBXO2	RBM15B
CHP1	RIMBP2
PPHLN1	RSAD1
PLP2	SERGEF
SLC38A9	SPATA2
HYAL4	STIM2
PLEKHF2	SUPV3L1
TTC1	TEX10
PDZRN4	TNFRSF10C
REG3G	TSN
SYT13	UTP11
APOO	ZFP207
PARM1	ZNF385B
VPS37B	DCTPP1
MRGPRX3	DPYSL4
PLIN5	HEG1
PYROXD2	LMO4
RBM12B	MAP7
SP5	NACC2
ZCCHC4	NRGN
TAS2R46	PLS1
JADE2	SULT2B1
CNEP1R1	KL
CCDC146	ABCF3
SULT1A4	ADAMTSL5
TENT2	AIM2
VIRMA	ALKBH1
RSRP1	ASB6
TENT5D	CTIF
CASTOR1	CYP2G1
TENT5B	GPN1
REXO1L1P	H2BC15
MIR320E	MTRF1L
MIR323B	PEX11G
MIR378I	PKHD1
MIR378D2	POLR2K
MIR378E	PPP1R11
MIR5193	SSH3
MIR4787	UGT2B34
MIR151B	ZDHHC16
MIR3679	BAG1
MIR378C	MEF2A
MIR320D1	ADTRP

MIR4505	AIF1L
MIR4476	IDH3G
MIR4741	JMY
MIR513A2	KLHL29
SERTM2	MSRB2
SAMD12-AS1	NAGA
MIR3678	NTHL1
MIR378D1	OSTM1
MIR4257	PDLIM4
MIR4324	PLCD3
MIR320C2	RPS9
MIR320D2	SARAF
MIR581	SEC23A
MIR4632	SPCS2
MIR4638	TAF15
MIR4481	TSPAN2
MIR3714	UACA
MIR3616	WDR33
MIR2113	AGAP2
MIR3135B	ALMS1
MIR1973	ARHGEF1
MIR4467	ATP10A
MIR4749	BET1L
MIR4310	CDH22
MIR4717	CLDND1
REG1CP	CPVL
RIEG2	CTNNA2
HNP1	CYSTM1
LOC109279247	DENR
	DLG3
	DPM3
	GAS2L1
	KAZALD1
	MIR200A
	MKRN1
	PARD3B
	PDS5B
	PPIL4
	PTCH2
	PTS
	PUS7
	RBM4
	SFRP5
	SPATA7
	SPR
	SPTY2D1
	STAM2
	STN1
	SUMF1
	TWNK
	UTP4
	VPS29
	TG
	CYP2C19
	IFI44L
	MMP19
	RBP7
	SGMS2

STIM1
XRCC2
NAV2
PECR
ACRBP
CDIPT
CTNNBL1
CYTH2
DCUN1D3
DLGAP2
EIF1B
ENHO
FAM135A
GABRR1
KANK3
KRT2
MAEA
MAST2
METTL18
MRPL52
MSTN
P2RY4
PHPT1
PIGK
PITX3
RGS18
SDK2
SNRNP200
SNX6
TIGD2
VSTM4
ZFP503
ADAM22
CHN1
FNTA
HAGH
KAT6B
MIS18BP1
POLR3E
PRCP
R3HDM2
SCIN
SEMA4A
MAFG
SLC18A2
AFMID
BET1
CEP76
CIART
CLN6
DCAF7
DERA
DGKZ
FKBP2
FOXF1
GCSH
GIGYF2
H3-4

HECTD2
IIGP1
MARK1
MED21
METTL1
MIB2
MOSPD2
MPHOSPH9
MTFP1
NLRX1
NMRK1
ORC4
OTUD7B
PPIH
REG3G
RIN3
RRP15
SMAGP
SPNS2
CCL12
CD28
COX7B
CSNK1D
FZD5
MYL6
POLR2A
SCAMP1
SH2B3
CER1
ERDR1
FAM131C
B3GNT9
BRF1
C4BP
CDK5R2
CES2B
DNAJC14
DYNC2I1
FBXO43
GPN2
H2-DMB1
LAS1L
LEFTY2
MED12L
MIR362
MIR410
PSENEN
TMEM138
PTGER2
AP1M2
AQP11
CD164
CNTRL
CRMP1
CTCF
CTNND2
FOXJ1
HACD1

KIF21B
MAN1A2
MAST4
MOB3B
MSH3
PPP3CB
PSMD6
RENBP
RFX5
RPL24
SDCBP2
XCL1
ALPI
CYP2C6V1
EPHB2
GPR137B
INA
PSMD11
SPAG9
AFTPH
AKAP6
AMT
ANKRD28
ANO10
ARHGEF12
BANK1
BMP3
BRCC3
CCDC51
CHODL
DCTD
DDI2
ERC1
EXOSC4
FOXJ3
GOSR1
HOXB5
HYDIN
KRTCAP3
LONP2
MRPL48
MYF5
NHP2
NTAN1
NUDC
NUDT3
OASL2
OTULINL
PALD1
PAX7
PBRM1
PGAP1
RAB28
RDH12
RHBDD1
RNF2
SEC23IP
SFMBT1

SLC33A1
SMC5
SMCO4
SMURF1
SNHG32
SPINK5
SPTB
SPTBN4
STARD7
SUN2
TGIF2
TM2D3
TMEM42
TOMM70A
TREH
TTC12
TTLL1
UGT2B
USP12
UVRAG
VPS4B
VPS52
ZBTB40
ZBTB5
ZDHHC20
ZFP367
ZFYVE28
POLB
IRAK2
QARS
SERHL
ZFP622
ADH1B
AKAP8L
C1GALT1C1
GLCCI1
IDH3B
IKBIP
KCNAB2
LYST
PLA2G5
RETNLA
RYBP
SETD5
SLN
TMCC3
USP25
VAMP5
HS3ST1
MAPK6
MEF2D
SCG2
SCNN1B
AGK
ANAPC7
DDA1
DYNLRB1
ENOPH1

GOLGA7
HECW1
INTS12
LMBR1L
MAD2L1BP
MIR223
POP4
PTCD3
RDH13
RINT1
RNF185
SNAPC4
TOR2A
XAB2
KRT1
NPY1R
BPHL
CASQ2
CENPV
DGKI
ECE2
EXOC6
FAM241A
FOLH1
FYCO1
GDF9
GGPS1
GRHL3
GUK1
HLA-F
MRPL3
MTM1
NDUFA12
NTM
P3H2
PGGT1B
RPS28
S100A2
SDCCAG8
SNX7
SYNJ2BP
TMEM39A
XPO6
YARS2
H2-D1
LRRC24
NLRP6
SERPINA1B
TRIM34
WDR82
ZFP53
ZFP655
AATF
ACER3
B3GNTL1
BRAP
BRD7
CEP170B

COX10
DDN
GOLGA3
HSPB3
IGSF8
MAPKAPK5
MIR34C
NOL10
P3H1
PRELID3B
RCBTB1
RILP
SF3A3
TBPL1
TRAPPC1
TSSC4
TUT4
UPRT
WDR54
ZC3H8
ZNF217
FH
CENPJ
CTTNBP2NL
IFT80
KCNAB1
NUP98
RGS12
RPLP2
RPS17
SEMA3F
SLC14A1
STAC3
TENM4
CBL
PLCB4
IL13RA2
BAG5
CEP152
CIB2
DHX15
DLEU2
ELANE
GNB5
GTDC1
ITGA11
MCOLN1
MGAT4A
NAAA
NOXO1
NUP160
PLEKHH2
PLEKHO1
PLPP5
RAB10
RAB18
RAB8A
SF3A1

SLC25A11
SPATA20
SYNPO2
TAF1
UNC93B1
NF1
CHGB
GATA1
GMPS
GNG2
GRIA4
NIPBL
PTPRJ
RGS17
SCN1A
SFTPD
AARD
ANGPTL8
BAIAP2L2
CCDC137
CXCR6
FAM76A
GORASP1
KLHL22
KLHL8
LY6C
LYRM1
MSTO1
MTX3
PCGF1
POM121
PTK6
RALGAPB
SMAP2
STARD3NL
TAPT1
WFDC18
YIPF1
ACTL6A
ADAMTS12
ARHGEF40
CCDC25
CIB1
CIP2A
CYTH3
ELL
LRFN3
MAP3K13
MARF1
NMNAT3
POLR2L
PRPF3
RAPGEF1
RBM43
RHNO1
SNAPC3
SNX2
TCF15

UBL7
UBR3
UGT1A5
WDR5
ZFAND2B
UGT1A10
GNAS
ARL4D
BAIAP2
CAMK1D
CYP2D6
GABRB3
GHRL
ICAM2
KDM6B
ADH6
ALPK1
BCL9
CARD6
CCDC88A
CDCA4
CYP4F2
DENND4A
DNTT
DOCK5
EIF1AX
ELF4
EPSTI1
GJA5
GRWD1
H13
H2-Q1
HLA-DMB
IFI27L2
LMNB2
LMO1
MIR200C
MPRIP
MUG2
NCF4
NDUFB10
NRBP1
NXT2
PCLO
PLEKHA2
RMI2
RPL23A
RPS19BP1
RTN4R
SLC12A5
SORCS2
SWAP70
TFB2M
TM9SF3
UXS1
SLCO1A2
HRH1
ACAD8

BTBD9
DDX52
DENND2A
DIRAS1
EXOSC6
HOXB7
IFNAR1
LRRC3B
MAML1
MAPKAP1
MEGF9
MESP1
MIR10A
MIR150
MRPS34
PIGF
PROCA1
RASL10B
SHC4
SHE
STXBP3
TBRG1
TRIM29
TRIM33
TSR1
UGT1A6A
UNC13A
WIPI2
YME1L1
ZFP64
TRPV1
ADAL
CC2D1A
CIAO2A
DNAJC8
EIF3K
FBXO41
HELB
LRRC15
MEP1A
MRPS21
MRPS5
NAT8F2
PJA1
PSMF1
RBM10
SETDB2
SHISA4
SLC6A4
SOGA1
STAP1
SUPT5H
TARBP2
TBCC
TMEM9
TRAPPC3
TRPC4AP
TTC38

ZDHHC3
ZZZ3
ASIC1
CAPN6
GCNT1
IBSP
PAX8
SRSF10
VAMP1
WASF2
ADAP2
AGPAT5
AVIL
C15ORF48
CNNM2
DNAJC5
DONSON
EFTUD2
GPR160
HOOK2
IGSF11
LDB3
MED13
METTL7B
MYO9B
ND5
NELL1
NLGN3
NXPH3
PBLD
RASSF3
SCG3
SPTSSA
TBX1
UNKL
USP13
XPNPEP1
CH25H
P2RY1
AVP
SLCO1B1
ANKRD10
CD22
CTF1
JAG2
KDM4B
LTBP3
PCSK1
PRPH
RAPGEF3
SELPLG
TNRC6B
GNAQ
PTAFR
SERPINA3
CEACAM6
CHKB
CYP2D4

DPH2
ELOA
FIP1L1
GSTM7
HNRNPLL
IRAK1BP1
KIF16B
MEIG1
METTL3
MIR205
MRM3
MS4A6A
NENF
NKG7
NUP107
PAFAH2
POLR3D
PPHLN1
RAE1
SLC39A7
SMAP1
TMBIM4
UBE2Q2
USP33
VAT1L
WDR90
XRN1
XYLT1
COCH
KCNJ11
NKD1
APON
BORCS5
BPI
H2-DMA
MFSD2
MUC6
MYCBPAP
PRAP1
RPS6KL1
TFAP2E
TRIM41
WFDC12
ALOX15
HSPA6
C2CD2L
CHIL3
CMIP
COPZ2
DVL3
EPM2AIP1
ERP44
GGCX
GNA11
IFT122
IGDCC4
MGAT4B
NHSL1

PCF11
PDCD6
PPP2R3A
RIN1
RPL19
SETD2
SH3PXD2B
SNX16
THSD4
TPH1
VGLL3
ZC3H11A
AGGF1
ANAPC10
ANKS6
ARL10
DDX51
DESI1
DGCR6
EIPR1
ELFN1
ENDOU
FGF6
HABP4
HOXA3
LSM8
MUC16
NEMP1
OTOF
RNF122
SPRYD4
TCHH
TM9SF4
TMEM134
ANXA10
DOCK9
EIF2AK4
FAM149A
GLI3
GOLGA4
KDM5B
NMU
SARS1
SLC12A8
TMED5
TPBG
CEL
CYP19A1B
FRY
RPL13A
RPL7
TYRP1
CYP4A32
KRT25
M1AP
SPRED3
AKAP11
AMN1

ARHGAP31
ATL3
CEP68
CLASP1
CXCR5
DAP3
DOCK2
ECPAS
EVA1B
FUT4
FYTTD1
GTF2H2
HIKESHI
HOXA9
HS6ST2
IAH1
IFT57
LOXL4
MBLAC2
MYL12A
NDC1
NDFIP1
NLK
NUFIP1
NUFIP2
PDSS1
PGLS
PKP3
PLPBP
PMPCA
PTGES2
PTPRA
RHOV
SAMD11
SDHAF3
SF3B5
SLC17A3
SPTLC3
SYT13
TEAD1
TICAM2
TMCC2
TMEM117
TRIM21
UBASH3B
UBE2M
VWA8
WAPL
SERBP1
DSTN
FBXO9
IDS
PLEKHA6
SAMD9L
SCN4B
SCNN1G
SEPTIN9
SLC6A1

TLE4
GRIA1
SLC25A37
SRSF2
ARMC1
AUNIP
BCL2L12
COLEC11
COX4I2
DCTN4
DDX59
FBXO25
GCOM1
IL17RE
L3MBTL2
MRPL2
NBEAL2
OSGEPL1
PACRG
PISD
PPP1R21
PYGO2
RER1
SAV1
SDF2
SGCE
SHISA3
SLC7A6OS
XKR8
ABCA8B
AP3B1
CAPSL
CCL13
D17H6S56E-5
ECI3
FAM160A2
GPSM3
PELI3
POLDIP3
RDH7
RIT2
SERPINA3G
TAF1C
TMEM82
TMUB1
ZBTB14
ZCCHC3
ARHGEF6
BLZF1
CADPS
CCDC88C
CD109
DYNC1I1
FAHD1
FAM102B
FAM89A
GRIP1
KCNG1

NAGLU
NUP37
PNO1
PRICKLE2
RAB40B
RASGRF1
RPL11
RPL29
SLC32A1
STON2
TTYH2
ZCCHC24
ACE2
ATP1A3
ITGB8
RBM39
TMEM37
ZBTB10
ACOT11
ARAP1
ARFGAP1
B3GAT2
CCNT1
CRYBG2
EOGT
GABARAPL2
GRPR
HS6ST1
ITPKC
LRP3
MAN2A2
MGAT3
NIF3L1
NKIRAS1
PEX3
PRKRA
RABL3
SLC16A13
SLC2A14
SNAP29
STT3B
SULT4A1
TBL3
TMED4
HLA-A
KCNA5
PCDH18
PDE9A
SHANK3
GAST
HLA-B
ALDH3B1
EGLN1
ARHGEF28
CHST3
CPA1
DESI2
DNASE1

FNDC1
GABPB1
GDE1
HERC2
IGSF1
IL12RB2
MIOX
MOB1A
MT1M
PDE8A
POU3F1
RPL41
SENP6
SKA2
SP4
TMEM45A
TRMT6
CPLX2
KCNK2
PIK3C2B
PRUNE2
SEMA3A
SFRP4
APOV1
LRIT1
RAB9
SKIV2L
BIVM
CAR13
CCDC88B
CDC42BPG
CDK17
CECR2
CHMP2B
DHX35
DOLPP1
DPH6
EPYC
FCF1
GALC
GPR157
GPR162
GUCY1B2
HAGHL
METTL6
MEX3D
MGRN1
MIR203
MIR31
MOSPD3
MRPL55
MYOZ1
NAA80
NAPSA
NEUROG2
PIKFYVE
POT1
PTCHD4

RNF34
SNX12
TAF6
TBC1D7
UBL5
UPF3A
ZFYVE19
AHCYL2
ARMCX1
CD99
CLCA2
COLGALT2
COPB1
CTTNBP2
DHX37
ERGIC1
FAM126A
FZD9
GJB5
GPR68
HAVCR2
HDAC10
HYLS1
IBTK
JPT2
LRRN1
MAGEE1
MARCHF7
MED6
MRPS26
NSMF
NUDCD1
RILPL1
RNF39
SCAMP2
SLC10A6
SLC35B2
SNAPC1
TEC
TREM1
TSHZ3
UBE2V2
WHAMM
CHST15
PLP1
PSPH
SP7
BTNL9
PRRG2
SCGB2A2
SPRR2G
ZNF32
TMEM189
CRH
GRIN2B
IRAK3
NRXN2
TNFSF12

BAZ1B
BEX2
CCDC6
DPP9
HIP1
IL20RB
ITGAE
MCU
MIPEP
SFTPA1
SLC30A3
SLC30A5
SLC9A3R2
SNU13
STK17A
TRIOBP
UGT2A1
WDR77
IL10RA
PIK3C3
TRPM7
ABCA12
AGPAT1
ATP6V1H
BNIP1
C1QTNF3
CALCOCO1
CELF4
CUL2
DAZL
FRMD3
ICOSLG
LRR1
MACO1
MED30
MELTF
OLIG2
OPHN1
P2RY12
PHF21A
POP5
RASSF8
RGS11
RNF115
RNF149
RORB
RPP25
RTN3
RUNDC3B
SGCD
SGSM3
SNAPIN
TCAF1
TPD52L2
TRIM23
VPS33A
WHRN
WNT10A

APOO
ATG4A
C1QTNF7
CD200R1
COL21A1
CYP2D26
DDX20
DNAH11
EFCAB2
FEZF2
GRAMD3
HS6ST3
KCNQ4
LGI3
LMAN2
LMX1A
MIR15A
MS4A6D
NR6A1
NSG2
NUP62CL
PHF7
POFUT2
RAB2B
RXYLT1
SLC25A39
ST8SIA6
TPSB2
WDFY2
ZCCHC9
BAP1
CDC42EP3
EYA1
ND1
NRIP3
SLC20A2
TIMP4
USP9X
BRWD1
CHRNE
EPB41L4B
HLA-C
KPNA1
MTARC2
OSR2
PDGFD
PPCS
SLC35F2
SOX8
TBL1X
TMEM30B
TNKS2
WNT7B
ZBTB21
MT1G
ARF2
COMMD7
CYP2J5

DKK4
DRG2
FBXW11
FH1
GIMAP1
H4C2
HEMGN
HSP27
KMT5C
METTL17
NUPL1
POLR3GL
SLC25A18
SPCS1
TMEM183A
UGT2B36
VRK3
ZBTB34
ZBTB45
ZFAND4
AFAP1
CYP2D13
DHODH
DHRS2
MYOD1
THEM5
ZSWIM3
KCNJ2
APEX2
ARFGEF3
ARPC5L
BCL7C
CCDC136
CCDC18
COL9A2
CORO2B
DLG2
DPY19L1
ELL3
FKBP9
GOLGA2
H2BC9
HRC
ING2
KANSL2
LDLRAP1
LINGO1
MACIR
METTL21A
METTL8
MYL3
NAIP
NOSTRIN
NSUN2
PMPCB
PPP4R2
PRMT6
RGS8

RNF44
SAP30L
SIRT7
SOCS6
SS18L2
ST6GALNAC5
UQCR10
WTAP
ZBTB38
ARNTL2
CDC42EP1
CLSTN1
COL8A2
COMMD1
CYP2C18
FNTB
ITPKB
OSR1
POLR1D
SF3B4
TNFSF13
VPS13A
YPEL1
ADCK5
CCDC127
CEP95
CHERP
CHST12
CLCC1
CLEC4A
CMTM4
COPS3
COPS8
ELAPOR1
ELMOD3
GALNT4
KATNB1
LZTR1
MED15
MIP
MIR10B
MIR20A
MIR212
MIR30D
MRPL11
MYOT
NIPA1
PDE6D
PHLPP2
PLEKHA4
PMF1
POLM
PPP1R3G
PQBP1
PWWP3B
REG4
RETNLG
SMARCAL1

SRA1
SWT1
THG1L
THSD1
TOX4
UGT2B35
COL16A1
CPM
NARS1
PPP2R1A
KRT7
TPO
ABR
APBA1
CNNM3
CYRIB
CYTH1
DACH1
DET1
DOP1B
EFNB3
EGFL7
ELMO2
ETV3
FASTKD2
GLRX2
GRAMD1C
HELZ
IFT27
LSM3
LUC7L3
MATN3
NOP53
PARP4
PHF11
PIEZO1
PNLIP
POLR3K
PTRH1
SAFB
SGPP2
SMPX
TRIP6
VIPR2
XPO4
INCA1
KLK1B27
ABL2
H1-2
SEMA5A
SYN2
ARMCX5
BICRAL
BRPF1
BRSK2
CABLES2
CAR1
CILP2

DARS
DNAI4
DPM2
EMC1
ENTR1
FAM72A
GALNT9
HSF2BP
MPND
MRPS35
PIP4P1
RNF180
RNF220
SCGN
SNX15
TSTD2
TTLL10
IL24
AFG3L2
ARHGAP4
B3GALNT2
B3GNT3
BBS7
BEND3
CACNA2D4
CASQ1
CHML
CRLF3
CTR9
CYB5R4
DCBLD1
EXOSC7
FAHD2A
GTF2A2
KPNA6
LCMT2
LDAH
LSM12
MEX3C
MMP23
MRPL34
MRPL39
MRPS16
NDUFB11
NEURL2
PMEL
PPM1M
PPP2R5B
PPWD1
PRSS16
SLC17A9
SOCS7
SPATA5
TIMM8A
TRPC5
UBA3
WDR44
YOD1

ZBTB8OS
ZNF703
ACVR2B
ATP6V1C1
GAR1
IL18R1
ITGAL
MLLT3
MYCL
PSMD3
TLCD1
ART1
ASIC5
SLC22A13
ZFP612
ARL5B
B4GALT2
B9D1
CAMSAP1
CDC37L1
CDON
CLDN6
DCAF8
FANCL
GATA5
GCC2
HCFC1R1
LHX8
MARCHF1
MOCS2
NEURL1B
OTX1
PLAGL2
RAB15
RHOH
RUFY3
RYK
SH2D5
SMARCB1
SOX7
SSR4
STOX2
TRIM54
UBE2G1
WBP1
ZFYVE21
ZRANB3
CCK
HTR2B
CA9
HOMER1
FLVCR2
ING3
JAM3
PCMTD1
PREP
STARD10
TMED9

WDR43
ANKRD29
APOBEC3C
ARFIP2
ARL6IP4
C1D
CLEC3B
CNPY4
CNRIP1
COASY
CPA3
DAAM2
DDX54
DGKE
DIPK1B
DOLK
DPY19L3
ELAVL3
EXOC1
FAP
FGD3
GOLM2
KCNE4
KIF12
KLHDC8B
LANCL2
LEFTY1
LYPLA2
MIR23A
MRPS17
NEK9
PALM
PAX3
PNCK
PROK1
RASIP1
RBM18
RNF5
RPP25L
RRP7A
SIX5
SLC38A9
SLC6A18
SPSB3
STYK1
SYT3
TNFRSF8
TRMU
UBLCP1
DUS3L
HSDL1
MIR429
PKD2L1
PLAC9
RAB40C
SERPINB10
SLC39A2
TGOLN1

TLL2
TMEM104
TMX2
TUSC2
GAP43
CLASP2
COPS4
DLG5
FOXK2
GPSM1
H2AC20
H3C4
JAZF1
KCMF1
LGALS7
MCRIP2
MIR141
MIR221
MREG
PAPLN
PDXP
POU2AF1
PTPN22
SEC61B
SPAG1
STAG1
UBAP1
UPF2
UST
ARRB2
PVALB
HMGB3
NOP58
C1QTNF6
COL4A3
ITM2B
PAG1
PIK3R2
POLH
RBM33
RCN3
CLDN5
TNS3
AQR
BCAN
BMERB1
BTF3L4
CCDC9
CEP78
CHMP2A
CNIH4
CNOT2
CTLA2B
CYP4F6
DEF8
DYRK1B
FAM3B
FBXO4

INSC
LDB1
MCTS1
MMP24
OAS1F
PAIP2
PCDHGC3
POLR1E
PSD2
RBM6
SLC10A7
SLC35E2B
SLC66A2
SREK1IP1
TENM1
TMEM139
TMEM143
TMEM164
TRP53BP2
URB1
VPS8
ZFP9
ZNF148
ZNF438
ZNF827
CAMK1
CCDC85A
CLCN2
DPH5
ESRP1
EXPH5
GJA3
GPR176
HLA-G
KDM2B
LCOR
MAT2B
NUCB1
PANK3
PRR15
RALGAPA1
SECISBP2L
STYXL1
SYNJ1
TAF13
TEX264
TMEM204
TRPC1
UNC13D
VAPB
FOXO3A
MUP9
CGA
ADCYAP1
CCL24
HRK
KCND3
KRT80

PHTF2
PKD2
PRR5L
RNF213
TNRC6A
AGPAT9
ARHGEF10L
CASTOR1
COMMD8
CST7
DNAAF5
DPH1
FUNDC1
GFOD2
MKRN2
MRPS15
MRPS9
MYT1L
NHEJ1
NRSN1
OSCAR
PARP6
PCNX3
PPCDC
RCC1L
RHBDD3
RNF167
SCYL3
SEMA6C
SUPT6H
TLX2
USP54
ZDHHC7
LAMA4
MOCOS
ACSS3
ADCY4
BEST1
DCPS
EPC1
GNB4
GRM3
HEYL
MAP3K2
OAZ2
PARP8
PI4KA
SEC16B
SLC30A4
TRIL
VAMP4
ACSL6
ADGRA2
ADNP
ANKRD2
ARID5A
ARL2BP
BTBD10

CEP63
CHST7
CLDN15
DEGS2
GATAD2B
GDI2
HAUS7
ICAM3
IL22RA1
IQCK
LIN28A
LPCAT4
MIR29A
MTPAP
NODAL
PLRG1
PNOC
PPP4R1
RABGAP1
ROMO1
SETMAR
SH2D3C
SLC2A10
SMYD4
STMN4
TRIM26
UBE2D1
UQCRH
USP32
WDR6
YIPF3
ZFP90
ZNHIT6
PTH
TUBA1B
OXT
S100B
NR0B1
MUCL1
ASB14
ASB18
CAR5A
CHCHD4
COL20A1
COPS6
CYP26C1
DTX2
FAF2
FAM25C
GP2
GPATCH3
HOXA7
HOXB4
KLF17
KLK11
LAPTM4A
LIPT1
LIX1

MFSD13A
MLIP
MORC2
NAA35
NEU3
NT5M
PARS2
PSMD10
RNF216
SECTM1B
SENP3
SERPINB4
SPAG16
SRMS
STOML1
TGM5
TMCO4
TRIM44
UBE2D4
UFC1
UGT3A2
ULBP2
WDR55
WDR70
ZFP106
ZGPAT
AFDN
AKR1C19
AMDHD2
CAND1
CCKAR
EID3
FAM189A2
FILIP1
GLIS3
HPDL
HSF2
ISL1
KIF26B
LIPH
MARK3
MGAT1
MSMB
MTFR2
NECAB1
NR2C2
PITPNB
PLPPR1
SERPINB6
SLC30A7
SNHG1
SNRNP25
SNX29
THBS3
TNFSF13B
UBR2
ZCCHC2
BPTF

CACNB2
COL13A1
GALNT6
KCNJ5
LRRN3
SLC11A1
WFS1
CYP51A1
GCG
NUP210
PLIN1
ARHGAP21
B4GALT7
CRTAC1
DMPK
EFR3A
FAU
GPR35
HEMK1
ICOS
LETMD1
MARS2
MIR144
MRPS12
NEK1
NT5DC3
PACS1
POGLUT1
POPDC3
PPAP2A
RILPL2
RNF150
ROR2
RPL7L1
SH3RF2
TCAIM
TMEM256
TSC22D4
UBE3C
VILL
WNK2
WRN
ZBTB7B
ISOC2B
PFN4
TECTB
MOV10
RAD17
RB1CC1
S1PR2
SUV39H1
TRIM14
CTCFLOS
NEFM
BIRC6
CCDC77
CRBN
DTX3L

EPHA5
ERG
FOXG1
GABRE
HAS1
HES2
KIF13A
MTPN
ORAI1
PAK6
RDM1
RPL26
SBSPON
SLC44A2
SRSF6
TNFAIP1
TSEN2
SOS1
CNR2
CCL22
ADPRHL1
AP4M1
APH1A
ASPDH
CREG2
DHRS13
DHX16
DNAJC28
DOK7
FAM118A
FAM160B1
FAM234B
FBXW2
GZMK
IFNLR1
IFT46
KLHDC10
KLHL31
LARS2
MARK4
MED28
MIR126
MIR503
NCKIPSD
NECAP2
NIPSNAP3A
NOTUM
NTMT1
OTUD6B
PARP11
PODN
RALY
RBMX2
RCCD1
RGS7BP
RPH3A
SLC36A2
SPATA17

STRADB
TBC1D19
TENT2
TLCD2
ZBTB7C
ZFYVE27
ABHD17B
APOC4
BMS1
CAPN10
CCDC141
CEP164
COG4
CRTC3
DHPS
EMD
GCC1
HELQ
HSD3B
HYAL2
KBTBD2
MIR143
MIR146B
MOB4
NPY2R
POLR2I
PTK7
QK
RNF207
RNF38
RSBN1
SSU72
TOM1
TRHDE
TXNDC17
UBALD2
VPS33B
ACVRL1
BMPR1B
ELOVL7
EOMES
HDAC7
RCC1
SLC25A3
TMC6
BTN3A3
CIPC
CYP4A12B
HHATL
KLHL32
LENG1
MED25
OAZ3
PCDHB2
RASL2-9
STFA2L1
TECTA
ZFP280D

ASIC2
FOXD1
GPD1L
ITPRIP
LRRK1
LSM5
MCFD2
N4BP1
NCAPD3
PCDH8
RBFOX3
UIMC1
WDR45
YTHDC1
NCOR2
RPS6KA5
ACAN
ASCL1
MAGI2
MED13L
ZC3HAV1
AMN
CCL25
CENPS
CEP290
CEP41
CGGBP1
COPZ1
DMP1
DPF1
ENSA
FAM133B
FANCB
GPR87
GXYLT1
HPRT
HSD17B14
IFT20
KCNC1
KCTD10
LYPD6
MBOAT7
MED14
NCKAP5
NDUFB1
NUP35
RAD1
RPL35
RUSC2
SAMD5
SH3RF3
SOCS4
TMC4
TMEM177
TRIM8
TSGA10
UBE2Z
UBR5

UBXN1
UGT2B5
UHRF1BP1L
VPS26A
XPNPEP3
ZMPSTE24
BCAS3
CDK19
CTSV
DNAJC7
DOT1L
EME1
ENPP5
GABPA
MCOLN3
MYBPC3
MYO7A
NOL6
QPRT
SFMBT2
TMEM38B
ZHX2
ZIC1
ADNP2
AHSP
AKR1C12
ARHGDIG
C5AR2
CEP112
CMC1
COL26A1
DNAJC22
DOHH
ENTPD8
GTF3C4
HS3ST6
IFI203
INSL6
KIF9
MEAF6
MIR494
MTCL1
MYSM1
NUMBL
OSGEP
PIGS
PLEKHG6
RNF168
SDK1
SEC31B
SLC35A2
SLC35B4
SLC6A17
SMU1
SOX1
SRBD1
SYPL2
SYT8

TBCA
TEAD3
TSPAN11
TUBGCP5
ZC3H14
GRM5
ADRA2C
FERMT1
RBM14
SEPTIN8
TRPC6
BXDC2
FBP1B
FSCN3
KPTN
PPP1R3F
WFDC15B
ZFP707
ADRA2A
HSD17B1
ANKRD49
B4GALT3
BCDIN3D
CTDNEP1
DDX19B
DUOXA1
EML3
FABP12
FADS6
FAM114A2
HAUS5
IGHG
IRF2BP1
KBTBD12
LHFPL4
MFAP3
NDOR1
PAKAP
PAX4
PM20D1
PVRL1
SCAMP3
SLC6A3
SNURF
SULT1C2A
SZT2
TMEM175
YIPF2
FOXC1
SSTR2
VIP
CLN3
COL10A1
EIF4G3
IMMT
KLK6
NOP2
PEX13

PRSS29
PXDC1
SEMA4G
TMEM19
TP53INP2
UNC5C
WNT1
ATP11B
BRI3
CANT1
CEBPE
CPSF2
DMRT1
EXOC2
GNPDA2
HNF4G
ISL2
LGI1
LRRC40
MRTFB
MYBPH
NAPRT
NTNG1
PAOX
PHLPP1
PLEKHB2
PLEKHM1
PPP4R4
PRPF39
RNFT2
RPH3AL
SEMA4C
SEZ6L2
STON1
TBC1D2B
TMSB15A
USF2
WASF3
ZFX
ADCY3
ATP8A1
CSF2RA
MCC
MCTP1
SEMA4D
AOC2
ARFRP1
ATP5MF
BAHCC1
BCORL1
CEP250
COA3
CPSF3
CYSLTR2
DCLRE1A
DIMT1
DLK2
EMC3

GEMIN8
GIMAP6
H2AC4
H2-Q10
H4C14
KLRD1
MIPOL1
NSUN6
NT5C
PCID2
PGLYRP2
PI3
RANBP10
SIGLEC5
TGTP1
TLCD5
TMEM120B
TMEM168
TMEM60
TOE1
TRIM39
USF1
PDE10A
ANO6
FAIM2
GTF2F1
HLA-DMA
HNRNPH3
JADE2
KCNJ12
MSRB3
RCOR3
RICTOR
RO60
SLC2A8
ATF1
GABRA4
NEUROD1
POLQ
AGO1
AKIRIN2
ARHGEF18
CDYL
CLEC14A
CNOT6L
CSNK1G3
CYP2A2
DUSP26
FAM117B
FAM13B
FAM167A
GIT2
IYD
MINPP1
NUB1
PACSIN1
PLCXD1
POLR2C

PYCR2
RCSD1
REPIN1
RT1-DA
SDF4
SERAC1
SGTA
STK40
TP53I11
TWSG1
PAPPA
PIK3CG
REN
ADAM32
ALG11
APCDD1L
CEP97
DCAF12L2
FOXD2
GHRHR
GPANK1
HOXD4
MIR330
NPRL2
NUDT22
NUDT8
PEA15A
PECI
PIGB
RBSN
RTF1
SCD3
STAG3
TANGO2
TBC1D10B
THAP3
TRIM7
ALK
CCL1
PTPRZ1
CDH8
DUSP7
GNL3L
H2BC4
IGSF9
KANK2
MYOCD
NOD2
NPM3
SEPTIN6
STING1
UFM1
ZBTB4
ZSWIM6
ADARB2
ARHGEF25
BBS1
BMP15

C11ORF54
DDX28
FAM20B
GGT7
GNG3
HGH1
IPO11
MCTP2
MFSD11
MRI1
MTX2
NEUROG1
NKX6-1
PIGZ
PITPNM2
POFUT1
PRDM4
PRPSAP2
RAB11FIP5
RANBP6
RSPO1
SGIP1
SLC25A17
SLC35F3
SLC45A2
TMEM161A
TNFRSF18
TNPO2
TSPAN9
UGT2B15
USP22
VEZT
ZFP423
ANKRD23
CAPN11
DDR2
FIS1
HSP70
LMAN1L
SERPINB12
TEX12
TRP53BP1
APTX
ASCL2
ATP5F1E
CAMSAP2
CBLN1
CCDC69
CD19
CD5
CNPY2
DNPEP
FABP10A
FAXDC2
KIRREL3
MIR132
MMS22L
NDP

PAQR5
PDAP1
PLA2G12B
RHOF
RIMS2
SFT2D2
SIX4
SLC7A10
SNX4
SPATA18
TAMALIN
TOMM40L
TRAPPC9
TSPAN31
UBA2
ATP6
RPS10
SLC17A7
SLCO4C1
CCNT2
CRLF1
LMO2
OLA1
OSER1
RBM12
SDC3
SOAT2
TRPC3
B4GAT1
CCDC59
CCR9
CNPY3
COMMD5
COMMD6
DCTN5
FAM110A
FKRP
GABRG3
GARS
GLRX5
GPR153
GRAMD4
GTF2H4
HECTD4
INTS3
IRGM1
LPIN3
MTMR2
NAGPA
NIPAL1
NRROS
P4HA3
PDE6C
PGGHG
PIGM
PLBD2
RABIF
RAET1B

RIPOR1
SLC25A14
SMARCD1
SNX14
SRFBP1
STK25
TAF3
TAL1
TASP1
TMEM132D
TMEM41A
TRMT11
TRUB1
UNCX
ZMYND12
AKAP7
ARHGAP28
ATL1
CENPL
CPNE4
CXCL15
CXCR3
DTWD1
FAM53B
FCGBP
HOXB2
IGDCC3
IMP3
INPP4A
KRR1
LANCL1
MC1R
MED20
MIR99A
MMGT1
MRPS18C
NAA10
PRPF38B
PRRG1
REC8
SLC15A1
SRSF4
STRIP2
STXBP1
TRPM2
VTCN1
ZADH2
ZKSCAN1
AFG3L1
ANO7
CBX8
CCDC22
COMMD2
CYB561A3
DALRD3
DDX49
EEFSEC
EMC4

GMEB2
GTF2E1
INO80
INTS1
ISCA2
JTB
MIR455
NOM1
P2RY13
ROM1
SS18L1
SVBP
SWI5
TBC1D13
TMED6
TRMT112
UGT2B11
ZMAT5
ZSCAN21
ABCG2A
LINCR
MIR301A
ZFP335
CA4
GDI1
H4C8
HAPLN1
IRX1
PNPLA7
SREK1
TBCD
TIGAR
DRD2
NSF
GALR3
MIR181D
MIR708
MS4A4D
TMEM178B
TTC7
ZNF574
A4GALT
ABCD1
ADAMTSL2
ARCN1
ARFGEF1
CCNB1IP1
CCNY
CDH23
CLDN10
DCTN1
EIF3D
ELAC2
FBXO11
GFRA3
GPRIN3
H1-3
H3C1

IL9
MIA3
MLXIP
PADI4
PLXNA3
PTPRH
RNF114
SLC25A16
TP53BP2
USP24
ATP5H
EPHA7
FKTN
INSL3
LPAR3
MIS18A
NPPC
SLC13A4
SPATS2L
TFEB
WNT7A
ANGPT4
ASPSCR1
BRF2
CCDC93
CD7
CDC40
CDK5RAP1
CLNS1A
CNIH1
CPN2
CUTA
DACT3
EIF3G
EMID1
FHOD1
GALNT14
HOXC9
HVCN1
KANSL1L
KCNQ1OT1
LHX4
LSG1
LY6G6C
MAB21L1
MIRLET7B
MRPS36
NGDN
NPAS3
OLAH
PAQR3
PBDC1
PELP1
POP7
PURG
RAB7
RBM7
SLC24A2

SLC39A13
SLC66A3
SMIM1
SPSB2
SYCE2
TMBIM6
TMEM160
TNNI1
TPSAB1
WDR7
LNPEP
OSM
ASB10
ATG4D
ATP5O
CCDC106
H3F3A
MAST1
MYL10
MYO18B
NAT9
PIANP
PIGY
RANBP3L
REM1
RING1
RTL8A
TLE6
TMED8
ULK3
ZNF189
ADCYAP1R1
FGGY
FHOD3
NACA
PITX2
RTN1
SLC29A2
ACAD10
ANKFY1
AP1S3
CENPT
CHRNA1
CTPS1
DDX3Y
DGCR8
DPP6
ECM2
GIP
HOXD1
ITGBL1
KDM4A
MAPRE2
MGAM
MIR125A
NIPAL3
PEX6
PHC3

PSMG1
RAP2A
SELENOK
SLC19A3
SPIN4
TASOR
UGT8
VPS45
WSCD1
DIDO1
KIF1A
SRD5A2
ACKR2
AHDC1
AICDA
APOL1
ARID3B
ATP5L
CDC23
CDH7
CNOT8
COA7
COPS7A
COQ2
ENOX1
FOXN4
GPR180
GTF3C6
IL11RA1
INO80C
INPP5J
ITFG2
KCNC4
MDFI
MICU3
MMP28
N6AMT1
NOS2A
NOSIP
NUBP2
OCIAD1
PKN3
PLEKHA7
PLXNA4
PPFIA3
PPT2
PTOV1
RAB1B
RFXANK
RNF187
SDR42E1
SENP5
SERF2
SHBG
SHPRH
SNAI3
SPOCD1
TLNRD1

TMEM51
TSPYL1
ZC3H7A
APEH
CNKSR2
HLA-DQB1
MBOAT1
OPRL1
SFXN3
SLIT1
LONP1
ADGRL4
ARGLU1
ARHGAP19
CDC42BPB
CLK3
CTSO
ELF2
FAM13C
GYPA
MRPS27
MYBPC1
PLCL2
RALB
RBKS
REXO2
RHBDD2
SLC7A9
STX1B
THEM6
TRDN
ZBTB44
ASB8
CYP2C67
FAM167B
MOV10L1
OTOP1
PCDHB5
RTL8C
S100Z
TREML4
VAX2
VPS9D1
ZNF552
ADGB
AKR1E1
MUP21
NOTCH1A
RTF2
ABCC12
ATP5F1C
BAHD1
COX14
CRIP3
DANCR
DUS1L
DXO
FAM118B

FBRSL1
GLRA3
GZF1
KCND1
KHDRBS2
LIPF
LRTM2
MRPL21
PARD6A
PIGV
PLPPR2
PRAF2
RECQL5
RT1-BA
RUSC1
RWDD3
SIRPB1
TXNDC12
UBE3B
UNC93A
WDR48
ZFP395
CAVIN1
IRF2BP2
ADAMTS6
AGBL2
ALG8
ARL2
CADM4
CCT4
CD248
CELSR2
DGKD
DNASE1L1
DRAM2
DTX3
DUSP22
EDIL3
ERGIC3
FAM210B
FBXO33
FLOT2
FMNL1
GNG12
GRSF1
GSC
L3HYPDH
LMBRD2
LONRF1
MCPH1
MEGF8
MFSD10
MIR15B
MOS
MYOZ2
NEIL2
NFXL1
NOTCH4

PEX2
PHF2
PLD6
POLRMT
PPFIA1
PPP1R12B
PRKRIP1
RIOK1
RNASEH2C
RNLS
SCRIB
SLC9A6
SYF2
TBC1D2
TMEM150C
TUBB1
UBE2L3
USP46
WDR35
ZNRF3
ZRSR2
AEBP2
ATP2C1
BBS9
BNIP2
CTBS
FBXL5
HERC4
ITPRIPL2
ITSN2
LAMTOR3
MALL
MSRB1
MST1R
N4BP2
NOP16
NPEPL1
PDE4C
PHYHD1
PPP4R3B
RAB3C
SFXN5
SIRT4
SLC9A7
TRPM8
VPS37B
WDR46
WDR72
TNFSF4
CALB1
CCL17
FIBIN
MYOG
RPLP1
RPS12
ABCB5
ACSF3
ANKRD16

APOBEC3G
ARHGAP27
ATP1A4
CAPN9
CCDC124
CCDC91
CLK2
CRB3
CSDC2
CSNK1G2
CSRNP2
CWC25
CYP2C68
CYP2J4
DCP2
DMAP1
EN1
F13B
FCRLA
FGF17
FTSJ1
GDPD2
GPR65
HSBP1L1
IFT81
IQCD
MAGIX
MAZ
MED11
MINDY3
MIR342
MLANA
MPHOSPH8
MPV17
MRPL54
MS4A6C
OLIG3
OSBPL9
PADI1
PIGW
POMGNT2
PRR7
PWWP2B
RAB12
RAET1C
RGL3
RIOX1
RNASE2
RNASE6
RPAIN
RPRD1B
SF3B2
SHTN1
SPHKAP
SPRR3
TMEM131L
TMEM167A
TMEM184B

TMEM50A
TMEM59
USP19
USP45
WDR32
WDSUB1
ZC3H15
ZMYM4
ZNF503
AKR1C4
CALM3
ANKHD1
ARHGAP17
ATP6V1G1
BPNT1
DCX
EVI5
FNDC4
GKAP1
GPR183
ING1
L2HGDH
MCUB
NAPG
NRM
POLR2F
SPIRE1
SV2B
VWA5A
ZNF652
ERAL1
EVX1
HHIPL1
KCNK15
PDILT
SAMD4
TRIM68
TULP2
ZFP276
ZKSCAN8
CLMP
ELK4
OBSL1
PEG3
RGS9
SFTPC
JMJD1C
ANKRD9
C1GALT1
EIF5A2
GABRA3
GALNS
GDF6
HAX1
IRX4
KCNJ4
KCTD5
MBTD1

METRN
MFSD4A
NCS1
NPLOC4
NUP205
RIMS1
SALL2
SETX
TAF4B
TLK1
ACBD7
ARHGAP12
BCAS2
BRWD3
COIL
CYP4F4
DDX50
GNAT2
GPX1A
H2BC6
IARS2
KIF13B
LCT
LMAN2L
LRCH1
MAPK15
MRPL15
MRPL51
MYDGF
NDST2
NOL9
NSMCE1
PDXDC1
PI16
RASSF9
RPRD1A
RPS6KA4
SIPA1L3
SLITRK5
SNAPC5
SSBP4
SYNGR2
TMEM125
FBL
RAI1
RECQL4
TMEM106B
KCNH2
ACPP
BRDT
CCL15
NUPL2
RARS
TBPL2
CHGA
ADSS2
ARMC10
ARMCX4

B3GAT3
BBS5
C1ORF116
CTSW
CYP4F5
FMNL3
GDAP2
H2BC10
HINFP
IFT22
KY
MAP1S
MDGA2
MIR184
MKX
NCR1
NPY5R
PDCL
PRPF40B
PYGO1
RPUSD3
SCGB3A1
SH3BP1
SLC24A1
SMG6
TBC1D12
WNT9B
ZDHHC5
ZNF143
HTR1B
CHRNB2
AMH
ABCA8
ASH1L
CD46
DENND5A
ENY2
EPHB3
FBXL4
HSD17B8
INPPL1
LENG8
MOSPD1
OPCML
PHF1
PHTF1
RASEF
RPS26
RPS29
SLC12A6
XPR1
GRIA2
ABRAXAS2
ARXES2
ATP5K
B3GNT8
CCDC82
CDC42SE2

CEP83
COA6
COL22A1
COL9A3
CRNKL1
DGKB
DHX36
DRAP1
EFNA4
FAM178B
FAM78A
FBXL15
FGD5
GJC3
H4F16
HHIPL2
HOXC6
HSPA14
IMMP1L
ISM1
LY6G
MRPL14
MRPS22
MSL2
NALCN
NDEL1
NSMCE3
NTN3
NXPE4
PEX12
PNLIPRP2
PRDM8
RBFOX1
REXO4
RHBDL2
SCAF8
SERPINA1E
SLC26A3
SLC35E4
SLC4A8
SLFN4
THAP1
THOC3
TIMM22
TMEM238
TSFM
TUT7
UBIAD1
UROS
UTP14A
WDR83OS
WWP2
CYTB
ABHD16A
ANAPC13
AP5Z1
ARL11
ASCC2

ATP9B
CFAP251
CYS1
DNAJC13
FBXL12
GAB3
HNRNPUL2
ILKAP
IRGM2
LRRC45
MBIP
MIEF2
MPP4
MS4A6B
MTIF3
MTX1
NAA30
NOL12
NYNRIN
PDZD11
PRDM13
RARS2
RP9
SEC14L3
SLC27A6
SLFN2
SOX14
SRPK3
TAF10
TAFA2
TMA16
TMEM68
TUBG2
YIPF6
ZDHHC18
ZNF22
ZNF473
AP3D1
CPA4
DOCK11
F9
NMNAT1
RAB17
SSR2
TENT5B
AIP
AMDHD1
AMMECR1L
AMZ2
ATP5A1
B3GALT5
B3GNT7
CCL28
CDH10
CORO1B
DHRS11
DOCK6
ENOSF1

FMC1
FOXK1
GPIHBP1
GTF3C1
HCRTR1
ITFG1
KCNJ13
LPAR4
MAML2
MIF4GD
MIS12
MRPS31
MYPN
NMD3
NTPCR
NUP85
NXPE3
PTPN7
RAB21
SCUBE1
SIGIRR
SLC26A11
SLC35A3
SLC5A11
STAP2
STRADA
THAP11
THUMPD3
TMEM154
TYSND1
WWC3
YIF1A
HLA-DRB1
PLD2
RBM25
SETBP1
EMILIN1
GEMIN4
HECW2
KCNS3
LRRC17
MGARP
RIMS3
SNX5
STXBP2
TTC28
U2AF1
UGT2B4
XPO5
LRIT2
SECTM1A
SNORA74A
VPS24
ACOT6
AFG1L
AP4B1
BPIFB1
CISD3

CLBA1
CNOT10
CPSF4L
EDC4
FOXL1
HLA-DRB5
ISOC2
KLHL20
LRCH3
METTL26
MINDY4
PSKH1
RWDD4A
SNX22
SVOP
MIR181A2
DCC
HSPB11
ITGB1BP3
LCA5L
LRPPRC
SCARNA6
SLC1A6
TARS1
WDR21
ZFP54
ADPRH
ASB4
BTN3A2
COX20
DCTN2
DLX6
ECSIT
FAM219A
FUT2
GIT1
GNPTG
H2AC13
HOXB13
KLC3
KLHL18
KRT75
LGI2
LNPK
MATN1
MCM3AP
MTMR12
NOVA2
OLFM3
PEAK1
PHF23
PLSCR3
RPP30
SFSWAP
SLC26A10
SPNS1
SUCO
TAB1

TMCO3
WDR37
XKRX
ZCCHC10
ADGRB2
AKAP10
ALS2CL
ARHGAP9
BABAM2
CCL27
CLINT1
DAZAP2
DOCK1
DOK3
GEMIN5
GJB6
GXYLT2
HEATR3
IQCG
KLHL23
MEP1B
MPP5
MYO1G
NAT10
PAX2
PCYOX1L
PDSS2
PHC1
PLPP6
RFLNA
RT1-BB
SAE1
SARNP
SLAMF7
STX6
TMEM54
UBE2V1
XIRP1
ZFC3H1
CRHR1
VTG1
ACVR1C
BATF3
CHIC2
H1-10
HS3ST3B1
SH3BGR
TCF25
PPP3R1
ADAT2
ANKRD50
ASB1
CDC26
DCHS1
DYNC1I2
EDEM3
ESCO1
HCN2

HSBP1
IL17RD
INTU
KCNK10
LMBR1
LRRC8A
LY75
MFAP3L
MTHFS
MTURN
NDUFAF1
NUDT16L1
PSCA
PTGES3
RAB11FIP4
RFX7
SCML1
SRP68
TMEM67
TWF1
UGT2B10
VAC14
XYLB
ACSM2A
ADAMTS8
ANKLE2
AP4E1
ARHGAP30
ATP6V1B1
ATP6V1G2
AVL9
C3ORF52
CAMTA2
CARF
CCDC71L
CRB2
CSTF2T
DCAF4
DNASE2B
ELAVL4
FEM1A
FRAT2
FUNDC2
GATB
GYG2
HENMT1
KCTD11
KLK2
MBD6
MDP1
MEA1
MLST8
MTHFSD
PLEKHA8
PTDSS2
RAB5IF
RIMKLA
RNPEPL1

RPRD2
SCAF1
SCARF1
SLC37A3
SRSF9
STK16
STX5
SUN1
THOC6
TMEM267
TNFAIP8L3
TTC8
UHRF2
VPS72
WDR59
ZSWIM4
GNRH1
MID1
ACBD6
CFAP74
CPLX3
CUL9
CYP2D10
CYP4F22
FOXN1
GIMAP7
LYRM4
NCCRP1
NT5C3
RLBP1
RPAP2
RPP14
RPUSD1
SIAH1A
SIT1
SLC25A48
SLC38A11
TAS1R1
TEX11
TMEM127
TSPYL5
UTF1
UVSSA
VPS26C
ZFP68
ZNF580
OPRM1
ELOC
IRAK4
KCNE1
MMAB
ND2
SCN2A
TIRAP
ANKMY1
CNGA3
CYP2D12
LCN8

MIR106A
MIR702
NKX2-6
TMEM254A
XLKD1
ZNF70
CLDN11
GRIN2C
HTR7
ADCY10
ALDH16A1
ARHGAP35
B3GAT1
BEND5
BLOC1S6
CACNG6
CALCR
CASP14
CDKN2AIP
CLPB
CNOT7
CPNE5
D2HGDH
DDX11
EIF3E
ENPP4
EXOSC9
GPR88
ICE2
LTB4R
NECAB2
PDS5A
PEX1
PGC
PHF3
PHKA1
PIGN
PLCXD2
RIOX2
RTTN
SGCG
SHC3
SRGAP1
TBX15
TNS4
TOMM7
VPS53
ALG6
ASB5
ATP5E
CHST9
CNIH3
CNOT9
COG7
CPA5
CRISPLD1
CSN3
DAZAP1

DCUN1D5
EID2
FN3KRP
GMIP
HEATR6
JCAD
KCNF1
KLHDC3
LMTK2
MIR196B
MTCH1
MTMR9
MTO1
NKX6-2
NOC4L
PCED1B
PCSK7
PDPR
PNMT
POLR3F
RAD51D
RGS13
RNF126
SERPINB6B
SLC35F1
SNRNP27
SORCS1
TMCO6
TOMM22
TRAPPC2L
TTC13
VAMP3
VPS4A
WDR31
ZMYND19
ZNF593
AHNAK2
BRD8
FAT4
FGF5
LIMS2
NAP1L5
SULT1A3
TMEM50B
ACKR1
ANKRD42
ASXL2
B3GALT4
CCDC57
CILK1
COPG2
CRISP2
DAB2IP
ENAM
ENTPD4
EVI5L
FAM83G
FBXO44

FUT10
GGA1
GRM8
HASPIN
HTRA4
IGLL1
INPP5K
KRT71
LRFN4
LSM11
MOG
MS4A4A
NCBP1
NEUROG3
NHSL2
NSMAF
OSTF1
PCDH19
POC1B
PPP4R3A
RABL2A
RFX1
RNF208
ROBO4
RPS23
SEC24A
SELENON
STX16
SYNGR3
TACO1
TBC1D22B
TMEM234
UNC5A
UTS2R
VANGL2
XIST
ZBTB18
ZCCHC12
ROBO2
ARF4
ARL1
COX8B
KDM6A
MUC5B
NTSR1
SMCHD1
TESC
ACKR4
ACVR1B
AP3B2
ASNSD1
ASPHD1
CCDC28A
CCHCR1
CTNNA3
DCTN3
DDX31
DDX4

EDRF1
FAM161A
FAM81A
GPKOW
GPR135
HCP5
HOXA4
KCNMB1
KCTD13
LOXL3
MAML3
MARCHF8
MED7
MIR98
MRPS11
NFU1
NOMO1
PEF1
PGBD5
PHAX
POLL
RAB11FIP2
RDH14
RFXAP
SCML4
SERPINA1A
SH3D21
SMCR8
SNX17
SNX18
SORCS3
SRP54
SSTR4
STK32C
SYN3
TCTA
TDRP
TTC26
YDJC
ZBTB17
CNTN1
TNIK
BCL9L
CEP350
CREB3L4
DPP8
EPX
FAM168A
FHL3
FZR1
GPR39
LAMC3
LMO3
MIR27B
PAK4
PDE12
PEX7
PRTFDC1

RABEP1
RBM38
SLC36A1
SLU7
TBC1D16
TIMM44
C2ORF76
CHADL
CHES1
DCUN1D2
KIAA1522
LDOC1
NKX1-2
PMFBP1
RELCH
SARS
STFA2
UPK2
VAX1
ZFP110
NONO
RPL21
DRD1
CACNG5
CFAP36
CHST13
DNASE1L2
DNASE2A
ER
GCNT4
IFFO1
INSM2
LAMP5
LINC00261
MIR152
MUP4
PBX4
RHBDL1
RSPRY1
SHF
SLC25A40
SNUPN
TANGO6
TFPT
TRARG1
TRMT10B
UNC119B
ZNF507
ASTN1
ATG4C
BLOC1S1
C15ORF39
CACNA1S
CBFA2T2
CCNJL
CHIT1
COG6
DNAAF1

DNAH2
DNAJB13
ELMO3
ELMOD2
FAM149B1
FAM32A
FAT2
FDX2
FUT11
GIN1
GPATCH8
GPI1
HBA-A2
IL20RA
INTS2
IRS3
JKAMP
KEL
KHNYN
LMF2
MAFA
MAP3K7CL
MRPS10
MYLK2
NT5DC1
NUP43
PPP1R7
PTPN4
RAB3GAP1
RARS1
RNF130
RWDD2A
SCAI
SELENOF
SIRT6
SLC6A20
TAGAP
TMEM170B
TRUB2
TUBA3A
UBXN7
WARS2
WBP1L
ZBTB22
ZNRD2
ZSWIM5
ZZEF1
BRD4
ARHGAP15
ARID3A
BTK
CDK10
CDK13
COX7A2L
GNPAT
GSDMD
IAPP
KAT2A

MVB12B
NAPB
PHLDB3
PRKD2
SHROOM2
SLC35A1
SLC35E1
TAOK2
TDGF1
TXNDC5
ZIC3
CD3G
DRD5
GNB2
IL22
KCNMB4
SLC17A5
SUGCT
GRM1
MAPK12
CCNI
EPHA3
SUB1
TGOLN2
UBE2J1
AAK1
ACOX3
CLN5
CMKLR1
DISP1
FGF15
KIF3C
MYCT1
POLG
PPP1R1C
RAB3IL1
RAG1
SENP1
ATP5MG
AURKC
B3GALT6
CALHM2
CUEDC2
DDX41
DNAH5
DOK6
EFCAB7
EIF4A3
FCGR1A
GPR27
GPX5
HAPLN4
HEXIM2
IL5RA
INPP5E
KCNK12
MDH1B
MIR204

MIR20B
MIR25
MYL6B
NBEA
NEMF
ORAI2
ORC3
PDZD8
PHOSPHO1
PIP4K2C
PKD1L2
PLGRKT
POGZ
PPP6R3
PROM2
RAPGEFL1
SAFB2
SLC9A9
SNX13
SPAG6
SSTR5
SYT5
TAPBPL
TCF20
TLCD3A
TMEM156
USP21
ZNF704
ATP6V1E2
BCL7B
BMP8A
C1QL1
CLPS
CLRN3
CRACDL
CYP3A41A
FCSK
GSG1
H3-3A
HAUS3
HOXC4
INO80B
KCTD2
KDM3B
LMOD3
LRRC51
METTL15
MIR133B
NPIPA1
OTUD3
POC5
RAET1E
RBM45
SDR39U1
SNAP47
TDP2
TGM4
TMEM132C

TSEN34
ACCS
ATP5G2
CD209F
CD59A
CRAMP1
CYP4F13
EAR2
HIF1AL
IFI205
KLK9
LY6G6E
NMUR2
POU6F1
SRRD
ZDHHC19
ZFP523
ZFP750
LRRK2
BTNL8
CCDC84
CD101
CTRB1
GBP11
HOXD12
HSF5
KRT32
METTL2
MRC2
N4BP2L2
NOL1
SLC2A12
SPN
TBC1D15
ZFP511
ARHGEF10
ATP6V1C2
BSN
CACNB4
CD247
COMP
GPBAR1
H2BC8
IP6K1
KCNIP4
KMT2E
MAN1C1
MIR192
MPV17L
NLGN1
URI1
ANO4
ARSI
ATP23
B3GALT2
COG5
FAM120C
FAM169A

FERMT3
GBF1
GPAA1
H2BC11
HAPLN3
IDO2
IKZF5
LSM7
MCRS1
MED12
MIR375
MPDU1
NAA20
NSMCE4A
PAEP
PIN4
RABEPK
RT1-DB1
SLX4
SNW1
TBKBP1
ADRA1D
SLC15A2
APOBEC3B
CACNA1I
SEC14L2
SMARCE1
TDP1
TENM2
ADH6-PS1
ADAT3
CATSPERD
COQ8B
CROCC
EAPP
EMC2
FLT3LG
FSD1
HS1BP3
KANSL1
KBTBD3
KCNJ9
LCLAT1
MINDY2
MRPL28
MRPS23
NEK8
NSUN3
OLFM2
PIK3R4
PRR5
RNF135
RNF40
SCGB1D2
STX18
SYNDIG1
SYT10
TBX19

UBXN10
YBX2
ZC4H2
ZNF622
ZNF697
SPRY2
ADGRF5
BCL2L10
CORO7
FOXF2
MAB21L2
MRPL1
PTPN5
RAB3IP
RAC3
RPL18A
SCN4A
SNX8
USO1
ZC3H6
CHRM1
HES5
MTDH
PRKCSH
TINAGL1
ANKRD6
ATP4A
CACNG2
COQ6
DARS1
DHDH
DNAH8
DR1
FAM136A
GDAP1
H3C2
ISLR2
JAGN1
KIF3B
MAP3K10
MYRF
NHS
PDCD7
PHYKPL
PSMD9
RANBP9
SLAMF1
SPTLC1
UTP25
ZC3H7B
ADAMTS16
C19ORF12
CAR11
KCNA7
LIPN
LRRC10
MUL1
NACAD

NAT8B
NXPE2
PDE6G
SCRT1
PTPN2
ANKRD22
ATXN2L
CHST2
CRHR2
DENND1B
H2-AA
HPCAL4
INTS7
ITPRID2
MIR210
NSL1
RASGEF1B
RBM15
S100A3
SEMA4B
SLCO1B3
CARS1
DTNA
NFATC4
ADAMTS14
AURKAIP1
BUD23
CCDC17
CDK3
CLDN18
CMYA5
CST8
DHX32
DHX57
DNAH6
DUSP15
DYNLT2B
EEF1AKMT2
EIF2S3X
EMILIN3
FLRT1
FRG1
FUT9
GFRA4
H3C8
KIAA0040
MIR191
MIR425
MYG1
MYH7B
ND4L
NPB
RHAG
SMG5
SRRM3
SURF2
TEKT1
THAP7

TIMD2
TMPRSS15
TRAPPC2
VCPKMT
VPS18
WASHC5
ZBED1
ZBTB48
ZC3H18
ZFP638
ADAP1
BLK
CMAS
COA5
CRYBA4
CSRNP3
CTRL
DSCAML1
ELP6
FHDC1
GPC5
H2AC8
HARS
HCFC2
INAVA
LRRC41
MCMBP
MED23
MIR142
MIR34B
MRE11
MSL1
P4HTM
PABPC1L
PCSK1N
PRL2C3
RMND1
SHOC2
SLC12A9
SV2C
TMEM79
TTLL3
UPK1B
ZBTB26
ATP6V0A2
CCL26
CDK5RAP2
CMA1
CUBN
ERI2
GUCY2C
H2AJ
H4C9
IL27RA
KBTBD8
MPP2
NEURL3
PIAS2

PPP1CC
SLC2A13
SLC6A11
ST8SIA2
TRANK1
UNC45B
ES1
FERD3L
KRT28
PHLPP
RAPGEF2
RNF113A2
SLC22A28
TCAP
THA1
WDR93
IL4RA
BMYC
CAAP1
CYP2B7P
FLACC1
HOXC8
IRX6
KCND2
KLHL36
LUZP2
NECAB3
PCDHB15
S100A12
SATB2
SULT3A1
TNP2
TPSG1
TRP73
ZFP219
ZFP644
ZNF787
ZPBP
AK5
ARAP3
ARL5A
ATP5PB
BICD1
CEPT1
CHRNA3
EFHC1
ERF
GJB3
MRTFA
PCGF5
PHACTR1
RYR3
SLC16A14
THEMIS2
VANGL1
ATP6V1E1
ATXN7
CDKL5

CHMP1B
CNTN3
CPNE1
DERL2
DIPK1A
DLX3
ERLIN1
FAM214B
FRS2
GPR137C
H1-5
MCHR1
MGME1
NANP
NCLN
NETO1
NLRC4
PI4K2A
RPS6KB2
SARM1
SERTAD3
SH3TC2
SLC16A8
SYNE3
SYT9
TP53TG1
XYLT2
ZNF367
ABHD17C
ADGRB3
ANTKMT
BCAS4
BLOC1S5
CD96
CHST5
CRYBB1
CYP2C65
DNAJC4
EFCAB14
ENPP7
EVPL
EXOG
FAM83A
FAM8A1
FGF16
GPR173
H2AC15
KATNAL2
KCNH7
LRRC3
LTK
MAPK8IP2
MIR23B
P2RX6
PCIF1
PLA2G2E
PPP1R3A
SAMD4B

SP8
TELO2
TMEM63C
TTLL11
TUBGCP6
UBE2Q1
USH2A
UGT1A3
GNRHR
COPA
DOCK7
EFNA3
FAT3
SOS2
TBC1D14
SCN5A
BVES
C1RL
CA5A
CDH16
CHD8
EXOC7
FAM117A
GRIN3A
HUS1
KRT34
KSR2
P2RX3
PATZ1
PDE6H
PFDN1
PINX1
PLD5
PLEKHG2
RAD54L2
SEZ6L
SFI1
SVIP
TLL1
UHRF1BP1
WBP2
WDR74
ZBED3
ZFAND3
ZFAT
PIMREG
ROR1
SCT
ABHD8
ARHGAP39
B3GLCT
BIN3
C1ORF54
CAPS2
CHPF2
DDX47
DIRAS2
DSCAM

ELOF1
ENKUR
GALNTL6
GNGT1
GTF2IRD2
KCNH5
KLK7
LRRC23
LRRC4B
LY9
MARCHF4
MED26
MTMR3
NSUN7
OFD1
PYHIN1
RPAP1
RUSF1
SEBOX
SLC35A5
SMPD4
SPPL3
TBC1D22A
TM9SF1
VIPAS39
VPS25
WDR5B
WFDC21
WRNIP1
ZC3HC1
ANKRA2
CYRIA
DNAJA3
FTO
GRIK3
KCNH1
POC1A
PURB
RAB11FIP1
SLC22A9
VTG2
APOEB
ASTE1
CCDC184
CFAP97
CRYGN
GSDMC
H3F4
ICOSL
IGLV1
KCNS2
MC5R
MIR149
MTFR1L
NAIP2
OAS1B
PPFIA2
QPCTL

SAMD14
SELENOO
SLC22A27
SLURP1
SMIM22
SPEF1
SPRR2E
T
TP53I13
TTC21B
UTY
ZFP330
ZNF692
AWAT2
C20ORF204
CYP2Y3
GPIB
ADAD1
ALDOAA
AMY2B
AZU1
DEFA3
EIF2S3Y
KLRF1
MIR663A
MSL3L2
NOLA2
PCDHA7
PCDHB22
PRG3
SOHLH1
SYNGR4
UBE2U
YJEFN3
ZFP414
KCNJ3
RPL5
ACSBG1
ALKBH8
ANKRD26
BOLA3
CD320
CHST6
CPTP
CRYBG3
CUEDC1
EDA
GIMAP4
GPRASP1
H4-16
IL21R
JOSD1
KCNG3
KLHL14
LRRN4
MIGA1
NPAS1
P2RX1

PACSIN3
PARVB
PEX16
RAB11FIP3
RBM20
RBM22
RNF146
RNGTT
RPL38
RT1-EC2
SACM1L
SAMSN1
SBF2
SCLT1
SHC2
SINHCAF
TMEM218
TRNP1
U2SURP
USP48
AGAP1
ARFGAP3
ARID4B
CETN2
DIXDC1
GALNT18
IRGM
PLEKHO2
RAB29
RBPMS2
DLX2
PIDD1
ACOXL
AMY1A
ANKRD55
BRINP3
CALR3
CDHR5
CELF6
CLEC4N
COQ4
DHDDS
DNAJB12
DNAJC11
FAM50A
FOXJ2
H3C10
ITLN1
KIAA0930
LRRC75A
LRRTM1
NAA40
NDUFAF3
NXNL2
ORC5
PAM16
PCYT1B
PIGU

POLR2E
RAB9A
RBP3
RETREG3
RNF139
RRP8
RTCB
SERPINA3C
SIGLEC1
SLFN3
SPIDR
THOC7
TMEM63B
TRIM69
VSIG10L
WASHC4
ZNF467
ZNF641
APC2
ARFGAP2
ARFIP1
ATP5ME
CCSER2
CHD6
CNOT4
DPH3
EPN3
ERGIC2
FOXL2
HNRNPUL1
IRX2
KLHDC8A
MRPL42
NAGS
NOS1AP
NTNG2
OMA1
PAIP2B
PIP5K1A
PLEKHG4
PVT1
RAB7A
RAB7B
ROBO3
RPAP3
SNRNP40
SNX3
TMX3
UBN1
UQCC2
URB2
CDC42EP4
NAA50
OTX2
PLCB3
POP1
RAB6B
TEAD4

TNKS
NPL
BCL2A1D
COPS7B
CYP2A7
CYP3A43
DBF4B
DND1
FBXO45
GLTPD2
H2AC1
HPSE2
HSF4
KATNIP
LENG9
LRRC73
LRRIQ3
MEI1
MIR382
NDUFAF5
NELFB
PLAC8L1
PRSS36
RXFP2
SLC10A5
TMEM179B
USF3
SNAP25
CACNA1H
RAP1A
AKR1C13
ARV1
ATG2A
BBS12
CAND2
CBY1
CDYL2
CERS2
CHIA
COG1
CSN1S1
CTU2
CYP2F4
EDARADD
FYB1
GPR108
GPS2
GRIPAP1
GUCD1
KLHDC9
MB21D2
MED31
MEMO1
MINDY1
MINK1
MIXL1
MRPL27
MTMR10

NIPSNAP3B
NPVF
PLEKHJ1
POLR2D
PRND
QSOX2
RHBDL3
RMDN3
RNF8
RNPC3
SLC25A26
SLC45A1
SNRNP48
SUMF2
TAF11
TMEM141
TRMT13
TRMT9B
TYW5
U2AF1L4
UBXN11
UGT2B37
YTHDC2
ZBTB8A
ZMYND10
ZNF521
ZNF750
ZSCAN31
ZUP1
ASPN
ATP5F1D
FBXL20
PIK3C2G
SIAE
TBX2
CPNE2
CREBL2
DAGLA
DLL3
EPHA8
FAM172A
FAR2
GATAD1
GNPNAT1
GPR85
H2AW
JAKMIP1
LEPROTL1
MRPL35
NMRAL1
ODF3B
OLIG1
PHF10
PIP4K2B
PLA2R1
PPP1R16B
RAB3GAP2
RASAL2

SBNO2
SCAF4
SCN2B
SETD6
SLC28A1
STK3
SYNC
TAF7
MUP6
AMY2A5
ANGEL1
ANKRD53
C1ORF198
CES3B
CYP3A59
DLEC1
GLOD5
H1F3
H3C14
HOXC11
KCNK7
MCEMP1
MIR33A
NT5C1A
SLC22A21
SLC52A1
TREML2
TUBA3B
ZFP281
CDC42EP2
CTLA4
FSTL3
GNS
SRGAP2
ZBTB43
COL9A1
CYYR1
DSC3
DZIP3
EARS2
GARNL3
GINS4
IL4I1
ISCU
KCNJ10
LNX2
MICU1
MTNR1A
PEX14
SLC18B1
SLC35G2
SNHG8
STARD8
SULT1A2
TMEM209
TMEM30A
TRPM3
WDR18

WNT9A
AKR1CL
ATP5IF1
ATP6V0E
BFSP2
CCDC134
CERKL
CFHR2
CLCN1
CPA6
CYP4F1
DMBX1
EEF1AKMT3
GPR63
HOXC13
KANSL3
KLHDC1
LRP1B
LRRC61
MRPL43
MYO15B
OARD1
PSME3IP1
RGD1307603
RHD
RNF215
ROPN1
RPF1
RPS4Y2
TMEM184A
TMPRSS9
WDR83
ZNF616
ZNF710
ZNF79
AASDH
ADAMTS10
AFF2
ANKRD27
BTG4
CCDC115
DCAF12L1
DCTN6
DNAJC16
FBXO38
IDUA
IGSF21
KLHL15
KPNA5
MNDA
MTFMT
NXPH1
OXSM
PAN3
PAQR6
PDE6B
PRAME
RPL39L

RRAGB
SCO1
SMG7
SYTL3
TGDS
TMEM126A
TMEM131
TMEM52
TMPRSS3
TRO
UNC5D
ZBTB11
GRP
HELZ2
MYL7
CEACAM2
SLC1A3A
SNORD15A
ASB17
DMRTB1
TNFSF8
AAMDC
CERK
DIAPH1
DIS3
DNAH1
ESF1
GMFG
HCLS1
IGFBPL1
ITCH
KLRG1
NPTXR
P3H4
RPGR
SKAP1
TSG101
CAMK4
CNTNAP2
EIF3I
FLT3
TOLLIP
ABCA4
ADGRG3
ADPRM
ALG12
ALG5
BRINP2
CEP57L1
CNIH2
DCSTAMP
DDHD2
DEAF1
DHH
DHX29
DIPK2A
ECD
EXTL2

FAM193B
GALNT5
GIGYF1
GLT8D2
GRAMD2B
ILDR1
KCNH3
LRRC56
LRRC6
MED29
MIR130A
PUS7L
REX1BD
RFESD
RGL2
RHOT2
SBF1
SGSH
TRAPPC10
TRIM31
TRPV5
YEATS4
ZBTB46
ZNF462
CYP4A22
H2AC10
H2-Q2
LALBA
MIR491
PWWP4B
TRIM12A
ABCA1A
ABCA9
ANKRD39
AP5S1
ATP1B4
ATPAF2
CCDC116
CDH12
CFAP126
CHP2
CHRDL2
CLCNKA
CLEC12A
DCST1
ERVMER34-1
FAM160B2
FHAD1
FN1B
FYB
GP9
H2-T10
KIAA1328
LYSMD1
MID2
MIR188
MIR802
NPFF

OPTC
PCDHA11
PCDHGA9
PCGF3
PPP1R35
PRDM12
PRKAG3
RTL8B
SPOPL
TRIML2
TTC21A
TTLL4
TUBA2
VPREB3
ZFP292
ZFP809
ZNF200
ZNF362
AHI1
AIFM3
AKNA
CCNK
CDKN2AIPNL
CMSS1
COPS2
CPB1
ELF5
GOPC
LUZP1
MIR19A
MRPS7
MTHFD2L
MYT1
NGB
OGDHL
SBSN
SEC16A
SEH1L
TMEM205
TTYH3
TXNL4B
GABRA5
LHFPL6
PYY
SOSTDC1
ABHD10
ADPRS
ANKIB1
AOAH
B4GALNT4
CCDC15
CR2
CYP2C13
CYP2J9
DMRT2
EDC3
EFCAB11
ELAC1

ENOX2
ENPP6
ERI1
FSTL4
GPR84
GRHL2
HES4
HMGXB4
HS2ST1
MAB21L3
MIR134
NAP1L3
OVGP1
PDCD2L
PDIA2
RELL2
SCRN3
SH3GL1
SLC37A1
SNX27
SPRYD7
TARS2
TCTN1
TSEN54
WDR11
ZCCHC17
ANKS1B
EHMT1
EPHB1
GFUS
GGT5
H2AC18
LAT2
MC2R
MYH4
PNKP
RAB27A
RGMB
SLC7A3
VPS13C
ACTR1B
ACTR8
AMELX
APOBB.1
ATRIP
BTBD6
C1QTNF12
C9ORF85
CCDC71
CCDC85C
CDSN
CMTR1
ENTPD6
FTMT
GABRG1
GABRR2
H4C4
IGH-VJ558

KCNJ14
LRRC29
MON1A
NT5C3B
PDZRN4
SLC34A3
SMN2
TADA3
TEFM
TPRN
TTC7A
UQCC1
PNPLA6
FGF13
IRX5
KAT6A
PPFIA4
UGT1A4
ABCC10
ACY3
ANKRD34A
ASAP3
ATG13
ATP5C1
BCAS1
BTBD2
CA8
CD244
CHRNA7
CSN2
CYP4F3
ELP2
EPRS
EXOC5
FIBP
GLYCTK
GPR132
GPRIN1
HBE1
HDGFL3
HHAT
IKZF4
INPP5F
KIF1C
KLC2
LLGL1
MDGA1
MFSD8
MOAP1
NAP1L2
NME5
NMNAT2
PAQR8
PPIP5K1
RNF165
RSPO2
SCO2
SEPTIN5

SLC22A11
SNAP91
TBC1D8B
TBX5
TMEM214
TMEM243
USP16
UXT
APOBEC3F
BICRA
C1ORF210
CCDC162
CD300C2
CLEC4A2
CLVS1
EVI1
GPR45
GPX4A
PCDHB11
PKD2L2
PROB1
RAB44
TSPYL3
XLR3B
YARS
ZFP799
CYP2A22
EAR10
FAM110D
FREQ
HEATR4
SRF
ZFP653
ADM2
CELF5
CLDN12
CPLX1
DNASE2
DUOX1
KCNE2
MKKS
SRCIN1
STX7
TTLL12
VPS35
BTN2A2
CHMP6
EXOC3L2
FAM3A
GRID2
HDGFL2
IGH
KLHL40
MIR542
MNX1
PPP1R17
PSORS1C2
RAB42

SLC28A3
SSNA1
TOP3B
TRIM17
WFDC3
ZDHHC11
ADAM2
ARHGAP33
BCL2A1B
CCDC97
CELF3
CEP72
DNAL4
FBXW4
FOCAD
GAS8
GYG
HMG20A
INTS9
KCP
KTI12
MIRLET7C
PDZD4
PNPLA4
R3HDM4
RASAL3
RETREG2
STCH
STK32B
TMEM26
TOMM6
VPS11
WDR25
XKR5
ZNF195
ABTB1
ATP6V0E1
CAR4
CEP43
CLEC5A
DISP2
DUSP19
EDF1
FOXRED1
GALR1
GEN1
GPR107
GRIN3B
HOXB8
IGKC
IMP4
ITGB1BP2
ITK
KIF26A
LSM14B
MIER3
MOBP
MORN2

NEK3
PHYHIP
POLR2B
PYROXD1
RAB22A
RHCG
STK19
TMEM268
TMEM87B
TTF1
VASH2
WBP11
GNAL
MIR22HG
PDYN
CACNA2D3
CEP128
DENND1A
KCNQ5
MYO9A
OTUD4
PAQR4
RAB3A
SHROOM3
SOX10
TREX1
AGAP3
ARHGAP32
ARHGEF15
CCDC138
CLIC2
CYTH4
DGCR2
EFL1
EVA1C
EXO5
FAM241B
FBXO28
FGF11
GGACT
GLDN
HAUS8
HP1BP3
IL34
KATNBL1
LRRC2
MEFV
NFKBID
PPP4C
RAB35
RABL6
RNF123
SH2B2
SLC35B3
TBC1D9B
TBX18
TESK1
TMEM259

TPCN2
ADCK1
ALOX12E
ANXA8L1
ARRDC1
BARHL2
CELA3B
CENPC
COL6A5
EXD2
H2AC23
H2-OA
KIAA0100
KLHL10
KRT31
MIR151
PCDHGA3
PTH2
SHISA9
SOX30
SPTBN5
TCP11
TMEM229A
TMIGD1
ZFP280B
ZFP7
ZNF337
ZNF511
BTN3A1
CACFD1
CASS4
CCDC130
CCDC149
CCM2
CD1D
CDK11B
DEFB4A
DRG1
FSTL5
GNL1
GPR37L1
GTF3C3
IL11RA
KLHDC7B
KREMEN2
LHFPL3
LTB4R2
MEGF11
MGAT4C
MLLT1
MMS19
MUC15
NBPF1
NFIL3-6
NGP
PKHD1L1
PNMA1
POLR2J

PTDSS1
PWWP2A
RBMXL1
SHKBP1
SNX30
SPATA5L1
TAMM41
TMEM178
TRIM45
WFIKKN1
XXYLT1
ZAN
ZNF33B
ZNF562
ZSCAN2
CDC14A
CHRND
DIP2C
DNMT3L
EFS
FAM98A
OVAL
PARD6B
PCSK2
SEMA4F
ST8SIA1
SYT2
TMEM100
USP34
ADCY5
ADAMTSL1
AGO3
CYP4V2
DGLUCY
FBXO6
GAREM1
GLRB
GOLGA5
GOLPH3L
GPR18
HDHD2
HNRNPA0
HTR2A
LIX1L
MSANTD3
NACC1
NOXA1
PAXIP1
PBSN
PDZD3
POU4F1
PPP1R16A
PSD
PUM3
RABGEF1
RNF182
SLAIN1
SMUG1

SRP19
TBC1D23
UBE2F
ULK4
VPS28
VPS50
ZNF277
SCN1B
ADGRL3
CTNS
DLX1
HCRT
TPPP3
XK
CYP2A12
KLK1B26
MAGEA12
SLC2A7
SPZ1
SUN5
CMPK
NKX2-8
PCDHGB2
ACAP3
ARL13B
CNOT6
CSF2RB2
CWF19L1
CYP2J3
DACH2
DKKL1
EN2
FBXO17
FBXO46
GET4
GZMM
H1F5
HMX2
HOXD11
IFI202B
KNG2
LARGE2
LEMD3
LRRC25
LTO1
MANBAL
MAO
MCAT
MIR139
NRADD
PAX1
PCED1A
PIGL
PIH1D1
PRR14
PSTK
RAB36
RALYL

RASGEF1C
SLC10A3
SNX21
SOX15
STARD3
TAZ
TNFRSF17
TRIM55
UGT1A7C
URAD
VPS37C
ZBTB33
ZDHHC22
ZMAT2
ZNF354A
SIRT2
ABCC11
CIAO2B
CPO
CRISP3
CRYGC
EPB42
FAM163B
FBXO10
FBXW17
GPR26
GSTA
INKA1
INSYN1
MAEL
MARCHF9
MS4A8A
SLC25A2
SLFN8
STUM
SYCN
TEKT5
TLR12
TPTE2
TRMT61B
ZCCHC4
ZFPL1
ZNF688
ATAD1
ATP12A
ATP13A2
DCDC2
DNAJC18
EIF2B1
GPATCH2L
GPR161
GPR37
GTF3A
HOMEZ
LRRC8B
MAN1B1
MIR146A
NECTIN4

NINL
NOL4L
PIP5K1C
PPME1
PRR15L
PRRT1
PTPRCAP
RAB37
RMI1
SCRG1
SDE2
SIM2
SLC10A4
TMEM80
TOMM5
TRMT10C
UTP18
WNT6
EDN3
GRIK2
ATAD5
DGKG
HLA-E
MAP3K20
SSTR1
ZBTB1
ABT1
BAALC
CASTOR2
CCDC30
CDC20B
CFAP45
CMTR2
EPHX3
FBXL14
FCN1
FOXD4
FOXE1
FOXI1
GLIS1
IRGQ
KREMEN1
LARS
MIR107
MIR181C
MTCP1
NAF1
NHLH1
NR1H5
PAK5
PCBD2
PPP1R8
PPP6R2
RASL10A
RBMS2
RPP21
SEC22C
SLC35A4

TCEAL3
THOC5
TNMD
TTC32
ZFP1
ZGRF1
ARTN
CTXN1
DENND4C
ELOB
FAM189B
FBXW5
GGA2
INSM1
KCNT1
LRIF1
LZTS2
MLH3
NRG2
NUP214
PANK2
PPIL2
RPP38
SLC30A6
SRCAP
SRL
TBCK
TSNAX
VSX1
ZHX1
LARP1B
HTR3A
C11ORF96
CC2D2A
CPNE3
GPBP1
UHMK1
MAGEA8
RHOX6
CXCL12B
EGR4
HNF4
MELA
RAB6C
SNORD87
VTG
ZFP513
ASZ1
CABP5
CBWD1
CD1A
DAND5
DHX34
DPPA4
EMX1
FOXE3
IL36A
KRT12

LCE1C
LCE1F
LCE2A
LMTK3
LYZL4
MIR24-1
MIR296
MIR378A
OTUD7A
PABPC1B
PLK5
PRM3
SERPINB1B
SH2D3A
SPIN2A
SSPO
ZDHHC1
ZFP688
ZNF383
AKIP1
BRIX1
CNTLN
DDX42
FCGR1
FGFBP3
GLT1D1
H3-3B
HBB-B1
MAN2C1
MIR96
MPPED2
NIPSNAP2
PAGR1
PDCD1
POMT1
PTP4A2
SASS6
SEMA3G
TMEM130
ABCB8
ANAPC2
BATF2
BEAN1
CEP44
CFAP53
CYB5D2
CYP2D3
DUSP18
GDAP1L1
GGT6
GPR83
GRK4
GTPBP6
H3C7
IGSF5
KCNIP2
LEMD2
LIN52

MIR101A
MIR140
NSRP1
NUP188
OCEL1
PHACTR4
PHF21B
PLPPR4
QRICH1
RANBP17
SARS2
SFR1
SFT2D1
SLC25A35
SLC35C1
TNK1
TOP1MT
TTC19
UNC79
WDR73
ZBED4
ZER1
ZNF117
ZNF706
ADIG
ARHGEF4
CSKMT
DEFA1
DYNLT2A1
MIR24-2
PGAM1A
PKDREJ
RASGRF2
RNASEL
SLC22A30
SLC29A4
STFA1
TMTC3
ZFP449
ZFP61
ZIM1
C3ORF35
KRTAP5-4
LBX1
LRRC52
ZNF366
AARS
CD300A
CEP85L
CYTL1
DIS3L2
DOK5
GBP5
HAND1
KAT7
KIZ
KRT6B
METAP1

MICAL3
MUS81
NCSTN
REPS2
SEPTIN7
SIX3
SLC9A8
SPECC1
STAC2
UBXN4
UCN
USP40
WASHC3
ZBTB2
ZFAS1
GRM2
ND6
RHO
ALKBH3
ASPRV1
CAMK2N2
CCDC148
CCM2L
CCP110
CHAMP1
CMC2
DBR1
DENND10
E4F1
EIF4ENIF1
GPS1
IGHG1
KIF24
KLHDC4
MFSD9
MIR185
MIR26B
MTERF4
PDIK1L
PIGX
POU3F3
PPP6C
RADIL
SGCA
SLC2A4RG
SP140
SPATS2
STAU1
STK33
TAF1B
TMA7
TMEM151A
UBAC2
UBE4A
USE1
APOBA
ATP5MK
BRAT1

CCDC40
CLEC4A1
COL6A6
ERVFRD-1
FAM185A
KLHL17
KLRA1
MIR543
MIR92B
MUP3
NAA60
OCSTAMP
PROL1
SNORD104
TDRD5
TNFRSF6B
TUBGCP4
UBASH3A
VSTM2A
ZFP365
ZFP444
ZFP715
SLC6A2
GRIN2A
FBXO2
PRR11
RNASE1
ARX
CAPZA2
CARS
CCNJ
CLIP3
EFNA2
FZD10
GPC2
HAS3
MT1L
NADSYN1
PALB2
PEAR1
RHOG
RPE65
TBX20
TRIP4
WDR45B
AFF3
CYBRD1
KLK8
PTGIR
TSPAN18
ARSD
CAMKMT
CAMLG
CAMSAP3
CCSER1
CEP89
CHAD
CINP

DENND1C
DNAAF3
DNTTIP1
DSTYK
EIF1AY
FOXN2
H2-T23
HESX1
HS3ST3A1
IGSF9B
IL17D
LYL1
MFSD12
MPP3
MPV17L2
MRPL37
MVB12A
MYEOV
NEUROD4
NR2C2AP
PACS2
PAF1
PCGF2
PIWIL2
PPP1R3D
RGS14
S100PBP
SEC61A2
SLC13A1
TATDN3
TECPR1
TSPOAP1
UBE2QL1
UBXN2A
ZBTB41
ATG14
CHRNA9
ELP1
ICMT
MAP7D2
MON2
MTA3
PAX5
PEX26
UBE2O
BRIP1
CALB2A
CIDECP1
DBX1
FIGLA
GCM2
H1-6
RDH2
ZFP93
ZNF611
GPR101
ACTR5
ACTRT3

ADGRE4
BCL2A1A
C1RA
CRYGS
DDX19A
EPD
ISM2
KRT85
LAMTOR1
LCN15
PPP1R32
RUP2
TMEM186
VPREB1
ACTL6B
C14ORF93
CAR5B
FAM168B
GTF3C5
SH3BP5L
SLC66A1L
TAFA4
ZFP593
ZNF469
GRIN2D
IL17RA
PDE8B
POU2F2
SLC47A2
BRD1
DBNL
ELMOD1
ENTPD7
EPRS1
FAM210A
FNIP2
GBX2
INPP5B
KMT2D
KRT20
ND3
SEPSECS
SERPINB3
ST6GALNAC4
TEX30
TMEM116
BBIP1
BFAR
BFSP1
CCSAP
COLGALT1
DNAJB14
EXOSC10
FAAP20
FAM181B
GHDC
GPATCH11
H2BC7

IFI27L2A
IFTAP
LIME1
MAP3K9
MIR130B
MRPL32
MRPL36
NEXMIF
NFRKB
PGAP6
PODXL2
PPP2R3C
RNF112
RNF121
SASH3
SNRPC
SPINK4
STARD9
SYCP1
TMEM18
TMEM230
TXLNB
TYW1
UBTD2
USP38
VAMP7
ZNF185
ZNF267
ZRANB1
KRT35
LY6C2
THEM7
ZFP971
A4GNT
BIRC5A
EFCAB8
H1F9
ZNF157
BICDL1
C1QTNF1
CADM3
CNST
CPA2
CYP3A18
GPR19
ING5
MYNN
PLAAT1
PLEKHG1
PRSS35
RBM4B
RSPH9
SLC9A2
USH1C
USP31
ACAT3
ANKRD1B
BHLHE23

BNIPL
CARD14
CARD16
CD300LG
CETN4
CNOT11
CYP2J6
DEFA5
DYNLT4
EPHA6
EXOC6B
F8A1
FOXH1
GSDMA
GSTT1B
GZMC
KARS1
KCNS1
LGALS12
MAP1LC3C
MIEN1
MRGPRE
NFKBIAB
PLAAT5
PPP1R42
PTH2R
SDHAF4
SSUH2
TBC1D25
TLR13
TMPRSS7
TRABD2A
TRPM5
ZFP386
ZNF512B
AMIGO3
BHLHE22
C1ORF21
CDR2L
CNTROB
CRACR2B
DNAI3
DTWD2
EBAG9
GADD45GIP1
GAN
GORAB
IFT140
IL37
IQCE
JOSD2
LMLN
NUDT15
PCDH12
PHF13
PPIE
R3HDM1
RAB33B

RAP2C
RNF181
RNF20
RNF31
RWDD1
SERP2
SLC39A3
SS18
TASOR2
TBL2
TTC37
WIPF2
YIF1B
ZCCHC18
ZNF83
CCL3L1
INSL4
PTCRA
TIAF1
ZFP84
ADAM21
ANKS1
C15ORF62
C3ORF62
EBF4
KCNK16
KIAA1841
KLK15
KRT33B
MIR421
NANOS3
SAYSD1
TARSL2
TGTP2
TPPP2
ZFP180
ZFP672
ZFP74
HS3ST2
KIF5A
LDHC
PRKCEA
SLC5A12
ACTN3
EPG5
PPP1R9B
TEAD2
TMEM255A
TSPAN15
AMPD1
ATAT1
ATP5J
CCDC12
CCDC125
CCDC146
CIC
CLEC16A
CNKSR1

CRX
DDO
EPB41L4A-AS1
ETAA1
FBXL6
FER1L4
HCST
HEATR5B
LSMEM1
MIRLET7E
PHACTR3
PIP
POF1B
QRSL1
RNF183
SCARF2
SPX
SSC4D
TMEM231
TOM1L1
TRNT1
UBALD1
ZC3H4
ZNF219
CHL1
CHM
CLEC4E
DTX1
EBF3
GATD3A
HGSNAT
KCNN3
MIR17HG
MPI
MT1B
POLR3H
SIPA1
TPRA1
VARS1
ASTL
BBLN
BMX
CALHM5
CDNF
CEACAM20
CHMP7
CIAO3
CYBC1
DIS3L
DNAJC5B
DRC1
EXOSC1
FAM189A1
FAM50B
GOLGA8B
IER3IP1
L1TD1
MMGT2

MRPL44
MS4A4C
NABP2
PLA2G2C
PRCC
PRICKLE3
RAB19
RAPSN
RBP2A
SCARNA17
SHD
SLC25A38
SLC6A16
STX5A
SYT14
TBR1
TRAPPC8
TRMT2B
TSX
TTI1
ZNF239
ZNF512
ZNF660
ZNHIT1
CYP11B1
FGF23
RPL37A
DRD4
ADSS1
AMBRA1
ANKEF1
ARHGAP23
CCDC167
CCDC171
CHMP5
CHRNA6
CYP4V3
CYTOR
DHX40
DSG3
EEF2KMT
ERG28
FAM174A
FCGR2A
FXYD4
GJB4
GLE1
GTPBP3
H1F4
IFIT5
KAT8
KCNIP1
KLF16
KLHL6
LRMDA
NEUROD2
OTUB1
PAPOLG

RAP1GDS1
SCGB3A2
SCML2
SHQ1
SLC18A3
SLC25A43
SLC52A2
SLC8A2
SNCAIP
SOX12
TMEM185B
TMEM229B
TRAF3IP3
U2AF2
ULBP1
YTHDF3
IP6K3
VXN
NF2
METTL7A1
OR8B8
PISD-PS3
RT1-DMA
C1ORF216
CRYGE
IGF2B
KIAA0825
MIR872
MORN3
RUFY4
SLX1A
SMIM6
TULP1
ZAR1
ZFP131
ZFP958
AXUD1
CACNG1
CEND1
CENPC1
CYP2F1
DNAAF4
FRMPD1
GEMIN7
GINM1
GUCA2A
H2BC13
IQUB
IRS4
KBTBD6
KEAP1A
KRBA1
LINC00312
MED19
MIR195
MIR409
MTG1
MYF6

NARS
PLXNB3
PRIMPOL
THSD7B
TMEM94
TRP53I11
TRPC2
USHBP1
VPS37D
ZFP82
ZNF281
AGRP
DISC1
FGF14
GLMP
KCNK3
PTGDR
RAPGEF6
ZNF292
MIR194-1
GJA9
BMB
DMRTC2
FGF18
GBP10
H2BU2
KRTAP6-1
MAP6
RAG1AP1
SPRR2K
H1-8
HTN3
INHBAB
KLHL33
LCN10
MFSD7A
PRKCBB
TPRGL
BEGAIN
CABP1
CEACAM10
CHMP1A
CUL7
FBXO36
FCER1A
GMCL1
GUF1
IGFBP1A
IGFBP1B
KCNK6
LGI4
LYRM2
MIDEAS
MRPL41
NPAT
PCDH15
SDR16C5
SLC7A4

SMIM8
SMTNL2
SPG11
SYNE4
TCERG1L
TMEM108
TRAPPC6B
FRMD5
GNG8
LARP4
LZTS1
SYNGAP1
A3GALT2
ALT
B4GALNT2
C1ORF115
C4ORF46
CCDC152
CERCAM
CNTN6
CPSF4
CRNDE
CRPPA
EIF4EBP3L
FBXW8
GUCA2B
H4C6
IBA57
KLRC1
MIR154
MIR328
NAT14
NBPF10
NKIRAS2
NSUN4
PCDHB16
PIGO
POLR3A
RBP2B
RPS6KA6
TAF4
TARBP1
TBC1D10C
TRAM1L1
TREML1
ZFP385B
ZNF287
ZNF84
ATG9B
BICDL2
MAB21L4
MICOS10
RPPH1
RTL6
RTRAF
RUNDC1
ZBTB12
ALPK3

ARHGAP44
ART5
ATP13A4
CADM2
CCDC186
CD6
COX16
EVI2B
FBLN7
GDF1
GLUD2
IFNA2
IL21
INTS10
L3MBTL1
LEPROT
MBD5
MED24
MZF1
PFDN6
PRPF31
RFTN2
RNF217
SEC24C
SLAIN2
SLC30A9
TIMM17B
TMEM87A
TOM1L2
UBE2E1
ZCCHC8
EIF5B
KRT16
HCN4
HNRNPH2
HTR1D
KISS1R
RTN4IP1
ANKRD36
MIR101B
ASB12
BEST2
BTNL2
CCDC198
CEACAM19
DIABLOA
DPY19L2
ERFE
ERN2
GAL3ST4
GGCTB
GIMAP3
GLULA
GSX1
H2-Q7
LRRC71
MBPB
ODAM

PCDHGA2
PSG5
SLC35F4
TRIM30
USP6
ZFP40
ZNF442
CYP11B2
GABRA1
HTR1A
ALDH3B2
ALKBH2
AVPR1B
BEND7
BRMS1L
CAPN7
CISD2
CWC22
DPM1
ESYT3
F2RL3
FAM160A1
FGD1
ITGA10
KDM1B
LETM2
MAGOHB
MED16
MYLK3
NKAIN4
NKAP
NUDT9
RWDD2B
SALL3
SCUBE3
SECTM1
SLITRK4
SNRNP35
SNX33
ST6GALNAC1
SYDE1
TTLL5
ZNF532
ASXL1
CHMP4C
DNM3
LRRC8C
PHF6
SNHG12
CD207
MIR224
ALX3
ANKS1A
AP4S1
ARL5C
CABCOCO1
CDHR2
CIAO1

DCAF12
DHRSX
DHX8
DPCD
DRP2
EXD3
GATD1
GK5
GKN1
GPN3
H2-OB
HID1
HMCES
IFT52
IQCB1
KIAA0513
KRT222
LILRA5
MFSD4B
MISP
MPPED1
NEURL1
OGFOD3
PABIR2
PANK4
PIAS4
RMND5B
RPUSD4
SNTG1
SOGA3
SPAG7
SPIC
SPRN
SULT6B1
WRAP73
ZDHHC6
NOX2
SEC14L1
STX11
ZMYM5
ACOD1
ATP5J2
AZI2
BDP1
CACNG7
CCR8
CHRNB4
CHURC1
DCP1A
DSPP
FAM83H
LRRC34
MACROH2A2
NLRP1
PPM1E
RBAK
SI
SLC25A44

STK32A
TNR
UBE2R2
ZNFX1
CPHX1
NGFG
ZNF582
CCL3L3
EAR3
ESPNP
MIR365B
RGD1562339
SPRR2H
CAML
CES1B
FAM135B
H2BC22
HYAL4
IMPG2
KRT1-19D
PABPC3
PCDHGB4
PSG9
PTPRQ
RDH1
STAT5
TMEM167
VSIG2
ZFP97
ZNF628
ABCA17
BLOC1S4
C17ORF49
C6ORF47
CABP2
DHRS4-AS1
FLYWCH2
GGN
HSP90AA1.1
IRAG2
KLHL1
MCF2L2
MCL1A
PCDHA2
RBM23
RIMBP3
RRH
TCTE1
WDR53
XKR9
YIPF7
ZFP346
ZFP553
FOSAB
LTN1
MIR1224
MIR145
RCOR2

AGR3
ALG1
BPIFA1
C14ORF132
CDH24
CEP295
EDAR
FCHO1
GLB1L2
GPR20
GTPBP8
HOXB1
HSH2D
ICA1L
INSRR
IPPK
KCNK4
LRCH4
MED9
MIR497
MRPL10
NATD1
NEPRO
NFE2L2A
NIPA2
NR2E1
NUDCD3
PRXL2C
RIBC2
SLC26A7
SMIM29
SUDS3
TENT4B
TNNI3K
TRAPPC5
TTC4
ZFP703
ZNF561
CYP21A2
PENK
ALOXE3
APBA2
ATP5PF
CEP19
CRYBB2
DTNBP1
EDEM2
EVC
HDDC2
KLHL28
MIR206
PLCB2
PRSS22
SCMH1
SEPTIN3
SLC44A5
SLF2
UBA6

ZNF207
DSG1B
PRDM7
JAM3B
MIR499
OR52W1
AP1M1
CCDC66
CRIPT
DPEP2
H1-4
HPCA
ILRUN
KIF7
KLHL26
LAIR1
LYSMD3
MCPT1
MEIOB
MMP25
MPLKIP
NLGN2
PCNP
PCNX4
PLCH2
PRDM5
PRM1
PVALB8
RD3
TTYH1
ADAT1
BBOF1
C21ORF91
CNGA1
DNAL1
EPN1
FAM199X
FAM30A
FBXL2
GLYATL1
HECTD3
IQSEC3
MORN1
PLA2G4B
PSMG3
SLC4A1AP
TMEM126B
TMEM74B
TMEM8B
TRAF7
UBXN2B
VWA7
WIZ
ZDHHC4
ZMAT1
KIDINS220
TC2N
DCDC2A

KLRB1A
PNLDC1
SPATA25
ZFP459
B9D2
C20ORF194
CA7
CLUL1
COP1
CPLANE2
DYRK4
ELOVL4B
FAM86C1P
FCHSD1
FKBP6
GLP2R
H2-M3
H2-T22
KLK4
KRT27
LBX2
MIR424
NCMAP
PFKMB
POTEF
SMCP
STARD6
TEX36
ANKRD13C
ARHGAP42
CACNG4
CDK2AP1
DARS2
DSEL
FUOM
GRIK4
ILVBL
LHX9
LIN7B
MFF
MIER1
MRPS14
PREPL
PRRC1
TMEFF1
TMEM88
YEATS2
CHRNA5
H2AFX
ATG101
AUP1
BMP10
CCDC96
CDAN1
CEP104
CLASRP
COMMD9
DNAH17

EVC2
FBXL13
FSIP1
GIMAP5
GPR137
H3C12
H3C13
HACE1
HBG2
HMG20B
HYAL3
LHX3
LINGO2
LRRC27
MED22
MFSD5
MIOS
MIR135B
MIR193B
MRM2
MRNIP
MYO3B
MYO7B
NEURL1A
OLFM4
ORMDL1
PI15
PILRB
PPIL6
PPM1F
PTAR1
RAB9B
RNF111
SIKE1
SKIDA1
TCTN2
TMEM59L
TOPORS
URGCP
VMA21
YPEL4
CD59B
CYP2D40
DEFA6
DPYDB
FBXL21
FER1L5
GT(ROSA)26SOR
MAGEB2
MUP19
SNORA69
SULT2A7
AQP8A.1
DEFA29
HTR1E
MAGEA9
PCDHGA4
RBM46

SLFNL1
TMEM89
ZFP280C
ZFP92
ANO3
CCL19
CLDN8
COL23A1
CSTF1
MAK
MAP3K11
STK35
SV2A
AFP4
ANKAR
ANKK1
CCDC170
CEACAM3
CLDN17
DPPA3
EAR1
FAM122B
GAPT
GBP7
KCTD8
KIAA0754
LCE1D
LGALS2B
LHFPL5
LINC01554
NBPF3
PHETA2
PPP1R14D
RNF25
RRP36
RSC1A1
S100A5
SLITRK2
STRIP1
TBXT
TIGD5
TPRG1
UGT2B38
WARS
ZC2HC1C
ZFP24
ZNF263
ZNF488
FBXO39
CHRM4
HTR6
NAPEPLD
PATJ
ABHD13
ALX1
C12ORF57
CCDC103
CEP162

DIPK1C
DQX1
EIF2S3
FAM124A
HTR5A
LIN9
LRRC75B
LYRM9
MIR106B
MRS2
NAXD
NIPAL4
PEX5L
PGAP3
PRELID3A
RUBCN
SIM1
SPRR2B
TAC4
TCF23
TCHP
TIMMDC1
TP53RK
TRPT1
WDR41
ZDHHC8
ATP6V0A4
EGLN2
HLA-DPB1
ICAM5
KCTD14
MYOC
NCAN
SLC35C2
TPH2
UBQLN2
PDE1C
SLC30A2
ANXA9
B4GALT4
DPP10
ESYT2
KCNT2
OVOL1
SEMA3D
SPOCK3
SYT4
TAGLN3
AAGAB
ABO
AMER1
ATXN7L2
CYP1C1
DCAF17
DNAJC27
EBF2
FAM91A1
FRMPD4

GOLGA1
IQSEC2
KATNA1
KBTBD4
KCNG2
KCNK13
KIAA1549L
KRI1
LYRM7
METTL22
MIEF1
MIR423
MIR532
ODF2L
PDCD1LG2
PIBF1
RESF1
RPUSD2
RTN4RL2
SAG
SOX21
SPINK2
ST8SIA5
SYT6
TGFBRAP1
TRIM3
TXNL4A
VWA5B2
ZDHHC17
ZNF618
ZNF624
FNIP1
ALG10
ATP5MC2
CLDN7A
DUBR
EFCAB3
FAM25A
KARS
LGALS5
MIR339
NFE2L1A
NPIPB3
OMG
PBLD2
RT1-CE5
SLC30A8
SMIM4
TKTL1
TMEM222
TMEM91
TRIM15
WAP
WNT8A
ZFP608
ZNF639
ZNF823
CASC15

CCDC43
CLDN19
FAM65B
GPR150
KIF27
LRRC31
LYSMD4
MIR136
MMP27
NDUFAF6
PEBP4
POMT2
SNHG17
SPNS3
TMEM200C
VARS
VNN2
ZFP462
ZNF516
MCPT9
SEC1
SPACA1
TP53TG3
CYBASC3
CYP2C69
EQTN
GPD1B
H2-Q6
IZUMO1R
KIAA0319L
OAS1C
PCDHGA11
PRSS41
SPINK7
SPRR4
SULT2A8
TTTY14
ZFP524
ZFP639
ZFP664
ADAM30
C77080
CATIP
CNTNAP5
KLHL34
KPRP
LINC00482
MIR383
OSGN1
PSG8
QRFP
RNASE11
TMEM105
USP30-AS1
GABRB2
SSTR3
FAM76B
FREM2

KCNJ1
RTKN2
SIK3
SOST
SPOP
DIP2A
MIR324
PLEKHM2
ADAMTS20
ALG10B
ATF7
CCDC65
CD163L1
CDX1
COA8
CTRC
CYB5D1
DMAC1
FBXL17
FFAR4
FOXD3
H2AC11
IDNK
INO80E
LRRC7
MAP7D3
MIR100
MTUS2
MUC20
P2RY10
PADI3
PCGF6
PCNX2
PFKFB4B
PLA2G10
PLEKHH3
PRORP
PRRG3
RANBP3
RNFT1
SDHAF1
SH2B1
SMARCC2
SNHG15
SP2
SP6
TMEM169
TNRC6C
UBTD1
USP44
WDR86
WDR92
ZNF410
DOC2A
HLA-DPA1
IPCEF1
PCDH20
SUMO3

TOR1AIP2
CYP1A3
DAF-16
MIR433
NPY4R
PLET1
ANKS3
ARHGEF38
C11ORF52
C2ORF88
CCDC150
CD300LB
CYB5RL
DDRGK1
DMRTA1
DUSP28
EEF1AKNMT
ETV2
FSD2
GPATCH1
GPR12
HDHD5
HNF4AOS
KCNG4
KIAA0232
KLF8
LRIG2
LRRC57
LRRN4CL
MCL1B
MIR127
MIR411
MTRF1
NYAP2
PCNX1
PIWIL1
PPP1R27
PSG4
RAB6
RIC3
RINL
SLAMF6
STX4A
TMEM185A
YJU2
ZFP260
ZNF142
ZNF33A
ZNF382
ZNF397
ZXDB
H2BU1-PS
MAT2AL
MYCB
CENTD2
MOGAT3A
NRP1A
OLFR1014

SELO
UBALD1A
ZFP128
ADGRF2
SMFN
PRSS58
APOLD1
GRK2
ABRAXAS1
ACD
ADAMTS18
BAXA
CACNG8
CCDC102A
CHCHD5
DNAH12
ERICH5
FARP2
GPR4
HOXC10
LIM2
LLPH
LRRC8E
MESP2
MIR18A
MIR574
MIR99B
MOB1B
OXA1L
PCNX
RBIS
RGP1
RIMS4
SLCO5A1
SMARCAD1
ST6GAL2
STAC
TNFRSF13B
TXLNG
USP37
UTS2
ZBED8
ZNF430
GLP1R
SCN10A
BEND6
CALCB
CCDC85B
DCAF6
DUS4L
GALR2
HAUS2
IGSF10
IL1RAPL1
IQSEC1
KCNA1
MFSD6
SLC5A8

BOLL
ZRP
GLT28D2
H2-K2
KLK1B8
NUGGC
PRKACG
SFRS16
SLC22A14
SNORA5A
SPRR2I
TRIM30D
ABCC13
ANPEPB
APOA1A
CALML5
CCDC58
CD55B
DNM3OS
GJD4
GUSBP11
GZMH
IFITM5
PHF11D
PIGYL
POU4F3
PRSS55
SLC26A3.2
SSPOP
TAS2R31
TCRA
TRIM80
ZFP637
TMPRSS5
UBQLNL
UMAD1
ZNF773
AKAP17B
AP5M1
ARMC5
ATPAF1
CACNA1E
CD226
CDHR4
CHRAC1
CLDN16
CNGB1
COMMD4
CRYBA2
CYP1C2
CYP3A65
DMRTA2
DSC1
EFCC1
ERI3
FAM207A
FAM222B
FAM53C

FBXL19
GATC
GFI1B
GFM2
GH
GLMN
GNRH2
GRIK1
GUCY1A2
HLA-DRB4
HTR1F
IFFO2
IL1F10
JRK
KCTD7
KIAA1217
LRRC4C
METTL23
MIR351
MMAA
MZT1
NKX3-2
NSA2
OMP
PARVG
PAXBP1
PCDHAC2
PGAP4
PGCP
PHOX2B
PLEKHM3
PM20D2
PPM1J
RAX
REXO1
RSPH14
SAMD10
SAMD12
SCGB1C1
SGF29
SIX6
SLC6A19
SLX4IP
SPAG8
SRP14
STPG1
SUGP1
SUSD6
TCN1
THTPA
TM4SF18
TM4SF5
TMEM182
TRIM71
TSTD3
TXNDC11
WSCD2
ZFP30

ZFP521
ZMYM1
TYR
GABRG2
ATP2B3
CHRM5
SNX25
TTC9C
ANGPTL7
ANKRD13A
ATCAY
CDH18
FANK1
FIBCD1
FLYWCH1
GIMAP8
LYPD6B
MIRLET7D
MMRN1
MPZL3
N4BP3
NECAP1
PRPF38A
SCYL2
SETDB1
SLITRK1
SRRM4
TESMIN
TIPRL
TMEM74
TMOD3
TTLL6
VSTM2L
ZFHX2
ZNF518A
FEV
MIR128-1
RS1
ZNF232
AARS2
APOBEC2
ARMC2
C3ORF70
CCDC177
CCT6B
CDH19
CFAP77
CNEP1R1
CPNE6
DHRS7C
FAM174C
FAM89B
FCGR4
HDX
KDM5D
KLRC2
LINC00304
LRRN2

MEPE
MIR337
MIR345
NAIP5
NOTCH2NLA
RASGRP4
S1PR4
SMIM19
STX10
TRIM56
TRIM72
TSPAN10
TTC5
WDR91
YAE1
ZBTB25
ZNF600
ZNF606
SNRPD2
STK26
SYT17
VKORC1L1
CD1D2
MUP10
CSTAD
FLT3L
KLK1B5
LDLRAP1A
MIR26A
RIAN
SCAND2P
SPRY3
ZNF875
ADAM5
ARMH3
C11ORF1
C18ORF32
C4ORF48
CCDC144B
CCN2A
CHD5
DIMT1L
DNALI1
DTR
ELFN2
FASTKD5
FCAMR
FCER2
FKBP15
GNB1L
GOLGA8IP
HBA2
HDGFL1
ITLN2
JAML
KCNA4
LRRC14B
LRRC32

MBPA
MCM3AP-AS1
MIR493
MRPL58
NEUROD6
ODF3
OTOS
PANX3
POLR2M
POU6F2
PSMG4
RNASE10
SH2D2A
SH2D4B
SLC26A5
SMIM11A
SNORA61
SPATA3
TAF7L
TERC
TIMM50
UCN3
VEGFAB
VPS51
WDFY4
WDTC1
ZFAND1
ZFP263
ZFP277
ZFP428
ZFP472
ZFP760
ZNF333
ZNF347
ZNF575
ZNF93
ZSCAN26
ADCY8
ADD2
CAMKK1
SLC38A10
TOX2
UBE2W
CARTPT
ANKRD54
ARL14EP
ATP6V1G3
BIN2
CDX4
CHMP3
CKMT1A
CSMD2
DNAJC24
ECSCR
EME2
FOXB1
FUT3
GADD45AA

GKN2
GLYATL2
GOT1L1
GSKIP
HSC70
KIAA1109
KIRREL1
LANCL3
LPAR5
MIR451A
NEK11
NOP10
PARP10
PHF24
PRDM10
PRM2
REXO5
RNASEK
RPTN
SCN11A
SDHAF2
SERPINA1C
SNAPC2
SPATA24
SPECC1L
TLX1
TRIM4
TSR2
VGLL1
VSIG1
VWA3B
WDR24
CCDC9B
CCKBR
MS4A3
MVDA
SAH
ZFP33B
CLC
CLDNK
KRCP
MID1IP1L
MIR3911
MIR465
NDUFAB1A
SGO2A
TRIM43B
ZFP438
ABCD4
AJAP1
ATP8B3
C2CD5
CA13
CALCOCO2
CCNYL1
COPG2OS2
CXXC1
DOC2B

DPF2
GMEB1
GRAP2
INTS8
KCNN1
KNCN
KRTAP2-1
NAT5
NIM1K
OR5V1
PITHD1
RAVER1
RGD1304587
RGS6
RPGRIP1
SLITRK6
SNHG7
SOWAHA
STXBP5L
TBC1D20
UBQLN3
UNC13C
ZFP42
ALG13
CFHL1
EPHB4B
KDM4C
PDCL2
SEPTIN2
SERPINB9G
TMF1
TSEN15
USP11
CDC14C
GPR139
KRTAP1-3
HRH2
KLHL42
SHANK1
CALHM6
CBR2
CHRNG
FFAR2
NT5C3A
TULP3
AASDHPPT
CAMK1G
DIP2B
H2BC3
MROH1
SLC9B2
SMIM7
TRIP11
VPS54
GLULB
B3GNT6
FAM177A1
GP6

HBB-Y
KRT36
LCE3C
LENEP
LIP1
MIR302A
MIR434
MIR450A1
OTOA
PRH1
SERPINI2
THAP5
TMEM35B
UTP14B
ZFP597
ACTL7B
ADAM1A
BAIAP3
BUD13
C11ORF71
C5ORF15
CBLN4
CCDC62
CCR10
CD209B
CRYGB
CYP3A16
FAM220A
FDXACB1
FV1
G6PD2
GAL3ST2
JMJD7-PLA2G4B
KERA
KLHL30
KRT72
LRTOMT
MESD
MPIG6B
MROH2A
MYO15A
NCKAP5L
OCA2
PCDHGA5
PTCD2
RASSF10
RBM27
RCVRN
RGR
RNASE7
RNF166
SLC6A5
SLFN13
SLX1B
SNX11
SPINK13
THEMIS
TMEM217

TMEM52B
TRIM10
TTC9B
ZBTB8B
ZFP322A
ZNF280C
ZNF444
PROSER2
SLC25A27
ADAMTSL3
AK7
BCL2L15
CD72
CNFN
FLAD1
GRM7
LYPD3
MEPCE
MFSD3
OXSR1
PRPF4
PRRT2
PRSS3
RLN1
STAMBP
TOP3A
ZDBF2
CACNA1B
SLC17A6
ATXN7L3B
C6ORF62
DGKQ
KLF2A
PCDHB4
ADO
AK9
AREL1
C6ORF132
CDIN1
DMWD
EPGN
FAAP24
FAM162B
GADD45BA
GASK1A
GPHA2
H2AC7
H3C3
JMJD8
KLHL11
MAMDC4
MCF2
MCPT4
MON1B
MSGN1
NRL
PHETA1
PRDM9

PTGER1
RBPJL
RIPOR3
SHFL
SLC26A4
STIMATE
TCEANC2
TMC8
TMSB15B
TNFRSF13C
TNP1
TUT1
WDPCP
WDR17
XKR4
ZG16
ZNF330
ZNF711
FBXL16
HSCB
MSL3
MTSS2
OSCP1
SLC38A7
SMYD1
TRHR
UBR7
API5
ADAM4
CA6
CCDC63
CCL16
CD164L2
CLEC2G
IGL
MIR301B
UBXD2
ALDOCB
APOA1B
ART2B
BCL2A1C
BPIFA2
CCDC110
D16ERTD472E
DUSP27
FUT6
IL17C
MIR326
MST1L
NAIP1
OIP5-AS1
PIP5KL1
RIPPLY2
SIGLEC6
SNAI3-AS1
SYBL1
TBX22
TCRB

TMEM187
TMEM198
UGT1A6B
VWA2
WDR66
ZFP948
ZNF543
ZNF585B
AKR1A1A
C2ORF72
DYNLT5
LYG1
OTOP2
SMC1B
TMED7-TICAM2
ZFP212
ZFP771
TCTEX1
SERBP1A
MUC3
TOMM20A
APRIN
ETOHD2
DSG1C
SVS3A
CAPZA1
CNTF
GRM4
MIR467E
RHOX8
SPATA8
ACCSL
ANKDD1B
OLFM1B
PCP4A
PKD1L3
PRAMEF11
SLC22A26
ZFP687
ZNF28
ABRA
AKR1E2
ANAPC15
ANKRD36B
ARHGEF37
BORCS7
BROX
C9ORF72
CARD11
CCDC39
CFC1
CLEC1A
CTC1
CYP4X1
DCLK2
DCP1B
DDX55
DENND6B

DEPDC1A
DNAH10
EMC10
FBXL8
GPR50
GRM6
H4C1
IKZF3
JSRP1
LHX5
MATN4
MED17
MEDAG
MID1IP1A
MIR1915HG
MIR9-1
NKRF
NTSR2
POU3F2
PRMT8
RAB41
RAET1A
SCARNA12
SEL1L2
SIX2
SLC39A9
SLC50A1
SLC5A4A
SLC8A3
SNORA20
SPACA6
STRA8
TAC2
TAF2
THAP2
TMEM132B
TMEM201
TTLL9
UBN2
WT1-AS
ZNF654
ADGRG4
ALPPL2
ANKUB1
ARL6IP6
CFAP52
HLA-H
KRT26
LCE2C
LCN12
NHLRC1
PAX9
PRDM14
PRDM6
RBM12B
SAP130
TBCCD1
TRMT44

USP30
WDR89
ZFP354B
AKR1C6P
CGB7
SCGB1D1
TCEAL6
ASMTL
DYNC1LI1
FXYD7
GPATCH2
HCN3
HSPA12B
MRRF
MYO19
PTGDR2
RAVER2
ZNF644
BOD1L1
FABP9
AKR7A5
AMY2A
ANKRD13D
C4ORF19
CARD19
CCDC32
CFAP44
CGAS
CHST10
CHTOP
CXCL17
CYP4F18
DNAJC25
EEF1AKMT1
ELAPOR2
GUCA1B
H2BC18
HEPACAM
HEPACAM2
IL1RL2
KCNV1
KCTD4
KLK13
MIR674
NAXE
PARD6G
PCDHA9
PRDM11
PRSS27
SLC1A7
SNX20
SUSD5
TAF6L
TXNDC15
UBE3D
UBOX5
ZNF280D
ZNF44

AAR2
ASCL1A
CCDC83
DMAC2
DNAJB8
FNDC7
GAL3ST3
HCAR1
IARS
KRT33A
MKRN3
NWD1
PCDHGA12
RTL3
SLFN9
STEEP1
TOPAZ1
TPTE
TSSK6
URAH
WFIKKN2
ZFP617
ZFP706
ZFP800
ZFP810
ALDH9A1A.1
C11ORF24
COL25A1
FAM166B
FTHL17
H2-BL
KIAA0753
KRTAP3-1
LCE1E
MIR29B1
NAT16
PPP1R12C
PRKY
SPINDOC
PDE3A
FAM120A
GDF5
GTF2H3
MRAP2
RAD9A
SLC25A21
SPTA1
SCN9A
GABRA2
C5ORF34
CENPB
CEP20
FAM131A
FAM184B
GUCY2D
LRRC26
MOSMO
MYRFL

POLR3C
SIGLEC10
SULT1C3
TMEM121
VTA1
AP1G2
ASB3
ATMIN
CARD8
COX18
EPM2A
FAM163A
IL23R
INTS14
LAGE3
PLPP4
SLC6A7
SNPH
TRPM1
UTP23
VGLL2
DLGAP1
DYNC2H1
ST7L
XCR1
ZBTB24
COX19
EIF4EBP3
ELOVL4
PCDHA4
AQP6
ARR3
CCDC122
CYP4F39
DHDH.1
GDAP10
H4C5
KRT83
MIR369
MIR503HG
OVCA2
PCDHB9
PRSS32
R3HCC1
RNASE9
SDR16C6
SLC35E2
TCEAL2
TEPP
ZBED6CL
ZC3H12B
ZFP324
ZFP362
ZFP618
ZNF506
ZNF598
ZSWIM7
AARSD1

ARHGAP36
BTBD19
CDH20
DCPP3
H2AC14
INAFM1
KRT6C
MIR4435-2HG
MIR450B
PGBD3
S100A7A
SPATA33
TEFB
TRIM67
UGT8A
ZAK
ZFP451
ZFP787
ZNF275
ZP4
ZXDA
ACSL4A
CD209D
LILRA3
MAGEA4
MIR547
POTED
ZFP169
C6ORF52
CAPRIN1B
DIAP1
OPN5
REG1B
RTBDN
SNORA64
SOX19A
AKR1C21
GJA10
GNA15
MYEOV2
OLFR166
WDR88
ZFP568
ENO1B
LNK
P2RY5
PCDHB1
RXFP3
TNFRSFA
VCX3A
DGCR5
CORT
EXOSC3
GUCA1A
KCNC2
KLK10
ORAI3
PLAG1

WAC
SPRR2F
TRIM16L
AMER2
APOBR
ARPC1A
ATPSCKMT
BOD1
CCL18
CHRNB3
CNPY1
DNAH9
DTD2
ESS2
FAM83F
GRAP
KIF6
KIN
KIRREL2
LRCH2
MAGEL2
MIR99AHG
MOB3C
MRFAP1
NDUFAF8
PHOX2A
PHYHIPL
RC3H2
SENP8
SPTSSB
TRAF3IP1
TTC1
VMO1
ZNF573
TNFSF15
C1QTNF2
SIAH1
TTBK2
ACER1
ARMH4
ATG9A
ATP8B4
BTBD1
CLUAP1
CPLANE1
CYP2C22
DNHD1
DYDC2
FAM161B
GALNT17
GTPBP10
INTS13
KAP
KDELR1
LRFN1
MAP6D1
MIR202
MITD1

MYO16
PLB1
PLEKHA3
POGK
POMK
S100A7
SELENOH
SF3B6
SMIM24
SNHG29
TAS1R2
TECPR2
TMEM128
TTC33
TXK
ZFP14
ACTB1
AIRN
CAPNS2
H2-DMB2
H2-M2
IGHA
MYBPHA
MYOZ3
ZFP275
ADAM18
AKR1B15
ALKBH6
ARHGAP40
C1ORF159
FAM169B
FAM53A
FTH1A
GON7
HBQ1
MIR484
MIR505
MIRLET7G
MUCL3
NKPD1
NPSR1
PAXX
PGBD1
POU3F4
PRSS53
RDH12L
SNX32
TMEM92
VPS35L
ZNF202
ZNF428
ZNF684
DDX60L
TACR1
CARD9
FSD1L
PDE11A
SLC5A2

TACR3
TRIM5
RNF113A
ALKBH5
ALX4
ASB16
CCDC121
CCDC14
DNAJC30
ECRG4
FAM234A
GPBP1L1
IGFLR1
INTS5
KHDC4
KIF17
KLHL3
KLRG2
NPFFR2
PDE6A
RPL36AL
SYT15
THADA
UBP1
ZNF423
CCDC113
SLC18A1
SPDEF
ZMIZ2
ZXDC
MMP26
CTBP1-DT
GALNTL5
GPHB5
IGKV3-4
INSL5
OLFR114
SERPINA1F
SPDYE1
TMSB4Y
CLEC4B1
DHX32A
HMGA2-PS1
ID2A
LGTN
NEUROD6B
PRRXL1
RGS7BPB
RT1-A3
ZFP768
ASB15
C8ORF33
CLEC18B
CTRB2
G6PC2
GGTLC2
KLHL24B
KRTT1C19E

LGALS7B
NSFB
OTOR
PABPC1A
PCOTH
SERPINB6C
SLC5A4B
SNORA70
TRIM43
TTC24
TUBA4B
WASHC2
ZFP709
BRME1
C16ORF91
HLX1
KRT18P55
LCE1A2
NFE2L2B
TDO2A
TMEM211
ZFP160
ZFP58
ZNF471
BSX
F8A
ZFP697
DHFR2
NAT8F3
AKAP5
CACUL1
CCDC81
CCL21A
DNAH7
GADD45BB
GBP
GJD3
KLF11A
LOXHD1
MIR370
MIR638
MYH13
PCDHA3
PLAAT2
PYM1
QRICH2
RNASE2A
SERPINA1D
SPPL2B
SVOPL
TBATA
TCF24
TNFRSF26
ZNF236
ZNF777
ZNF831
ZSCAN25
EFCAB10

FAM155B
GPR61
HFM1
NR1D2A
NXPH2
PSMA8
TEX101
ZBTB9
FT
BSDC1
CNTN2
MEGF10
ADAMTS19
ASXL3
ATP8A2
BEND4
CCDC112
CIBAR1
CLEC2B
CRTC1
DUSP12
EML5
FASTKD3
GCFC2
GLYCAM1
HCRTR2
HS3ST5
KCNMB2
MCPT8
MIRLET7I
MRGPRF
NFKBIL1
PLAC1
PRR3
SLC23A3
SLC25A6
SLCO1C1
TADA1
TMEM161B
YTHDF1
ZBTB32
ZNF589
ZNF638
MSF
PRSS28
RNASEKB
DEFB18
MTUS1A
RPS27.2
IGSF21B
MIR378
MAPK14B
SAT1A.2
FIP1L1A
PRAC2
ELAVL2
GABRA6
IRX4A

MRGPRH
MT4
MTA2
DUXA
EPHA4B
HMP19
KIAA0087
KRTAP2-2
OR5K2
OR8D2
TUBA7L
FAUB
KRTAP9-3
DRR1
KRTAP10-10
RBMXL3
IFNA7
OR2T11
AIRE
LTB4R1
MIR452
ADAMTS17
ADCK3
APLF
C6ORF141
C9ORF24
CBLN2
CHRNA10
CHST4
CPAMD8
CRYBA1
ELP3
EPS15L1
FAM155A
FCER2A
HYPK
MIR379
MRM1
MYL5
NAA25
NEK5
PHKG1
SERPINB7
SMARCD3
SPRYD3
STOX1
TRIM62
TSR3
UST5R
ZFYVE9
ZNF85
ARHGAP5-AS1
CA14
CAPS
CCDC38
CCNB3
CTCFL
DENND11

GPRC5D
KCNH4
KIAA1549
LINGO3
LYPD5
MBOAT4
MC3R
MIGA2
NEU4
NT5C1B
OGFOD2
PROKR1
PRR32
RCE1
SAMD3
SNORC
TMEM181
TPGS1
TUSC1
ZBTB42
ZFP318
ZKSCAN2
ZKSCAN4
ZNF266
ZNF286A
ZNF597
ZSCAN22
ANG2
FAM104A
TDRD9
ZDHHC15
ZNF114
ZNF432
ZSCAN10
ESR2A
ALB1
ZP2.3
PYGMB
AGXTA
GNG13B
ZFP574
ANO9A
ATP1B1A
C17ORF67
CATSPER4
CD209G
MKRN2OS
SIAH1B
SNORA75
TRIM61
ACP4
ANTXRL
CRNN
CYP2A13
GPR152
PCDHA12
POM121L8P
SMGC

SPEM1
ST20
STKLD1
TRIML1
ZNF771
CASC3
ERLEC1
GCNA
HNRNPCL1
IGFL3
LSM1
PTGDSB.1
PXDNL
R3HDML
RBBP8NL
RMRP
SERPINA1L
SUMO4
ZFP120
ZFP512
ZNF440
ZSCAN4
BBS10
CAMKV
CCDC87
CLEC2I
CST11
CYP2B19
DMC1
EP400P1
ERV3-1
GLYR1
LCE2B
LGR6
LMX1B
MADCAM1
NKAIN3
PCDHGA7
RGSL1
SPDYA
SSC5D
TFG
TMEM200A
TRABD2B
ZNF431
SH3TC1
TNFRSF4
ZC3H12C
COA4
MIR148B
TRIM50
WFDC17
ACTRT2
B4GALNT3
BCLAF3
C1ORF52
CARNMT1
CCDC142

CCDC158
CYP2W1
FAM83E
GJA8
GPALPP1
HPF1
IL1RAPL2
JHY
KDF1
KRT77
LRRTM4
MPX
MTG2
PNMA8A
PODNL1
RAD9B
SLC16A6B
TMPRSS11D
UBAP1L
ZNF248
ZNF443
C2ORF15
CCDC163
CCZ1
CD160
DPH7
IFITM10
PMP2
POLN
PROSER3
SCRT2
SPAM1
ESR2B
SYNRG
AGTR1B
ANKLE1
C16ORF72
CRTAM
CWF19L2
CXCR7
DNAAF2
GVIN1
HSP70.3
ICAM4
KNOP1
LAYN
NMBR
TOR4A
AGBL4
ALPG
ANKRD35
ATXN7L3
CCDC157
H4C15
IPO8
LMNTD2
MPL
TCAF2

THUMPD2
TM4SF20
ZFP422
ABITRAM
ACAP1
ACTR3B
ALKAL2
ARMT1
ATP2C2
CALML3
CAPN12
CCDC24
CEP120
CRYGD
DZANK1
FAM98C
GBX1
GPRASP2
H1F10
H2BS1
HROB
IGIP
KRT73
LAX1
MYO1A
OTP
PAGE4
PIK3R3A
PPIP5K2
PPP1R37
PSPN
RIC8B
SHHA
SNHG4
SYNPR
TAC3
TIFAB
TINCR
TMEM167B
TRAPPC11
TREX2
VWDE
ZNF689
ALLC
SVS5
ZFP101
PPARB
RGSL2H
SLC16A9
ZFP598
ZFP689
ZFP775
ATP1B2B
CYM
H2-KE6
MIR129-1
MIR1306
SLC15A5

SLC22A18AS
SPON1A
SYPL
TUBA8L4
ZFP11
ZFP668
ZNF429
ABCB6A
ALDOCA
CDR1
MTMR8
PPP4R1L
PRG1
PRPS1L1
RAB39
RAET1L
RHOX9
SLC22A24
WBP2NL
ZFP142
ZFP692
ZNF354B
ATP6V1AB
ATP6V1BA
CST5
DGAT1A
FAM71F2
FAM83C
GLIPR1L1
GSTA.1
KCNA10
KIAA0408
LNP1
LYZL1
MOV10B.1
NUAK1A
PIRT
PITPNAA
SLC2A11L
TBL1Y
TSPO2
ZFP746
AMD2
CCDC89
CYP2AA12
EXOC3L1
GAREM2
REREB
SLC4A9
TMEM114
TUBA3C
UFSP1
ZFP236
ZFP536
ZNF320
ZNF687
ZNF846
NMRK2

ADAM33
CATS
CDKL1
CLCNKB
CYB561D1
GDPD3
GJC2
GPR17
GST-4
SCD5
ZFP467
CAR7
FABP7A
IL36RN
KLRA22
MIR361
PTF1A
ALKBH4
ATP4B
BARX1
C1ORF53
CBLN3
CCDC181
CTXN3
DNAH3
FAM98B
GLRA2
IPW
KIAA0319
METTL14
MTMR1
PCDHB14
RGD1305347
SMLR1
TMED1
TSPAN32
URM1
USP35
WTIP
ZNF131
ZNF260
ZNF326
ZNF609
ZNF746
ZNF77
ATRAID
CMTM5
FAM151A
IGLON5
RUBCNL
SERHL2
SLC5A10
TTC30A
XKR6
ZC3H12D
ZMAT4
TAS2R108
NDR1

PLCH2A
SCN3A
MIR293
RN4.5S
CACNG3B
PRPS1L3
UBAP2B
ATP8B5
CRLF2
FPGT
HERPUD2
NDNF
PTBP1A
SNORD118
TCFAP2B
ZNF451
CALY
EIF1AD
MAMLD1
RPS7P5
SYT1A
VSIG10
ZC3HAV1L
ZFPM2A
ZNF224
BMPR2A
CNBPA
FGF13A
MTVR2
NSFA
SNX10A
SP8B
H2-D4
UBE2DNL
HBB-BT
MFGE8A
RORAA
SYT2A
KIF2B
MAGEB4
P2RX4A
PPP1CAB
PRAC1
RDH10A
SMCO2
XPO1B
ANP32D
CLDN15LA
KRT91
RPL23AP53
SELENOJ
ZFP398
LGALS3B
PIWIL3
R74862
RAB40A
SCARNA16
STON1-GTF2A1L

TEX55
CATSPERG2
DLEU2L
NOTCH1B
OR7E91P
CT55
CYP27A7
FAM9A
LUZP4
TP53TG5
TRIM51
KCNJ15
PLA2
GLRA4
MCHR2
ZFP641
AHR2A
BLF
C2CD4D
GPX4B
RGD1310507
TRIM42
APOBEC3
CYP2B12
EDDM3B
GSDMEB
KLK1B24
RPE65A
SDR42E2
ZFP213
ZFP518A
ZNF544
ACSL1B
BTLA
CLNK
CNPPD1
COX5AA
HEPHL1
LILRB4A
MIR1275
MIR28
MOGAT3
SLC25A15B
TPI1B
ZNF250
ACTR3C
AGBL3
ARL17A
C7ORF57
CACTIN
CBWD3
CIB4
CYP2K21
D5ERTD579E
EHBP1L1B
ELNB
FCNB
GDPGP1

GJA1B
GSTT1A
HN1L
ITPR1A
KLF14
LAMTOR4
LCE3D
LILRB1
LINC01588
MAMSTR
MIR30B
MORC2-AS1
MUC19
PCDHGA8
PDZD3A
PLA2G4D
PLA2G4F
RHPN1
RNU11
RSBN1L
RTN4RL2B
SH2D1B
SHARPIN
SLA2
SLC38A8
SLCO1D1
SOHLH2
SPACA5
STK24A
TBX4
TLDC2
TMC3
TMEM248
TMEM72
TNFRSF23
WDR20
XCL2
YBEY
ZFP871
ZNF713
CNTN5
COL19A1
CSL
FAXC
FYB2
GPX1B
GSX2
KLHL35
LDLRAD1
LY6H
MAP3K21
METAP1D
MIR335
MIR374A
NDST4
OXLD1
PRR4
RTL5

TLX3
TSPEAR
UFSP2
UNC45A
ZNF165
ZNF75D
GALNT13
HLA-DQA1
IL17RC
SEMA6B
SUPT16H
ZMYND15
OPRK1
NUP62
ABCA13
ADGRB1
FAM193A
FXR2
ODAD4
MTMR14
NUDT11
REM2
GRIFIN
EMC7
RHOX5
ACR
AIPL1
C1ORF226
CFAP43
CYP3A
FAM71E1
LY6G6D
MIR374B
MIR483
NME8
NPAS4
NRG3
PCDHB7
SRY
UPK3B
ZNF37A
ZNF385D
ZSCAN20
ARMC7
C2ORF68
C8ORF58
CA1
CA11
CDPF1
HTR3B
TMEM216
UEVLD
USH1G
ZNF493
TMEM252
ASPHD2
BGLAP3
CNOT3

CYP2D2
LY6K
MIR449C
MYH8
RX3
SAP25
SIN3B
YIPF4
FBXO40
PEG12
PTCD1
SBP
USP29
ZFP827
CXCL12A
ICN
MIR203A
MIR489
MMP23B
POTEE
RGD1310587
RT1-N1
SPRR2C
ADGRF3
ASCL3
C11ORF86
C16ORF70
COX7A2A
FBXO48
GIMAP9
GIMD1
GPRIN2
KRT84
OPN1SW
PMS2P5
SPANXD
ZNF496
ZNF850
CYGB1
FAM181A
FAM86B1
FBXL21P
IGFL1
IGK
KEAP1B
LCE3A
LINC00324
MROH6
PMT
PNMA6A
RGD1310352
SLC49A3
TAFA3
TMEM31
TMEM39B
ZNF329
ZNF354C
ZNF407

ZNF626
ZNF691
ZNF700
ADGRD2
ASAH2B
BCYRN1
CCDC191
LRRC37A3
PAPOLB
RFX8
TMEM250
TSSK2
FOLH1B
TVP23B
ACTBL2
ANO5
ATOH7
BRD9
CERS1
FAM78B
FAM83B
FBRS
MSH4
NDUFAF7
OPA3
PIH1D2
PUDP
ZDHHC12
ZNF483
ZNF91
ZSWIM8
AMZ1
ANOS1
CRACD
DAW1
GANC
KRT81
ODR4
SRSF12
TMEM129
TMEM219
TRAPPC12
TRIM66
WNK3
ZNF254
ZNF37BP
MACC1
HCAR3
RPGRIP1L
TDRKH
MIR181A-1
RAET1D
CIR
MIR572
CA10A
SNORD49A
TRY10
MIR194-2

SNORD38A
ANK2B
ATP5MC3
DCUN1D1
KLHL41
NRXN1A
PIGC
RFX3
SMARCA5-PS
TFAP2B
BMP7A
DIRC1
MTB
SPSB3A
CES2G
CYP2D1
HSD17B12B
MIR331
MUP20
OLR1375
PUSL1
SNORD59A
TCFL1
ABCG3
C22ORF46
DENND4B
DLC
GLUD1B
HLA-DOA
HSP70L
LINS1
MEF2B
MIR676
NOA1
PERM1
PIK3R6
PRELID1
RFT1
SRPR
TCIRG1B
ZBED2
A2ML1
ALDH2.1
APOBEC4
CLCA4
DYNLRB2
EMR4
GSG1L
JADE3
JAKMIP3
LCE2D
NAIP6
SPDYC
TMEM178A
UCA1
ZBTB49
CLEC-3
H2BC20P

NDUFS8A
OR2H2
SNORD116-13
SSX3
WASH2P
DNMT3BB.3
EXDL2
HAND2-AS1
MIR16-1
SLC16A9B
ZNF137P
AK6
AKAIN1
AQP7P2
C1ORF68
COL1A1A
DDTL
KIAA1456
MT1IP
PCDHB17
PSG3
RTL10
SLC25A31
ANXA1B
C1QTNF8
CP110
F13A1B
H2-EA
METTL24
OR8I2
PCDHB21
PGPEP1L
RTN2B
SLC25A38A
SOCS1A
VTG3
ZNF177
ZNF439
ZNF577
AFG3L1P
C3ORF18
C6ORF136
FEZF1
FHL5
GRID2IP
HGC6.3
ITLN3
KLK1B16
MIR656
NAA11
NOBOX
OVCH2
PATL2
RPL26L1
SH2D6
SLC22A7A
SMIM12
TPMT.2

ZFP316
ZNF767P
ABCC6P1
C6ORF89
IGFL2
MIR128-2
PSAPL1
TRHR2
TFAP2D
PLCXD3
MTERF1
NFATC2IP
AHR2
HMGCRA
IL20
NDST3
OTOG
PDZD7
PPP1R3E
RGD1309534
SIGLECH
CFAP70
CLHC1
DBNDD1
EML6
HOXD13
JAKMIP2
JPH4
KCNH8
L3MBTL4
NRSN2
NUDT10
SHISAL1
TMEM132E
WRAP53
ZC3H10
ZNF264
PLA2G3
CSH1
ARL13A
CD209C
KRT42
LILRA2
DSG4
ERMARD
IFNK
MIR30C2
RGD1309350
ROM1B
ZFP35
ABP1
ARMC12
CFAP221
CYP2D11
DUOXA2
KIAA2026
LINC00597
LY6B

METTL25
RLN3
RNF175
RSPH10B
SFTPA2
SIRPB2
SLC2A15B
SMIM10L2A
ZG16B
ZNF212
ATF5B
BRF1A
FAM151B
FRRS1L
H1-1
HPDA
LHFPL1
MFSD12A
MIR381
NRN1L
PFKMA
PSG1
SEPW1
TEX37
TSKS
ACRV1
CCDC154
CCDC178
CCDC74B
CLDND2
HMGXB3
JMJD7
LRRC18
NLRP7
NPM2
PCDHGA1
RHCGL1
TVP23C
ZNF426
ZNF614
ARFGEF2
CYP2A3
HBZ
LEKR1
LY6G5B
PCDHB8
PLA2G2F
POU1F1
PRDM15
SMG8
TACR2
ZFP28
ZPLD1
BMT2
CCDC153
CEACAM5
DNLZ
FAM131B

FAM71F1
FOXO6
FRA10AC1
MIR320A
MT1HL1
MUSK
PTCHD1
RADX
TCFL5
TMEM223
TMEM38A
VPS26B
WASHC2C
ZFP57
CFAP69
ENGASE
GRIP2
KCNH6
MYORG
RNF32
SLC6A15
SYNPO2L
TOP2
KCNC3
PRIMA1
CDHR1
NPRL3
FOXF2A
MACROH2A3
CATSPER2
NTN1A
UBE2E3
ARHGEF11
ERMN
FAM177B
MIR198
MIR1981
POLR1G
AMIGO1
ARPIN
ARXES1
BLOC1S3
BTBD17
CYP4F12
DOP1A
FAM171A2
FANCM
MSANTD4
NBAS
NKAIN2
ODAD2
RNF41
RNVU1-7
SLC36A4
SLC9A5
SLIRP
SNHG6
STK36

TBC1D32
TMEM242
UNK
ZNF721
SMR2
SNORA2A
ATP5MC3B
KIR2DL3
MUP18
REG3D
ZNF229
CFAP54
H2AC22
MIR3473
MIR540
SPPL2C
HER6
KLRA12
RT1-DMB
SERPINB3A
TMEM253
ZFP384
CYP2AA2
GLT6D1
MIR302B
MIR412
RNASE13
ZFP560
ZNF665
BTN1A1
CRYBB1L3
EBLN2
FZD8A
IGKV6-14
LDLRAD2
LECT2L
OR7E13P
SERPINA3A
SNORD50A
ATP2A2A
EHD1A
EPHX5
FNDC10
FREM1
GPR89
LRRC19
MIR629
MROH8
NTD5
OVCH1
SLC25A25A
SLFN1
ZNF449
C22ORF39
C6ORF118
CCDC190
CD300C
CDCP2

CNTNAP5B
ENO4
FAM186B
GBP8
GML2
GPR31
GRCC10
IFITM7
IFNA4
IL31
LRP5L
MIR487B
MSX3
PABIR1
PSMG3-AS1
PTTG3P
SEMG1
SLC22A29
SMTNL1
SRSF8
TAAR2
TERB1
TMEM200B
UGT1B5
ZBED5-AS1
ZFP329
ZKSCAN6
ZNF319
ZNF414
ZNF524
AKAP3
C1ORF105
C22ORF23
DRC7
EGFL8
HSP90AA1.2
KIAA1958
KRT74
LGALS3BP.1
MINAR1
MINAR2
NOX3
OC90
PCDHB10
SERF1
SERINC4
SERPINB13
SLC2A11B
SMIM30
SNORD17
TAS2R4
TCL1A
TMEM61
UGT5A2
VRTN
ZBBX
ZFP710
ZNF776

ADAM29
AKAP4
AP5B1
C8ORF74
DUSP21
GLRXP3
MSC-AS1
ZFP748
ZNF30
EML2-AS1
ENDOV
FOXB2
OR5P3
RNF222
SNORD15B
KC6
GRK1
APOL2
EFCAB1
IL17F
MAU2
RETNLB
TFA
ZP2
AP1AR
H2AC12
H2AC19
KRT78
MAP9
MOB2
POU2F3
SNHG5
TRMT1L
ZBTB39
PPOX
SLC5A7
HTR2C
LOXE3
MBL-1
GBP9
CYP19A1A
ABHD12B
RWDD4
SNORD123
GP1BA
MIR652
MIR760
PIGG
ZFP354C
FPR3
IL27
PABPN1L
SERPINA3F
UTS2B
ZNF555
ABHD18
ACSBG2
AGBL1

AGXTB
AHSA2P
AMZ2P1
CCDC102B
EFHC2
ERAP2
GIPC3
H2AC17
LATS1
MEAK7
MIR346
MKS1
PMCH
PRSS57
RBM41
SAMD13
SFRS6
ST20-AS1
TEX15
TMPRSS13
TMSB15B2
ZNF225
ZNF623
CKMT1B
CLEC12B
CNGA2
DISP3
GPR6
KCNK18
MIR100HG
MYLIPB
PAAF1
PPAP2B
SFRS3
SPATA21
SPATA22
SPO11
SQRDL
ZNF197
ZNF365
ZNF415
ZNF549
CLVS2
DPY19L2P2
MYO3A
STAG3L1
ZNF76
SEC14L5
CYP4A6
AHR1B
ZFP113
COA1
HAAF
C1ORF112
CD8B
MGAT5B
MICU2
MIR126B

RBFA
RBM42
SLB
ASIC4
CCNQ
FAM174B
FAM221A
FGD2
INTS4
LIN28B
MORN4
TMEM254
UBXN8
MIR679
DP1
HNRNPA0B
MAGEC1
ZFP111
ATP2B1B
FGFBP2
LGALS6
OAS1G
TRIM60
COL9A1A
DEDD1
HIBADHB
KLRA9
KRTAP8-1
OXCT2B
PTGDSB.2
SULT3ST4
TNXBA
WFDC13
ABCG3L2
HMGB1P1
IGSF22
MFAP1B
PRSS42
SLCO1A3
ADKB
GZMF
LINC00652
MIR216A
PNMAL2
UBE2D2A
USP41
ZFP358
CYCT
GST-1
LDB1A
NME1-NME2
RDH18-PS
ZNF821
APOL10B
CRP3
GJA6
GUCY1A3
IQSEC3A

MIR217
NFIXB
PLOD1A
RHOX10
RIC1
RS1A
TLCD3B
TMSB
ACTL8
C17ORF59
C1ORF162
COX8C
CTSL.1
DRL
FAM228B
FMN2B
ITGB2L
LOH12CR2
MILL2
MIR101C
MIR1-1HG
REP15
SH2D1A
SMCO1
TCEANC
TMEM241
ZBTB37
ZFP317
C3ORF33
FAM69B
FDPSP2
HIGD1B
ITPRIPL1
KCNV2
KIF19
LRRIQ4
MMP21
NPW
ODAD1
PGK2
PTX4
SPATC1L
ZFP941
ZNF34
ZNF775
C8ORF31
CEL.1
FCRLB
FTL2-PS
GPR149
KCNB2
LINC00052
LINGO4
MIR9-3HG
PEDS1-UBE2V1
PNP4B
PPIAL4A
PROP1

RALAB
SHISA7
SOX9B
UMODL1
USP32P2
ZNF121
ZNF550
CDK15
CEACAM4
CTU1
CYP2AA1
GBGT1
NRAV
PROX3
TMEM150B
TMPRSS12
ZNF3
ZNF800
APOBEC3H
CTBP2A
D1PAS1
IZUMO2
RETSATL
SAGE1
D10WSU102E
MIR6956
GPM6BA
MIR6328
RBM48
ATP8
CCDC174
DEFB15
EMSY
GNAT3
KCNK9
MIR1190
MTNR1B
SLC4A5
SLCO1A5
USP12B
ZKSCAN5
CCSER2A
CNNM1
CYSRT1
ERICH1
FANCF
NUDT2
PRAM1
RPRML
ZFP709L1
BRD1A
OXR1B
SPRED2A
STK17AL
PRAMEF7
DEFB35
GNSB
DEFA-RS12

EEF1A1A
MUP-PS16
RAB10OS
RHOGD
BRIP1OS
H2-M10.1
SIGLECF
ZNF106A
MSP-113
SGSM1B
SLC38A3A
SV2BA
ALDH3B3
ARVCFA
MIR196A1
NDUFA9A
PHB2A
ZNF41-PS
MCTP2B
OR10G7
SUMO1P1
TAS2R140
RGD1565356
SIL
CYMP
RAB3AB
TRPC4
MUP-PS14
SLC17A6A
DCD
LINC00326
OR2A7
SCP2-PS2
ZNF408
OR2G6
PRR23B
PVALEF
ZNF146
BTR02
CBLIF
CXXC4
DCTN1B
DRAXIN
HSPA7
LCA5
PAPPA-AS1
SETD1A
SNORA36C
TREM3
ZNF592
ZNF681
AKNAD1
CCDC78
CRFB2
MIR671
OCM
P2RY11
POU5F2

RAB6D
RGS9BP
RIIAD1
ZNF324
C2ORF69
C9ORF152
EMX2OS
GTSF1
GUCY2F
HMX3
KCTD16
MAIP1
MIR363
MYPOP
NR2E3
OXGR1
SERPINB11
STARD7-AS1
ZCRB1
ZFY
ZNF384
ZNF551
ZYG11A
GCH2
IGFL2-AS1
KCNAB3
LINC00543
LINC01597
MCMDC2
ODF1
SHISA6
STX19
TSNARE1
CATSPERG
PMCHL2
ZSWIM1
TMEM95
BCL11B
TSP1
OBP1F
GABARAPL3
H2-Q4
ZFP429
CYP3A37
MIR196A-1
ZNF655
BTBD8
CYP3A62
DRC3
FAM216A
IGHMBP2
TRP63
ZFP125
CLDND
CSHL1
HIGD1C
NXPH4
OVOL2

PART1
WFDC16
ZNF24
ABCA1B
FAM228A
SNORA54
HLA-J
ASB13A.2
CAR10
CYT1
MGAA
SOX2OT
CGB5
FUT5
GOT2B
GPR116
HIST2H2AA2
KLRA2
MIR762
MUG-PS1
OLFR1260
OLFR1383
SLC25A32A
TNXA
TTC30A1
ZFP930
DNMT3AB
MCIDAS
RAET1G
VTNA
ALDH1A4
BZW1A
C9ORF129
DEFA4
DGCR6L
HSD11B1LA
KIAA1614
MYCNOS
SCGB1D4
TOB2P1
ZFP865
A
ASB16-AS1
DEFB132
DYDC1
GST-5
H4C17
LINC00473
LINC01140
MHC1ZBA
NAPAA
PSORS1C1
RHOBTB2B
RNASEL3
SULF2A
VIRMA
ZFP579
C11ORF45

C6ORF15
FOXQ1A
IGLL3P
KRTAP2-4
MAPK1IP1
PCDHGB5
SLC25A53
TEDDM1
UBL4B
ZNF541
ZNF671
ADM5
CCDC159
CELA2B
CNO
H1F1
MIR592
PPDPFL
PRINS
TAL2
TEX13A
TRIM40
XGB
ALDOART1
C1QL4
CAR12
CST1
FAM219B
KIF25
MEIS3P1
MIR744
PCDHGA10
RSPH3
SLC35G3
TKTL2
TRIM65
BPIFB3
DBIL5
DNMT3BB.1
KRBA2
LAIR2
LINC00525
LINC00638
LRRC37B
MOCS3
MTRES1
OAZ2B
OPALIN
PTPN20
SNORA65
VWA3A
ZFP185
ZNF133
ZNF730
ZNF761
CABP7
GSDMB
HBD

KCTD18
MCRIP1
PCDHGB6
TIGD3
ZNF273
ZFP143
INO80D
NTF4
QARS1
TNFA
BARHL1
GHSR
NKX2-2
PLCH1
SLC24A4
TRPA1
GPR55
SUPT20
UFL1
FANCE
HIST1H2BQ
HSP-16.1
TNFSF18
KLRA16
MIR32
NLRP5
PER1B
TTN.2
ZNF10
FAM183B
FNDC11
MFAP1
MIR187
MIR877
PGA5
PRRT4
SIMC1
ZNF234
ACP7
BRSK1
C12ORF75
COPRS
CRISP1
LCE1B
MCPT2
MIR376C
PPP1R36
RNF151
H3C11
MCM9
R3HCC1L
HRCT1
MIR125B-1
SOD-2
CYC
PRLH
ERICH6
PCDHA5

SOX11A
SULT2ST2
AKAP17A
C1ORF50
HERC2P2
MIR350
MS4A2
RPS26L
DEFBL1
FAM122A
TEFA
TPTEP1
ABCA10
CCDC166
MIR463
CALR3B
COL6A4
DCST2
DEFA-RS2
EEF1DB
IFITM4P
MIR377
PPDPFB
ZNF135
AKP3
APEG3
EXOC3L
GTF2IRD2B
ICAL1
NDRG1B
NKX2-9
PSG19
RNU6ATAC
S100A10B
UCHL4
UQCR
XLR4B
ADRB2B
BACH1A
BCO2A
C20ORF96
C9ORF50
CYP4V7
FAM81B
GJA9B
GPR78
HULC
MIR92A1
NAMPT1
PLD1B
RFPL3S
RGD1306271
RXFP4
SELENOP2
SLC5A4
SPECC1LB
SSTR5-AS1
TMPRSS13A

TXLNBB
XIRP2A
ZNF720
ANP32A-IT1
CLPSL2
CST2
CYP2AD2
DGAT2L6
FAM86B3P
FLG2
FOXR1
GJB7
KIR3DL2
MIR372
PLA2G4E
PPEF2
RPS2P32
SLC36A3
SOX19B
TSBP1
VTGA2.L
WDR64
WEE2
ANKRD30A
C1ORF43
CAPZA3
CFHR5
H2-Q8
KRTAP5-5
LCE3E
MAK10
NOP14-AS1
NPFFR2A
NPY6R
RFWD2
WDR49
ZFP39
CALHM1
GYPB
HAPLN1A
MIR181B1
PCDHGC5
PTTG2
RNU12
C9ORF40
EVX2
GSC2
HOXA6
METTL21C
MSX2P1
SLCO1A6
SMIM20
TRNAU1AP
UST4R
ZFP629
ZFP830
ADGRA1
ANGPTL5

ATP13A5
CASP5
CCAR2
DNAJB3
FRS3
HEXD
HRH3
MIR7-3HG
MRGPRG
RTL9
ZNF100
ADAM7
CYP2K19
NCR3
PRADC1
SFT2D3
SYT11
V
ALOX15B
H2BC17
NRIP2
RBP5
TAF8
VWC2
VWC2L
ZFR2
ZNF211
BTBD16
CYP2P7
NEURL4
PTPRV
SLC52A3
ADAMTS3
CAVIN4
CNTN4
RGD1564865
C4ORF3
H2BC1
MZB1
XIRP2
C3ORF38
FAM204A
GPX-5
LRRTM3
MYZAP
NOX5
RIBC1
TMEM121B
TMEM198B
TRIR
TXLNGY
VSX2
CLTCL1
FAAP100
KXD1
RFX6
TECRL
TOGARAM2

ZFAND5A
ZNF557
JMJD4
NAALADL2
RNF214
ZFP69B
CARMIL3
OTOGL
PPARDA
TPRB
CYP2K5
DAK
KIR3DL1
ZNF799
TP53AIP1
CAMK2D2
DLG5A
FHOD3B
CCDC37
HMGB1A
KRTAP19-1
MSANTD1
PARP
SMYD2A
FOXO3B
NUCKS1B
OLR701
SPINK6
SULT1A4
TXNDC8
ABCB11A
CLDN3D
DPYSL5
FAM187B
ITM2CB
KBTBD13
LIMS3
MTHFD1A
RAB33A
RAP1AA
RTL4
SCG2A
ZFP202
ANKRD36BP1
APBA1B
C8ORF44
FLVCR2B
HER7
KLK1B22
PRX
SUZ12P1
AQP7P1
C6ORF226
CCDC140
CCL23
CFAP299
DA2-19
DUSP22B

JUPB
LDHBA
MYST2
N6AMT2
NPSN
PPIAA
PRPS1B
RTN4B
SCARNA9
SFXN5B
SLC7A5P2
SNORA80A
TMEM273
TRPM4A
ZNF536
C3ORF22
CA4A
CYP2C53-PS
GRHPRB
OLFM2A
PHACTR3A
PRDM8B
SMCO3
SSMEM1
TEX13B
TXNIPB
WDYHV1
ESPNL
KLHL38
LRRC74A
NAPSB
ODF3L1
PPIEL
RBPMS2B
SNORA38B
SPACA4
SSX8P
TCEAL5
TMX3A
UBE2Q2P1
VTG7
ZNF610
BDNF-AS
IQCN
NBEAA
OLFR644
TCEAL4
WDR97
ZFP770
ZNF391
GK2
NOLA3
ONECUT3
TMC2
VOM1R76
C2ORF74
CIRBPB
HSP-16.2

PYCR3
RND3A
ABHD10A
ADAMTS15A
ARL9
ASIC1B
NRDE2
SERPINB9F
WDR79
ZCWPW1
ZNF274
IL36G
INKA1B
PLEKHD1
RGD1565695
SLC22A16
ATP5G3
BSP
CACNG3
CFAP65
FAAH2A
FSCN1A
HDLBPB
IGG-2A
NAV2B
NUDT17
PPP1R9ALB
TMEM191B
UCKL1
ZNF436-AS1
ADA2
EFCAB6
IGK-V8
KRT92
LMOD2
RIMS1A
RMC1
SUZ12A
USB1
ZNF519
ZNF672
ASCC1
EPHB4A
LMO4B
MNF1
MROH7
SLAMF8
SNORD73A
STXBP1B
ATP6AP1L
GON4L
KIF1AB
ZFP108
ADAM28
BACH
CES1H
SCN1BA
CCDC192

EFNB2B
HTRA1A
MEI-41
MEI-9
MSP-10
MSP-51
PRAMEL6
ZFP313
AIR
BIN3-IT1
BTR12
KDM2BB
MIR103A2
NMT1A
OTUD5A
AKR7A2P1
CYP2P8
DEFB123
GPR148
MIR1910
OLFR55
PROM1A
DNAJC3A
PALD1A
UBE2D2L
CST9
MUP-PS12
OR5H1
SCGB1B2
SEMA3AA
C1ORF100
CLCA3P
KRTAP6-3
MIRLET7A1
MN1B
PEAK3
REG1CP
TSPAN16
OR5H14
BPESC1
KRTAP12-3
KRTAP4-2
KRTAP4-5
LCE5A
LINC00242
LINC01555
NRXN1B
OR2J2
OR51V1
OR6K3
OR8B12
SNORD115-5
SSX5
TIEG3
TMEM176L.2
ZNF560
DEFB115
DULLARD

MIR627
OR5A2
DCLK1B
MIR181B2
PRL2C2
SLC34A1
SOUL5
MIR663AHG
NCRUPAR
SNORA59A
SNORA59B
ZFP874A
ADGRF4
LINC00588
LINC01508
LUARIS
OR12D3
PIWIL4
TPRG1L
ZFP2
ZNF833P
BRK1
CDK11A
GTF2IRD1P1
INE2
MIR323
TCTN3
TMEM251
ZIK1
ZNF678
FBXO7
MIR133A1
SMG9
TEX14
ZNF124
ZNF518B
NMRAL2P
PEX10
PLPP7
RLN2
SAMSN1A
SNORA28
THAP6
VOF16
CDRT4
CRYBGX
MSANTD2
NLRP2
PRSS21
SPANXA1
SULT3ST3
AHRRB
FAM216B
GATA2A
KLRA8
OLFR544
IL11A
MEP1A.2

MIR1247
ACP5A
ARHGAP29B
CARMN
ENO1A
EPDL2
HCG18
LRRC16A
MIR216B
RSPO4
YY2
ZNF2
DPPA5
GCNT7
RGPD5
THAP8
TTC34
AHR1A
BHLHA9
BRE
MTMR9LP
NAALADL1
PPP1R3DB
PRAMEF12
TBX10
TMEM232
ZNF230
CCDC73
GSTM.1
KLLN
LGALS2A
SAGA
SMIM10L2B
TAAR5
TSIX
WDR67
ZNF445
DBH-AS1
EYS
KLK12
LINC02693
OSBP
SULT1C4
THEG
USP28
ZFP94
ZNF677
GNAS-AS1
GNG10
H4C13
IDI2
NKX2-4
RNR2
TPM4A
C10ORF95
C3P1
ZNF568
ZFP326

SOD-3
C18ORF25
CENATAC
TRIM46
C4ORF33
EPOP
HMOX1A
ZKSCAN7
C12ORF4
EIF2D
MIAT
PLPPR5
RAMAC
SNF8
TTC29
CCDC74A
PRRT3
TMEM220
TTC22
LRFN5
MIR3102
CHIL1
RNVU1-18
ARR3A
LRATA
MIR449A
MRLC2
SERPINA3H
TRIQK
CCIN
CNTD1
GPR174
MALSU1
MFSD14B
MIR590
SLC7A13
VIT-5
WDCP
ZSCAN18
BORCS8
CDC2L1
CRYGF
GPR143
MMEL1
MS4A10
MYCS
PPP1R26
SLC35D3
ZFP319
ZFP652
ZNF331
ADGRE2
ATF7IP2
BMI1A
DNAI2
IFT43
KIAA0895L
NFAM1

PRAMEF8
SBK2
SMR3A
ZFP41
ZFP758
ZNF358
ZNF804A
APOA4B.1
ARMCX6
C2CD4B
CEBP1
CLRN1
HES7
PRL6A1
SEPTIN10
SLC6A19A.1
SPANXC
SPATA1
TYMSOS
USP9Y
ZFP51
ZFP945
ETV5B
FKBP1AB
GLI4
LINC00339
REG2
RGD1305464
STC1L
ZNF25
AGPAT4-IT1
AHRRA
C16ORF89
CYP2AA3
FAM120AOS
FCRL1
OPN4
SLC9B1
SNORA73B
ZNF19
CCNI2
DRD3
FAM104B
FAM209B
GLIPR1L2
SRARP
TSPEAR-AS2
ZFP516
CTSJ
CYP3C1
LARGE
LRFN2
MARCHF10
SCARNA2
SERTAD4-AS1
TAS2R10
TSACC
HN1

PGAM4
RSPH6A
TMEM215
ACSM2B
FAM133A
PKD1L1
VDRB
ZNF45
MIR3074-1
CYP8B
BIRC5B
C7ORF26
EFHB
ASIC3
CA5B
HARBI1
LYPD2
MUP17
NEMP2
PAQR5A
TRPC7
C1QL3
DEFB12
FSCN2
OLR649
PCP2
SRSF3B
SYCP2
CD33
EID2B
ERMAP
KRTAP20-2
NRGNA
SLCO6C1
TUBA4L
KIAA1671
OLR288
PLK
GSTP3
NECTIN3B
ORG
USP49
CHIA.3
MIR1
MUP16
NSD1A
OLR544
MROH9
NMES1
RB
UGT2B3
VMN1R51
GNAO1B
IP6K2A
IP6K2B
KIR2DL1
MGAT1A
PROM1B

ATP6V1C1A
CUTAL
KSR1B
MIR193
MSI2B
NOP9
MARCKSA
MYCLB
RFX1B
RNF225
SCARNA15
ACAP3A
APOL10A
HNRNPA3P1
MICALL2B
NTNG2A
PPP1R14BA
SNORA38
SNU13A
SOX6OS
SULT2A3
CYP2C66
MIR466B-1
SLC6A20A
ZAR1L
AMTN
ANXA2R
CRY1A
CYP3A73
EEF1A1L1
FCRL3
G6PC1A.2
GLSB
MIR667
SNORD54
ZBTB2A
ZNF648
C19ORF18
CLDNA
DDX43
DEFB41
DPY19L2P4
HNRNPH1L
MSTNB
RASGEF1BA
SPANXA2-OT1
SYNE1B
ADH8B
ATP1A3A
CD27-AS1
DIO3OS
HSD17B12A
KRTAP17-1
LMAN2LB
MARCKSL1B
MIR576
OR5W2
OR9G4

SHOX
SNHG3
TMEM88B
TSPYL6
ZCCHC13
APOA4B.3
ARIH1L
C11ORF87
FOXN3-AS1
FUCA
GPRC6A
GYG1A
KCNE1L
LPPR1
OLFR281
SNORD114-1
SUB1B
TGM7
TMEM35
ZFP273
ZNF747
C17ORF58
CTSLB
HIST2H2L
IL17B
KCTD19
LVRN
OPN1MW2
PAGE5
TARP
TUBA3E
ZKSCAN17
ARIH2OS
C3ORF80
CEBPZOS
CENPBD1
CYREN
DEFB19
DSG1
GPR171
KIAA1755
MMP20
NTAQ1
NYX
PRR14L
SNORA29
TSPAN7B
ZNF788P
B3GNT4
C10ORF88
C15ORF61
CRCT1
CTS7
HS3ST4
HSD11B1L
INSRA
KRT9
LINC00174

MIR518F
PPA1B
QKIA
RGD1559459
SPATA46
ZNF740B
ZNF883
CCL19B
CSMD3
CYORF15A
DHRS7L1
EAR4
FRMPD2
GSP-4
KIF26BA
MAP3K14-AS1
MORC1
MSP-33
MSP-45
MSP-49
MSP-56
MSP-57
MSP-64
MSP-81
NEURL1AA
OLFR1274
OR2M1P
OR7E24
SNORD89
SPAG17
SSP-10
ZNF530
CD300LD
KIAA0895
MEIS2B
MIR1268A
NKX6-3
PSMD11B
RP1
SIX3B
TAS2R19
UGT1B1
ZFP410
ZNF425
ATG4DA
OLFR154
PAGE2
PDLIM5B
RNF5P1
RTN4A
SLC8A1A
TMEM196
ZKSCAN14
IGKV10-96
IGKV15-103
LINC00528
LINC01093
LYVE1A

RBM39A
THSD1P1
ACOT16
FAM69C
GCSAML
MIR1-1
MSRB1A
NLK2
OR1C1
OR2A4
RPL7AP36
SERPINB14
SLC16A12A
SUPT4B
ZBTB16B
CHKB-CPT1B
SLC35G6
TGIF2-RAB5IF
KRTAP24-1
NPBWR2
DCAF8L2
DUSP29
GBGT1L4
KRTAP12-1
KRTAP12-2
KRTAP4-12
KRTAP4-8
KRTAP9-8
MUC5.2
NLRP13
OBOX2
OLFR846
OR11A1
OR51S1
OR52E4
OR52L1
OR5T2
SNORD115-6
TAS2R1
BCO2L
CGB8
EBLN3P
PPP3R1A
PSG11
SNORD93
ZFP954
CDKL4
CYP2AA7
EMBP1
FKBP9P1
HSP70.2
SMIM10
ASCL4
HTR3D
HTR5B
LINC01505
MAGEB6
OR11L1

OR5H6
SLC25A5-AS1
SNORD103A
ANKRD40CL
AOX2P
FAM183A
H2-EB2
MIR3646
OR10G4
OR4D1
OR51A2
TAAR3
ZFP85
ERCC6L2
COG2
LGSN
LRRC10B
PABPC1L2A
RUNX1-IT1
ZNF436
UNC80
IL9R
RGD1563941
AVPR2
KLRA17
NCBP3
TASL
ZNF768
FOLT-1
OL-VIT1
RDH10B
TP73-AS1
GP5
PPP3R2
TMEM260
ZFP628
C2ORF42
PCDHA1
RIPPLY1
TIMM29
ZNF385C
ZNF830
BOD1L
C5ORF46
CD300E
HORMAD1
KCTD12B
SNX31
TUBB8
AND2
C1ORF56
C1QL2
ITPRID1
MBD3L2
RAX2
SYT16
TXNDC2
UBE2CBP

ZBED9
ARSL
PDK2A
PLP1B
PRSS30
WDR38
ZFP606
A2ML
CDRT1
LINC00673
RGD1311946
SPIN3
C6ORF58
CCDC70
DCAF16
ETV7
GTF2A1L
H3F3C
SFTA1P
C5ORF51
CBX7A
DMRT3
IQCC
MIR3085
PWAR5
RNF17
SOX9A
TMEM101
DCDC1
DHRS13L1
H3C6
IL19
PGLYRP3
TCEAL7
INIP
MIIP
RAG2
LARS1
MAPK1IP1L
WDR13
ZNF160
CRB1
MED8
MIR669N
PRR12
PHRF1
MBL
HSP7C
MC4R
ZNF112
HSC70-PS1
VED
OTOP3
SNORD43
SOWAHCB
FAM43B
KPNA7
MEX3A

MIR199A2
MIR219A-1
MIR3475
ZNF57
CHIL4
CST4
SYS1
TOP1L
CABP4
CYP2C23A
DUSP23
KRTAP19-4
MIR1915
PTGS2B
UBE2IA
CZIB
IGFN1.3
KLRA4
LINC00526
OLFR569
PCDHA10
TIMP2B
ZNF587
ZNF839
CFAP410
HMGB2A
OLR828
PLIN
RT1-CE16
SPATS1
AQP10
CAVIN4B
CRABP2A
GPR15
H2AC16
HMGA1A
INSYN2A
MIRLET7C-1
PFN3
RP1L1
TCRB-J
TUBA3D
XRRA1
ZFP763
AIFM4
PRR33
TMEM102
ZFP119B
BPIFB2
BPIFC
CYP4F17
ISX
KCNJ6
KRT76
LRRC9
MIR665
MRGBP
PTPN23A

SEPN1
ZFP819
ZNF282
ZNF486
CATSPER1
CDCA9
DCAF1
DDX53
EIF4E1B
GPR32
MAGEF1
MAP1LC3B2
SVILA
THAP10
ZNF304
ZNF853
APOL11B
CA10
D5ERTD605E
FAM186A
LRRIQ1
MIR1226
MMP1A
OPN6A
RDH8A
SFRP1B
SLC12A10.3
SLC35E2A
SMAD3B
SNORA71A
TDRD12
ZFP282
ZNF350
ZNF585A
BSNB
CARNS1
HDLBPA
MIR1983
OLFR206
ZNF674
ZNF844
COL28A1
D9WSU90E
HNF1A-AS1
LY6G5C
OXCT1B
AKAP1B
ELOCA
MIR466J
NPCD
PTBP1B
SLC6A1A
SNORA6
CHTF8
FUT8A
MIR712
OCLNA
OLFR1381

OLFR558
SMARCAD1A
SNORD4A
SNRPD3L
SPEF2
CDKN1BA
CES2F
DNMT3BA
ETL4
HCFC1B
HSP90B2P
IST1
MARCKSB
PLVAPB
RND1B
SHDB
ZNF296
BCL11BA
CD82B
FBXW15
IQCA1
MIR466G
MKNK2A
PRKCBA
SLC1A2B
SLC6A4A
ECRG4A
IFNA5
IFNE
IGKV19-93
MMP
NFASCA
OLR1517
PRODH1
PVALB5
ST18
GPM6BB
PFN2L
SNORD38B
TAS2R137
TERA
ARL8BB
C11ORF53
CASR
JARID2A
TEN1
TRP53I13
ATP5S
MSP-142
MSP-152
MSP-19
MSP-3
MSP-40
MSP-53
MSP-55
MSP-59
MSP-65
MSP-76

MSP-77
MSP-78
NFIXA
RGD1560925
SOBPA
AHSG1
CACNA1DA
CAPN3B
GST-30
OR2T4
PRELID1A
SDK1A
SDZ-8
SH2D3CA
TEX44
TNRC6C1
UCK2A
UGT-48
ZEB2OS
ZNF322P1
DNAJC3B
GNG12A
LRRD1
PSG23
SNORA60
SNORD116-2
VTNB
BAMBIA
BTF3P11
C17ORF64
FAM86JP
GRNA
HOXB5OS
IQCF3
LINC02875
POLR2J2
RGD1308134
ZFP383
ZNF343
C9ORF131
FKBP10B
KTN1-AS1
MIDEASB
MIR211
MIR518C
MIR663B
RGD1560883
TMEM213
TRGC2
VCX
ZNF66
COL1A1B
COL2A1A
DEFB104B
DHRS13A.2
F55G11.2
GLNS-PS1
JUNBA

PER1A
POTEC
SPEM2
CTSBA
EFCAB13
HSFY2
IRG1L
LINC02870
MIR672
MXRA8A
NUCB2B
OR10G2
PABPC1L2B
RBMY1A1
SNORD34
TCL1
TRIM6-TRIM34
ZNF35
INAFM2
KRTAP10-7
MYHC4
OR52B2
PDHA1A
SHISAL2A
SNORD3A
WDR95
ZNF271P
AADACL4
ANXA11A
BTNL3
C10ORF67
CSAG1
DEFB113
FRMD1
GOLGA2P5
IGFN1
KRTAP10-6
KRTAP10-8
KRTAP4-4
KRTAP7-1
LINC00671
LINC01121
OOSP2
OR10V1
OR11G2
TCL6
TFIP11
ZNF559
ZNF679
ANGPTL2B
C5ORF38
CNGA4
CYP4Z1
DESI1A
HIPK4
IGKV12-46
MMP23A
PTPN4A

RNF227
RPL13AP3
RRNAD1
THAP12
TTC6
ZFP235
ZNF513
ZNF658
ZNF792
C16ORF92
CARD18
CYP3A57
GPR119
IDI2-AS1
MBLAC1
PLPPR3
PRORSD1
SLC25A36A
SLC4A10
SNORA27
SNORD116-22
TOGARAM1
ZNF653
ANKRD65
C8ORF34
EVPLL
LINC01239
LY86-AS1
MIR589
PDHA2
RP9P
ZNF280A
ZNF484
C10ORF90
MZT2B
OGFR-AS1
PNMA3
SOD2-OT1
TYW1B
UCMA
ZFP87
ZNF280B
C1QTNF9
CCER2
CFAP61
CYP6G1
HPDB
LINC00475
STK31
USP27X
ZFP595
CYP6A2
ANKDD1A
RT1-M3-1
RASGEF1A
GPR21
HER3
KLRA15

KLRE1
TOMM70
AKAP14
HBA
KDM5BA
MIR582
PCDHA6
TUBB2C
WNT8B
KLK1B3
MIR199B
SAAL1
SAXO2
TRAC
KIAA1143
MIR190B
MIR295
RGD1561157
ZNF335
AHR1
FBXO24
GUSBP4
MIR879
SPART
ZFP157
ZNF583
KIAA0586
LRRTM2
MRGPRX2
PACRGL
SPATA19
TAS2R114
ZNF302
ACNAT1
CCDC160
GARRE1
GBAP1
WASH5P
ZFP3
ZNF708
ANXA2A
FAM217B
NRARPA
TMEM145
TMEM270
TMEM35A
ZNF184
KLRI2
MIR9-1HG
PADI6
SIX7
ZFP189
ZPBP2
C16ORF74
MKI67IP
TRIM30B
ASF1BA
BPIFA2F

CNGB3
NEDD4A
ACAD12
P22K15
ARPC5B
OLFR342
AMA-1
GUK1B
IFNA14
LDHBB
NEUROD6A
RLBP1A
SYT5B
C1RB
DCANP1
DEFB4
SNORD56
ZFP691
CXCR4B
DDX39AA
MICAL3B
MYCH
RGD1308065
STX11A
TMEM269
BPIFA3
CHIA.4
MIR1948
OLFR47
TNNI2B.1
TPI1A
ZFP867
OTULIN
PRODHA
RGD1309540
TAK1
ADIPOR1A
CHI3L4
GCM1
MIR706
NDRG3A
OLFR1215
C1ORF131
PBLD1
PCDHGB8
ZFP931
CCNP
CTSLA
KRT38
OLFR460
OR52H1
PDCD4A
RNU4-2
SPIN
TSSK5
CAPZA1B
CBWD
CDH15

LSMEM2
MYO15AA
OLFR738
SIR-2.1
AMY2A3
ARHGEF17
CCDC44
KNDC1
PTGES3B
SLC2A11
ZNF317
ZNF731P
CCDC105
CCDC13
CSPRS
IGHG2B
KRT24
LILRB2
MFSD14A
MGL2
OPRPN
SNORA3A
IGHG2A
MRPL42P5
NBR2
NPIPB7
PATE4
PPP2R4
RNASET2A
SNORD55
ALDH9A1B
ATP2A2B
BOLA2B
CASTOR3
CPSF7
ELOVL6L
GID8
GST-12
MID1IP1B
MSP-36
MSP-50
PLEKHG5A
RNF148
TMEM14B
USP2A
ZFP609
ZNF621
ZNF7
C6AST4
C7ORF50
CCR9A
DMRTC1
FOSL1A
IMPG1
OR4K2
PNPLA1
SURF4L
TLCD4B

ZNF738
ADAM6B
AK8
CXORF38
GTF2H2C
LCE4A
OR1A2
PAGE2B
SLC4A2A
SOX2-OT
TMEM170A
ZNF204P
ZNF441
ZSCAN5B
ARMC3
BANF2
C12ORF29
CFAP57
FANCD2OS
MAP3K15
MIR373
OLFR70
OTT
PHOSPHO2-KLHL23
PRR36
SLC9A4
TADA2B
TMEM233
TOMM20L
ZFP119A
C11ORF91
CAPN13
COPS9
EMC3-AS1
MGST3B
MIR138-1
NANSA
NR5A5
OR6A2
RN7SL1
SIGLECE
SLC38A5A
SNORA63
TSGA8
YWHAEP1
ZNF695
AIM1L
C11ORF42
C2ORF66
CDR-1
CGB2
CYP2AA4
DOD-24
GPR75-ASB3
HLA-DQB2
HRH4
IL26
KHDC1L

MIR600HG
OR1I1
POM121C
PRB3
SP9
ZFP791
ACBD5A
H2BP1
IFIT1BL1
MIR1307
MIR659
N4BP2L2-IT2
OR10J3
PCDHGA6
CUL1B
LCE6A
LINC00574
MROH5
MTERF1A
NPAP1
PSG2
RPL23AP82
SIGLEC16
SLC25A41
TEX19.2
TEX21
AGAP6
BRP16
EGFBP2
H2-T24
HSP70.1
IRF1-AS1
LCN4
MAGEB3
SLC25A47A
SNORA8
SNORD116-21
TMPRSS11BNL
TSPAN19
ZFP36L1B
DEFB25
DHRS4L2
HPS6
LINC01133
TSSK1B
ADORA2A-AS1
C10ORF71
CELA3A
FAM153CP
GIOT1
MIR616
ANG3
C9ORF43
CASP16P
CECR7
CLEC9A
MT1DP
OR2B2

SPATA16
C11ORF80
RGD1309362
SNHG10
GPR22
NLRP10
RTP1
SMDT1
ZFP69
FCAR
PDF
ATP5MJ
C17ORF80
C9ORF116
MIR197
TPI
ZDHHC24
ZNF542P
CRACR2A
NUPR1L
PYCRL
STAG3L4
C2CD4A
FCRL5
LURAP1
SNHG16
TGM6
ZNF664
AFDN-DT
CES5A
KIAA1191
SPRR2A2
ZNF805
ADGRG5
CFAP73
CPNE9
GJD2
IL31RA
ZBTB47
ZNF714
CD200R1L
DLGAP1-AS1
ZNF480
MAGEA10
HSP47
CLDNG
HBAA1
SCD4
SPEER4D
MYL6L
ZNFL2A
GRIN1B
PCDHB19
TIMP2A
EFNB2A
EGL-1
INKA1A
MYLIPA

UQCC
FGFBP2B
H2AC21
HMGB1-PS7
MIR448
PRP15
RS5-8S1
SNORA84
SP5L
SPON1B
TULP1A
CAR6
CYT1L
LPCAT2B
MIR8114
PISD-PS1
RPL3L
TRIM30A
DLGAP3
ELOVL7A
OPN1LW2
SLMAPB
CRYGM4
CYP2B23
KLRB1C
MIR466I
SKIA
CFAP298
CGB1
PMELB
RBM11
SLC27A2A
SNORA72
ANXA2P3
BABAM1
MED14OS
RARAA
ZFP347
ZP3B
ZRSR1
GPAT2
IL11RA2
MIR1180
BZW1B
CCDC61
CYP2AA9
HD
LEMD1
MIR486
MIR669C
OLFR915
PPP1R3CA
SNORA47
SVS3B
TENT5BA
ZFP518B
ZNF395B
BIN2B

C14ORF28
CES1A
CTSLL3
FAM3D
OLFR749
REX2
S100A10A
STMN4L
TCRD
TRGV9
UCN2
ZNF485
ZNF74
CCDC33
FABP1B.1
GRIK1A
MMP13A
RT1-M6-2
RXRGB
SLC26A9
SLC46A2
SNORD46
TMEM179
ZP2.2
AHR2B
C18ORF54
C2CD4C
CELP
HMT-1
HOXB5B
LMO4A
RRN3P3
SNAI1A
UNR
WDR87
ZNF605
ZSWIM2
CLDNE
CYP4T8
DNAH14
OLFR1316
RGPD8
SLC7A5P1
SNORD74
STATH
HCN1
LCN9
SERF1B
SNORA14B
TERB2
TTI2
EFNA1A
KRTAP19-5
SCINLB
TAS1R3
CYLC1
FAM105A
LCE1A

PYDC1
RGD1565222
ZEB2A
BXDC5
EIF3J2
GJE1
MXC
ZNF876P
C17ORF75
C1ORF167
CSRNP1A
GADD45AB
OR10J5
RAB11AL
SERPINB1L3
SYNGR2A
YWHAG2
CNTNAP3
CSTL1
DDX26B
DHRS13A.1
GOLGA6A
GST-7
HR96
MIR322
RPL37RT
HEATR2
KLRA21
MIR155HG
POTEB
ZFP959
ANKRD60
CCDC27
LGALS14
LINC00477
OR2B11
TDRD10
CAM
D6WSU163E
GPR151
SLC35D1B
ANKRD19P
AQP12A
C20ORF27
FGF20
GGT8P
MORC2A
RGL4
TMEM170
ANKRD66
CATSPERE
CCDC162P
CYP1D1
HECTD2-AS1
KDM4D
KIAA0430
KLRC3
LUCAT1

MIR380
MS4A8
SID4
TDPOZ2
TMEM51-AS1
ZNF793
APCDD1L-DT
DPPA2
PAPD5
PCDHGB1
PDXDC2P-NPIPB14P
ZNF222
C1ORF94
TDRD6
HTR4
POSTNA
WFDC21P
ZNF141
C11ORF49
FAM72D
GFRAL
KRT39
PSORS1C3
VMN2R29
ZFP790
ZNF572
ZNF670
DGKK
FBXW10
MEIOC
SMIM15
SMIM5
FCGR2C
ACIN1B
FBLL1
RNASE2B
FBXL22
SNORA55
ZNF680
SNHG28
CRABP1A
CRYGM5
ABCD3A
MIR33
STAG2A
AVD
CDH9
LONRF1L
LOXL3B
MUP12
PRSS34
GNGT2A
H1F8
NFKBIAA
OR4D6
VEGP2
ENTPD2A.1
RAB32A

SLC16A9A
SMIM38
ZNF668
AKAP12B
ATF4B
NRXN3A
NXPE5
ZP1
ACIN1A
ACLYA
CYP-35B1
FGF22
METTL4
PJVK
PRSS54
C3ORF14
CASP3A
DENND6A
PYK
RNASE12
SEC22BA
TARM1
CRYGN2
KIAA1586
MIR215
MIR298
PRL2C4
SEPT5B
SNORD21
TEKT4
TLR10
DENND10P1
MILR1
MIR743A
NT5C2L1
TPGS2
ZFP764
COLCA2
DEFB42
HDR
ZFP46
ZNF138
ZNF235
ADAM26A
APPA
CLCA4A
DCDC2C
HOTAIRM1
PLEKHS1
POT1A
SNORD3C
TMIGD2
TOB1A
CFHR3
G6PC1A.1
MIR30C1
MT
RAB39B

RIMKLB
RT1-CE12
SNORD35A
PARA
KRT40
KRTAP21-1
OLFR780
ACTA1A
ANKRD20A8P
CHKB-DT
GIMAP2
LY6G6F
MAGEA2
METTL2B
MIR942
OLFR46
PMS2P9
THAP7-AS1
ULBP3
ADKA
ARHGEF9A
CAMKK1B
CAX2
CYP2K20
DBNLB
GST-38
HIPK3A
MHC1LAA
MIR207
NOTUM1B
OR128-3
OR2F1
RHOL
RN18S
RTN1A
SLC1A9
SLC2A9L2
SNORD13
SRD5A2A
TRIM25L
TSPAN34
ZFP27
ZFP788
ZNF81
GLYATL3
RAB2
CEACAM8
CETN1
DAO.2
FAM66C
IFRG15
NIPSNAP
OOEP
ZFP36L1A
ZFP60
ZGLP1
ZNF584
ZNRF4

ANXA5B
CHASERR
FOXI3
KRT82
MAGEB18
ODAPH
PCARE
UFD1L
CD74B
DNAJA3A
DPY19L4
PGM5P2
PYURF
STPG2
C19ORF48
C1ORF158
CST9L
KIRREL
MIR1260B
NCR2
PAX6A
SNORA24
COX4
CTAGE4
FGF7P6
HIST1H4B
LASS6
LINC00938
LIPI
MAGEA11
MIR511
MROH2B
PRSS30P
RBMXL2
ZFP956
ADH8A
ANDPRO
BPIFB5
C17ORF99
C22ORF34
FAM170B
KRTAP4-1
MUC21
PBX1A
PDC
PMP22B
SLFN12L
SUPT20H
TPRXL
CASPB
CYP27C1
LRRC46
MISP3
SNORA10
SNORA31
ZNF774
NUPR2
OLFR458

OSTCP1
ZC2HC1B
ARL16
HHLA2
STAG3L3
SYCE1L
WBSCR17
ZFP445
ZNF433
ZNF528
ZNF808
ZNF829
ADGRE3
LINC00470
NKAPL
RGD1311739
ZNF385A
HOXA-AS2
KCNA6
LINC00844
PLGLB2
MIR1894
SLC2A3P1
AGBL5
HIST1H2BG
IL17RA1A
C11ORF68
IQCH
MIR504
GNB3A
HSP-90
MLL2
OPRD1
PRL8A4
RCVRNA
ATP1A3B
MIR137
OLFR1394
ZNF791
EFCAB5
PROSER1
PTGES3L
SIGLEC9
TAS2R118
ZFP740
ZNF92
ATXN1L
PIFO
ZNF770
ATP10B
RGD1307100
RGD1561149
ZFP385D
ZFP640
CTPS1A
HSP90AA2P
RGD1562378
ZNF492

MIR743B
PABPC4L
PCDHB6
PLP1A
STM
C1ORF35
SNORD50B
FIRRE
GHRB
NMUR1
PCDHB18
CES2H
DPY19L1P1
FENDRR
GAD1B
GMNC
PCDHGC4
SPATA48
AND1
DHRS3B
HNRNPA1L2
ITIH3A.1
LINC00623
GPR89B
GUCY1B3
NPIPB15
PRL2B1
RLBP1B
RT1-DOA
SLC2A4RG-PS
CD209A
GNB3
ID2B
KLRC4
OR2A9P
PP2D1
RGD1565367
RT1-N2
SOGA2
TPD52L3
ZFP296
ASIC4B
C1QL3A
C1QL3B
CCDC53
CHST3A
CMASA
CRFB15
GBP2B
GRK7A
HARS1
HECW2A
IKBE
K6PP
KCTD9A
LPAR2B
LRRC30A
LRRC8DB

MC5RA
MFSD6B
MYBPC2A
NCANA
OR2W3
PCYT1AB
PCYT1BA
PIP5K1BB
RAP1GAP2A
RNF150B
SCG2B
SELENOV
SERPINE3
SHISA9A
STOML3A
UBE2NL
UGT2A4
ZIM2
EIF3CL
FAM177A
GZMD
NOTO
RGS22
ZFP663
C2ORF92
KRTAP3-2
LGALS9C
LINC01587
LRRC14
MTAG2
PROKR2
SULT1ST3
TEKT4P2
XLR4A
ZNF22-AS1
ACCN3
HS90B
LINC00641
NKX2.5
RN45S
TUBAL3
C20ORF203
CXCR4A
DLX3B
GALNT8
H1F6
LILRA6
OR1F1
RXFP1
ZNF180
ZNF213
ANG4
CCER1
WFDC8
ZFP112
ZNF268
ZNF682
ANKRD33

CFAP91
FRMD7
KRTAP6-2
LINC00479
LINC01116
SAPCD1
TEX29
CYP-35A2
DDX19
KDM6BB
LCN6
MIR3091
MLN
SH2D1B1
TBC1D3F
ANKRD63
C2ORF49
CERS3
ERV3-2
TRPV3
ZNF558
ATOH1
BORCS8-MEF2B
LINC00941
REG1
SERPINF2B
SPINT3
TEKT2
CSNK1A1L
GOLGA2P10
MIER2
TRHDE-AS1
AKTS1
ARRDC5
CD200R2
CMLC1
RGPD1
TTC35
ZNF620
ANXA1C
BMPR1AB
MIR186
CMTM2A
MEA
NPM1A
VOM1R92
VSTM5
CFHR4
KA11
MCO3
PRAMEF13
SLC7A3A
HOXC13A
RORAB
SST2
GSTE8
OLFR1402
RNU2-10

ZMIZ1OS1
CRYBB1L1
CSH2
EIF3S10
STMN1A
UQCRHL
VDAC1P3
ERICH4
GHRA
MESPAB
BSK
MIR622
NCR3LG1
OSER1-DT
PMS2P3
RGL3A
ACTL9
CFAP300
CROP
HSP90AB2P
MIR598
PPP1R9BB
SNAP25B
STAT1B
CDH26
LGALS1L1
NPFFL
PRDX6B
SLC43A2A
ZFP870
APOA4B.2
ARSK
EIF4A1B
GNG13
IVNS1ABPB
KLRA3
WDR27
APS
BPIFB4
GAREML
H2BC24
FAM27E3
OLFR110
TMEM266
CREG
HOXA3A
IGF2A
LINC00476
XLR3A
H2-Q5
MIR-375
REC114
SSRP1B
TMEM244
ZNF1065
CKMT2B
CSPG5B
CYP2AA11

CYP2AE1
FLOT2B
KLRB1F
MCUR1
MS4A17A.8
NAT8F4
P
PCGF5A
PPM1LB
PRTGA
RALAA
SDHDA
SIX4A
SOUL5L
SRP54A
TCRA-V54
TMEM237B
ANKRD20A11P
ARSH
CLDNB
FAAH2
FAM176B
HES3
MIR669A-1
MIR669A-10
MIR669A-4
MIR669A-5
MIR669A-6
MIR669A-7
MIR669A-8
MIR669A-9
PIMR213
SPRR2A1
ZNF12
ZNF281B
ZNF782
ATP11AUN
CEBPA-DT
H2AP
LRRC38
MIR125B1
GOLGA6L9
GRXCR1
HUS1B
MIR302D
MIR6997
MIR8118
MSS51
OLFR156
SEPTIN12
AKAP2
CDR-4
D3ERTD108E
DNAJC5G
LINC00460
LINC00965
PYP
ZNF696

ANKRD34C
ARSE
KHDC1
PPYR1
SHOC1
TAAR1
VOM1R67
VOM2R11
VOM2R8
ZNF181
ZNF41
CAMK2N1A
CUX2B
CYP2D5
CYR61
ENTHD1
GSTT4
PARP12A
ATP8B5P
CD209
DELE1
DVR1
JPT1B
KCNU1
LELP1
MRGPRX1
THEX1
ZNF182
ARL14
CBX8B
CDC42EP4B
CHD4B
CYP1A4
GPA33
KRT37
MIR29B2
PCDH18B
RAD21A
RGD1309104
RRP7BP
SLFN5OS
ST7-AS1
C1ORF74
CABP7B
KLK1C9
OLR776
RBMS1A
SLC37A4A
SLURP2
TAGAP1
TRHDE.1
VSTM1
ANKRD31
ATAD3C
C6ORF223
CD3EAP
FLVCR1-DT
GAREM

LINC01119
MIR4524A
NUTM2A-AS1
RRN3P1
TMEM190
CATSPERB
OPN1LW
PPNR
PPP5D1
TMSB4XP6
ZBED3-AS1
GPR179
OLFR60
ARHGAP32A
CLDN23.1
CYP3A26
DNASE1L4.1
IER2A
KIR2DL4
MPPED2A
PHC2B
RBMS2A
RGS12B
UBL3B
FAM71D
HOXC12
MRGPRB11-PS
TNFRSF22
UGT1AB
ZFP446
ZNF566
ABCA3B
GFRA1A
NEXMIFA
SCN8AB
TDG.2
TRIM34B
OLR1635
OLR81
PAK7
SPART-AS1
DNMT3AA
TLR11
ZRSR2P1
BPIFA5
HSP25-PS1
LRRC4
RGD1559600
SERPINA3I
ZBTB6
ZNF627
CHK1
EXOC3L4
LCE3B
LRRC55
MIR1896
OLR124
OLR1250

OLR215
OLR382
PCDHA13
RGD1559804
ACC
ALDOAB
DCPP1
ELDR
IAP
LYC2
OLFR52
TSPAN36
VTG6
ABCC6B.1
BCL11AB
C14ORF119
C19ORF25
FAM156A
PIMR168
SMEK1
SNORA3B
TMEM88A
CATSPER3
DEFB44
H4C7
SKOR1
SQT-1
ZNF20
CYP4F41-PS
FABP3P2
GPR82
MIR520H
NNR
ARCH
C80502
CCDC136B
CCL21D
CYP11B
CYP2AA8
EPB41L3A
ERAS
ISP2
MAGED4
METTL7A2
PHYHIPLB
PPS
RGA
SERPINA3L-PS
SPATA45
THBS4A
GIMAP1-GIMAP5
LRRC37A2
PCDH2G28
RGPD6
TBX2B
TRAPPC2B
WHSC2
ATAD5A

C9ORF135
CRISPLD1A
EFNA2A
FAM87B
HE1.2
INAA
LSM12B
PHF20A
PPM1NA
PRP19
PTGER2A
RCAN1A
RGD1310495
RTL1
RTN4RL2A
SLC1A8B
SLPR
TSPAN13B
UGT5A1
ZFAND5B
ZFP105
CEP83OS
COL-173
EEF1A1-PS1
NPN2
RAB7-PS1
SSP-11
TTLL8
UMPK-PS
ANKRD50L
CALM4
CDHR3
SMIM27
SSX6P
ZFP65
ZNF322
ZNF841
SAMD15
AAT1
ASH1L-AS1
B3GALT5-AS1
CPL2
CWC15
ENO1-PS1
GIPR
GSTR
HMX1
MUP-PS4
PSMB11
TNPO3
ZNF615
ANO10A
CDX1A
EOLA1
FGT-1
HEXDC
KCNN1A
LINC01089

MIR384
MYLK4
OAFA
OR6J1
PPFIBP1A
SET-17
TPT1-AS1
TSGA10IP
YWHAB.L
CKBB
IL17REL
OLFR1080
RSPH3A
CDC34B
HOXB-AS3
OR10Z1
TTLL2
C12ORF77
CTXN2
FOLR3
HELT
IGLV1-44
MAP1
NOTCH
OXCT2
SNORD115-15
SSX9P
TEX26
ZFP26
ZNF474
ZSCAN29
AGLA
AWAT1
OLFR201
OR2T2
SLC38A3B
TSPAN33A
WBP11P1
ZFP711
AQP12
C3ORF20
MUC3A
OLFR315
OLFR836
PRCD
PSG6
SERPINB9C
SYCP2L
ZNF487
AJM1
AMPD2B
APBA1A
DYNC1I2A
ECM1B
GLS2A
GNAL2
ITGAE.1
KIF16BA

KIF16BB
LMF2A
MALRD1
MPDU1B
MS4A4B
NBR1B
RND1A
RNF126P1
RNR1
SERINC2L
SLC22A7B.2
TAS2R16
TRIOB
DEUP1
MIR657
MIR888
UGT2B6
Y59E9AR.1
ZNF630
ERICH6B
FBXW26
GKN3
MIR4688
SNORA2C
TTC18
CCDC8
D1ERTD692
GABRQ
IL22RA2
MAP10
METTL2A
SKN-1
UBA1Y
ACOT11B
C14ORF177
C3A.4
CCDC106A
CST13P
CYP27A1.4
CYP2X12
DYNC1I2B
FCOR
MIR1256
MIR1283-1
MIR142HG
MIR2052
MIR320B1
MIR351-2
MIR519A1
MYO7AA
NSPA-8
OLFR1382
OLFR687
OR2A12
OR6F1
OTX2OS1
RCA2.2
SNORA71E

SNORD109B
STC2B
UGT5B5
URAHA
ARHGEF33
CT47B1
DEFB125
FAM138D
KRTAP12-4
KRTAP5-7
LBHD1
MIR7-3
OR10H5
OR2T6
OR4K17
OR5AU1
PPY2P
PRB2
SPATA31D1
SYS1-DBNDD2
TGM2B
EPHA2A
GYPE
MIRLET7C2
OBP2B
SULT1ST2
ZSWIM9
C3-3
COX4I1L
DAO.3
FOXO
OLR1584
TCEB2
CD1C
EPHA10
GPIA
MIR378G
MUP-PS10
MUP-PS18
OLFR978
RPS12L2
C12ORF63
CDY2B
CYP2A7P1
CYP2K6
LINC00598
LINC01720
LINC01743
SNORD29
CYP4V8
LINC01128
PRMT9
ZFP91-CNTF
DEPDC4
OR2M7
PRKCDBP
ZFP385C
ABCA11P

ZFY2
ADMA
ATP1A1A.4
CAMK1GA
CLEC4GP1
CTSBB
DAAM1B
HMSD
MEP1A.1
MIR526B
NFE2L1B
PRSS37
RPL23AP32
SPRR2J-PS
STAG3L2
ZNF389
ZSCAN32
CD177
OXSR1A
PTRF
C2ORF27A
CATSPERG1
MIR3081
NBPF14
SPATA32
ZFP287
ALOX12P2
BRD2B
IGF2BP2B
LINC00320
LINC01366
LINC01831
MIR1266
MIR548I2
OLFR373
PAQR9-AS1
RPL32P3
SLC7A12
SNORD114-4
SNORD114-8
ZFP36L3
ZNF735
MUC12
NDNL2
ZNF461
DCHS2
LRRC43
MAGEE2
MYO1H
PRKCQ-AS1
TEX19
ZNF404
ALDH1L1-AS2
AOX3P-AOX2P
ATP2B2-IT2
BPY2B
C17ORF77
C1ORF220

C3ORF49
CASC8
CCDC144A
CFC1B
CLPSL1
CRYM-AS1
DCAF13P3
DCDC2B
DEFB133
DIRC3
EIF3IP1
ESX1
FGF
FIGNL2
FOXN3-AS2
FRG2DP
FYTTD1P1
HPVC1
IQCA
KARS1P2
KCNIP4-IT1
LBX1-AS1
LINC00303
LINC00466
LINC00519
LINC00628
LINC00643
LINC00971
LINC01020
LINC01189
LINC01194
LINC01615
LINC02018
LINC02044
LINC02267
LINC02506
LINC02520
LINC02691
LINC02694
LINCR-0002
LIPS-7
LRIG2-DT
MFF-DT
MIR153-2
MIR5103
MIR548F3
MIR7-1
OR2W5P
OR52A4P
OR52E8
OR5K4
PAGE3
PCNAP1
PER3P1
PPEF1-AS1
PSG10P
RNASET2B
SEPTIN14

SKINT1L
SLC26A4-AS1
SNORD115-21
SNORD116-18
SNORD116-3
SNORD116-9
SRGAP2D
SYCE3
TGIF2LY
TIGIT
TTTY8B
APOL4
GSTD1
KCNMB3
MS4A14
SMR3B
VMN1R54
ZNF646
GNGT2B
TOB1B
WIBG
ABHD16B
C11ORF21
HLA-DRB6
MAP3K19
MRFAP1L1
NTF5
SCGB2A1
ZMIZ1-AS1
L1MD-TF26
OLFR1436
OLFR748
RT1-N3
CFAP47
PCDH11Y
ZUFSP
ASIP
ZNF554
BTNL10
CLEC3A
IQCF1
NEK10
RT1-A2
SERPINB9B
SLC16A12B
TRAPPC13
ZNF576
DNAJB7
GUSBP14
KIFBP
ADAM10A
EAR6
LEXM
LY6I
SKINT3
IGSF23
LRFN4B
RT1-T24-4

TKTB
ZNF18
CYP-29A2
FBXO15
NMS
NOL4
FUT8-AS1
MIR1955
PCDH11X
RAD51-AS1
TAS2R119
CRY5
GUCA1D
KIR2DL2
KLHL38B
PHKG1B
S6K
CAR9
OR51E2
PDXDC2P
TCRG
XAGE2
CALHM3
OXER1
PDZD9
PVALB2
ZFP780B
FAM71A
TSSK4
OLR1135
PRICKLE4
SELK
ZNF398
MIR495
NLGN4Y
PCDHGB7
WASHC2A
CFAP100
CYP12D1-D
CYP12D1-P
LINC01003
RFPL2
C11ORF95
FAM153A
ZNF106
GNRHR2
IGKV1OR2-118
KRTAP4-13
MIR18
OLR104
OLR1147
OLR1325
OLR1439
OLR436
OLR610
PLEKHG4B
MEL
MS4A15

PCDHB3
SLC35D1A
SYNDIG1L
ZNF394
KRBOX4
MFSD4B1
NBDY
P2RY8
SYT7A
UGT1A
CAMKVA
CSPG5A
FCRL2
MIR340-1
NTNG2B
OLFM3A
PCDH1GC5
RGS5A
SLC22A31
TAFA1A
WASHC1
ALCAMA
MIR1825
ZFP369
KLRB1
MIR6215
SPINK8
ZFP418
DUSP5P1
C4ORF45
DEFB21
TBRG3
TIGD4
ZFP13
ZFP868
ARID3C
F7I
HBB-BS
HNRNPA1B
IGFBP5B
C1ORF109
OR130-1
POU5F1B
RAB39BB
TAX1BP1A
TMEM41AA
ZNF134
ACTL7A
CACNA1F
CREBBPA
FMR1NB
HSP-6
IGHV1-56
MAP1AA
NHLH2
RTN1B
SRSF2A
TSGA13

ZDHHC15B
GSTM.3
LINC00173
LINC02210
PEDS1
PHKG1A
PILRB2
TIGD1
TMEFF1A
UQCC3
CRYGA
GUSBP2
HAGLR
MIR6948
MIR883A
PCDH2AA15
RIMS2A
C5ORF22
CBWD5
DEFB10
FREM2B
NXF7
PRL8A2
RPSAP58
ATAD5B
CCDC56
FMO9
KCNE5
MIR145A
NR0B2A
SFRP1A
SSU2
VOM1R46
EBF3A
FTR51
MIR548V
OLFR1156
PDZRN3A
PIMR188
SNORD103C
SNORD14C
SP8A
TMPRSS11F
TMTC2B
ANK1B
ANKRD45
ARID1AB
CSTDC4
ESYT2A
FAM200A
FAM40A
GNAO1A
HNRNPL2
MCF2LA
MIRG
PCDH1G22
PONZR3
RAB11BA

SENP3A
FAM166A
NIBAN3
RCVRN2
SLC22A25
SSBP3-AS1
UTP14C
ZNF420
ADAM34
ATP5MEA
CDCA7B
COLCA1
LY49S6
MIR302C
TCP10C
TUSC2B
ZNF207B
CCL27A
CYP2C24
CYP-35C1
SGCZ
SNORD114-23
SNORD114-26
SNORD37
ARFIP2A
CPT1
FTLP3
GIGYF1B
GPR137B-PS
H1M
MIR33B
OLR658
PENK-RS
SVS4
T2
TBH
UGT1B7
ZFP473
ADMP
ALOX5B.3
FBN3
LINC01278
PTRHD1
TEX22
ZNF510
ANKRD28B
APLNRA
ARHGAP27L
CXCR3.1
DIP2CB
ELOAL
FAM227B
FAM90A1
KRT96
LINC00337
MORC3A
NPTNA
PRICKLE1B

SEMA4BB
SLC16A1B
SNX1A
THRAP3B
TP53I11B
TUSC2A
VGLL4L
ZNF106B
CACNG2B
FAM117BB
FXYD6L
GUCY2G
KCNMA1A
MFSD4B5
RPS4L
SMTNB
SYNGR1A
ZFP947
AP2M1B
ERVK-6
GABPB1L
ITM2CA
KLK14
OLR1579
VMN1R212
VOM2R16
DUX4L1
MIR525
OR4C15
SAMD1
TRIM12C
ZNF26
EIF4A1A
ERICH2
QRFPR
RGD1561636
ZNF346
CBY2
KIAA1430
PRLHR
ABCC6B.2
ADAMTSL4-AS1
GP96
HYMAI
MAP2K3OS
MIR3094
MIR682
NME2P1
OLR1453
OLR292
OLR515
OLR633
OLR77
OLR956
PARTICL
PRLRA
PTPRJA
SULT2ST1

ZNF175
ZNF6
ATP5IF1A
BICC1A
FGF8A
HIBADHA
HOXAAS3
IGKV3-12
KRTAP31-2
NLRP5-PS
PFKFB2B
PRAMEF22
RPS6KA3A
SLC12A7B
SNHG14
SPON2B
STAMBPA
VENT
WASA
WBSCR25
CNK
DLEU1
LGALS9L1
RGD1560523
SAGB
SLC35F6
VMN1R192
XAGE1A
C19ORF54
CCDC169
COL-138
DPY-2
EGR2A
KCNN1B
LILRC2
OLFR1412
PAG
ROL-6
SCGB1B27
ZFP109
ZNF419
ZNF586
ARHGEF7B
CYP2D34
LYZL6
MCART1
MRGPRX3
OGDHA
OLMALINC
PRB1
SLC8A2B
SULT2A5
TRIM52
ZDHHC20B
ZNF525
CAGE1
IGHV1-64
KRTAP4-16

NKRFL
OLFR613
OR13F1
OR14A16
OR52B4
PATE1
PRORSD1P
RGD1307929
SPATA4
CHST12A
DAB1B
DNM1B
OLR131
OPN1LW1
PACSIN1A
PCDH2AB2
SPEER3
TMEM136
ZFP469
ZNF257
ADGRE4P
BFSP2-AS1
CLEC-74
KCNJ12B
KDM2AB
MYO1CB
NDRG3B
OXCT2A
PRKACAA
RFX1A
ZFHX2OS
ZNF285
ZNF814
C1ORF122
COL-129
COL-62
COL-63
CYP-13A12
F55H12.2
HNRPKL
MSP-32
OLFR73
T25B9.4
VSIG8A
ZNF816
ZP3.2
BAI3
C8ORF48
GTPBP1L
HBAA2
HSP90AB5P
LBFABP
MIR551B
NXPE1
PFKPB
PTENA
RGD1309808
RMST

ZNF570
ZNF862
BAT1
GAS1RR
GIT2A
MIR376A
RDH16F2
SCAMP2L
TEPSIN
TMEM151B
VTG4
BEST3
CHMP2BB
MIR103A1
MIR631
NLGN4X
OLFR1155
PCDHA8
VMN1R205
ZCWPW2
ZNF540
ANO5B
DNAJC11A
DNM1P35
FRRS1B
LPAR6A
MEF2CB
NPFFR1L3
OOG1
PHLDB2B
PLEKHA7A
RGS3B
SPR-5
ST6GAL2A
BEX5
OLFR1044
OLFR284
OLFR347
OLFR574
OLFR736
SETA
SPRING1
ATP5MD
C20ORF85
GID4
IMPDH1A
OLFR323
PSG18
TCP1-PS1
CDRT15
IRX5A
MTL-2
OR2T35
OR4C3
OR56A1
OR5C1
OR5M10
OR5M11

OR7E5P
PLCD4B
PRSS45P
PSG7
RGPD4
SNORA78
EOLA2
HSP-70
IGHV1-62-1
MIR711
MUP14
OLFR1093
PSG27
RGD1561517
SNORA44
KLK1B21
KLKP1
OLFR1440
OLFR172
OLFR822
PTPRFA
RGD1562690
RITA1
RPL10L
ZNF579
ABCD3B
ANOS1A
ATP6AP1B
BRPF3
CAMSAP2A
CDC42EP1A
CNSTB
CYLDB
DNASE1L4.2
EFCAB12
EFNA1B
FAM26F
FLOT1A
FYNB
GRK5L
LDHAL6B
LINC01164
MIR133A1HG
MOV10A
MSRB1B
MYO7AB
NPR1B
OAZ1A
OR51B5
OR7G3
PLCD3B
PRKG1B
PRNPRS3
RAD23AB
RAF1B
RBM24A
SCARNA21
SH3GLB2A

SLC5A6A
ST3GAL3A
SYNE2B
TBC1D12A
TBCELB
TYMS-PS
VCAM1B
WBSCR27
C04G2.3
C17H12.8
E03H4.8
F09C8.1
FABP4A
GZMG
IRG-1
KLRA10
MIR507
MIR518B
MIR519B
OLFR958
SLC35G5
STOML3
TFAMP1
TTBK-2
W03D8.9
W03F9.4
WFDC5
HIST1H4L
ITGB1B
KLK1B11
MIR4530
MLLT4
NPHP3-ACAD11
NTN5
PIRA4
RGD1307595
RREB1B
SEPTIN1
SRP14-AS1
ACKR4B
ATAD1B
C32H11.3
CBLN9
CHI3L3
CLEC-143
COX5B2
CYP2AD6
CYP2X8
FNDC3BA
GBE1B
GPD1C
IL15L
KRT18A.2
LEG1.1
MLLT1B
MTX1B
NSPA-5
OATX

OAZ2A
PFKLB
PPP2R1BB
PRKACAB
SEC14L7
SEC61A1L
15-Sep
SEPT4B
SLC6A22.1
VMN1R27
XPO1A
ATP6V0CB
BSPH1
C2ORF78
C5ORF52
DDI1
EIF4A2P4
EVPLB
FOXC1B
FZD9B
HNRNPA1P7
KMT5AB
MIR1205
MIR9-3
MVL
NOG1
OLFR1008
OLFR1349
RT1-M4
VENTX
VENTXP7
VMN2R4
XLR3C
C15ORF32
C17ORF102
CDY1
CXADRP1
DEFB136
DMBT1L1
DOC2G
ECEL1P2
ERVK3-1
H3-5
ITIH6
KRTAP10-1
KRTAP10-12
KRTAP13-3
KRTAP5-10
KRTAP5-11
MAS1L
MIR378H
MIR6085
OR4F15
OR51A7
OR52I2
OR52N1
OR6C4
OR6N1

SELENOT
SNORA17B
SNORD9
TAAR8
TARS3
TBC1D28
ULK4P3
ZFP366
ZNF649
ANKFN1
C12ORF60
EFL1P1
FZD3A
GTF2H2C_2
HMG1
ODF3L2
OLFR160
OLR744
RBPMS-AS1
ZFAT-AS1
ZRANB2-AS2
CNBD2
GRAMD2
KCTD12.1
MIR541
NK4
ZFP426
ZNF460
C14ORF178
C8ORF87
CD300LD3
DYNLL2A
FAM177A2
GOLGA6L1
HIST1H3E
HIST1H4A
MIR1587
MIR4507
MIR709
OLFR242
OLFR54
OLFR775
RGD1562080
VLDLR-AS1
XKR3
ZFP850
CYP2B31
EIF3LP3
MIR654
OLR1093
OLR1200
OLR1416
OLR1451
OLR1668
OLR241
OLR295
OLR424
OLR653

OLR763
RPL5P29
RPSAP28
RPSAP3
RPSAP31
SNHG9
TMEM212
ZNF676
BUC
C12ORF45
CITED4B
EWSR1A
GSTE2
LINC00663
LMNL3
NCF1B
OPN1MW
TBX16
DEFA23
DEFB127
EXO70
KIF13BA
LY49I2
NCANB
OR5D14
SSXB1
WDR20B
ARHGEF3L
BTBD6A
CD248A
CDK5R2A
COL7A1L
CRYGM2D20
CYP2C1
DLB
KHDC1A
KIR2DS2
LY6F
MIR518E
MIR519C
MTMR1B
NAIF1
PLPPR3B
SV2BB
TKTA
CCL4L2
MAP2K4P1
METTL21EP
PTENP1
SLC43A2B
SNORA74B
SNORD116-5
SNORD116-7
SORD2P
STOX2A
UXT-AS1
ZNF500
ZNF736

ANKRD34B
CEP-1
CTPS1B
FAM227A
KLRA5
MIR523
SETD9
TMC1
TSNAXIP1
APOA-IV3
LINC01000
MIRLET7F2
SKINT9
VMN1R46
ZFY1
ARSF
C3ORF85
CYB561A3B
CYSLTR1
EMILIN1B
FLG-AS1
FMO6P
GSTA7P
LIMK1A
LINC01474
LINC01787
LRP2A
MIR1197
MIR124-1
MIR3150BHG
MIR546
OR10W1
PONZR5
PRR23A
PTGS2A
SEMA3BL
SNORA53
SNORD116-25
TCERG1A
TEX46
DIPA
LCN1
MIR376B
PCDHB12
ST1A8
ARMC1L
CBX3A
GRB2B
OLR386
SNORD49B
SRSF6A
ZFP592
ZNF784
ACNATP
ALG1L
ANKRD26P3
ANXA11B
AQP7P4

BADA
BBOX1-AS1
C1ORF141
C5ORF60
C8ORF37-AS1
CD81-AS1
CLLU1-AS1
DEFB106B
DEFB116
DEFB121
DEFB122
DEFB126
DNMBP-AS1
DRD5P2
FAM167A-AS1
FAM66B
FAM71C
GLDCP1
GNAV1
HBM
HCG9
IRAG1-AS1
KIAA1210
KRTAP20-1
LEF1-AS1
LINC00029
LINC00244
LINC00301
LINC00314
LINC00620
LINC01098
LINC01122
LINC01563
LINC02026
LINC02443
LMNB1-DT
MACF1A
MAPT-AS1
MIR1185-2
MIR3078
MIR4326
MIR4475
MIR9-2
MUC17
OR10Q1
OR11H12
OR14C36
OR1S2
OR2J3
OR2L2
OR4K5
OR52N5
OR56A5
OR5B17
OR5D13
OR6C1
OR6C6
OR6C75

OR8D1
OR8G2P
PSKH2
RABGAP1L-DT
RBM15-AS1
RNASE8
RNF216-IT1
SAA3P
SDHAP3
SH3GL1B
SIGLEC11
SILC1
SNORD114-20
SNORD114-21
SNORD115-10
SNORD115-35
SNORD115-38
SNORD115-8
SNORD115-9
SOX1-OT
SVIL2P
TAS2R8
TBL1XR1A
TESC-AS1
TTTY20
TUBBP6
WFDC6
XAGE5
ZDHHC8P1
ZNF223
ZNF236-DT
ZNF727
ARL4AA
CHI3L2
KRTDAP
TLCD3BA
RAD23AA
VN1R3
DEAR
GABRR3
MIR26A-2
MIR344B
MIR3569
MIR7224
NOMO2
ZNF581
1-SF
AKR1B10P1
FIZ1
HCG4B
L1MD-TF30
MAGI1B
MRGPRA6
OLFR1182
OLFR1198
OLFR1424
OLFR480
OLFR873

OR10R2
PABPC2
TESPA1
TUSC4
ZNF705G
F3A
FAM166C
KAZNB
KCNJ3B
MAGIXA
MYADML2
NR2F1-AS1
OLFR959
RBFOX3A
SERT1
SMIM26
TMEM181A
VOM1R2
ZNF675
LMNTD1
MIR3916
NOS
NYAP1
OLFR1202
RL
SMIM11
ZNF548
BSND
GOLGA7B
KIF20BB
MON1BB
NXNL1
PCDH1B
PLEKHA1A
PTK6A
SLC25A38B
ZIC2B
BORCS6
CHMP4A
STEAP1B
TMEM255B
ZFP747
AOX4
CEACAM16
KCNC3B
MIR194
MIR466H
MIR669F
MIR714
PARP12B
RAB3AA
TMX2B
TTN.1
VOM1R38
H3-2
GPR156
IGFBP2A
LFT1

MIR468
MIR871
NKX2-3
TAS2R14
TBC1D21
TDRD1
APLNRB
CCDC104
CDKN1CA
CRABP2B
GRAMD4B
NAP1L4B
NEURL1AB
OAS1E
OLFR851
RABGAP1L2
KIFAP3A
OSTN
RSPH4A
2-Sep
BUB1BA
ITPR1B
OLFR1121
OLFR661
PLECB
PPP2R5CB
PVRIG
SGIP1A
SH3PXD2AA
TAS2R135
TNA
VMN1R227
YY1AP1
ABCA4A
APOBEC2A
IGF2-AS
MPP6A
PLCD1A
SPOUT1
TCF3A
ZFP820
C17ORF100
C1ORF189
MIR21A
OLFR629
OR13C4
OR1N2
OR1Q1
PAGR1A
VMN2R111
ATOH1A
CALM3B
FKBP1AA
NAB1B
SNORA5C
ANKRD18A
GRIN1A
MIR3968

MIR632
MTRNR2L1
MYH15
SH3BP5B
ZNF140
APOA-I-1
OLR1061
OLR1307
OLR1316
OLR1418
OLR1589
OLR1691
OLR416
OLR661
OLR774
RGD1562885
TPH1A
ATF7B
CHCHD6A
EPHA5-AS1
FAT3A
FBXO30A
GPR75
GRM6B
OBSCNB
OLFR1061
OLFR895
OLFR904
OLFR935
OLR35
PRKCHA
RGD1563606
TNNT2C
GLB1L3
MAGEA2B
OVOS2
PRSS42P
RGD1359508
COL-49
KLK5
LRRC66
MIR431
SNORA30
SPATA6L
TAS2R20
TMEM221
ZNF300
ANKRD10A
ANXA1A
CTDSPL2A
FBXW14
FUT9D
FZD8B
HHATLA
HOXA1A
MIR190A
MTRNR2L8
NACP60E

OR6Y1
PTPRZ1B
RAS
REG1A
SEPT5-GP1BB
TPTE2P1
CLCA3A1
CRESTIN
HRNR
MIR584
MMP15A
NLRP4F
NOMO3
OLR1376
OLR1434
SLMAPA
ABU-11
ABU-7
ASS1P13
ATP5MC1P5
CAP1P1
CRATB
CSNK2A3
CST
CXORF51A
FRS2B
GHR1
GIF
MARK2A
MIR218
OLFR508
OLFR820
PCDH2AB12
PHBP11
RGD1561413
RPL17P50
RPL21P62
RPS27AP17
SEC11B
SERTM1
SLC25A5P7
SOUL4
ULE-3
ZDHHC16B
DARC
FAM229B
LHX8A
XLR4C
ARPP19A
C06A8.6
CLDN22
COL-140
COL-38
CYLC2
F58E6.5
F58G1.3
GCSAM
GLN-2

MIR548B
MSP-31
MSP-79
OLR1226
SLC2A1A
SNORA21
SPE-11
SSQ-4
CCDC189
DDX39AB
DSTNP2
FLOT2A
GST-8
KIR2DS4
PPP1R9BA
PRL7C1
RGD1562625
RGD1563818
RGD1566227
SERPINB9D
TEX264A
UBE2D1A
ACT88F
ARPP19B
CPT1AA
DAP1B
DAZ4
DEFB45
FFAR1
IL25
PRPH2
RABL6A
RPTOROS
SMIM2
SNORD101
SP3OS
TEKT3
TESCB
CERS2A
DNAJA
LILR4B
OLFR862
PRY
SET-30
SNORD20
SQSTM
TNIP3
ADCY2B
ATP6V0A1A
C19ORF67
CACNA1SA
CCDC180
CFL1L
CHCHD4A
CTL-1
DEFB49
DENND2DB
GSNB

MAPRE1A
MOTO
OLR1159
OLR143
OLR631
OLR717
PAX7B
PPM1AA
PRPH2LB
RNF34A
SLC1A3B
TIAM1A
USP51
FAM223A
FHL1B
KRTAP16-1
OLFR292
OLFR402
OLFR943
PSG17
VMN1R58
VMN1R-PS103
CUEDC1A
KCNIP1B
PVALB6
SEPT8B
SHCBP1L
SYPB
ZP2L1
C12ORF50
CYP2P6
HOMER1B
KRTAP4-9
LNX2A
OR1D4
OR4S2
RGD1564480
SHISA8
SHLD1
SLC22A20
TBC1D29P
TRBV14
ZFP942
AMY2
CISHB
CLPXA
ERVW-1
KISS1RA
MIR568
OLFR1420
PIN4P1
POMZP3
PTPRFB
RGD1562844
SNORA34
TNFSF13OS
U90926
BIVM-ERCC5

DUSP23B
GLIS3-AS1
INO80B-WBP1
KRTAP13
MIR365A
PPP2R3B
RPL23P8
SNORA49
SPATA12
SSH2B
URGCP-MRPS24
ADGRF6
ANXA2-PS1
ARGLU1A
ARHGAP35B
ARRB2B
C16ORF78
CLPXB
CYP46A1.3
DACHD
DCLK2A
DENND3A
ERRFI1A
FAM24B
FAM49AL
FUCA1.1
ITGA3B
JAG2B
KRTAP13-1
MARK3B
MGAT4D
MIR544
MLSL
MYOM2A
NAPAB
NHSL1B
NRCAMA
OR10A2
PLK2A
PMP22A
PRKAB1B
RAPH1A
ROCK2A
SEMA3FA
SLC4A4A
SMYD1B
SNORA80E
SPEGA
STAG1A
TMPPE
XKR8.3
YRK
CATP-3
CATP-4
CLEC-123
F17E9.5
FHL2A
KMT2BB

LBX2-AS1
MIR492
MIR570
MKXA
MSD-2
OLFR1055
SCGB2B27
SERPINB1L1
SNORD12
SSS-1
T13F2.9
Y43F8A.2
Y57G11A.2
Y69E1A.1
Y69E1A.2
BCL2A
C17H12.6
C32H11.4
F56D5.3
GST-13
GTF2IP1
KLK1C6
KRTAP19-2
MIR4488
MIR4787
MYO6A
SMIM13
UGT5B2
W06H8.2
ZFP12
ARPC5A
ATP6V0A2B
CPEB4A
CRYBA1A
CTHL
CYP4F8
DEFA-RS10
DESI1B
GOLT1BB
KRTAP9-1
LEG1.2
MARCKSL1-PS4
MIR101-2
MIR467C
MMP23BA
MTMR7A
OLFR157
OLFR67
SEMA4GB
UGT5B3
ANKHA
C10ORF53
C9ORF106
CCT8L2
CRATA
CYCB
FAM102AB
FAM180B

FAM205BP
FSTA
GJA5A
GSTA6
H1-7
IQCF2
LINC00852
MBD3L1
MDS2
MIR294
NAT15
NDEL1A
OLFR1168
OLFR225
OLFR314
OLFR531
OLFR536
OR6P1
SERPINA10A
SYNGR3A
TNN
UGT5G1
ZFP872
C10ORF120
CDRT15P3
CPS1-IT1
GGNBP1
HTR3E
KHDC3L
KRTAP10-11
KRTAP10-2
KRTAP13-4
LINC00523
LINC01565
LINC01600
LLCFC1
MIR124-3
MIR1303
MIR340
MIRLET7C1
NKAPD1
OR10K1
OR13J1
OR2T3
OR2Z1
OR4D11
REXO1L2P
RFPL4B
RTP5
SCARNA5
SNORD27
SNORD32B
STK35L
TBC1D26
TDRG1
CCDC41
FMO9P
LINC00602

MIR338
SNORA22
SNORD26
UNGA
ZNF107
CCDC60
GSTE5
KRTAP26-1
LRGUK
MIR1933
MIR3099
MIR3960
MIR7654
PDXKA
RAD51AP2
SIX4B
STRM
ZIM3
ANKRD30BP2
APOEA
ARMH1
DEFB36
DLX6-AS1
FMR1-AS1
HECTD2OS
HIST1H2BK
MIR378I
MIR605
OLFR1378
OLFR938
PPP1R14AA
RGD1559972
RPS18L1
TDGF1P3
C16ORF87
DOCK9B
FRMPD3
GCGRA
KB15
KCNRG
NEWGENE_1310139
OLFR1051
OLFR849
RAB1BA
RPS2-PS10
SLC39A12
SOCS3B
CRYGM2D2
MYH9B
PPM1LA
RNU6
SOD
EVLA
GPR34
GSTS1
IQCF4
KHDRBS1A
KRT97

LRATB.1
MBD3B
MIR759
NR2F1A
OR8G1
OR9K2
RASSF7B
TMCO2
TNIKA
TRIP10A
BRICD5
FAM66D
FPGT-TNNI3K
HAR1A
LINC00691
MIR502
MIR508
MIR519E
MIR618
OLFR635
OLR1069
PANE1
TWIST1B
ZFP266
CT62
CYP4F29P
ELK2AP
FTX
GABBR1B
KAAG1
LINC01011
MIPA
MIR181A1
MIR320
MIR637
MTL-1
OLFR1148
OLFR796
PRDX6-PS2
SIGLEC12
SNORA40
TAS2R125
UQCRBP1
ZNF43
C10G11.8
CAMTA1A
GADD45GA
GTF3AB
KPNB3
LINC00836
MYH9A
QDPRB.2
SNORD71
UGT1B4
BPIFB6
C16ORF82
CLDNF
DLEU7

GPR52
GPSM2L
LINC01781
LINC02532
MIR3613
MIR887
NANOS2
NAP1L4A
NLGN4XB
PINCR
PLGLA
RAB11FIP4A
RGD1311517
RGD1562890
RPLP0P2
S100U
SELENOW1
SEMA4BA
SLIT1A
SNAR-I
SNORD116-29
SOD3A
SPG1
TAS2R39
TIPARP-AS1
ZFP661
ZFP78
ZNF705A
ZNF732
ALCAMB
ATP6V1AA
C19ORF73
EVPLA
FTR83
GABARAPB
HER1
KRT8P12
LIPT2
OSBPL2B
PRC1B
RGD1560350
SLC25A36B
SLC7A8A
TMEM243A
TMSB1
TPRA
ZNF702P
ANO9
BA1L
LRRC36
SLFN12
CMTM2
GSTE6
MIR1-1HG-AS1
MORF4
OR4F6
VCX3B
ZNF724

C12ORF42
CASC9
CLLU1
COL18A1-AS2
DAOA-AS1
DEFA1B
FAM58A
FAM90A10P
GNA14-AS1
LINC00229
LINC00323
LINC01102
LINC01456
LINC01599
LINC01954
LINC02133
LINC02227
LINC02241
LL22NC03-63E9.3
MACROD2
MIR352
MIR3619
MTCO3P12
OACYLP
OR10A3
OR10A6
OR10AD1
OR10X1
OR1S1
OR2C3
OR4A5
OR4C12
OR4C45
OR4C6
OR4M1
OR4M2
OR4P4
OR4S1
OR52M1
OR56B1
OR5K1
RXRAB
SLC9C1
SNX29P1
STX12L
TAS2R42
TAS2R9
TRIM49
TTTY15
TTTY8
UMODL1-AS1
UNQ6494
ZNF564
CFAP46
CYP-34A9
FTCDNL1
LNCRNA:HSROMEGA
OLR1646

OLR530
TRGV2
LRRC30
SULT
CCN2B
CD99P1
COX5AB
MAFGA
OLFR1451
RNU5D-1
SLC7A10A
SVA
DEFA20
ESRRGB
FAM109A
HNRNPABB
IFNA6
IGKV4-86
JMJD2C
LINC00917
NOTS
NUDC-PS1
OLFR1444
OLFR378
OLFR720
OLR1424
OLR232
OLR397
OLR903
PRAMEF20
RN7SL2
SEMG2
SNORD3B1
SNORD77
TLM
VMN1R229
VMN2R81
ZNF208
CFAP94
CYP2D37-PS
GID8A
PPP1R3DA
PRKCDA
PRPMP5
RGD1304770
RGD1565355
SNORD41
SSRP1A
ST3GAL8
TMEM110L
CCL34B.1
MIR1949
PALM3
PCOLCE2B
SEC6
CNC
GSTT1L
HK2P1

MUP7
NBPF4
OLR1381
OLR1481
OLR1701
OLR826
PIT54
PRL7B1
RPSAP12
SHHB
SSTY1
YBX1P2
ZBTB11-AS1
ZBTB3
ZSCAN5A
CMTM2B
DNMT3BB.2
OR10A4
OR14J1
PIAS4B
SNORD1C
TBXTA
ZNF215
ZNF717
ANXA2P1
ABU-6
ABU-8
CDHR1A
CLEC2L
FAIMB
GOLGA2P2Y
GPM6AB
HAPLN1B
KRT18P65
LASS2
LRATB.2
MAP7D2B
MS4A6BL
NPM1P8
OLFR1537
OLR1058
OLR1064
OLR1364
PQN-54
RNF128A
RPL15P11
RPL26P11
RPS2P18
RPS2P28
RPS3P6
RPSAP47
SCNN1D
SPDYE21
SYN2B
SYPL1
TMEM173
XKR7
YWHAQA

APPB
CEP295NL
COL-120
COL-8
DMRTC1C2
F52H3.6
F58A6.9
FAM149B
GSP-3
LYS-4
MIR181B-1
MIR3095
PDZPH1
PRR18
RPL5B
SSP-16
SSQ-1
C14ORF156
CLDN15A
DARMIN.L
INSM1B
PLXNB2A.1
PRAMEF5
RPL13AP5
SCARNA20
TRDV3
ABI1A
BGLAP2
C9ORF64
CSNK1G2-AS1
CTAG2
DHS-8
GCS-1
GST-20
NUTM2D
RANGAP1B
SNORA67
SYT6A
UCMAB
ACY3.1
C9ORF153
CCDC173
EIF4BP1
GAS8-AS1
HPXB
IGHV1-77
KRTAP14
OLR1351
PCDH10B
PRX3
SAO
SLY
SNORD1A
CTDSPLB
EIF4BB
EIF4E2RS1
EXT1A
FRYB

MYH11A
OGT.1
OLFR1348
OLFR798
SLC25A1A
SPETEX-2G
STMN1B
CWH43
DRB1
OR115-14
PARK
PES
PHF11A
PTK2AA
SELENOU1B
SNORA3
TPM4B
ZNF843
CASQ1B
CT45A3
DEFB24
OLFR435
PCDH1GB9
SEMA6E
VMN1R28
ZFP352
ZFP940
CCDC144CP
CLCA4L
FAM181A-AS1
HOXB2A
HOXB8B
LINC00923
MAPTB
MARCKSL1A
NEFMB
SERPINB9E
SNORD3B-1
SUGT1P1
TTC41
TUSC7
ARL6IP5A
FAM215A
KRT6
PRSS56
TEX47
ZNF227
CYP2C79
DPYSL5A
FAM24A
FBXW11B
IGKV14-111
MAGEA5
MIR6540
MIR6933
MIR7025
MIR8106
OLFR441

RFPL4
RIN1A
SNORD42B
TAS2R144
VOM2R37
ZFP943
ANXA2P2
C4ORF47
CALM2A
F8A3
FAM9C
LINC00869
MIR323B
MKNK2B
PCMT
SMG1P7
SNORA16A
SNORD10
SNORD97
AGR1
ANKIB1A
AQP9B
ARPC4L
CTSS2.2
DACHA
DHRS11B.1
DOPEY2
IFI208
MIR30C-1
MMP30
MORG1
OR51B2
OR7E156P
PANK1A
RHOCB
RORCA
SSX4B
STIM1B
ARGK-1
C2ORF50
CIPCB
CRYGM2D6
LDB2A
LYPLA2P1
MIR4497
MIR4508
MIR595
MIR653
RNF223
SPAAR
ST5
SYNE2A
T16A9.5
T27E7.1
TGFBR1A
TVP23A
Y1A5A.1
ZSCAN9

ATP5MF-PTCD1
FAM154A
GNAI2A
MIR4521
MYCLA
OBOX5
PRKCDB
RGD1564899
RHOX2
RHOXF2
SOX11B
ZFP174
ZNF101
AHSG2
ASIC1A
BAIAP2A
CBSA
CYP2C80
FSTL1A
GDPD5B
GNAI2B
HMGCLL1
LILRB3L
LRP1AB
MAPRE1B
MIR490
MYL9B
OLFR432
PBXIP1A
RGD1561232
RLN3A
SLC22A6L
SLC43A1B
SNORD51
SULT1ST4
APOL5
ARF4A
C10ORF55
C1ORF194
C20ORF197
C5ORF47
CDCA7A
CRYBA1B
CRYBA1L1
DSCR10
DSCR8
FAM170A
GFY
H2AC24
KCNK17
KCNQ1DN
KRTAP10-4
MHC1ZAA
MRGPRX4
NUCB2A
OLFR1388
OR10H2
OR2A2

OR51F2
OR51Q1
OR8H1
OTOL1
PCDH10A
POM121L12
PYY2
RPL29P2
SNORD115-11
TAX1BP1B
XLR
ZNF835
DNAJC25-GNG10
HAS2-AS1
LINC00511
MIR4492
MIRLET7F-2
TSC1A
ZFP879
ANO8
CD1B
CTSS2.1
CYP4F40
DEFB13
ENKD1
IGLJ3
METTL11B
RBM4.3
SPANXN1
ZNF529
C1GALT1B
C24H7ORF50
C87436
CRABP1B
CTL-2
EP300B
HPCL2
JHEH2
KHDRBS1B
KIAA1524
KLF7B
MSX2B
MYBPHB
NFIL3-5
NR2F5
OLFR434
OLFR68
OR52A1
RARAB
RGD1308706
RT1-T24-3
SERPINA3B
SRSF5A
ZFP869
AQP7P3
ARAP1-AS2
ATXN8OS
FAR2P1

LINC01060
MIR378E
MIR4485
MIR7704
MS4A17A.3
NPIPB6
PCOLCE-AS1
SLC6A1B
CADM1A
CCT8L1
CRIP
KIF20BA
OLFR1393
OLFR1480
OLFR676
OLFR974
TRAF4A
VMN1R4
YY1P2
ZNF256
ANG1
COL27A1A
DYNAP
FAM225B
HBBE1.1
ILDR1A
LIPEA
MSI2A
MYBPHL
PAFAH1B1A
PCDH2AC
RGRA
S100V2
SAMSN1B
SLC29A4P1
ZFP938
CALM3A
CEACAM21
CETN4P
ELA3B
HTN1
IGLV3-21
OLR1353
PRY2
RT1-A1
SNORA50A
SPON2A
TMPRSS11G
USP9
ZFP667
BMP2A
CA15B
CAPN2L
DTX2P1-UPK3BP1-PMS2P11
GUSBP15
HIST2H2BB
LINC01002
LINC01347

MDGA2A
MIR563
MIR564
OLR1070
OR12D2
PRKAR2AA
PRL2C1
SNORD4B
SNORD63
SNORD95
SYNGR2B
ANKRD18B
FBXO47
KLRA7
LINC00964
MANCR
SAMD7
SNORD57
USP17LD
ZNF205-AS1
AANAT
APODA.2
CHICO
CLDN15LB
CRY3A
FOXQ2
MANEAL
MIR208B
MSX1A
NR1D2B
OLR285
OLR675
OLR786
OLR821
TMPRSS11E
UGP2B
ADAM1B
BGNB
CALUA
CALUB
HMGB2B
HYAL6
IGKV1-132
IGKV1-135
KRTAP13-2
LINC00693
LINC00880
LINC00955
LINC02158
MIR1188
MIR124-1HG
MIR2278
MRPL23-AS1
NHEG1
OLFR26
PCBP1-AS1
PCXB
PDI

PSMD4A
SLC38A5B
SNAR-G1
SNORD116-11
TMEM176L.4
VN1R1
ZFP939
ZFP984
ACT87E
FMO6
GCGA
MYOZ3A
OLFR1153
PRL3D2
SOX21A
CGM4
FTN-1
OLFR649
PB1
SNORD44
SNORD79
UGT-1
CLDN13
D7ERTD443E
FAM27E5
MIR1304
MUP13
OR1J4
OTPA
RPS8A
TRIM48
VEM-1
WDR20RT
XAGE3
AADACP1
ACTRT1
ADAM3A
AGAP11
ANKRD26P1
APOC1P1
C1ORF87
C2ORF16
C8ORF86
CLEC17A
CYP4Z2P
DAOA
DEFB124
DHRS4L1
DNAJC27-AS1
ERV3-1-ZNF117
FAM157A
FRG1BP
GAGE2E
GSDMCL2
KIAA2012
KRT8P41
KRTAP9-9
LANCL1-AS1

LHX1-DT
LINC00161
LINC00200
LINC00486
LINC00578
LINC00824
LINC01096
LINC01104
LINC01187
LINC01317
LINC01411
LINC01465
LINC01551
LINC01772
LINC02210-CRHR1
LINC02351
LORICRIN
LSP1P4
MAGEB10
MAMDC2-AS1
MS4A18
MS4A6E
NKAPP1
NUDT16L2P
OR2M3
OR4A47
OR51D1
OR6C74
OR7A17
OR8U1
OR9Q1
PACRG-AS1
PDIA3P1
PLEKHM1P1
PLUT
RGD1561916
RGS21
RNF217-AS1
SFTA3
SNORD115-7
TCHHL1
ZBED6
ZNF136
ZNF664-RFLNA
ANKRD36BP2
ANP32BP1
AZIN1B
HSPD1P5
NOP56P2
OLFR1030
PDL1
RGD1560559
RPSAP30
SNRPGP10
TAAR7A
ZSCAN16
CFAP99
NFYC-AS1

OLFR30
SNORD53
SRSF3A
STX18-AS1
TCL1B1
TCL1B3
TUBA8L2
ATP2B3A
CALCRLA
COL-19
GSKA-3
MIR557
NRXN2A
OLFR374
OLFR691
TGIF2LX
W02B12.12
ZFP607A
ZYG11
ARHB
ASAH1B
GST-14
GST-16
NBPF15
ZFP455
ADM2A
C2CD6
CYP2X9
SOX4A
THAP9
ALPHATUB84D
CRXOS1
FAM129A
MIR625
RGD1560108
SNORA1
SNORD6
TAF1L
C19ORF33
FAM209
LARS1B
MDR50
PPAN-P2RY11
PRR12B
SELENOT1A
SMIM23
TEAD1B
CAVIN2B
IFNG1R
JRKL
MAPK14A
MICOS13
NRARPB
OLFR433
OLFR960
CRYBA2B
CRYGM2D13
MDKA

MIR467F
MYH16
RHOXF1
SERPINB1L4
SNORD61
STX3A
TMEM191C
FGFRL1A
MIR881
OR5AR1
SLC43A1A
CLEC2E
ITPKCB
OLFR1026
PDCD4B
UGT5D1
ZFP454
ABHD14A-ACY1
IRGC1
METTL10
OLFR821
RGD1307461
SNORA71B
SPIN2C
TCEB3
VMN2R1
ZFP286
ZFP605
ANK3A
ASCL1B
ATF5A
ATP6V0CA
CYP6A8
ELOVL1B
FHL2B
FSCB
KLF6A
KLHL24A
MIR190
MIR367
NOV
NUTF2L
OLR1696
RPRD2B
RRBP1B
12-Sep
TRNAU1APB
VIT-1
AFAP1L1B
CROCC2
DCAF10
ECR
EHD1B
IRG-5
MIR466D
MIR517A
MSP-38
PIRA6

SPCH-1
CCDC54
CYP3A12
DOD-17
GST-39
GST-6
HER9
MIR3178
MUL-1
NUCKS1A
OLFML3B
OLFR996
OLR63
SCAMP1-AS1
TAAR7E
TCP10B
TMEM30AB
DHRS12
EEF2L2
GLS2B
HNRNPA0A
HSP-16.41
LOXL2B
RXRAA
SELENOO1
SLC13A5A
SLC25A15A
SLFN11
SULT1ST5
SULT3ST2
TAS2R129
TSPAN18A
UGT5B4
VOX
ZFP541
BEND2
CYTH1A
IGFL4
KLF5A
MIR496
OAS1D
OLFR149
OLFR457
OLFR92
PATE2
PRRX1B
RDH19
TNFRSF9A
ZFP583
ZFP846
ARL3L1
CELSR1A
CRYGM7
ELOA3BP
ELOA3P
ESRRGA
LINC00311
MAFG-DT

MIR3065
N5
OLFR638
PCDHGB3
PRR35
RNF113B
RQCD1
SIK2B
STARD4-AS1
TBC1D3G
TGM2A
ARHGEF26-AS1
C10ORF95-AS1
OLFR1350
GRN1
LINC-PINT
PAX7A
PTGES3L1
SPEER5-PS1
AB124611
ACTC1A
EPHX4
IGKV6-15
IQSCHFP
LINC00702
MORN5
OLR1002
OLR1455
RPS3A1
SPTLC2B
ZNF226
ADOB
FZD7A
HERC2P4
RNA28SN4
CCDC135
CIARTA
EIF1AXB
INPP4AB
LY49I4
OLFR549
PVALB9
RGD1563365
SLC20A1B
SLCO1B7
CR1
DCAF4L1
DIEXF
GK3P
INE1
JDP2B
OR1L8
PI4KAP2
PRUNE
RGD1566359
SLC34A2A
TEX28
BTBD18

CCDC187
CLDN11A
DCAF15
EIF5AL1
LINC02035
MIR612
MPDU1A
NOXRED1
RARRES3
RNF216P1
SNORD82
SPDYE3
TEX33
ZFP267
ZFP961
C19ORF38
C6ORF99
CFHL4
FAM86HP
HPN-AS1
LINC01341
OR52K2
RBM12B-AS1
RPS10P7
SERPINB1C
SLC30A1A
SNORD116-4
SNORD47
SNORD58C
SYNE1A
ATP5F1
GADD45
MKL1
PDE4BA
SETSIP
TAS2R46
ZFP712
ASMTL-AS1
CLEC4B2
EEF1A1P22
GOLGA8EP
HSPD1P6
L1CAMB
MAP1LC3BP1
MIR219A-2
MIR7021
NPIPB1P
SUMO1P3
COL5A3B
CRTAC1A
DNAAF6
FTH1P2
IPF1
LINC01146
LNCOG
MIR1469
MIR1973
MIR548H3

OBOX3
OLFR513
OR6C65
OR6T1
POM121L1P
ROPN1B
SIRPD
TEDC1
ZNF300P1
ARL4AB
ATP5F1EP2
CD74A
EPHB3A
MIR101-1
NEXN-AS1
RHOAB
RT1-CE1
RYR1B
TCL1B2
ABCC6P2
FCRL4
GML
HMGB4
MIR3591
MIR651
NUTM2A
TPSD1
TTBK1
UCHL3-PS1
UGT-13
ZFP951
ANKRD20A9P
ATAD3B
BAGE
BMS1P2
C1ORF127
CARD17
CCDC197
CIRBP-AS1
CYCSB
CYP1B1-AS1
DDX12P
ELOVL2-AS1
EOMESA
EPCAM-DT
GLIDR
GNG12-AS1
HEPN1
HORMAD2-AS1
KLHL41B
KRTAP11-1
KRTAP5-9
LINC00189
LINC00461
LINC00469
LINC00592
LINC01169
LINC01305

LINC01340
LRRC52-AS1
MAGEC3
MAJIN
MCPT10
MEF2C-AS1
MEG8
MIR646HG
MYLPFB
NLRP11
OLFR1410
OLR1750
OR10J1
OR1G1
OR2B6
OR52D1
PLGLB1
POTEH
PRR30
SERPINB3B
SNORD42A
TAAR6
TAS2R13
TAS2R30
USP2-AS1
VN1R4
VOM2R44
VTRNA1-3
ZNF337-AS1
ZNF804B
ZNF860
AKR1A1B
AMOTL2B
HSP68
IRBP
ITGB1B.2
LIPCA
SNORA33
TFR1A
OLFR830
PRL8A7
RGD1565685
SQLEA
C2ORF81
NXF2
OLFR33
PPARAA
CCT8P1
CTS6
HBEGFB
LINC01512
MAILR
MBL1P
OLFR65
OLFR829
OR7D2
PMS2P4
RPL34-PS1

SRSF1A
USP26
CALM2B
CED-4
KRTAP1-1
QDPRA
RABL2B
ZFP300
ZFP719
ARNTL1A
CALM1B
CBSB
CBX1A
COL5A2A
CRYBA2A
CST12
HSP40
KDELR2B
MIPB
MIR687
OLFR1262
OLFR1395
OLFR786
PHACTR4B
RGD621098
RT1-M2
TMEM210
VCANB
ATP1B3A
COL11A1A
DYNLL2B
EK1
EPDL1
ETV3L
FHL4
GALCB
GRNB
IVNS1ABPA
LINC00622
MGST1.2
NME2A
OR10A5
PI4KAP1
PRL8A9
RNASEKA
RPL13AP20
RPL13AP6
SCARNA10
SERPINB3C
SNORA9
UBA6-AS1
C04G2.9
CPT1AB
CYP3C3
GSTM6L
HIST1H2AIL1
ASCL5
HRASLS

LINC00899
MIR4324
SMG1P1
TRIM34A
UGT5E1
WWC2-AS2
BACE
HRAT92
MIR1839
SCGB2B2
XRCC6BP1
CLDN3C
F9A
FLNCA
GMPPAB
JAM2A
LINC01605
MIR764
NID1A
PRG4B
PSMA6A
SCP2A
AACSP1
CTAG1A
DHRS3A
GSTD4
HLA-DQA2
KIR3DL3
KRTAP4-3
LINC00305
MIR766
MMP14A
OLFR1252
OLFR190
OR1M1
OR51G1
PRDM16-DT
RFPL1
RGPD3
RNF212B
RNU1-3
RXRGA
TNXA-PS1
ZNF324B
ZNF454
ZNF479
DOCK8-AS1
FPR-RS6
IQANK1
ITGAD
NBPF9
PAX8-AS1
SNORA50C
SSR4P1
ABCG3L3
ARF2B
CRY4
DUPD1

KRTAP15-1
MTNC
OLFR705
OLFR914
RGD1359127
ZFP341
ADD3A
CACNA1AA
CTSM
EAR12
FAM63B
HOXB1A
MOXD2
OLFR1396
PILRB1
SFRS1
SNORD58A
TDPOZ1
TH2
TMEM225
TMEM238L
ZFP811
ADAM3
CCDC196
DEFB6
HSP-3
LCE1G
MIR196A
NARG2
NRP
SNORD28
SNORD30
YAE1D1
BCL6AB
GVINP1
OLFR695
POLR3GLA
D13ERTD608E
MDKB
NWD2
SNORA25
SPACA9
TPBPA
TUBA8L3
ADAM6
ARL17B
CEACAM18
CREMA
CYP-35B2
DEFB29
GOLGA6L5P
LINC00888
MDR65
OLFR187
OR4Q3
RNA18SN4
SNORA62
TEX35

BCO1L
EWSR1B
KRTAP21-2
MIR498
NACA4P
PCTK2
PNP5B
SIGLEC15
TCL1B
AATBC
CXADRL1
DIAP2
FOXL2NB
LINC00313
LINC01270
MEN
OLFR905
OSBPL3A
RAB11B-AS1
RLTPR
SHFM1
TMEM254-AS1
WDR52
ZFOC1
ALDH2.2
C19ORF44
CED-9
FAM49A
IL17R
KLF12A
KRTAP5-1
LAMB4
LINC00319
POT1B
RGD1559896
SATL1
ZFP248
CRISP4
DAZ2
LINC00624
MUC7
OLFR122
SNORD83B
TTC16
BRP44
CALB2B
DPPA5A
MFSD2AA
MIR471
PRL3A1
RNF113A1
SLCO6B1
SVAL1
TSSK1
ZNF252
ATF4A
CRYGMX
FDCSP

RGD1310166
RGD1564804
YWHAE1
ANKRD30BL
AZGP1P1
C17ORF98
C1ORF186
C1RL-AS1
CCT6P1
EGFEM1P
GMDS-DT
GPR62
GPR89A
GRK7
HCG26
HLA-F-AS1
IGF3
IQCJ
LINC00114
LINC01235
LINC01237
LINC01559
MS4A5
NBPF19
NLRP8
NPSR1-AS1
OLFR771
OR2L13
OR2W1
PCDHB19P
RACGAP1P1
RGD1562987
SEMA6A-AS1
SLITRK3
SMPD5
SNAP25A
STMP1
STXBP5-AS1
TAS2R5
TBC1D3E
TMCO5A
TTC23L
ZNF80
ZSCAN1
CYP1A5
ECT2L
JAFRAC1
RD3L
TESCL
ZFP786
ALOX8
MMSA
PIGBOS1
PVALB3
RUVB1
TCL1B5
ADCYAP1B
AGE-1

EPS8L1
FAM223B
LIPO1
MDH1AA
MIR486-1
MIR675
OLFR111
SUPT5
TLR2A
XLR5C
CYP2J8
DSG1A
KASH5
KDM6BA
MFSD2B
MRGPRA2B
NAG3
NOCTA
OLFR345
PALM2
PVALB1
RERGL
SNORA48
SSX2
ZFP563
ZFP825
APOA4A
BRD2A
ENO
GPX-6
GSDMA2
HCAR1-4
HMOX
HSF-1
HSP83
LINC01619
PGP-5
TNNT2A
CXORF65
D3ERTD751E
HORMAD2
IGLC2
SCARNA13
SNORD75
ATP5MC3A
ATP5MPL
CBLN8
GST-10
HOXB5A
LINC01094
METTL13
OLFR1384
OLFR611
PAPSS2A
PGR15L
SELENOT2
TAAR7B
ATP1A1-AS1

C12ORF54
C7ORF61
MIR630
OLFR1122
OLFR56
OLFR815
TPMT.1
WFDC11
ZFP777
ZNF169
ADAM20
C16ORF54
CSAG2
HEATR9
KLF2B
KRTAP19-3
LINC01137
MIR660
RXR
SPDYE4
TMSB15L
VTRNA2-1
FCGR1B
LCE1I
METTL11A
MIR327
NDUFA6-DT
CED-3
CRY3B
RGD1565616
TXNIPA
ZFP81
ZFP946
AGAP7P
ANKRD36C
CEACAM7
EGFRA
FAM198B
FAM209A
MIR151A
NSUN5P1
OLFR18
PAX6B
PSA
RBP4L
RGD1564664
SMIM10L1
SNORA68
UNC5B-AS1
AHSA1B
FGFR1A
HBBE2
HSPA4B
NUTM1
SNORD3D
VPREB2
FABP4B
MYLZ3

SNORD32A
CROCCP3
H2-M5
HMGN5B
HTR7P1
KRTAP4-7
LCE1M
LINC01138
PMELA
TAS2R43
HBA-X
PRL7D1
RARGA
TFPIA
TLR21
APOBEC3D
BNIP5
C11ORF94
C4ORF36
C6ORF120
CCDC7
CFLAR-AS1
CHRFAM7A
CRYBB1L2
DDX11L2
FAM222A-AS1
LINC00839
MIR19B
MIRLET7BHG
SCINLA
STYXL2
ZNF286B
CYP2N13
DAF-2
RHCE
ZFP146
ZFP458
AKR1C8P
ATP2B1A
PFPL
PLCZ1
ZNF490
CTSR
DEFB9
ELOVL7B
H2-L
HE1.3
JAKMIP2-AS1
KRTAP3-3
OR51I2
PFK
ABCG3L1
SLC20A1A
TMED1A
C18ORF21
NPHP3
ABCA14
BLOC1S5-TXNDC5

C5ORF66
CATSPERZ
CMC4
CPXCR1
DTHD1
ELOA2
FAM221B
FAM86DP
KIAA1644
LILRA1
NUDT9P1
PRECSIT
RT1-M5
SCART1
SIAH3
SIRPG
SLCO6A1
SNX29P2
SOX9-AS1
SP2-AS1
ZNF155
APOBB.2
C22ORF42
CAPN14
CC2D2B
EGFEM1
FBXO16
JMJD1C-AS1
METTL7A3
SIGLEC7
TPM3P9
URB1-AS1
CALHM4
FAM29A
MAGED4B
OLR1366
PRL2C5
PSMC1B
ZFP334
ZNF395A
ARHGAP11B
C6AST3
CCDC172
CEL.2
EEF2B
LINC00665
MIR761
PNMAL1
SIRPB3
ACSM4
BLID
C7B
CALR4
CYP2J11
FAM86EP
HNRPM
MAT2AA
MYCA

OR2H1
SR140
USP50
ZFP804A
INTS11
MAFBB
MIR758
OLFR482
OLFR608
OTUD6A
PRP2L1
RBM44
SOWAHD
ZNF781
ZNF837
LINC01558
SFTA2
SPESP1
WFDC10
ZNF813
AADACL3
AMY1C
CA5BP1
EAT-3
ERBB3A
ES2
FZO-1
GCSHB
LINC01088
MIR325
MIR432
PFN1P2
PRSS38
RPL17P33
RPSAP52
SPANXN5
TPTE2P5
ZNF17
ZNF658B
AGAP4
LYG2
NHLRC4
PPP6R1
SNORD14E
CKI
H2-T3
IGFN1.1
MIR1260A
NLRP9B
SWSAP1
TBC1D3B
TCSTV3
BNIP3LB
DEFB3
IGHE
PHGR1
SODH-2
GYLTL1B

OLFR288
OLFR332
RT1-CL
XG
ZFP532
ZNF276
AIP-1
HISCL1
NLRP14
NUP42
PRL2A1
SLC4A1A
TAS2R105
BIRC8
MIR26A1
MIR874
RAB40AL
RGD1560010
RT1-T24-1
ANKRD20A12P
FAM41C
FAM86FP
LINC00926
NBEAP1
OLFR524
P4HA1B
PRR19
PVALB4
RGD1311595
SIGLEC14
THOC4
CD9B
DEFB2
ERV3
FTH1P3
LOXL2A
MMP14B
PSG28
SKIV2L2
ZNF417
GATA3-AS1
IL6STP1
MATN3A
MRGPRB2
OR1D2
PRPS1A
SELM
TCTP
ACTC1B
LCN3
OPN1MW1
ARPIN-AP3S2
FTH1P20
GGTLC1
MLNR
PA2G4B
SIGLECG
SMG1P5

SNORA23
SNU13B
TAS2R50
C9ORF47
CYP2B21
MIR129-2
PAGE1
ZFP558
ZNF565
ZNF90
ACTB2
CNBD1
DELEC1
DUXBL1
FAM218A
GAS2L2
IGK-V1
IRGC
MAFBA
MAGEB1
PLSCR5
RAD21L1
RNU5F-1
C1S2
PXT1
RGD1311251
VTG5
DPY19L2P1
DRGX
FBXW12
HCG27
HOMEZA
MUT
NBPF11
OLFR1110
ZNF311
FAM71E2
HISTH1L1
LINC01503
PLEKHG7
VCX2
C9ORF78
GAS6-AS1
MIR1249
MIR147
MST1P2
PRSS46
VMN2R89
ZNF205
ALKAL1
HIST3H2BA
WHAMMP3
FOXG1B
MIR4516
NBPF8
OLFR1284
PLA2G4AA
RGD1566085

SHP
SVAL2
ZNF826P
H2-M11
IGHA1
NEPN
HIST1H2AF
KRTAP1-5
NRK
SMKR1
CAV
CHP
GTF2H2B
HBB-BH1
LRRC63
PROX2
SELENOW2B
TUBA-RS1
VMN2R26
ZNF790
HIST3H2A
MIR1228
PRR5-ARHGAP8
RHEX
RT1-CE4
SOD-4
ZNF252P
ALDOART2
AQP3A
C12ORF43
IDH
SDHAP2
AT
FAM65A
GBAS
LHR
PONZR4
PRKRIR
TPBGL
TUBB7P
VIG-2
GRP94
HSD17B7P2
ITGB3B
MFAP1A
MIR135A
NR5A1A
RGRB
ZNF283
AXDND1
CCL25B
CYP2B4
FLICR
MEF2CA
MIR299
PASD1
MUC
PRL3D1

RGD1306063
TADA3L
ZNF468
CAHZ
INSYN2B
PET100
PRR29
ACSL4B
C1S1
CKMT2-AS1
LINC00960
LYPD8
MFSD2AB
MIR924HG
OLFR555
PCDHB13
PNMA5
PRR21
SLC3A2A
SPACA3
SUGT1P3
SULT1ST6
TMEM240
USP17L2
C10ORF99
CYP3A41B
HE1.1
MYMK
NPFFR1
OLFR339
TAS2R38
TMEM174
ZNF662
ZNF740
FMO-2
HOXA11-AS
MIR208A
MIR3074
MYLPFA
PRL3B1
SMAD5-AS1
THUMPD3-AS1
OR51B4
RGD1565033
TRA
ZFP654
ZNF556
MTNB
RUSC1-AS1
VANABIN5
CXCL8A
ZNF595
COX6C-PS1
STRC
ZNF233
FAM106A
PF4V1
RNU1B6

HLA-DRB3
PCDHB20
ZFP382
ZNF737
CMTM1
IL36B
SPDYE8
TOB1-AS1
ZNF674-AS1
MFSD6L
STC2A
TRY4
CBR1L
CESL1
MIR18B
TGFB1A
TTLL13
ZCCHC11
ZNF526
CYP19B
IGF1RA
LCE1L
ZNF594
ANKRD30B
GNLY
LRRC74B
SDHAP1
SNORD96A
ZNF497
ZNF563
ZNF699
BRS3
CKMB
FCRL6
HSP22
KLK1C10
MRGPRD
NR2F2-AS1
THAP9-AS1
DBX2
GPR25
ACAT2L1
BRD3OS
RGD1308742
ZFP161
LRRC69
OLFR153
TMPRSS11A
DNAJB1B
DEFB14
KLF9.L
RGD1560242
SYCE1
CLEC18A
JUB
SH2D7
SMIM43
ZNF71

ZNF785
CCN6
DAO.1
FNDC8
LEPA
LINC00265
MIR500
PDCD4-AS1
TIGD6
TUBA3FP
WDFY3-AS2
GSTS2
YF6
ADAD2
C7ORF31
CFAP92
CYP2K4
ERVH48-1
SOA
TYRP1B
ZNF772
C8ORF82
FSIP2
TRIM58
C16ORF86
MIR125B2
MZT2A
RDH8
SPI15
TCIMA
ZNF845
C3A.1
CFAP206
CHK2
IGHG3
PCK
PNP5A
PSME2P3
TM4SF
ZNF48
ZNF502
ZNRF2P1
C5ORF63
GUCA1C
HSP-60
LINC00957
LINC01018
ZNF667
OLFR633
TBX5A
RNASE3
RPS3AP6
ANKHD1-EIF4EBP3
LGALS15
SULT1ST1
DMRTC1B
FREM3
GSTD2

KIAA2013
PGLYRP4
RGD1563888
WFDC6A
NPIPB5
RNF212
SGK2B
GPRK2
KIFC5B
MIR466F-1
MIR467A-10
MIR467A-3
MIR467A-6
MIR669A-2
SCGB1B21
TRAV13N-4
ZNF75A
ZNF765
MIR770
NLRP1A
MTNA
ZNF607
C12ORF65
FFAR3
GPR165
ASMT
PKD1P1
ZFP239
ZNF571
C14ORF39
CCDC42
IFNG1
IZUMO1
MIR31HG
ODF4
OR51E1
PARA-LIKE
ZSCAN23
ESRG
PABPC5
PHF11B
RT1-CE3
SCIMP
ALPHA-EST9
CYP314A1
JPX
KLF3-AS1
MIR181B-2
ZFP184
AMBN
CCDC183
LRP2BP
PCDHAC1
RNY1
SERPINH1B
ZNF132
ZNF334
NHR-121

PBP2
LINC00515
ZFP407
ZNF396
RS11
VSIG8
CYP11A
LILRA4
ZNF154
ZNF701
ZNF763
FCGR3B
KANSL1-AS1
MCTS2
MIR205HG
NPAS4A
SLC6A10P
CTGF
FER1L6
MIR1290
ZNF14
ZNF764
EAR-PS9
RPSA-PS4
VMN2R14
VMN2R27
SOD-1
CROCCP2
HEN1
MIR193A-1
MIR2985
RPL7P26
PPEF1
REN2
ZNF879
CEMP1
DLGAP1-AS2
ZNF836
FSBP
TMEM28
ZFP874B
FAM71B
GADL1
IFNL3
ATP2A1L
C1ORF174
DYNLL1P4
ELOBP2
GAPDHP60
HLA-DPB2
KRT18P18
MIR6333
MTRNR2L9
OLFR976
OLR1442
PABIR3
PFN1P9
RATNP-3B

RNA5SP191
RNA5SP202
RNA5SP336
RNA5SP481
RPL10AP2
SNORD48
TMSB10P1
TRI-TAT2-2
UHG1
VOM2R-PS142
ZNF345
ZNF707
EPPIN
H2-M10.2
IFNA
STRL-1
TRX
ZFP944
IFNA8
OLFR601
TRMO
TTC28-AS1
CABS1
CLUHP3
FAM124B
FAM63A
MIR300
ZNF174
CKMA
VEGFAA
ARRDC3B
ASL1
OLFR1095
OLFR412
SPIA1
TMEM202
USP17LE
VMN2R20
CXCL18B
DLDH
NCAM
BFB
VSTM2B
ZNF253
ZNF669
ZNF69
CYP-35A5
ELA3L
MFRP
MIR669A-3
OLFR1352
SPATC1
SSTY2
CYP-35B3
MIR3076
KIR2DS3
CFAP58
IFNA17

LINC00467
NINAB
POM121L2
TMEM99
MIR501
ACTN2A
ALKAL2A
AP1S3A
C17H15ORF52
CDAB
DOCK2-LIKE
ELFN1A
FMNL1B
GFI1AB
GHSRB
HBB2
KCNAB1B
KCNK10A
KCNK1B
KIF5AB
MPP7B
MRVI1
PDE1CA
PROZA
PTPN9A
RANBP3B
RHOBTB2A
SLC1A7B
TMEM151BA
USP43B
ZFP646
ZNF1159
CIBAR2
PINLYP
SLC26A8
SRGAP2C
NHR-14
NHR-69
TESTIN
ZNF780B
C06H2.2
C12ORF76
SOX5OS3
VMN1R184
SKIP
BAIAP2-DT
KAL1
STPG4
MIR1901
MIR1982
MIR28B
MIR3083
MIR3088
MIR3108
MIR3113
MIR344G
MIR5127
MIR669J

MIR873A
SUN3
TTC31
IFNA16
ZSCAN30
CFAP161
CYP2A21-PS
ADAM26B
DEFB46
HB
OLFR58
VMN1R124
VMN1R64
ERICH3
OLFR1404
ATG1
MIR3573
LCTL
APOBEC3A
TMEM17
ZBTB11OS1
ZNF470
ELA2
BEST4
MARCHF11
TCP10L
ACTG1P9
CUTALP
EPT10
EPT13
EPT2
EPT6
EPT9
HIST2H2AC
LIMD1-AS1
MIR374
NTAQ1_ANOGA
NUBP1_ANOGA
OLR1757-PS
RPL27-PS1
SHISAL2B
SNORA15
SNORD113-4
SNORD126
ABHD15A
ADD3B
AKR1B1.2
ANO1A
ANO9B
CIRBPA
CLDN23.2
CRACR2AA
CUL5A
DDX61
DEPDC7A
DEPDC7B
ELAVL1A
EPHA2B

FZD7B
GIG2O
GRB2A
HIST1H2A6
HIST1H2A8
HSP20
IFI27.1
KRT18A.1
LFT2
LINC.SETD1BA
MC6ZB
MCF2A
MIER3B
MS3_0012184
MS3_0015983
MS3_0016535
MS4A17A.2
MSL2A
MXTX1
MXTX2
PITPNC1B
PKDC
RASSF4A
RBM14A
RNF165A
RNF213B
SLC7A1B
SPHM
TRIM35-29
TSG101B
VG_1
ZMP:0000000991
ZNF319B
ZP3E
ZP3F.1
AKT-1
CRZ
CYP2J13
CYP4G7
EEF1DP3
EIF3FP2
FRMD8P1
GALP
GSTE4
REA
TREM5
LIN-29
MIR577
MUP-PS21
RPL21P4
RPL23AP1
H2BU1
IGHV9-2
KRTAP1-4
LINC00472
MIR3965
MRCL3
OLFR1385

OLFR147
OLFR825
PPAR
ZFP953
ANTXR1B
C11H19ORF12
C3H1ORF198
C3H6ORF203
C6H10ORF88
CALN2
CATHL2
CELF5B
CMASB
CYP2J21
FAM192A
FAM200B
FAM78BA
FTR19
FY
GADD45GB.1
HES2.2
HMX3B
KRT95
MAPTA
MO25
OPN1MSW
PARP4P2
PCDH2AA3
PCDH2AB7
PCDH2G1
PCDH2G12
PCDH2G16
PCDH2G29
PCDH2G3
PCDH2G4
PCDH2G6
PCDH2G9
PMCHL
RPGRB
RPP20
SCRT1A
SPIN1Z
TMEM63BB
UGT2B28
ZNF1156
CFAP20DC
OLFR541
RMCS2
OLFR1371
PRL3D4
AF357426
D1ERTD705E
DEFB34
DQ267102
GPR141
IGKV2D-29
KCTD21
KRT90

MARCOL
RNA5SP366
RNA5SP63
RNA5SP74
RNU5E-4P
RNY3P2
RNY4P5
RUBIE
SCGB1B3
TOMT
TRE-TTC9-1
TRY-GTA11-1
VWA5B1
AMER3
C12ORF66
MIR7020
MIR7085
SLC6A21
OLFR311
OLFR784
SAGG
GDF7
LINC01006
DPEP3
GNRH3
BTN2A1
CIR1
ESP38
HSP90
MIR1952
MIR21-1
MIR21-2
MIR3087
MIR3584
MIR7039
MKP3
MRGPRB8
SLUG
ACSL3A
C1QL4A
CCDC80L1
CPLX4A
DCUN1D2B
DLGAP4A
DNAJC30B
FMNL2A
GRIA4A
GSDF
HOXC10A
HS6ST3B
IRX6A
KANSL1A
PAOX1
PARD3BB
PDE4BB
PLPP7A
RAB14L
RPE65B

SFXN5A
SLC44A5A
STXBP1A
TEAD1A
TIPE
TRAK1A
TRPC2B
XKR6B
ZEB1A
AER61
DNMT2
EH
IF4
PHOX2
TRAV6D-6
VOM1R3
VOM1R4
VOM1R43
VOM1R72
VOM1R75
VOM1R82
VOM1R84
VOM1R89
VOM2R72
TUBB8B
ATPSYND
MIR1246
PI3K92E
TER94
TRNM
H2BC23
IGH-V
MUP15
OLFR16
VIT-6
VMN1R32
ARF6B
CDH14
CUX2A
D1ERTD646E
D3ERTD162E
FGD4B
FTR07
GCNT4B.1
GPR155B
JAZF1A
MESPBB
MIR1934
MIR1962
MIR2139
MIR3059
MIR3067
MIR3090
MIR3110
MIR3962
MIR3966
MIR5621
MIR718

NEO1A
NMUR1B
NPAS4B
OLR392
SPINT1B
TRIP10B
VMHC
OLFR1494
OLFR152
OLFR890
SNORD111
TEX16
C79685
ZNF318
ZNF567
APOA
CHIA_1
CYP2A19
DMRT4
ESP15
ESP8
HINTW
IGKJ2
KCT2
OLFR1372-PS1
SPEER4B
ZNF514
ANKRD52
ARHGEF7-IT1
BTBD35F20
DNAH17-AS1
DNMT3AOS
EIF4G1.S
FAM240B
GOT2P1
LRRC37A5P
MIR650
OBP99A
PHXR2
PSG20
TRAV8-3
VMN1R-PS8
ZEN
ZNF29P
TRPL
COL-14
IGKV4-77
MAS
MIR658
OFA
PLK-PS1
PRDX2-PS1
PSG-PS1
RNU1A1
SOCS3A
TCRA-V11.1
TRAV6-2
TRP53-PS

ZFP783
CRYGEP
KIR3DS1
C86807
DHRS7_ANOGA
MRGPRA3
ZFP648
AOC2-PS1
CHK-1
CKN1
ESCO2-PS1
GUS
IGKV4-81
KLK1B7-PS
MIR6326
OLFR1023
OLR1459
OLR1667
OLR231
OLR443
OLR611
RGD1561206
RGD1562462
RGD1564095
SEC61G-PS1
VMN1R50
VMN1R76
VOM1R27
ZFP353-PS
ACVR1L
ADGRF3B
AFG1LA
ASF1BB
BAZ2BA
BRD3A
BUC2L
CDH7A
CDKN1CB
CLDND1A
CLK2B
COL6A4A
DAB2IPA
DDX3XA
DRLL.3
EF-1G
ENTPD5A
EPC1A
EPS8L1B
FGF18B
FOXP3A
FTR67
GJB1B
GOLGA7BA
HCCSA.1
HNRNPABA
IRGQ1
IRX7
JAK2A

JAK2B
KAZALD2
KITLGB
KLF7A
LARP4AB
LARP6B
LCTLB
LGALS13
LIPIA
LYG
MAPK12B
MEGF6B
MIER1B
MRTFAB
NF1A
NIBAN2B
NTRK3B
OR13C5
OR7D4
OVOL1A
PCSK5A
PIP5K1AA
PLAGX
PLD1A
PPP2R5EA
PPP3CCA
RAB34A
SALL3A
SP5A
SRSF7A
STAG2B
SYT7B
TAGAPA
TAOK2A
TLE2A
UBN2B
WNT7BA
ZNF1041
ZNF1059
ZNF143B
ZNF983
ZPAX2
KRTAP5-3
ARP5
FTH1P1
HEXO1
KIS
KRT18P44
KRT18P50
MTF
TDGF1P5
UNG-1
ACT1
CYP2C45
KIAA1024L
LINC02656
MIR290
MIR347

MIR3473B
OLFR591
OLFR776
OLR396
POTEB3
RPRL1
SPINK11
STK-PS2
VMN1R37
VMN1R80
VMN2R116
DICER1-AS1
EAP30
FRS3OS
LARP
MLN51
OLFR466
SBP2
ZFP934
ZNF569
ACL
ASB2B
C5H14ORF159
C7H2ORF47
CACNG5A
CBX1B
CDC25
CYP17A2
DOCK4B
FUT9A
GSL
IGHV1-20
IL8L1
KIF1AA
LRFN5B
MAGE
MCCC2L
MIR3526
MPP6B
OLFM1A
PCDH1A6
PCDH1G2
PCDH1G33
PCDH2AA1
PCDH2AB10
PCDH2AB11
PCDH2AB3
PCDH2AB5
PCDH2AB6
PCDH2AB9
PCDH2G20
PCDH2G5
PCDH2G7
PCDH2G8
PHACTR3B
PIH1D3
RAB6BA
RGD1564243

RGD1565844
S100S
SEZ6B
SLC35G2B
SLN35-A
SS2
STX5AL
TAFA5A
THSD7AB
TMPOA
TRPC5A
ZNF995
ABU-2
C21ORF58
PQN-76
PQN-91
PRX2540-1
ACSBG3
BTNL7-PS
CCPN-PS
CPG21
CSMD2OS
ECRG4B
FBXW27
IGHV1-31
IGHV4-2
IGHV5-12-4
IGKVK33-85
KCNMB4OS2
KHDC1B
KRT87
LYPD9
MIR1251
MIR135A-2
MIR1943
MIR1945
MIR1968
MIR3060
MIR3063
MIR3089
MIR3093
MIR3097
MIR344F
MIR466M
MIR5620
MIR5625
MIR6539
MIR705
MIR7116
OLFR257
PIMR115
PIMR191
PMIS2
PSG22
RGD1561648
SCGB2B20
SCGB2B7
SPINK12

TEX13C3
TRDV2-2
UBTD1B
ZFA-PS
ZNF1043
AVBD1
CYP6Z1
EPP13
HOXD3OS1
KRT18P49
OLFR1002
RHOX2A
SNORD1B
TRG-CCC6-1
TRNC
ZFP935
C25A8.1
CLDN34B
COL-182
DDIT4L2
DIRAS1A
F27E5.5
F54D12.1
GSTM.2
K07F5.7
KRTAP16-5
MAGEB1L1
MAP1SB
MIR5132
NEDD8L
NHR-169
NHR-244
OLFR688
OLFR697
OLR1151
OLR1194
OLR1196
OLR1222
OLR1345
OLR1369
OLR1456
OLR1529
OLR1582
OLR1588
OLR161
OLR1675
OLR1731
OLR223
OLR279
OLR282
OLR283
OLR313
OLR325
OLR376
OLR39
OLR476
OLR495
OLR5

OLR541
OLR577
OLR621
OLR624
OLR646
OLR669
OLR671
OLR702
OLR714
OLR727
OLR782
OLR812
OLR848
OLR916
OLR952
OXR1A
PTPRNA
RGD1562558
RGD1564513
RPH3AB
SLC6A11B
SRB-12
SRB-13
SRBC-11
SRBC-70
SRH-145
SRH-187
SRH-22
SRI-27
SRSX-13
SRX-17
SRXA-2
SRZ-74
SSP-19
STR-108
STR-145
UGT-10
VMN1R114
VMN1R207
VMN2R33
VMN2R73
VOM1R14
VOM1R95
VOM2R29
GPR33
OLFR344
RPL13P5
RSKR
RAPTOR
RGD1565588
SMAD3A
KLRA14-PS
MAGEA9B
MIR30C-2
MIR6370
MIR7018
ASAP1B
BTR32

CREB5A
CRLF1B
ENDOU2
GPD1A
GRHL2B
HIPK1A
HSPD1P7
KCNH5A
KLHL43
LINC00837
MAGI2B
MIR320C2
MIR5087
MIR527
MIR548N
MIR719
NLGN2B
NRBF2B
NRBP2B
PANDAR
RGPD2
RPLP0P1
SNORD7
TP53COR1
TSPAN9B
ACOX57D-P
AMY-P
C1GALTA
CACNG1B
CASP8L1
CBT
CBX6B
CHTOPB
CNN1A
CP19
CPR100A
CPR49AB
CPR49AD
CYP11A2
CYP317A1
CYP6A20
CYP6A9
CYP6T3
CYSLTR2B
D17ERTD165E
D9ERTD115E
DMGLUT
DRD4A
DRSL1
DSX
E
ECT3
ELOF
FC18A08
FJ
FUSL
GIIISPLA2
GS2

HECW1B
IGKJ1
IM
IN
INVADOLYSIN
IR31A
IR52B
KARL
KOP
LIN-28
LRT
MACON1
MAL-B1
MED19A
MIR1301
MMP17B
MOB1BA
MOL
MTERF1B
NACHRBETA3
NPC2D
NXPH2B
OBP56E
OBST-B
OLFCK2
OR45B
OSI8
OUT
PGRP-LB
PGRP-SB1
PK1-R
RDGB
RIC3B
RNP4F
RNU2
RPL5A
RUMI
SC
SCOCA
SEPT7B
SER8
SMVT
SNMP1
SRO
TLCD5A
TMEM169B
TMEM182A
TMEM264
TOBI
TOR1
TRK
TSPAN2A
UGT302E1
UGT37C2
UGT37E1
ZFP599
ZMP:0000001200
ZNF687B

ADH5P4
ATP6V0CP1
BMM
DPP6A
EIF4G
FAM83A-AS1
FER1L6-AS2
FTH1P19
GGACT.3
KCTD9P2
KIRREL3-AS3
LINC00315
LRRC53
LY49S3
METHIG1
MIR105-1
OR2T33
OR7E19P
RPL10P12
RPL27P2
SKINT1
VN1R10P
VOM1R45
VOM1R71
VOM1R86
VOM2R41
VOM2R54
VOM2R7
ZFP979
IL11B
INR
LINE
RN5S
TRNP
CYP3A29
EGG-4
OLR37
PRO2268
RAB1C
ACOT18
ADGRG11
ADGRL2A
CCNT2B
CHCHD4B
CNOT6B
CREB3L3L
CREBBPB
CYP3A24
DIABLOB
EIF4BA
ENC3
FRMD4BA
GNA15.1
HER11
HER5
ILDR1B
MIR1A-1
MIR6216

MORC3B
NME2B.1
NUPR1A
PHACTR4A
PKDCCB
PPP1CAA
RBPMS2A
SALL3B
SCARNA23
SETB
SH3BP5LA
SHISA2A
SLBP2
SMARCA4A
SNORA58
SP140L
SSH2A
SZL
TARDBPA
TMEM51A
TRPC4APA
ULK1A
WASLA
YY1A
ZFPM2B
143B1
ATOH1B
BEAT-VB
BETACOP
CCS1
DELTACOP
FSCN2B
GCL
HMGN2-PS1
MLRN
NG
OLR1132
OLR1160
OLR1223
OLR126
OLR1279
OLR1525
OLR156
OLR1565
OLR1621
OLR206
OLR208
OLR375
OLR453
OLR659
OLR742
OLR832
PRDX-2
REL1
RGD1562036
RGD1564571
RL23A
RPS3.L

SAGE
SAKS1
SAP47
SRCR12
SYX5
TACD2
TUBB3-PS1
VEGFR-1
VOM1R8
VOM1R88
ABHD10B
ADAM19B
ADPGK2
ASF1
ATP6AP1LB
ATPV0E2
BMI1B
CACNA1HB
CCDC106B
CCNB1L
CCNT2A
CNOT4B
COL12A1B
CRYGM2D3
CRYGM2D7
D14ERTD725E
DRD6B
EPN3B
FAM49BA
FAM83FA
FHOD3A
GLRBB
GRIA2A
H2-T18
KAZNA
KDM5BB
LIFRA
LNX2B
LRIT3B
MESPAA
MIR1930
MIR3103
MIR697
MMP20A
OLR1384
OLR48
OLR724
OLR95
OPN4B
PIMR75
PTPN2B
RARGB
SDHDB
SEC14L8
SLC17A9B
SLC2A3B
SLC7A8B
ST6GALNAC1.2

UACAB
ZFP862
ZNF613
ZNF740A
CRMP
CYP51A1P2
HMGN2L6
OLFR1229
OLFR1387
OLR1423
VMN1R214
ACE-1
ALPHA-EST7
CEP112IT
CSTA2
D4ERTD58E
D5ERTD615E
IGH-VS107
IGKV1-35
IGKV4-50
IGKV4-79
LAP
LCE1J
MCCC1OS
MIR129B
MIR344C
MIR6373
MIR6412
MIR6904
MIR6909
MIR6950
MIR6952
MIR6976
MIR7678
MIR883B
NRG3OS
OLFR1171-PS1
OLFR664
OLFR75-PS1
OLFR9
PANCT2
PITPNM2OS1
PRAMEL13OS
S100A11-PS
SDHB-1
SPAG11BL
SULT2A-PS2
TACO1OS
TGIF2LX2
TMA7-PS
TRAV16D-DV11
TRAV6D-5
TRIM30C
VMN1R151
VMN1R45
VMN2R3
VMN2R-PS111
ZFP652OS

ABTB2A
ATP12A-LIKE
ATP5F1AW
AVBD7
AVDL
BCRP2
BTBD3A
C22H8ORF4
CNTNAP2A
CPR8
CRABP
EEF1B2P5
G12
GRIA1A
GRID1B
HOX3
HOXC3
IF2
KCNJ11L
KCNQ2A
LRTM2B
MEIS
MYL9A
MYT1LA
NOL4LA
NOS1APB
NPHS1OS
NPTNB
OR7H2P
PCDH1A
PCDH1G18
PCDH1G26
PCDH2AB1
PCDH2AB8
PITX
RAB42A
RCVRN.1
RGD1311300
RGD1559588
RNF145A
RPR
SHISA9B
SPRB
TECRA
TH1L
TMEFF2A
TMSB2
TPCN3
XA-1
ZNF729
BIN2A
BMP7B
CAB39L1
CICA
DENND2DA
IGKV12-98
PLEKHN1
SHANK3A

SPINK10
STXBP5A
TRD
ALDOB.L
BCAR3-AS1
MAGMAS-PS1
METTL21E
RAB35B
SPE-4
SWM-1
BIN2P1
EGL-9
LCAL1
LINC00527
LOK
MMP1B
OLFR62
OR1E3
PAXBP1-AS1
TCAF3
MIR195A
MSMP
BTNL5-PS
CYP2C2
DEFA-PS1
DPY-4
DPY-7
DPY-9
HMOX1B
IF
IGKV8-30
LGALS3BP.3
MOB1BB
MUP8
OLFR1442
OLFR78
SMTLB
AVBD6
BCAR4
LDH
MIR3086
MIR3109
MIR5107
MIR666
MIR875
MMP11A
PRL5A1
RDH3
TB
THP2
AATKB
ACTG1P20
ADMB
ALDH3A2A
ANXA3A
ARHGAP12A
ATF7A
B3GNT7L

BCL2B
CCKA
CHMP5A
CHRNB3A
CLOCKB
CORO1CB
DBNLA
DDHD1A
DHRS13B.1
DNAJC16L
DNAJC5GB
EPN3A
FAM20CA
FAM219AB
FIBINB
FMNL2B
FNDC4B
FNDC7A
FTR20
GABPB2B
GLIPR1B
GPR137BB
GRM8A
HIVEP2B
HMGXB4A
KCNH4B
KDELR2A
MAFAA
MAN1B1B
MCOLN1B
MERTKA
MSL2B
NFYAL
NLGN3B
NT5C2B
OGDHB
PPP1R3AA
PRSS60.2
RAB33BA
RPH3AA
SEPH
SLC2A13B
SLC35A3A
SLC35G2A
SLC44A1A
SMX5
SNX8A
STOX2B
SVOPA
TEAD3B
TMEM30AA
UNM_HU7910
WAPLA
ZMP:0000000606
ZMP:0000000711
ABHD11OS
ACKR3B
ART2A

CD44A
CHRM2A
DDX3XB
EHMT1A
FOXD5
GAGE4
GRINAB
HNRNPUB
INO80DB
ITPK1A
NIBAN2A
OLFR1087
OLFR1102
OLFR1135
OLFR1157
OLFR1242
OLFR129
OLFR13
OLFR1360
OLFR141
OLFR1512
OLFR237
OLFR560
OLFR623
OLFR739
OLFR741
OLFR746
OLFR845
OLFR983
OLR421
PAFAH1B1B
PHO
POU5F3
PRL8A1
PSME2B
RBM4.2
RGD1559859
RGD1561185
RGD1562660
RGD1563747
RGD1565590
RHOAA
RPL36A-PS3
RXFP2A
SEMA6DL
SETDB1A
SLC25A55A
SMARCC1A
SMN
SRSF1B
STARD14
SUMO3A
SUMO3B
TIAF2
TMPRSS4A
TMPRSS4B
VMN1R44
AFR2

ALBFM1
BTBD35F1
C80012
CRYZL2
CYP3A71-PS
CYP51A1-PS1
D7ERTD413E
D9ERTD338E
DAF-18
DNAJA1-PS
FRG2F1
GCNT6
GGNBP2OS
HSPD1-PS19
HSPD1-PS8
HSPE1-PS2
IFI207
IGKV4-62
IMPDH2-PS
LY6G2
LYRIC
MIR340-2
MIR3577
MIR434-2
MIR466F-3
MIR5124A
MPH1
MUP-PS1
MUP-PS11
MUP-PS13
MUP-PS15
MUP-PS2
MUP-PS6
MUP-PS7
MUP-PS8
MUP-PS9
NDUFS5-PS1
NUPR1L1
OLFR446
OLFR449
OLFR64
OLFR645
PROX1OS
PSME1-PS1
RGD1559499
RGD1560234
RGD1560795
RGD1561787
RGD1562140
RGD1562387
RGD1563322
RGD1564292
RGD1564585
RGD1564981
RGD1565361
RGD1565664
RGD1565988
RGD9310068

SAA-PS
STAU
TMEM250-PS
UGT1A4-PS
B3GNT10
CYP2AB4
CYP2J22
CYP2J24P
OLR25
TRAV7-4
CACNA1SB
D2ERTD295E
DEFB17
HIF-1
KCNQ5A
KLRH1
LY49I7
LY49I9
MRGPRB13
OLFR125
PFC
RGD1309998
SU(R)
VIPR1B
VOM2R22
VOM2R75
VOM2R80
A2MP1
ATP6V1G2-DDX39B
BOULE
C1QTNF3-AMACR
C9ORF84
CTSSL
DCDC5
DENND5B-AS1
DRP1
EIF2S3B
GATD3B
HSTRPA
IGLV3-25
LCA10
LINC00559
LINC01719
LINC01844
MIR1292
MIR3141
MIR3162
MIR3200
MIR376A1
MIR4280
MIR4505
MIR4667
MIR4669
MIR4701
MIR5106
MIR519D
MIR520C
MIR6753

MIR6820
MIR6865
MIR7843
MUC3B
NHR-119
NHR-171
NHR-194
NHR-210
NHR-246
NHR-261
NPIPA7
OLFR1038-PS
OLFR371
OLR1323
PIP5K1BA
RN7SL3
RNA5-8SN1
RNA5-8SN2
RNA5-8SN3
RNU5A-1
RNU6-807P
RNU6-9
RPSAP15
RSL24D1P3
SERTM2
SLX1B-SULT1A4
SMG1P6
SNORA10B
SNORA17A
SNORA31B
SNORA35B
SNORD115-32
SNORD13P2
TAS2R126
UNC-55
V165-D-J-C MU
VCAN-AS1
VLC
VOM2R1
ADAM24
ASB14B
ASB5A
C1H21ORF91
CCDC88AA
CELF5A
CRISPLD1B
CRYGM6
CRYGS2
GABRB4
GRIA2B
HER2
HES2.1
IGSF21A
MTNR1BB
NOG3
OTX5
PARP6A
PCDH1G32

PCDH1G9
PIMR173
S100T
SLC6A6A
TNK2A
B0334.6
BATH-36
C02D5.4
C13A2.12
C21ORF62-AS1
C24H12.2
C39F7.5
C43D7.7
C47E8.3
C49F5.6
CNC-11
CPR-4
DCPP2
EEF1E1-BLOC1S5
F07C6.6
F17B5.8
F17C11.15
F39B2.14
F43D9.8
F46C3.7
F53G12.9
FBXA-95
GMD-2
HLA-DPA3
K07C5.10
LINC01193
LINC-72
LIPL-3
M110.8
M162.9
MATH-43
NSPB-8
RAET1E-AS1
VMN1R30
W06H8.12
Y24D9A.9
Y37H2A.18
Y37H2A.7
Y47G7B.2
Y51B9A.8
Y54G2A.48
Y57G11A.5
Y7A5A.10
ZK637.18
ABU-3
ABU-5
CYP-13A6
FIT1
GLYP
IGKV13-84
IGKV7-33
ILP5
MIR1B

MIR218-2
MIR569
MIR639
MIR645
MIR677
MSTNA
OLR1350
OLR197
PQN-78
PRX6005
SYNJ2BP-COX16
ACD-1
ACDH-6
ACKR4A
ACLY-1
ACPL2
ACSL2
AEP1
AKT2L
ALDH9A1A.2
ALH-6
AMPD3A
APOOB
ARGLU1B
B0025.5
B0238.18
B0238.9
B0285.T1
B2ML
BE0003N10.3
BED-3
BGNT-1.8
BON
C01B10.11
C01G6.3
C02B4.8
C02D4.15
C02F4.4
C04F1.1
C06B8.T2
C06E7.4
C08A9.10
C08D8.3
C08G5.7
C13C12.2
C14B9.T2
C16C8.21
C16D9.12
C17B7.12
C17E7.9
C27A7.2
C28H8.5
C29E4.14
C30G7.4
C33F10.17
C33F10.21
C33H5.2
C35B1.4

C35B1.8
C36C5.5
C44B7.5
C50D2.1
C52A10.4
C54D10.16
C54D10.3
C79461
CACNA1FB
CACNB4B
CCDC85AL
CCN1L1
CDK5R1B
CELSR1B
CEST-35.1
CHE-14
CLIC
COL-111
COL-131
COL-135
COL-152
COL-65
CPG2
CPT-1
CRYGM2D1
CRYGM2D21
CRYGM2D4
CYP-33B1
D1014.6
D1014.7
DAF-28
DDO-3
DHARMA
DHS-18
DHS-20
DKK3B
DNAJB9B
DPF-1
E03E2.T2
E03H4.4
ECH-8
ENDU-2
EXC-4
EXP-2
F01D5.6
F01G10.10
F07C6.5
F08G5.14
F10D2.10
F10D2.15
F15E6.10
F16A11.9
F17B5.1
F21C10.11
F22B7.T1
F22F7.3
F22H10.9
F22H10.T1

F23A7.4
F28D1.13
F30A10.14
F32H2.11
F35E8.18
F35F10.5
F36H12.10
F36H12.5
F38E11.11
F41C3.2
F41G3.10
F42D1.6
F43G9.15
F43G9.18
F44A6.5
F45D11.15
F45D3.3
F46C8.8
F49C12.1
F49C12.2
F49C12.3
F49C12.4
F49C12.5
F49E12.12
F52F10.2
F53B1.2
F54D10.8
F54F7.3
F56F10.10
F56F4.10
F57C2.4
F58A6.1
F59A6.11
F59D8.3
FAM117BA
FBXW22
FHDC3
FIP-2
FIPR-10
FIPR-8
FKH-9
FMO-4
GLB-1
GLI2A
GLY-1
GNB5B
GRH-1
H12I13.6
H23N18.5
H36N01.3
IGHV1-66
IGKV2-116
IGKV4-51
IGKV4-63
IGKV8-16
IGKV8-34
IIGP1B
IMP-3

INS-30
K01A2.5
K01A6.7
K02E11.10
K06A4.13
K06C4.20
K07A1.6
K08C7.4
K10C2.8
K11H3.T2
KIFL
KLRA20
LAYNB
LCTLA
LFOR-2
LGALS8A
LIN-12
LIPS-10
LIPS-15
LIPS-3
LONP-2
MAM-2
MATH-3
METRNLB
MF-CPA
MIR130A-1
MIR3472
MIR3576
MIR461
MIR606
MIR732
MORF4L1-PS1
MPST-6
MSA-1
MSP-58
MSP-71
MUG4-PS
NAB1A
NAS-36
NAS-7
NEP-16
NEP-23
NHR-161
NHR-205
NHR-234
NHR-267
NHR-62
NITR8
NPR-1
NSPB-2
NSPC-1
NSPC-16
NSPC-19
NSPC-2
NSPC-4
NSPC-6
NSPC-7
NSTP-8

OAC-10
OAC-28
OAC-46
OCR-1
OLFR1269
OLFR1336
OLFR223
OLFR684
OLR1218
OLR1219
OLR1220
OLR1238
OLR1239
OLR1415
OLR1596
OLR20
OLR567
OR119-2
OXSR1B
PALS-23
PFKPA
PGAM1-PS2
PHO-9
PIMR62
PPS-1
PQM-1
PQN-74
PRDM16OS
PRX-11
PST-1
PTC-3
PTR-11
PTR-15
PTR-16
PTR-6
PUD-2.2
PXNA
R02E4.3
R03G8.6
R04B3.3
R05A10.4
R07E5.4
R09F10.17
R11F4.1
R74.2
RAPGEF5B
RBM24B
RDH14B
RHOX7
RNCS-1
RPL23AP37
RPZ5
RRN-1.1
RRN-1.2
RRN-2.1
RSF1OS2
SCGB2B26
SINUP

SLC2A15A
SLC37A4B
SLC47A4
SMC1AL
SQT-2
SRPR-2.2
SRU-40
SRXA-3
SSP-33
ST3GAL3B
ST8SIA7.1
SWSN-7
T01B4.4
T01D1.3
T01D1.4
T04A8.13
T05B4.14
T06E4.12
T06H11.T1
T07G12.3
T08B2.12
T16A9.6
T16G1.13
T17H7.7
T19B10.2
T19B10.5
T19C4.17
T19C4.T1
T24A11.5
T24D3.2
T27A10.8
TAG-297
TAO
TLK1A
TPST1L
UBE2AL
UGT-35
ULE-1
VMN1R53
VMN2R43
VMN2R71
VMN2R84
VMO-1
VT23B5.1
W01C9.6
W02D9.7
W08D2.9
Y105E8A.42
Y11D7A.9
Y18H1A.T1
Y25C1A.13
Y26D4A.24
Y34B4A.10
Y38A10A.2
Y38C1AB.1
Y38F2AL.11
Y40C5A.19
Y40C5A.22

Y42G9A.8
Y43F4A.6
Y48A6B.9
Y51H4A.T2
Y52B11B.1
Y53G8B.2
Y54E2A.7
Y57G11C.1130
Y58A7A.1
Y59E9AL.2
Y62E10A.19
Y62H9A.3
Y65B4BL.6
Y69A2AR.44
Y71F9AM.8
Y71G12B.18
Y73B3A.24
Y73B3A.28
Y73C8B.1
Y87G2A.2
Y95B8A.16
Y9C9A.16
ZC21.3
ZC373.2
ZC455.14
ZDHHC20A
ZFP974
ZK1025.2
ZK1025.4
ZK1025.5
ZK1025.8
ZK1067.10
ZK546.3
ZK550.6
ZK792.4
ZK813.3
ZK813.7
ZK897.2
ZK897.4
ZMP-6
ZP2.1
APOL7E
BVHT
CLEC6A
CLN9
DEFB27
H2
H2-M1
HIST1H2A11
NDUFA4A
SAA2-SAA4
42SP43
42SP50.L
ACSS2.1
ADA.2
ADRA2DA
ALDH3A2B
ANAPC13.1

ANKRD2.S
ARCN1A
ARHGAP11A.2
ARX.L
ARX.S
ASB12.2
ASP
ASTL2C
ATG4A.S
ATOH7.S
ATP1A1.S
B3GNT5.S
B4GALT1.2
BAF-L
BIRC5L
BIX1.1
BMP2.L
BMP7.2
C24H11.1
C6.2.L
CAMK2G1
CAR1_PREDICTED
CARM1P1
CAT.1
CAT.2
CCN3.L
CCNB1.2
CCND2.L
CCNDX
CDCA5.S
CELF3.L
CFOS-A
CLDN15.1
CLDN6.1
CLDN6.1.S
CLTRN.L
CNDP2.L
COL25A1.2
COMT.2
COX5B.2
CPEB1.L
CPLX2L
CRADD.1
CRHR1.1
CRHR1.2.S
CRYBB2.L
CTNNB1.L
CYP2C8.2
CYP4B1.2
DCK.1
DCK.2.L
DES.1
DLG1L
DMRT1.S
DMRT2A
DNAJA4.1
DNAJA4.2
DPPA3L1

DPY-8
DUSP11.2
DYNLL1.S
DYRK1A.2
DZIP1-PS1
EDNRB2
EEF1A1O
EEF2.1
EEF2.2
EFNA5B
ELN2
EN2.S
ENPP7-L
F35C11.2
F35C11.3
F47B3.1
F49E11.7
F54H5.3
FAAH.2
FABP2.S
FCN3-A
FOXA4
FOXC1-A
FOXD4L1.1
FOXF1.L
FOXI2.S
FOXJ1.2
FOXJ1.L
FRZB2
FSCN1B
FUCOLECTIN.S
FZD8.S
GABPB1.S
GATA4.L
GATA4.S
GDNFA
GNA11A
GNGT2.1
GNRHR2/NMI
GPHNB
GRHPR.1
GRHPR.2
GRIK5-LIKE.1.L
GST-37
GTF2A1.S
GTF2E1.2
H1-6.L
H2AC1.S
HAL.1
HBA3
HBA-L2
HBA-L5
HCST.L
HES1.S
HES6.2
HES7.1
HIATL3
HMBS.2.S

HOXB9A
HOXD10A
HOXD11A
HSBP1B
HSF2.2
HSPH1.L
IGF2BP3.L
IGHV4-31
IGLV5-48
INKA1.S
INVS.S
IPO5.S
IRF6.2
KIT.L
KLK1C8
KRT12.4
KRT61
KRT70
LCT.1.L
LDLR.S
LGALS4.1.L
LMNB3
LP1
LPAR1.L
MACROH2A2.L
MAP3K12.S
MAX.S
MCHR1.1
MCHR1.2
MCM6.2.S
MHC1B2
MMP9.1.S
MOB2.1
MOGAT1.L
MSMB.3
MSTN.1
NEUROG2.S
NHR-265
NIF
NKX3-3
NOT
NOT.S
NR5A2.S
OCM4.2
OLFR215
OLIG4
OLR1006
OLR1012
OLR1024
OLR1096
OLR1213
OLR1233
OLR1246
OLR1302
OLR1315
OLR1409
OLR1448
OLR1492

OLR1519
OLR1619
OLR1653
OLR1689
OLR259
OLR470
OLR551
OLR597
OLR601
OLR637
OLR660
OLR668
OLR819
OLR943
OMP.S
OR111-5
OR115-10
OR13C2
PAR3
PC.2
PDHA1.S
PDIA6.L
PED
PEO1
PGQ
PICALML
PITPNB.2
PKDCC.2
POLR1D.2
POLR2L.1
POU3F2.S
POU4F1.2
POU5F3.3
PPP1R3C.2
PRL.1
RAB11B.1
RAB11B.2
RAB11FIP4L
RACGAP1.2
RAD51.L
RAMAC.L
RAS-DVA1
RAX.S
RBBP4.L
RCC1.S
RCVRN.2
RDH7.2
RFLCII
RGD1306227
RGD1559752
RGD1560225
RGD1561079
RGD1562134
RGD1563060
RGD1563578
RGD1563815
RGD1564320
RGD1564599

RGD1564645
RGD1565299
RGD1565323
RGD1565534
RGD1566265
RHBG.L
RPS28P9
RRM2.1
RRM2.2
RTRAF.S
S100A10.L
SASS6.2
SCN12AA
6-Sep
SHISA1.2
SIRT3.2
SIX3.L
SLC16A3.S
SLC22A15.2
SLC35A3.1
SLC4A10B
SLC5A1.1
SLC66A2.2
SLC6A14.2.L
SNRNP40.L
SOX11.S
SOX17A
SPDYC.L
SSB.L
SST.2
ST3GAL2.1
ST3GAL2.2
STXBP6L
SUMO2.L
SURF4.2
SYN2.L
T10G3.4
T2R19
T2R29
T2R53
TAAR7H
TAMALIN.L
TAS2R124
TBC1D20.1
TBC1D24.1
TDGF1.3
TECTA.2.S
THDL18
TMEM150B.L
TMEM178.2
TOP1.2
TRG.L
TRHD.S
TRH.L
TRIM3B
TSKU.S
TTLL13P
TUBB2

UGDHL
UGDH.S
UGT-34
UMOD.3
VAX2.L
VEGT
VELO1
VENTX3.1
VOM1R36
VOM1R6
VOM2R19
VOM2R24
VOM2R32
VOM2R36
VOM2R-PS138
VSNL1B
WARS1.S
WNT11B.L
XNF7
YBX2.L
ZBTB8A.1
ZFP18
ZFP36L2.1
ZFP36L2.2
ZFP36L2.2.L
ZK1248.4
ZK809.1
ZNF238.2
ZNF706L
ZNF750.L
ZPAX
BOKB
CHD4A
FTR82
H3F3D
HMGN6
HMGN7
ILF3B
LYE
MED19B
MTP
OLR961-PS
RPLP2L
SETD1BA
STMN2A
GNL3LP1
IFI211
N-TUTCA2
ROCK2B
SELENOP1
SNORD8
SPE-6
TRY-5
WEE-1.3
ABCF2B
ACTR3BP2
ADGRE5B.2
ADGRF8

AP3B1A
APOOA
AQP-11
ARAP1A
ARHGEF25A
ARHGEF28A
ASMT2
ASNSP1
ASNSP4
ATF-4
ATG10-AS1
ATP6V1E1A
AURKAP1
AVPR2AB
BPIFCL
C1QTNF9-AS1
C3B.1
CABP2B
CACNA1C-AS2
CACNG2A
CASC18
CASC22
CBLN10
CBLN14
CBLN17
CBX3B
CCL20A.3
CCL39.2
CCN1L2
CEACAMP3
CGNB
CHIA.6
CHMP4BA
CHMP4BB
CKLF-CMTM1
CLDN34
CLEC-198
CNGA2B
COA3A
COL14A1A
COX8
CRFB4
CT47A6
CXL34B.11
CYP4F9P
DACOR1
DANT2
DEFA8P
DENND3B
DICP1.16
DNAJA2A
DNAJB12A
E1
EFCAB13-DT
EGLN1A
ENC2
ESYT1A
EVI5A

EXOC3L2B
F55G1.9
FAM219AA
FGF12-AS1
FHDC4
FIBPA
FIP1L1B
FLI1RS
FTH1P27
FTHL28
FTHL30
FTHL31
FUT9B
FZD9A
GLCEB
GLIS2B
GPA33B
GPC5-AS1
GPC5C
GPR22A
GPR35.1
GRA
GRHL2A
GRM1B
GTPBP2B
HDAC7A
HIS-66
HIS-8
HRASB
HS3ST3B1B
HSP30E.L
IFFO2A
IFIT16
IGHV1-46
IPMKA
IQCM
ITPKCA
KALRNB
KAT7A
KIRREL3L
LAMP1A
LDLRAP1B
LGALS8-AS1
LINC00290
LINC00417
LINC00463
LINC00550
LINC00551
LINC00659
LINC00683
LINC00857
LINC00911
LINC01010
LINC01048
LINC01120
LINC01151
LINC01204
LINC01277

LINC01282
LINC01333
LINC01348
LINC01386
LINC01445
LINC01493
LINC01497
LINC01501
LINC01690
LINC01702
LINC01727
LINC01789
LINC01931
LINC02036
LINC02086
LINC02104
LINC02223
LINC02228
LINC02302
LINC02361
LINC02369
LINC02453
LINC02519
LINC02630
LL22NC01-81G9.3
LNCNEF
LOTGIDRAFT_154656
LRRC38A
LY6M7
MAST3A
MED-1
MHC1ZEA
MIF4GDB
MIR1258
MIR3125
MIR3126
MIR3151
MIR3179-3
MIR3919
MIR3935
MIR4518
MIR4644
MIR4646
MIR466E
MIR4694
MIR4728
MIR4747
MIR4763
MIR4764
MIR548AH
MIR6132
MIR6739
MIR6763
MIR6801
MIR6813
MIR6824
MIR6826
MIR6833

MIR6894
MIR7114
MKK6C
MMP25B
MUC13A
MUC5.1
MXF
MYO1EA
MYO1HB
MYO9AB
NANOGP1
NEGR1-IT1
NLRC3L
NLRP16
NPIPA5
NSPA-3
NT5C1BB
NUO-6
2-Oct
ODC1-DT
OGNA
OLFM2B
ONECUTL
OR10AC1
OR11H7
OR14A2
PANX1B
PARGA
PARP6B
PCBD-1
PDGFBA
PIMR214
PKN1A
PLPPR5A
PNPLA7B
PONZR1
PPP1R14BB
PPP1R14BL
PRDM1B
PRKCAA
PRR5A
PSG8-AS1
PTP4A2B
RAB25A
RANBP3A
RBB4L
RBM8A2
RHOUA
RIMKLBP1
RNA5SP237
RNA5SP265
RNA5SP269
RNA5SP439
RNA5SP510
RNU7-47P
RNU7-63P
RNY4P3
ROCR

SAMD1A
SDK2A
SEMA4GA
SH3GL2A
SIM1B
SLC16A5B
SLC16A6A
SLC41A2A
SORBS2B
SOX4B
SPARTB
SPINK2.1
SSUH2.1
STYK1B
SWAP70A
TBC1D10AA
TCIMB
TCNBB
TDRD7A
TMC2A
TMEM168A
TMEM212-IT1
TMEM54B
TMEM92-AS1
TMLHE-AS1
TMPRSS11GP
TOPORSB
TPBGA
TRA-CGC3-1
TRAF2B
TRAV26-2
TRBV28
TRBV3-1
TRPC6A
TSSC2
TTTY17A
TTTY17C
TUBB2A-PS2
UGT2A5
UGT-58
UGT5B1
UNM_SA911
VHA-16
VWA8-AS1
WFIKKN2A
WIPF1B
YWHAE2
ZBTB20-AS2
ZDHHC12A
ZDHHC3A
ZDHHC8A
ZNF1179
ZNF362A
ZNF576.1
ZNF630-AS1
ZNF649-AS1
ZNF977
ZSCAN5DP

CLDN34C1
FPR-RS4
H2AB3
LRRC77P
MIR2861
MIR3075
MIR341
MIR741
OBP1A
OLFR1022
OLFR1367
OLFR659
OLFR763
OR2F2
SCOSPONDIN
VMN2R10
AQP3B
CACYBPP2
CIBAR1P2
CT45A5
DNMT1A
FABP5P9
FAM25E
GOLGA8K
KIR2DS1
KIR2DS5
LINC00410
MAS1LP1
MIR320C1
MIR580
MIR681
PPIAP26
RPS15AP10
SSXB10
TFDP3
ACVR1BB
ADIPOQA
ANO11
ATTB
BAIAP2L2B
CCNL1B
CEST-4
CLEC-52
CNKSR2B
CPLX3A
CWO
CYP11C1
CYP4AA1
CYP4AE1
CYP4D14
CYP4P2
DAB1A
DAB2IPB
DNM3A
DPY-13
DPY-5
ERFL3
F11R.1

FAM96A
GLCEA
GMPPAA
GRID2IPA
HMBSA
HS3ST3L
HTR2AB
HUG
IGHV1-54
IOTATRY
KANSL1B
KAT5A
LECTIN-33A
LHFPL4B
LSD-1
M
MINPP1A
MYO9AA
NDFIP1L
NFYBA
NINAD
NLGN4XA
NPM2A
NPTX2B
NYAP2A
OBST-A
ODC2
OLFR1161
OLFR71
PALD1B
PANX1A
PARVAA
PCDH15A
PGLYRP6
PHKA1B
PITPNBL
PRMT8B
RC3H1A
R-L
SBK3
SCAF4A
SDR16C5A
SER6
SMARCD3B
SPOPLA
SUG
TBC1D3
TMED11
TMEM144B
TMEM222B
TMEM238B
TNKSB
TOLL-9
TPD52L2B
TSPAN5A
UBE2NB
UGT35C1
VERM

VVL
ABHD17AP4
ALAS
C20ORF181
C3ORF56
CASP16
COL17A1B
COX6B
CTAGE7P
CYP2A10
CYP2J1
DLSTP1
DMRTC1C1
DPPA3P2
FAM47A
FBXW13
GAGE12B
GSN-AS1
KRTAP20-4
LINC01620
NFE4
OR1K1
OR2T34
OR3A4P
OR6Q1
OR7E125P
PRAMEF19
RLA-DMB
RLA-DRB1
RPS2P35
TESL
VCSA2
VOM2R73
CSN1S2A
HIF1AA
MIR5099
MIR5121
MMP13B
MMP17A
RPL27AP6
SNORD45C
SYNA
GJC4B
HCFC1A
OLFR726
PHF20B
RGD735065
SLC24A4A
CPG1
FAM213A
HIP14
KAT3
MIR1193
MIR1298
NLRP4C
OLFR1120
OLFR1158
OLFR1406

OLFR1501
OLFR177
OLFR248
OLFR270
OLFR305
OLFR39
OLFR643
OLFR734
OLR1644
OLR540
RGD1306502
RGD1560324
RGD1561231
RGD1562143
RGD1563581
RT1-O1
SLX
SPETEX-2H
TPT1P8
VMN1R29
ACLYB
ANKRD30BP3
ATP5MEB
ATP7
ATRIP-TREX1
BCL2L1-PS1
BHMT-PS1
BNIP4
BPY2C
C10ORF71-AS1
C20ORF202
C5ORF17
C80406
CA15P1
CADM2-AS2
CAND1.11
CDH18-AS1
CDY1B
CPEB2-DT
CRAT37
CRYGM2D12
CRYGM2D15
CRYGM2D16
CRYGM2D19
CRYGM2D5
CRYGM2D8
CRYGM2D9
CTR1A
CXXC4-AS1
CYCSP6
D15ERTD509E
DAAM2-AS1
DAD1P1
DEFB105A
DEFB106A
DEFB107A
DEFB107B
DNASE1L1L

DPPA2P3
DUSP22A
DUX3
DUX4L2
DUX4L3
DUX4L6
DUX4L7
EARS2L1
EDDM13
EXORH
FABP7B
FAM30C
FMO13
GACAT1
GCSAML-AS1
GS1-279B7.1
GUCY2GP
HIPK1-AS1
HMX4
HSD3BP5
HYAL6P
IGHV5-78
KCNK1A
KCNK5B
KDM4E
KRT8P25
LINC00254
LINC00307
LINC00330
LINC00457
LINC00534
LINC00575
LINC00587
LINC00958
LINC00972
LINC00989
LINC00993
LINC01019
LINC01029
LINC01037
LINC01039
LINC01152
LINC01231
LINC01307
LINC01324
LINC01477
LINC01483
LINC01484
LINC01492
LINC01525
LINC01531
LINC01553
LINC01568
LINC01579
LINC01581
LINC01602
LINC01752
LINC01753

LINC01878
LINC01883
LINC01894
LINC01915
LINC01947
LINC02042
LINC02052
LINC02098
LINC02107
LINC02162
LINC02192
LINC02198
LINC02212
LINC02236
LINC02292
LINC02314
LINC02346
LINC02376
LINC02378
LINC02424
LINC02450
LINC02476
LINC02505
LINC02523
LINC02533
LINC02567
LINC02568
LINC02588
LINC02618
LINC02627
LINC02645
LMO7B
LT1
MHC1UDA
MIR1286
MIR29C-2
MIR3545
MIR3614
MIR3681HG
MIR4258
MIR4283-1
MIR4302
MIR450B-2
MIR466B-3
MIR466B-4
MIR466C
MIR4686
MIR548H2
MIR548XHG
MIR5572
MIR598-2
MIR6515
MIR6771
MIR6825
MIR6838
MIR6864
MIR7667
MIR7705

MIR7974
MMP24-AS1-EDEM2
MOXD2P
MPP5A
MTA
MYCNUT
NCBP2L
NRIP1B
NTN1B
OLR1102
OLR1138
OLR1162
OLR119
OLR1329
OLR1338
OLR1378
OLR1388
OLR1431
OLR1436
OLR1457
OLR1470
OLR1507
OLR1536
OLR1539
OLR1555
OLR1607
OLR1630
OLR165
OLR1660
OLR196
OLR209
OLR210
OLR227
OLR286
OLR305
OLR310
OLR311
OLR401
OLR417
OLR420
OLR428
OLR456
OLR463
OLR491
OLR560
OLR641
OLR710
OLR745
OLR838
OLR85
OLR907
OLR92
OLR98
OR4F17
OR4F4
OR4K14
OR51A4
PCDHB17P

PGAM1B
PIAS
PICALMA
PISRT1
PLBD1-AS1
PNMA6E
PPP1R13BA
PRAMEF2
PRDM2B
PRF1.6
PTENP1-AS
RAB6C-AS1
RASAL2-AS1
RBMY1B
RBMY3AP
RGD1561106
RGD1561410
RL19
RN7SL396P
RPSA-PS10
SLC34A1A
SNORA92
SNORD115-34
SNORD121A
SNORD90
SNORD96B
SPCS2-PS
ST7-OT3
SUCLG2-AS1
TAAR3P
TAC3B
TAS2R104
TMBIM7P
TMEM72-AS1
TNFSF10L3
TNRC18P2
TTTY1
TTTY1B
TTTY6
TTTY9A
TTTY9B
UCHL1-AS1
USP12-AS1
USP17L30
VAV3-AS1
VHAPPA1-2
VIML
VOM1R21
VOM1R31
VOM1R99
VOM2R28
WG
XACT
ZBTB46-AS1
ZNF426-DT
ZNF571-AS1
ZNRF2P2
ZRANB2-AS1

CYP2B5
DNAH7A
G4
NG3
OLFR221
OLR1117
OLR179
OLR192
SULT6B1L
UNCX4.1
ZFP569
CSTA3
CSTDC6
H2A
IFNA9
MIR133
MIR599
PDK
AVBD10
DOCK9-DT
EMILIN3A
GLMNB
KIAA1462
MAGI2A
OLFR380
OR13D1
PTX1
RBMY2HP
RPRL2
RPS4
SSFA2
TAX-4
TCP10L3
TMEM183B
C16ORF90
C78228
CEROX1
CTAG1B
CYPT4
D1ERTD507E
D7ERTD128E
D7ERTD807E
FAM27B
FSN
FTHL17A
GLIPR1L3
HOX1
HOXB1B
IGK-V21
IGKV3-1
IGKV8-27
ISL2A
LINC00672
LIPO4
MIR6395
MIRA
MUCL2
NR2F6B

NRON
OLFR1077-PS1
OLFR109
OLFR1428
OLFR1474
OLFR965
PLATR25
PLATR26
QUO
RARA-AS1
RBM3OS
RBMS1P1
RHOX1
RHOX13
SEPT5A
SKINT4
SOL
THOC2L
TRAV3-3
TTTY12
UBE2L1
VIPB
AANAT1
MIR467D
PUF-8
RGD1561551
SPE-15
SPE-17
DEFA2
HMGB3A
ILP3
MCP1
SLC3A2B
UNC-43
ASTX6
CBP
DAT
FOXD2OS
GZME
IGHD
IGHV14-4
IGHV1-47
IGHV1-72
IGKV1-133
IGKV4-55
KLRA18
LINC00299
LINC02603
MAGEB5
MK1
MRLN
NGS
NLRP4B
OLFR1213
OLFR1245
OLFR1453
OLFR507
OLR1374

OLR1768
PIMR60
SNORA71D
SPIN2D
STAI
ZFP394
ATP5MGL
C78513
FABP5L2
P76
PPIAL4G
SNORD78
TRAV9D-3
B0207.7
C43E11.5
C55B7.3
D2045.5
DPYSL2B
F40G12.10
F42C5.5
F59F5.4
FRK-1
GPX-1
GST-15
GST-2
GST-40
GST-9
HEATR7B1
HNRNPUA
HSP-16.49
MAP1AB
MSP-74
MYO5AA
NEFMA
OLFR1130
OLFR1301
OLFR320
OLFR476
OLR1051
OLR1071
OLR1090
OLR1137
OLR1308
OLR1319
OLR1396
OLR1407
OLR1428
OLR1498
OLR1742
OLR360
OLR378
OLR427
OLR44
OLR582
OLR613
OLR807
OLR811
OLR82

OLR841
PALM1B
PCLOA
PLPPR3A
PUF60B
RGD1562515
RGD1563159
RGD1563263
RGD1564177
RGD1564657
ROL-8
SEC61GL
10-Sep
SMIM18
TSC22
VOM1R33
VOM2R18
VOM2R4
VSNL1A
AF366264
AHR2D
AHR2G
AMD-PS6
CACNA2D2B
CDC34A
COX7A2L2
CPEB1B
CRYGMXL2
CTH1
CYP21A2-PS
CYP2D41-PS
D19ERTD744E
DCTN3L1
DLA
DPH3B-PS
DRBP1
EEF1A1P15
ESP4
ETR1
FBXW18
FN1A
FTR50
GAS1B
H4C18
HA
HCEB
HIF1AL2
IGK-V20
IGKV6-32
IGLV2-14
IMPG2B
KHDC1C
KIRREL1B
LSM14AA
MCM6L
MPZL2B
MTCO1P12
MUP-PS17

MUP-PS19
MYH7BB
NEWGENE_620180
NSD1B
N-THGTG5
OLFR1084
OLFR1116
OLFR1133
OLFR1175
OLFR138
OLFR1454
OLFR322
OLFR328
OLFR597
OLFR773
OLFR912
OLFR981
OM-II
OPGA
PIN1RT1
PKMA
PPP2R5EB
PRL3D3
RAPGEF4OS1
RGD1307182
RGD1310852
RGD1561694
RGD1562863
RGD1564405
RGD1565798
RIP
RMDN2-AS1
RPL17-PS1
RPL23A-PS1
RPL31-PS8
RUNDC3AB
SOS
SULT2A-PS1
TAS2R139
TRBV4
TSPY26
UGT1A9-PS
UGT5C2
USP17LE-PS
V1RG10
VIPR1-AS1
VMN1R66
VMN2R-PS11
VMN2R-PS57
ZFP970
ZNF728
ABLIM1A
ACAP3B
ADNPB
APBB2B
APODB
ARHGAP12B
ARHGEF1B

ATP8B5A
B3GLCTA
B4GALNT4B
BAMBIB
BMP2B
CABP5B
CAPZA1A
CBX6A
CCDC85CB
CCL44
CCSAPA
CDC42BPAA
CLDN11B
CLIP1A
CLUHA
CNTN1B
CREB1A
CUX1B
CXXC5A
DGAT1B
DHRS11A
DYRK1AB
EDNRBA
EPHA4L
FBXO40.1
FNDC5B
FYCO1A
FYCO1B
GAL3ST1A
GNB5A
GRAP2B
GRIA1B
GRIA4B
H2AZ2A
HHLA2B.2
HIPK3B
HLFB
HMOX2B
IGSF5A
KCTD9B
KDF1A
KIDINS220A
KRT1-C5
KRT94
LHX1A
LIPEB
LRRC4BB
MAP4K6
MCH
MCTP1A
MRM3B
MUL1A
NCK1A
NDUFAB1B
NOL4LB
NR4A2A
PDE5AA
PHF21AA

POTEM
PPIP5K1A
PPP1R14AB
RNF145B
RREB1A
RTN2A
SH3BP5LB
SIX3A
SKOR1A
SLC18A3A
SLC29A1A
SLC9A6A
SLKB
SPAG9B
SPPL2
STAT1A
SYNPO2B
SYT14B
TAPT1A
TMEM41AB
TNK2B
TP53BP2A
TRIM2B
TRIM33L
TRPM4B.2
UBE2IB
UBTFL
ULK1B
VAPAL
WAPLB
WNK1A
YWHABL
ZDHHC23A
ZNF576.2
ZNF710B
ZRANB1B
CSTDC5
IL12BA
NAP1
OLFR1183
OLFR143
OLFR1497
OLFR1510
OLFR17
OLFR429
OLFR437
OLFR516
OLFR651
OLFR855
OLR1330
OLR1392
RGD1304624
RGD1561667
RGD1564463
TLR15
VMN1R201
VOM1R24
AGFG1-PS1

ASS1P11
C17ORF50
C9ORF102
CCT6A-PS7
CCT7-PS1
COL6A4P2
DNAJC19-PS
EFCAB9
EIF6-PS1
EPHX1L
FABP5P2
GAPDHP72
GRLF1-PS1
IGD
IGHV1-53
IGHV1-55
KIR2DL5A
KLF5-PS1
KLRA19
LPPOS
MIR292B
MIR297-1
MIR5125
MIR548H4
MUP-PS3
NAT
OLFR1013
OLFR1111
OLFR1115
OLFR1355
OLFR1411
OLFR203
OLFR668
OR2L3
OX
PPIDL1
RAB5AL1
RGD1304567
RGD1305704
RGD1311164
RGD1560341
RGD1560398
RGD1560813
RGD1562281
RGD1562451
RGD1562667
RGD1563410
RGD1563546
RGD1563601
RGD1564515
RGD1564613
RGD1566212
RGD1566325
SERPINA3J
SLC22A7-PS1
SLC25A52
SMS-PS1
TCAF2C

TIMM17AL1
UAP1L2
UBA2-PS1
VOM2R50
VOM2R-PS40
ADRA1AB
ALPHA-EST10
ANKRD52A
ASB12A
BTBD6B
CEACAM9
CEP170AA
CLDN34A
CTDNEP1A
CYP12A4
CYP12B2
CYP12C1
CYP303A1
CYP6A17
DEFB103A
DMRT3A
DRD
EIF3HA
EVLB
EZRB
FBXO30B
FBXW16
FLR
GALR2B
GIT2B
GPC1A
GSTD6
GUK1A
HML
HOXC5A
HYDR1
ILLR1
IRK1
ITGA3A
JHI-26
KANTR
LECTIN-24DB
MAP6A
MFSD14A2
MTBL
MTHL2
NR4A2B
NTR
OBP57A
PAX1B
PIP4K2AB
PKN1B
PSGB1
RBM25B
RBPJB
RRBP1A
RTKN2A
SB

SELENOW2A
SIAH2L
SLC35A3B
SLC7A14A
SLKA
SUB1A
SYNCRIPL
SYPL2A
SYTL2A
TES3-PS
TIMM23A
TNS2A
TOTM
TRPN1
UBE2Q2L
UGT37B1
VOM2R-PS45
YELLOW-F
YP2
YP3
ZFP229
ZP3C
ZPCX
BF2
C15ORF56
CHIA.1
CTRB
DEFB108B
EWSAT1
H2BW1
IGKV1-5
LGALS9B
LINC00634
MAGEB6B
OR5L1
PRAMEF15
RAF
RHOGB
RPL21P44
RPL29P15
SPE-10
TINP1
TTTY13
ARA
BRWD1-AS2
BTF3P12
BUB1B-PAK6
CAPN10-DT
CEACAM15
CKS1L
COL18A1A
CORO7-PAM16
CRYGM1
CRYGM3
DTX2-PS1
DVL1B
EFR3BB
EGFR-AS1

ERB2
ESR1.L
FAM230B
IGKV1OR15-118
IL8R
KLC1B
KRT16P6
LIMS4
LINC00881
MIR1248
MIR512
MIR524
MIR624
MIR6769A
MIR6779
MIR876
MMP24OS
MMP25A
MROH7-TTC4
MTRNR2L10
NBPF25P
NF2B
NHR-140
NHR-47
NHR-59
NPTN-IT1
OLFR131
OLR1000
OLR129
OLR142
OLR1684
OLR204
OLR339
OLR414
OR10B1P
PABPC5-AS1
RASGEF1BB
RGD1559575
RGD1561134
RGD1561853
RGD1563835
RGD1563947
RGD1565862
RNU1-2
SERPINA14
SKINT8
SNORA26
SNORA51
SOCS9
SRSF6B
STAT1-2
STXBP3-PS
TAX-2
TCP11X2
TNNI1D
TRIM74
TRYP
VIG1

VMN2R94
YPEL2B
ZC3H11B
ANCE
C3ORF36
CYCE
PDE5
ABU-15
ADPGK-AS1
ARF79F
ASNA-1
BORCS7-ASMT
BSN-DT
BTBD10A
C14A6.8
C16C8.14
C32H11.9
C35B1.5
CLDN10L2
CLEC-66
CPR-5
DPY19L2P3
DSG2-AS1
EGR
F36G9.15
F39B2.3
F55G11.6
F56A4.2
FAM21A
FBXA-2
FBXA-217
FLOT1B
GAL3
GAS5-AS1
GCDHB
GRNAS
GRO1
IGFBP7-AS1
IRG-6
KIAA1919
KRTAP14L
LINC00102
LINC00858
M03E7.3
MIR1204
MIR1282
MIR181A1HG
MIR1902
MIR3661
MIR4458HG
MIR601
MIR9
MIR92A2
MLLT10P1
MTRNR2L3
NAPA-AS1
NMA
NRP1B

NRP2A
OLR108
OLR1083
OLR1087
OLR1105
OLR1125
OLR127
OLR1303
OLR1341
OLR1380
OLR1397
OLR1425
OLR1450
OLR1491
OLR150
OLR1509
OLR1530
OLR154
OLR1606
OLR1631
OLR1726
OLR1736
OLR181
OLR198
OLR230
OLR304
OLR336
OLR484
OLR493
OLR56
OLR684
OLR704
OLR715
OLR718
OLR741
OLR868
OR102-1
OR115-1
PCNA-AS1
PPT2-EGFL8
PSN
R09E10.2
RAB4B-EGLN2
RAD51L3-RFFL
RBAK-RBAKDN
RGD1562492
RGD1562914
RGD1563917
RL15
RPLP2P3
RS14
SERF2-C15ORF63
SLC7A11-AS1
SLCO6D1
SMPTB
SNAR-A1
SNORD124
SNORD84

SPAG5-AS1
TALDO
TM4SF19-DYNLT2B
TMEM256-PLSCR3
TNFAIP8L2B
Y60A9.3
ZNF559-ZNF177
ACANB
ACTG1P1
AK3L2-PS
ALS2CR11
ANAPC1P2
ANKRD18CP
ANTXRLP1
APITD1
ARHGAP27P2
ATP5SL
ATXN2-AS
BCMO1
BET1P1
BF
BI-1
BLOC1S2-PS
BNIP3P4
BRK
C11ORF88
C12ORF71
C22ORF24
C3C
C76213
CA5AP1
CADM1B
CALM5
CASC19
CD8B2
CELF2-DT
CES4
CHCHD2-PS
CHODL-AS1
COX5BP6
CRYGM2D10
CT83
CXADRP2
CXORF21
D4ERTD617E
DCI
DDX39
DEFB104A
DEFB128
DEFB134
DEFB135
DICP3.1
DLG1-AS1
DLG4B
DNAH10OS
DNAJB8-AS1
DPP4-DT
DPRX

DTNBB
DUX4L26
EFCAB4B
EIF2S2P2
EMR3
EXOC1L
FAIM2A
FAM123C
FAM129C
FAM134A
FAM150A
FAM150B
FAM159A
FAM179A
FAM197Y2
FAM19A4
FAM237A
FAM26D
FAM46D
FAM48B1
FAM55A
FAM57A
FAM90A13P
FBXO30-DT
FCGR1CP
FER1L6-AS1
FGF1B
FRMPD2B
FTLP10
GAB4
GAGE12F
GAGE7
GNG5-PS
GOLGA6D
GOLGA8M
GSDMC4
GST
GULOP
GUSBP10
GUSBP5
GUSBP9
H2BFWT
H3P44
HIST1H2AL
HIST1H2AM
HIST1H2BA
HIST1H2BJ
HIST1H3B
HMGN2P47
HSD11B1-AS1
HSD52
HSPC324
IGHV1-24
IGHV1OR15-6
IGKV1OR-2
IGLV3
IL28B
IL29

IMPDH1P10
IQGAP
KCNJ2A
KCNMA1-AS2
KRT18P33
KRT18P34
KRT18P41
KRT8P3
KRTAP10-9
KRTAP19-6
KRTAP25-1
L1MD-TF17
L1MD-TF18
L1MD-TF5
LECT1
LEUTX
LINC00028
LINC00160
LINC00298
LINC00484
LINC00494
LINC00593
LINC00636
LINC00703
LINC00710
LINC00853
LINC00895
LINC00922
LINC00928
LINC00987
LINC01056
LINC01081
LINC01090
LINC01173
LINC01226
LINC01303
LINC01480
LINC01509
LINC01520
LINC01629
LINC01630
LINC01634
LINC01749
LINC01822
LINC01845
LINC01910
LINC01928
LINC01973
LINC02006
LINC02009
LINC02015
LINC02057
LINC02141
LINC02169
LINC02209
LINC02226
LINC02347
LINC02418

LINC02447
LINC02478
LINC02542
LINC02550
LINC02591
LINC02609
LINC02685
LINC02692
LINC02731
LINC02802
LINC02843
LINC02872
LINC02880
LSP1P5
MCM5.L
METTL8P1
MINA
MIR105-2
MIR1182
MIR1250
MIR1252
MIR1253
MIR1260
MIR1283-2
MIR1295
MIR135C-1
MIR1470
MIR184-2
MIR1906-1
MIR1941
MIR199A-1
MIR2117
MIR2185-1
MIR2185-2
MIR2185-3
MIR34AHG
MIR430A-12
MIR430A-13
MIR430A-14
MIR430A-15
MIR430A-18
MIR430A-2
MIR430A-3
MIR430A-4
MIR430A-5
MIR430A-6
MIR430A-8
MIR430B-1
MIR430B-10
MIR430B-11
MIR430B-12
MIR430B-13
MIR430B-14
MIR430B-15
MIR430B-16
MIR430B-17
MIR430B-18
MIR430B-19

MIR430B-2
MIR430B-3
MIR430B-5
MIR430B-6
MIR430B-7
MIR430B-8
MIR430C-10
MIR430C-11
MIR430C-12
MIR430C-13
MIR430C-14
MIR430C-15
MIR430C-16
MIR430C-17
MIR430C-18
MIR430C-2
MIR430C-3
MIR430C-4
MIR430C-5
MIR430C-6
MIR430C-7
MIR430C-8
MIR430C-9
MIR430I-1
MIR430I-2
MIR430I-3
MIR4432HG
MIR451
MIR453
MIR454A
MIR4649
MIR4660
MIR5046
MIR512-1
MIR515-2
MIR516A2
MIR516B1
MIR516B2
MIR519A2
MIR521-2
MIR526A1
MIR526A2
MIR548F1
MIR548I1
MIR549
MIR550-2
MIR620
MIR642
MIR669A-11
MIR669A-12
MIR669P-1
MIR669P-2
MIR6797
MIR680-3
MIR7161
MIR7-2
MIR892A
MIR892B

MOBKL3
MOD2
MRPS36-PS1
MYOSLID
NDUFV2-AS1
NF1P1
NINJ2-AS1
NPM1P9
NRXN2B
NTRK2B
OLFR1024
OLFR213
OLFR859
OLR1148
OLR1292
OR10A7
OR116-2
OR137-2
OR2G3
OR4K13
OR4N3P
OR52K1
OR6V1
OSTM1-AS1
PAPLNB
PAR1
PBOV1
PDE4DIPP1
PHKA2-AS1
PICSAR
PLAC9P1
POLRMTP1
PPARAB
PPP1R2P1
PRAMENP
PRL2
PRNT
PSLNR
PURBB
PYDC2
RBAKDN
RBMY1J
RHOXF1-AS1
RN7SL128P
RNF219
RNU6-2
RNU6-8
RPL23AP49
RPL31P11
RPL39P40
RPL5P12
RPL7L1P3
RPS2P37
SBNO2A
SCARNA11
SCAT1
SELT
SEPHS1P1

SIF1
SLC17A6B
SLC25A51P4
SMCR8B
SMILR
SMIM9
SNAR-A14
SNAR-B1
SNORD105
SNORD113-9
SNORD115-13
SNORD115-17
SNORD115-19
SNORD115-48
SNORD116-12
SNORD116-17
SNORD116-26
SNORD119
SNORD18C
SPATA31A6
SPDYE11
SPRED2B
SSX7
TAAR12H
TBC1D3P1-DHX40P1
TGFBR1B
TMEM155
TRIM3A
TRIM43A
TRIM64B
TRMT112-PS2
TSPY3
TSPY4
TTTY10
TTTY19
TUBAP13
TUBBP5
UBTFL1
UGT1A2P
XRCC6P5
ZNF645
ZNF767
ACL-14
ACOX-3
ACS-20
ACS-7
ADT-1
ALH-13
AMT-1
ARHGAP33OS
ARHGEF10LB
ASM-3
B0272.4
B0379.2
BAH-1
BGNT-1.3
BIGR-1
BUS-19

BUS-8
C01B10.6
C05B5.2
C05C10.5
C06B3.6
C06G1.1
C09F9.1
C10G8.4
C12D5.9
C14C6.2
C14H10.2
C16C8.18
C17F3.3
C25F9.11
C26B9.3
C27D6.3
C28D4.4
C28D4.7
C29F3.3
C30F8.3
C30G7.3
C34C6.3
C35E7.9
C36E6.8
C39B5.5
C39D10.7
C39D10.8
C42C1.9
C45G9.4
C49G7.3
C50B6.7
C53B7.2
CALR3A
CAPNS1A
CBD-1
CERS2B
CEST-1.1
CEST-12
CEST-27
CEST-28
CEST-35.2
CLCN5B
CLEC-117
CLEC-153
COL-139
COL-142
COL-156
COL-172
COL-176
COL-179
COL-54
COL-71
CPR-1
CXX1A
CYB-2.2
D1014.5
D1054.10
D1081.12

D1086.11
D1086.17
D1086.6
D1086.7
D2063.1
DAF-22
DHS-19
DPY-1
E03H12.7
EEF1A
ENTPD2A.2
EPB41B
EXL-1
F07H5.4
F08H9.2
F10D11.6
F10D2.8
F12E12.1
F13E9.15
F13H8.5
F17E9.4
F18A11.2
F23C8.8
F23D12.11
F23H12.5
F25H5.8
F26B1.8
F27C1.3
F30A10.12
F33D4.6
F34D10.8
F35D2.1
F35E8.1
F40E3.5
F44F1.4
F45D11.14
F45D11.16
F47B8.10
F49C12.7
F49D11.6
F53B1.4
F53E10.1
F53G12.8
F53H4.2
F54C8.1
F55B11.2
F55B11.5
F55F8.7
F56A11.6
F56B3.6
F56D6.13
FIPR-5
FIPR-9
FMO-5
FURINA
GEI-13
GLB-29
GRL-10

H10E21.4
H2AX1
HEPHL1B
HIST1H4I
HOXAAS2
HRDE-1
HRG-1
IGKV5-43
K02A6.3
K02E11.7
K07A12.5
K07B1.4
K07H8.5
K08B12.1
K08C9.1
K09C6.8
K09E4.1
K11D12.13
KLRA13-PS
KLRA6
LGC-21
LMX1BA
LON-8
M03F8.1
M28.9
MAM-3
MAOC-1
MBOA-1
MEC-1
MEX-5
MEX-6
MIR146B-1
MIR2188
MIR2189
MIR731
MIR936
MIRLET7J
MLC-7
MLT-8
MLT-9
MPST-5
MSD-1
MSD-3
MSP-42
MSP-52
MSRP-6
MTUS1B
MUP-4
MYO-2
NA
NAA15A
NAS-29
NELL2B
NEP-10
NHR-113
NHR-21
NHR-264
NHR-73

NSPD-6
NSPD-7
OAC-1
OAC-25
OLFR1308
OLFR266
OLFR604
OLR1237
PAG-3
PALS-37
PARGL
PERM-2
PERM-4
PHAT-4
PRL3C1
PTR-1
PTR-14
PTR-18
PUC
PUD-1.1
PUD-1.2
PUD-2.1
PUF-3
PYCR1B
R04B3.1
R09E10.6
R09E12.9
R105.1
R13H9.5
RBM-3.1
RHOUB
RMD-4
RNY2
SCN1BB
SCOCB
SCRM-6
SDZ-24
SEM-2
SKPO-1
SKPO-2
SLO-1
SNF-2
SPE-8
SPN-4
SPP-13
SRW-86
SSP-34
SSP-35
SSP-9
STIM2B
STX2A
SWSN-9
SWT-6
SYCN.2
SYNGR1B
T05H4.8
T06D8.10
T06E4.10

T06E4.14
T06E4.8
T10B5.8
T12B5.15
T19C3.3
T20D4.12
T20F5.5
T22B7.3
T26C5.2
TAS2R130
TBA-8
TENT5BB
TMEM-135
TMEM79A
TTR-34
TTS-2
TWK-11
UBXN-5
UGT-37
UGT-5
ULE-2
ULE-4
ULE-5
VOM2R38
W03C9.8
W03D2.9
W03F8.6
W09C3.7
Y105E8A.27
Y106G6A.4
Y32G9B.1
Y37D8A.19
Y37F4.5
Y38C1AB.5
Y39A1A.2
Y47D3A.31
Y47D7A.15
Y47G6A.15
Y49E10.10
Y49E10.29
Y49F6B.8
Y53F4B.25
Y53F4B.27
Y54G2A.10
Y57G11B.5
Y57G11C.52
Y57G11C.8
Y58A7A.3
Y59E9AR.7
Y62E10A.13
Y62H9A.4
Y65B4A.9
Y66A7A.9
Y6E2A.10
ZC434.3
ZFP738
ZFP950
ZK1025.3

ZK1251.5
ZK512.8
ZK795.2
ZK813.1
ZK813.2
ZK829.3
ZP2.6
BAMBI-PS1
HBAE1.3
HBEGFA
KIR3DX1
M17
RGD1359158
SERPINB6E
42SP50
AHCY.L
APLNR.S
APOL7B
CALB1.L
CAMK2G2
CELA1.1
DEFA10
DEFB28
DRD-5
FADS2B
FASN1
GJA8B
HAL.2.L
HIST1H2AE
HIST1H3H
HOXA9A
IFNA12
IGKV16-104
IGKV3-10
IGKV3-7
IMP
IRX3A
IRX3OS
KCNK5A
KRT12.4.L
KRTAP16-3
LEC-7
MCMDC1
MIR6385
MIR6990
MIR8112
MSP-63
NKX2-5.S
NLRP9C
OLFR103
OLFR1261
OLFR1317
OLFR207
OLFR285
OLFR403
OLFR414
OLFR551
OLFR91

OLFR923
OLR1201
OLR1240
OLR1293
OLR1454
OLR1523
OLR16
OLR606
OLR652
OLR67
OLR737
OLR749
OLR769
OLR818
OOG2
ORLY
PIK3R3B
POLR2H-PS1
PRAMEL29
PTX
RGD1307554
RGD1308601
RGD1559532
RGD1559574
RGD1562569
RGD1563157
RGD1563222
RGD1563667
RGD1564324
RGD1564937
RGD1564999
RGD1565332
RHOX3A
RNF138RT1
RT1-CE15
SDR16C5B
SERPINB6D
SKINT7
SNCGB
SPATA31D1B
SPOPLB
SSP-31
TAAR8A
TEDDM2
TGIF2LX1
TPRG
TWSG1B
UBE2G1B
UGT-2
UNC45BOS
USP17LC
VMN1R195
VMN1R211
VMN1R25
VMN1R52
VMN1R70
VMN1R81
VMN1R87

VMN2R65
VMN2R88
VOM2R27
WFDC6B
XA-1.L
ZFP759
ZFP992
BK
BRACHYURY
CCDC85CA
CH25HL1.1
D2ERTD127E
DIXDC1A
FTHL17B
GAS1A
HOXB3A
IL17A/F3
KCTD15B
MS4A17A.5
OPN4A
OR7E47P
PBX3B
SNORD12C
SUV39H1B
USP46-DT
ZSCAN4F
CHCR1
CHCR2
CHIA.2
CYP2AC1
H2-M10.4
OLFR677
TAS2R123
UPK3BL1
WASIR2
ABI3BPB
ABTB2B
ACOT15
AFG1LB
ANGPTL1B
ANK3B
ANKRD6B
ANTXR1C
APLNR2
ARHGAP21B
ARMH2
ARRDC1A
ASAH1A
ASB13A.1
ATAD3
ATP6AP1A
ATP6V1E1B
ATRNL1A
ATRNL1B
ATXN1B
ATXN7L2B
AVPR1AA
BAS-1

BICDL2L
BNIP1B
BOK-AS1
BTBD10B
BTR04
C17ORF107
C1QL4L
CAPN1B
CAPN5A
CASPBL
CBX7B
CCR12A
CD28L
CDC42BPAB
CFAP97D1
CFHL3
CHL1A
CHMP6B
COBLL1B
CORO2BA
CRP2
CRYBG1A
CST14A.1
CST14A.2
CXCL8B.3
CXXC5B
CYP27A1.2
CYP2K8
CYP2X10.2
DHRS13A.3
DHS-23
DIXDC1B
DPY19L1L
ECE2A
EDIL3A
EIF4EA
EIF4G3A
ELAV1
ELOCB
ELOVL8A
EMILIN1A
EPOB
EPS8A
EPS8L3B
ERI3-IT1
ESAMA
ESPNLB
ESYT2B
EVA1BA
FAM160A1A
FBXA-46
FBXA-60
FGD5A
FGD5B
FKBP10A
FNBP1A
FOXO1B
FRRS1A

FTR59
GABRA6A
GAD1A
GB:BC139872
GCSHA
GGT1B
GGT5B
GIGYF1A
GLMNA
GLRX-10
GPR61L
GRB
GRINAA
GRM6A
GSTM2P1
HGFA
HS1BP3-IT1
IGSF5B
INKA2-AS1
INPP4AA
IPMKB
IQSEC1A
KANK1A
KCNH6A
KCNJ2B
KIF5BA
KIFAP3B
KLF6B
KRT18B
LDLRA
LHFPL2B
LINC00222
LINC00900
LINC01058
LINC01132
LINC01215
LINC01351
LINC01356
LINC01376
LINC01518
LINC01592
LINC01929
LINC02273
LMO7A
LMOD2B
LNX1-AS2
LPAR6B
LZTS2B
MAP1SA
MAP4L
MBF-1
MCOLN3B
MICALL1A
MIR199A1
MIR2909
MIR4284
MIR4489
MIR4748

MIR4804
MIR6321
MKK6A
MMRN2A
MOB2A
MPEG1.1
MYHA
MYO18AA
NADKA
NAV2A
NOTCHL
NSPA-9
OLR1063
OVOL1-AS1
PAPLNA
PAQR5B
PBX1B
PCDH15B
PDAP1B
PIMR129
PK
PLCH2B
PPM1NB
PPP2R2AB
PRELID1B
PSDA
PTK2BB
PTPN23B
RAB11FIP4B
RAB1AB
RAB5AA
RASGRF2A
REEP3A
RGS3A
RHBDF1A
RNASET2L
RWDD
RXFP3.2B
RYR2B
SACM1LA
SASH1A
SC:D189
SCPP1
SELENOO2
SEMA3FB
SEMA6BB
SH3GL3B
SHDA
SHRPRBCK1R
SLC12A7A
SLC16A5A
SLC25A30-AS1
SLC47A3
SLC48A1B
SLC6A19A.2
SLC6A22.2
SLC6A6B
SLC9A3R1B

SLCO5A1A
SMPD4P1
SNRKA
SOWAHAB
SPTLC2A
SRFB
SSBP3A
ST7-AS2
TBC1D3P2
TBR1A
TGFBR2B
THORLNC
THRIL
TLCD4A
TLR5A
TMEM107L
TMPOB
TNFAIP2A
TNKSA
TOPORSA
TSPEARA
TTBK1B
TUSC8
UBE2D1B
UBE2E4P
UBE2KB
UBL7B
UNC119A
Y52E8A.3
YME1L1A
ZK742.4
ZNF865
ZP3D.1
ZP3D.2
ANKRD20A5P
CD9A
CEH-14
DOXL2
HIGD2AL1
IFI213
IGHV1-69
IRS2A
LINC00968
NBPF12
OR52K3P
TMEM176L.1
TPI1P2
ZFP982
BEX6
CATSPER2P1
FABP5P1
HSPB1P1
LINC00240
LINC01881
MIR467B
OR7A10
RPL21P87
UTS2D

ABHD17AB
ALP4
ATPCL
CXCL8B.1
CYP12A5
CYP4D2
CYP4G1
CYP6A21
CYP6W1
DESAT1
DIP2BA
EIP55E
EP300A
FLN
FOXG1A
GSTD10
HOXA2B
HTR1AB
LINGO4A
LYSS
MAST1A
MF
MYO61F
OLFR464
PPO1
RAC3A
REG-2
SAM-S
SCP2B
SENP3B
SLC22A7B.3
SMAD6B
SPETEX-2D
SPINT1A
SRSF10B
STRN-MLCK
TMCC1B
TMEM54A
TOTC
TOX4A
TRAF4B
VSIG8B
WT1B
YP1
ZDHHC16A
CELF2-AS1
DIAPH2-AS1
KRTAP4-11
LINC00474
MIR29B
MIR323A
MIR571
MTERFD1
OR2S2
POM121L10P
POTEI
SKOR2
SPATA31E1

VOM1R90
AK7A
ALPK3A
ARL15B
ARVCFB
ASAP2A
BMF2
BRINP3A.2
CACNA1BA
CEC3
CFBL
COX7A
CTDNEP1B
CTDSPL2B
CYLDA
DDHD1B
DLX6A
DNAJC5GA
DUB
EBF1A
EEF2A.1
EFNB3B
EIF3S6IP
EPHB2B
FAM83HB
FGF12A
FLRT1B
FYNA
GALCA
GBGT1L3
GCDHA
GRAMD1BA
HPRT1L
IGF2BP2A
JAGN1B
LAMB2L
LMBRD2A
MALT2
MATN3B
MTMR7B
MYLK4A
MYT1A
MYT1B
NCK2B
NRXN3B
OTUB1B
PCDH7A
PDCB
PHB2B
PHLDB1A
PIP4K2CA
POC1BL
PPIFB
PTDSS1B
PVRL2L
RCVRN3
RHOGC
RHOT1B

RNF11B
RP1L1B
RPRD2A
SATB1A
SCDB
SH3BP5A
SLC5A8L
SMCR6
SOGA3A
SPI1B
STAP2A
SYT9B
TCIRG1A
TGM1L1
THSD7AA
TRAF2A
TRBV21-1
UGT5C3
UQCRC2B
ZIC6
ZNRF2A
CRYGM2D18
IFNL1
IGLC1
OLFR12
OLFR1311
OLFR283
OLFR368
OLFR99
OLR1700
SMOK2A
SPG4
C17H6ORF52
CCT6A-PS1
CD1
CD82A
CLEC2DL1
CTR1C
CYCA
D7WSU130E
DEFB50
ENTPD3-AS1
FOXB1A
GST-24
HSC70-PS2
IGHV1-2
IGH-VX24
IGKV4-59
ITP
LCN11
ME
MIR3120
MKRN4
MTNE
MYOM1A
N
NCL-PS1
NDUFV3-PS1

NEK2L1
NSUN5P2
OAZ1-PS
OBOX1
OLFR1086
OLFR1097
OLFR32
OLFR5
OLFR715
OLR107
OLR1077
OLR1111
OLR1172
OLR120
OLR1356
OLR1391
OLR1394
OLR1399
OLR144
OLR1475
OLR1516
OLR1531
OLR1638
OLR1639
OLR1682
OLR221
OLR434
OLR444
OLR50
OLR788
OLR862
OLR889
OLR921
OLR97
OR103-5
OR111-8
PEBP1-PS1
PLA2G4CL1
PPP1R26-AS1
PRAMEL1
PRAMEL7
PRR15LA
RBM12B1
RBPJL2
RGD1311744
RGD1561715
RGD1561777
RGD1561795
RGD1561796
RGD1563049
RGD1563482
RGD1564400
RGD1624210
RPL31L2
RPS24-PS3
RS13
RT1-M10-1
TAF4A

TMPRSS3A
TPC1808
TRDC
TSPAN35
UTS1
VOM2R15
VOM2R51
VOM2R60
ZFP141
ZFP683
AF067063
CYP21A1P
F13A
KRT18P30
MIR1203
MIR1268B
MIR1273H
MIR1285-1
MIR2467
MIR3144
MIR3605
MIR4267
MIR4433B
MIR4448
MIR450B-1
MIR466B-2
MIR5588
MIR6815
MIR6852
MIR721
NAGLT1
OLFR1273-PS
OR52N4
OR5M3
PRL8A3
RGD1564425
RPL37AP8
SNORA35
SOX19
TRX2
TUBB4B-PS1
VMN1R239-PS
VMN1R65
ADAM21P1
ADGRL3-AS1
BABAM2-AS1
BAGE3
BAGE4
BARX1-DT
BCDIN3D-AS1
C2ORF83
CLEC2D2
COL6A4P1
CSN1S2AP
CYP4F37
DAZ1
DEFB105B
DUX4L5

FABP5P3
FOXD4L6
HSFY1
INHBA-AS1
JAZF1-AS1
KATNBL1P6
KRTAP9-2
KRTAP9-4
LARGE-AS1
LHFPL3-AS2
LINC00237
LINC00272
LINC00310
LINC00504
LINC01012
LINC01036
LINC01159
LINC01170
LINC01250
LINC01494
LINC01561
LINC01566
LINC01586
LINC01608
LINC01683
LINC01762
LINC01924
LINC02337
LINC02388
LINC02389
LINC02487
LINC02762
MBD3L3
MBD3L5
MIR2052HG
MIR4500HG
MIR4700
MYO5BP2
NACA2
NDST1-AS1
OR11H1
OR1L1
OR1L6
OR2AK2
OR51G2
OR5M9
OR9G1
PCDHB18P
PCGEM1
PMCHL1
PRSS40A
PTCSC2
SEPTIN7P9
SHLD2P1
SLC25A51P1
SNORA41
SNORD108
SNORD113-2

SNORD115-39
SNORD31
SNORD91A
SPATA31D5P
ST7-OT4
STAU2-AS1
SUGT1P4-STRA6LP-CCDC180
TM4SF1-AS1
TP53TG3B
TRIM77
TTTY6B
ULK4P1
ERR
LGALS16
PIRA2
RGD1309779
SSX4
TTX-1
VMN2R37
ANKRD20A1
CART4
CBSL
CCT6A-PS2
CENPBD1P1
CHURC1-FNTB
COL28A2A
CYP3A40
CYP3C2
DUXAP8
FAM205A2
FAM230C
FBXW20
GAJ
GATD3A.L
HERC2P3
IGKV3-2
ISY1-RAB43
KCNJ2-AS1
MIR3135B
MIR3620
MIR4423
MIR548M
MIR579
MIR7083
MIR7687
MIRLET7F-1
MYCLP1
NEFLB
NHR-214
NKX1-1
NMDAR1
NPIPA8
OLFR1134
OLFR1307
OLFR224
OLFR330
OLFR372
OLFR391

OLFR456
OLFR469
OLFR538
OLFR681
OLFR703
OLFR787
OLFR794
OLR136
OLR137
OLR140
OLR141
OLR1500
OLR1673
OLR533
OPN4.1
PORA
PPP1R3AB
PRKAG3B
PTGES3L-AARSD1
RASA4B
RGD1309106
RGD1561381
RGD1564148
RIMBP3B
RIMBP3C
RNASE1L1
RNU4-1
SALL1A
SIK1B
SMG1P3
SMOK3B
SNORA2B
SNORA7A
SNORA81
SNORD3B-2
SPATA31
SPEER8-PS1
TRAM2-AS1
TRPD52L3
TUBA5
VMN1R73
VMN2R-PS54
VOM2R-PS24
ZFP948-PS1
ZNF528-AS1
ZSCAN4C
ADCY6A
AY074887
CDC42L2
FER-1
GLIPR2L
H2-Q9
HER8A
INSM1A
KCTD15A
MSX1B
NCK2A
PCDH1GB2

RAR
RPS15A-PS4
SCCPDHA.1
ST8SIA6-AS1
TEDDM1B
TMX2A
TUNAR
ZFP600
ZNF503-AS1
ALPHA-EST3
BIDA
C16ORF95
C18H9.6
C49G7.12
CALD1B
CED-13
CERS6-AS1
CLEC-5
CLIP1-AS1
COMMD3-BMI1
COMT-4
CYP-31A3
CYP4E3
CYP4P1
CYP6D4
CYT-B5
DCST1-AS1
DEFB109A
DRD-50
ENO1-AS1
F01D5.2
F35E12.6
F46A8.9
FAM66A
FBXA-163
FGD
FTH1P16
FTLP2
GNBP3
GRD
HAR1B
HCG25
HER4.5
HOXC6A
IQCJ-SCHIP1
KLHL7-DT
LINC01136
MCTS2P
MDY
MIR1287
MIR196C
MIR3936
MIR4271
MIR4273
MIR4321
MIR4664
MIR4737
MIR743

MSANTD3-TMEFF1
OAS1H
OLFR898
POLH-1
POLR2J3
PRX2540-2
PTP-5.1
RDL
RGD1304694
SAA
SENP3-EIF4A1
SNORA70B
SNORD139
SNORD76
SST-20
T24C4.4
TCF3B
TEN1-CDK3
TRIM35-27
UGDH-AS1
UGT-31
UGT-53
USP3-AS1
ZNF643
C11ORF16
CART1
DIP2C-AS1
GSDMC2
IFNL2
MIR1914
MIR673
NUTM2F
OLFR360
OLFR399
OLR1867
RGD1309748
SPEER2
TRIM35-34
VCY
ZNF446
AAED1
ABT-1
ABU-10
ABU-9
ACS-10
ACS-15
ACS-18
AGMO-1
ALG-3
ALG-4
ASP-8
B0205.10
B0207.1
B0218.7
B0261.6
B0280.11
B0379.7
BASL-1

C01G6.2
C01G8.1
C02B10.6
C02F5.5
C04F12.7
C05C12.5
C08F11.13
C08F8.6
C09B9.2
C09B9.4
C09D4.3
C14C10.1
C14C11.1
C14F11.4
C14F11.6
C15C6.2
C15H11.1
C15H7.3
C16A11.7
C17B7.4
C17F3.1
C18E9.8
C24D10.2
C27B7.6
C27D8.1
C27D8.2
C28C12.11
C32E8.4
C33F10.1
C33F10.12
C34D4.3
C34E11.2
C34G6.3
C35E7.10
C36H8.1
C38C3.3
C39H7.1
C40H1.8
C45B2.1
C45G9.9
C46E10.1
C47A4.3
C47E12.11
C48B6.4
C48E7.7
C50F7.3
C53D6.10
C54D2.1
C54G4.2
C55C3.4
CADPSB
CCDC94
CEY-2
CEY-3
CHT-3
CKB-4
CLEC-206
CLEC-73

CLEC-79
CLEC-87
CLEC-88
CNC-3
CNP-2
COL-101
COL-106
COL-119
COL-122
COL-20
COL-41
COL-60
COL-88
COL-96
COMP-1
CPG-1
CPG-2
CTBP-1
CTL-3
CYC-2.2
CYP2V1
D1086.10
D2092.8
DBPHT2
DCT-9
DHHC-13
DHP-1
DHRS-4
DHS-2
DNAH5L
DOD-22
E03H12.5
ECH-9
EEF1A1L2
EGG-1
ELO-8
F01D5.10
F07A5.2
F07G6.10
F09C12.8
F11G11.4
F13A7.1
F13A7.7
F15B9.8
F18F11.4
F21H7.5
F26D2.10
F26H11.4
F32B4.2
F32B6.4
F32H2.7
F33D11.2
F36A4.4
F36D1.4
F36D3.4
F36H12.3
F36H12.4
F36H1.3

F37A4.4
F37A8.1
F38E1.3
F38H4.4
F40F9.3
F40H6.1
F42A8.1
F42A9.3
F42A9.7
F45D3.4
F47B8.2
F47D12.7
F48D6.4
F49C12.15
F52E1.14
F52F12.5
F53B2.5
F53B6.4
F53B6.7
F53C3.1
F54B8.4
F55B11.3
F55C10.4
F55D12.6
F59A1.15
F59A6.4
FAT-5
FBXA-105
FGF13B
FHL3B
FIS-1
GFAT-2
GIPC-1
GIPC-2
GLD-1
GLF-1
GPD-1
GRL-15
GSTK-1
H06I04.5
H34I24.1
HOXB6B
HOXB8A
HOXC1A
HS3ST1L2
HYAL2A
IRLD-8
K01H12.4
K03H1.12
K03H1.9
K04G2.4
K05B2.4
K05C4.9
K05F1.9
K06A1.2
K07A1.5
K07A3.3
K07H8.8

K08C9.2
K08E7.5
K08F4.5
K09C6.7
KIN-21
KREG-1
LAB-1
LGC-22
LIPL-5
LTD-1
M117.4
M70.3
MAB-7
MIR216A-1
MIR3615
MIR738
MSRP-1
MSRP-2
MSRP-3
MSRP-5
MTSS1LA
NEP-1
NEP-8
NHR-11
NHR-25
NKB-2
NSPA-4
NSPD-1
NSPD-10
NSPD-2
NSPD-3
NSPD-5
OAC-29
OLFR1502
OSR-1
PARG-2
PCK-3
PQN-2
PQN-31
PQN-63
PRKACBA
PRSS59.2
PTP-5.2
PTP-5.3
PUD-4
PUF-5
R03C1.1
R08A2.2
R10E9.2
RGD1305807
RHOCA
RMD-3
RME-2
RNH-1.1
ROL-1
SCRM-8
SLC27A1B
SNF-4

SNF-5
SNF-9
SPCH-2
SPCH-3
SSQ-2
SSS-2
STH-1
SUPS-1
SWAP70B
T03F6.6
T04D3.5
T05A7.6
T05D4.5
T05F1.8
T07D3.9
T08B6.4
T08B6.9
T08H10.3
T10E9.4
T23B3.5
T23F6.3
T23G11.1
T25B2.2
T28B8.4
T28H11.7
TAG-10
TAG-344
TBH-1
TBX-34
TTBK-4
TTS-1
TXT-4
VRP-1
W01B6.5
W01B6.6
W03D8.5
WHT-4
WHT-8
WRT-1
XNTRPC
Y106G6H.13
Y113G7C.1
Y116A8C.23
Y11D7A.3
Y22D7AR.10
Y37A1B.5
Y38E10A.17
Y39B6A.30
Y39F10C.1
Y39G10AR.16
Y43F8C.5
Y43F8C.9
Y47D3A.13
Y47G6A.33
Y48B6A.5
Y51H7C.13
Y53F4B.11
Y54G2A.13

Y57G7A.6
Y59E9AL.3
Y59E9AL.6
Y62H9A.5
Y65B4BR.1
Y66D12A.11
Y69A2AR.14
Y69A2AR.23
Y71G12B.3
Y73F8A.20
Y76B12C.4
YWHAQB
ZC434.8
ZC443.3
ZC477.7
ZK1010.5
ZK1225.4
ZK154.1
ZK265.3
ZK354.2
ZK354.3
ZK354.6
ZK354.8
ZK512.7
ZK546.7
ZK596.2
ZK616.8
ZK858.8
ZK892.3
ZK945.7
ZK970.7
ADGRL3.1
GAPDH-PS1
GST-3
HBBA2
HPXA
HYPM
MEI4
OLFR1414
OLR1229
OLR404
OLR60
RGD1565059
VOM2R31
ADSSL
ANKRD2.L
BIRC5.S
EEF1A1O.L
FOSL2.L
GBX2.2.L
GNAO1.S
HOXC8A
IL8L2
LEFTY
LRE3
LRRC70
MSNA
NOT.L

NPY8BR
PLAUA
PLAUB
PRL4A1
RGD1562171
RGD1566373
RPT4
RX2
SEC22BB
SUMO2B
TEX49
TGM1L4
TLX3B
VALOPA
VENTX2.1.L
WASH1
APOAI
CHRNA2B
DEFB30
FOXG1C
GSTS5
HMOX2-PS1
IGKV3-20
INAB
MILL1
MYHZ1.2
MYO6B
OLR1387
OLR1411
OLR1513
OLR1515
OLR1734
OLR689
OLR728
OLR791
PRL5A2
RGD1359449
RNF41L
RPS27P29
RT1-M1-5
SUR-7
TTC39D
ZP3.1
ZP3A.1
ABCG2C
AGAP10P
ANK1A
AQP8A.2
ARID3A.S
BTR22
C3-1
C3-2
CAPSLA
CCDC149B
CEACAM13
CES2D-PS
CLDN5A
COMTA

CYP1A1.S
CYP3A6
DHRS7CB
DOC1
EHBP1L1A
EPB41L4AOS
ETHE1.L
FAM210AB
FAM226B
FBXW21
FGD4A
FGF10A
GAPDH-PS2
GEMIN7L1
GJA12.1
H3F3B.1
HLA-K
HOXC9A
IAP2
IFFO2B
IGHV1-82
KCNC3A
LIMCH1A
LINGO1B
LRRC4.1
LYGL1
MIR146
MIR376A2
MIR561
MIR6236
MIR6240
MIR6775
MLPHA
MLPHB
MRGPRA9
MTHFD1B
NEWGENE_2116
NEWGENE_2813
NGFRA
NKX6.1
NOTUMOS
OLFR1010
OLFR1020
OLFR1028
OLFR113
OLFR1154
OLFR1201
OLFR1248
OLFR1271
OLFR1362
OLFR1461
OLFR1506
OLFR2
OLFR211
OLFR235
OLFR263
OLFR293
OLFR298

OLFR304
OLFR310
OLFR376
OLFR453
OLFR566
OLFR592
OLFR593
OLFR598
OLFR610
OLFR646
OLFR653
OLFR692
OLFR707
OLFR724
OLFR727
OLFR765
OLFR799
OLFR834
OLFR867
OLFR891
OLFR90
OLFR918
OR2U1P
OR7E18P
PCK1.L
PLIN6
PLVAPA
POSTNB
PPAP2D
PPP1R27A
PRL7A2
PTP4A2A
RABGEF1L
RASA1B
RBMY2FP
REEP3B
RGD1561333
RGD1561662
RGD1562404
RGD1563668
RGD1563834
RGD1564053
RGD1564247
RGD1564606
RGD1597339
RHOT1A
RNF207B
RPL31L4
RPL5L1
RPS17L
RPS21-PS1
RPS27A-PS1
RXRBB
SDK2B
SERPINB3D
SLC22A13B
SLC6A11A
SNORD16A

SNPHB
SPSB3B
SSXB2
ST13P7
STAG1B
SULT3A2
TCF7L1A
TCP10A
TFDP1B
TMEFF1B
TNS1A
TPBPB
TRIM101
TRK-TTT3-5
TYRP1A
USP12A
VMN1R15
VMN1R174
VMN1R178
VMN1R200
VMN1R215
VMN1R220
VMN1R23
VMN1R230
VMN1R49
VMN1R59
VMN2R34
VPS20
WASH
XLR5A
ZFP677
ZFP983
AAK1A
ABLIM1B
ABTS-1
ACOT11A
ACTN2B
ACVR1BA
ACVR2AA
ADARB1A
AGLB
ANGPT2A
ANKRD10B
AQP10A
ARHGAP23A
ASB5B
ATAD1A
B3GNT5B
B4GALT1L
BAHCC1B
BCL11AA
BMP1B
C9ORF92
CA16B
CABLES2A
CABLES2B
CABP5A
CACNA1HA

CAMK1GB
CAVIN1A
CCL34A.4
CD151L
CD81B
CERT1A
CHADLB
CLEC-163
CLEC-2
CNN3B
COL15A1B
COMT-3
CREB1B
CSDC2B
CSGALNACT1A
CSNK2A2A
CTB-178M22.2
CUL1A
CYP-14A1
CYP2G1P
DAAM1A
DNAJA3B
EDNRAA
EIF3JA
EIF4G3B
EMILIN2A
F11R.2
FAM20CB
FAM20CL
FAM245A
FBXA-116
FTR01
FTR54
FURINB
FZR1B
GALNT18A
GAS7A
GCGRB
GJA11
GPR155A
GRK7B
GSTO-2
HBL4
HECW2B
HER12
HYKK.2
IER2B
ITIH3B.1
KCNAB2B
KIF26AB
KITB
KITLGA
LAMB1B
LAMP1B
LINC00605
LINC00700
LINC00892
LINC01486

LINC01548
LINC02103
LMAN2LA
LRP1BA
LRRC58A
MAGI1A
MAMDC2A
MAP3K14A
MCPH1-AS1
MEIS2A
METAP2B
MICOS10-NBL1
MIR196A2
MIR3188
MIR3196
MIR4301
MIR4690
MIR4800
MMP11B
MMP23BB
MPEG1.2
MS4A17A.10
MYH7BA
MYH7L
MYL12.1
MYOM1B
NAA15B
NDST2A
NEKL-2
NEXMIFB
NFAT5A
NIBAN1B
NKD3
NRBP2A
NRG2B
NYAP2B
OCLNB
OR10P1
OSBPL10B
OXGR1A.1
PCP-2
PDE4CB
PDE5AB
PDZK1P1
PGA4
PGFB
PIK3R6A
PIP4K2AA
PLCD3A
PLCD4A
PLSCR3B
PNP4A
POLR2EB
POU2F2A
PPFIBP1B
PPFIBP2B
PPM1AB
PRF1.5

PRICKLE2B
PRKAG2A
PRKAG2B
PRMT10
PROCA
PROZB
PSD3L
PSMA6B
PSME4A
PTK7A
PTPS-1
PTTG1IPA
RAB18B
RAB3DA
RAB5AB
RLTGR
RRAGCA
SERPINF2A
SGK2A
SH2D4BB
SKILA
SLC43A3A
SLC44A1B
SMARCA5-AS1
SMU1B
SORT1B
SOSTDC1A
SST6
SSUH2RS1
ST14B
ST6GALNAC1.1
STAP2B
STFA2L2
STX11B.1
SYNPO2LB
SYT5A
T19D12.3
TAOK3A
TCEB1
TDP2B
TGFB2-AS1
THBS4B
TIAR
TLE3A
TLR18
TMEM144A
TNRC6C2
TSPAN18B
TVP23C-CDRT4
UBE2L3B
VASNA
VASNB
VEGFBA
VMO1B
WASF3B
ZNF1015
ZNF341
ANTXR2A

CES2I
CLK4A
CYP3A85-PS
DMBX1A
IGKV6-25
LINC02878
MIR138-2
MIR30C
NDR2
NDUFA10L1
OLFR140
OLFR800
OLFR807
OLR1271
PIRA1
PNP2
PPP1R2-PS9
RGD1308750
RGD1559909
RGD1561154
RGD1566134
RNF11L1
RPL21P68
RT1-T18
SEK-1
TRNV
ZNF527
ZP3R
ACCOAS
CYP2B22
CYP4D21
CYP9B2
D6ERTD527E
EIF2S1A
GSTD7
GSTE7
IGKV5-39
MIR5119
MIR678
OLFR1243
OLFR1276
OLFR128
OLFR338
OLFR362
OLFR477
OLFR612
OLFR617
PBXIP1B
PGLYRP5
POTEKP
PRRX1A
PST
RABL2
RNF224
SAP130A
SNHG18
TAS2R113
TIE

TOTA
VMN1R63
VMN2R58
ZFP784
CDC42EP4A
DLX5A
EFEMP2B
FAM182A
MIR644A
MIR686
OLR875
RGD1560455
RPLP2-PS1
RT1-CE7
TTC30A2
ZFP831
ZFP839
CCDC182
DCHS1B
DLX
FABP1A
FGF6A
GUSBP3
H2BW2
INDO
MINPP1B
MIR1229
OLR132
OLR135
OLR1352
PR
RGD1564958
RGD1566035
TNNI4B.3
TRAT1
ADAMTS9-AS2
BA2
BANCR
CYP2K18
GTF2IP4
HEIH
MIR4298
MIR4732
MIR4750
MIR636
OLFR1033
OLFR308
OLFR490
PMS2CL
PSG16
SNORD64
TRIM39-RPP21
GPR142
OLR1346
ABHD11-AS1
ABHD2B
ALMS1P1
ATP1A1OS

BIB
C16C8.4
CALML6
CASP
CDF-1
CLEC-60
F55G11.4
GEMIN8P4
GGT3P
HIF1A-AS2
IGHG4
IL8
LAMB1A
LINC01569
MEK-1
MIR301
NACHRALPHA1
NCF1C
OLR1347
OLR157
OLR733
OLR736
PA2G4P4
PRSS40
RGD1559482
RGD1565498
RPS10-NUDT3
SLC25A25-AS1
SNORA52
SNORD45A
SNORD5
SNORD60
SNORD94
SNRKB
T01D3.6
TMEM262
TTC3P1
ZFP512B
ZNF561-AS1
ATP2A1-AS1
ATP6V1FNB
BAGE5
C12ORF73
C13ORF42
C20ORF173
C4ORF17
C5ORF58
C5ORF67
C9ORF62
CARD8-AS1
CBWD6
CCAT2
CDY2A
CNPY3-GNMT
CRYZL2P
DEFB114
DLX6OS2
FTH1P8

GPR158-AS1
GUCY2EP
HIST1H2BO
ISOC2A
KCNK15-AS1
LINC00260
LINC00448
LINC00535
LINC00661
LINC01707
LINC01754
LINC01842
LINC02008
LINC02092
LINC02167
LINC02188
LINC02288
LINC02363
LINC02511
LINC02516
LINC02538
LINC02613
LIPK
MBD3L4
MFSD4A-AS1
MIR1291
MIR1343
MIR151B
MIR1911
MIR3064
MIR3191
MIR3194
MIR3559
MIR3663HG
MIR5196
MIR6741
MIR6887
MIR710
MIR7977
MIR880
MIR940
MIRLET7A-2
MSTO2P
NBPF6
OLFR112
OLFR15
OLFR197
OLFR847
OR6B3
OR8G5
OR8J1
OR8U8
OR9G9
OR9I1
OSMR-AS1
PARGP1
PIN1P1
PNMA8B

PPBPP1
PRAMEF10
PRH2
PTCHD3P1
PWAR1
RGD1562178
RGD1564138
RGPD4-AS1
RPL23AP64
SNORA77
SNORD114-2
SNORD115-12
SNORD36B
SNORD66
SNORD91B
SPATA31C2
SYT14P1
TMEM14EP
ULK4P2
UPK1A-AS1
VEGF
ZFP862-PS
ZNF517
ZNF619
ZNF99
AAY
ABCF2A
ABU-1
ACDH-5
ACDH-8
ACSM6
ADGRB1B
AKR1C5
AMAC1
ANAPC1P1
ANT-1.3
APOA1-AS
APOOP5
ARGFX
ARID1AA
ARL4CA
ASNA1
ATP1A1B
AZGP1P2
BNIP3P1
BPIL3
BRP44L
BSK146
C01G12.3
C10ORF105
C10ORF62
C17H12.12
C18G1.9
C18H7.4
C1ORF228
C22ORF15
C29F7.2
C33F10.11

C33G8.2
C4ORF51
C7ORF33
C7ORF65
C7ORF66
CBPA1
CCDC155
CCDC168
CCDC36
CCL15-CCL14
CCNQP1
CDC-48.2
CECR1
CGB
CH17-340M24.3
CHST2A
CLEC-266
CLEC-6
CLEC-61
CNGA3A
CNN2P1
CNN2P3
COL11A1B
COL-184
COL-7
COL-80
COL-81
COL-90
COL-95
CRB2B
CRB3B
CRSP8P
CRYABB
CRYZL2P-SEC16B
CTAGE10P
CTAGE11P
CTAGE3P
CTSA-1.2
CXADRP3
CYN-2
CYP-13B1
CYP-25A1
CYP-25A2
CYP2J10
CYP-36A1
CYP9B1
DCAF8L1
DCXR-DT
DDX11-AS1
DECR-1.1
DHS-26
DIPK2AB
DM1-AS
DNM1A
DOK1B
DPRXP4
DRD4-RS
DRD5P1

DSCAS
DYTN
ENPP7.1
ENTPD1-AS1
ERC1A
F15D4.5
F25E5.8
F25F2.1
F26F12.3
F27C1.1
F31E8.5
F32A11.3
F58D2.2
FAM105B
FAM109B
FAM115C
FAM129B
FAM164C
FAM21
FAM224A
FAM224B
FAM47C
FAM49B
FAM90A20P
FBN2B
FILNC1
FUNDC2P2
GAGE10
GAGE12J
GDNF-AS1
GOLGA2P6
GPD-4
GPER
GPR125
GPX1P1
GRD-14
GST-33
GSTD9
GUCA1G
H05L14.1
H1FOO
HACD-1
HANG
HHLA1
HIST1H2AA
HIST1H2AI
HIST1H2AJ
HIVEP3A
HKR1
HNRNPCL2
HPYR1
HTAS-1
HTR3C
ICK
IGHV1OR21-1
IL22B
IRG-4
ISCA1P1

JMJD5
K04H4.5
K07A1.4
KCNJ1A.1
KIF9-AS1
KIR3DP1
KRT18P23
KRT18P68
KRT8P36
KRTAP19-7
KRTAP20-3
KRTAP22-2
KRTAP27-1
LDHAL6A
LILRP2
LINC00159
LINC00348
LINC00488
LINC00626
LINC00654
LINC00696
LINC00840
LINC00865
LINC00866
LINC00930
LINC01085
LINC01107
LINC01124
LINC01182
LINC01257
LINC01285
LINC01419
LINC01524
LINC01591
LINC01609
LINC01725
LINC01777
LINC01975
LINC02022
LINC02085
LINC02145
LINC02325
LINC02535
LINC02593
LINC02610
LINC02611
LINC02860
LINC02877
LINCR-0003
LINGO1A
LIPL-1
LIPL-2
LPHN3
LRRC2-AS1
LRRC37A11P
LRRC37A16P
LZTS2A
M60.7

MANEA-DT
MEX-1
MGRN1A
MIBP2
MIR1191
MIR1195
MIR1200
MIR1208
MIR1265
MIR1297
MIR1323
MIR153A
MIR329-2
MIR3976HG
MIR4251
MIR517B
MIR522
MIR548I3
MIR548I4
MIR548Q
MIR585
MIR591
MIR670
MIR727
MIR736
MIR941-1
MIRLET7A2
MPST-4
MRP
MYADML
NAS-20
NBPF7
NHEDC1
NHR-12
NID2A
NIPBLA
NKD2B
NLP-26
NOTUM2
NSPD-4
NUP62L
NXF4
ODC-1
OGT-1
OLFR1085
OLFR1105
OLFR1330
OLFR1466
OLFR192
OLFR329
OLFR594
OLFR824
OLFR95
OLR1115
OLR327
OLR778
OMT2B
OR10AG1

OR10G3
OR10G8
OR10G9
OR10H3
OR10H4
OR10S1
OR11H6
OR1B1
OR2AE1
OR2AG2
OR2G2
OR2M2
OR2T27
OR2T8
OR3A2
OR4A15
OR4A16
OR4B1
OR4D9
OR52I1
OR56A4
OR5A1
OR5AC2
OR5B21
OR5BH1P
OR5D16
OR5H15
OR5K3
OR5T1
OR6C3
OR6C68
OR6K6
OR6M1
OR6X1
OR7E37P
OR8B3
OR8D4
OR8K3
OR8K5
ORK1
OSGEPL1-AS1
OTOL1A
P2RX6P
P4HA1A
PAPSS2B
PCAT4
PCDH7B
PCMTL
PGBP
PGP-12
PGP-7
PHF2P1
PHOX2BB
PICART1
PIK3CD-AS1
PIP5K1CB
PISD-PS2
PLA2G16

PONZR10
POTEA
PP12613
PPIAP80
PPP1R2C
PPP4CB
PROX1-AS1
PRR23C
PRR7-AS1
PTR-22
PUD-3
R05H10.1
R07E5.15
R10E4.7
R155.3
RARRES3L
RBMY1F
RGD1304884
RNU1-70P
RPL7AP34
RPS10-PS1
RPSAP9
RRS1-AS1
RSU1P2
SAMD12-AS1
SATB2-AS1
SBF1P1
SCGB1B2P
SCTR-AS1
SEPSECS-AS1
SEPX1
SH3RF3-AS1
SHAW
SIX3OS1
SLC25A48-AS1
SLC44A3-AS1
SLMO
SLPIL2
SMIM21
SMIM32
SMZ-1
SNAR-E
SNORD113-5
SNORD114-10
SNORD114-11
SNORD114-13
SNORD114-14
SNORD114-16
SNORD114-17
SNORD114-25
SNORD114-27
SNORD114-29
SNORD114-31
SNORD114-7
SNORD115-14
SNORD115-16
SNORD115-2
SNORD115-29

SNORD115-3
SNORD115-41
SNORD116-14
SNORD116-20
SNORD36C
SNX18P7
SOBPB
SOCS5A
SPAG9A
SPANXN2
SPE-26
SPINLW1
SSH1A
SSQ-3
SSX9
STRIT1
T03G6.1
T06E4.9
T08G11.2
T21G5.1
T22B3.3
T23F11.2
TAGLN3A
TAOK2B
TBX5-AS1
TCEA1P2
TENM3-AS1
TEX36-AS1
TEX43
TMEM132D-AS1
TMEM272
TMEM85
TMEM8A
TOP1P1
TPTEP2
TRBV19
TREML5P
TRIM75
TRX-3
TSNAX-DISC1
TSPEAR-AS1
TSPY1
TSPY2
TTR-14
TTTY16
TTTY23B
TTTY4B
UBE2D2B
UGT-16
UGT-33
UGT-51
UNC-79
URO
USMG5
VAP-2
VENTXP1
VHLL
VMN1R16

VMN1R231
W01A11.1
W03F11.4
W09D6.4
WHT-5
WISP1
WISP2
WISP3
WTAPP1
XRCC6P1
Y106G6G.4
Y119D3B.13
Y38C1AA.7
Y38H8A.3
Y40H4A.2
Y45F10C.4
Y58A7A.4
Y59H11AM.1
Y69A2AR.19
Y71G12B.27
Y75B8A.23
ZFP955B
ZK938.1
ZNF238
ZNF534
ZNF716
ZNF733P
ZNF793-AS1
ACTR2A
ANOS1B
AP1S3B
ATP1A1A.5
ATP8B5B
CALCOCO1A
CNTNAP5A
D0KIST5
DNAH7B
DUX4
EIF3EA
FGFR1B
GNB1B
GTF3AA
HDAC9B
HIGD1A.L
HIGD1A.S
KMT2CB
MACO1A
MIR219A1
MYOZ2A
NDRG1A
NT5DC4
OLFR1036
OLFR1170
OLFR1280
OLFR1341
OLFR1532-PS1
OLFR259
OLFR297

OLFR530
OLFR577
OLFR901
PHF23A
PTK2AB
PYYA
REXO1L1P
SELENOT1B
SERPINH1A
SNAP23.2
TCNBA
TXLNBA
UBTD1A
VMN1R26
ZBTB16A
ZNF644B
AND4
ATP5I
CML
CRSP7
GAS-1
GLUD1A
HOXD4A
MHC1UBA
NABP1A
NEUROG2.L
OLFR155
PAX3A
PKP1B
PLECA
PPP1R2-PS3
PRLR.L
PROM
PSX1
RAP2C-AS1
RGD1561897
SIM1A
SPAG11B
SPAG6L
SULF2B
LY6AL
MLC2
OLFR318
PPP2R3D
RGD1559724
RGD1559960
RGD1562392
RGD1562652
SLC22A22
TRBV31
VMN1R42
ZFP781
ACOT17
ADGRL1A
ADRA1AA
AMOTL2A
AP2M1A
ARF4B

ARHGEF9B
ATP5IF1B
B3GNT2B
BAIAP2L1A
C3A.3
C55A6.6
C8ORF89
CANT1B
CASP6B.1
CBL-1
CBLN11
CD81A
CDKN2A/B
CFHL5
CHCHD3B
CHORDC1A
CLEC-9
CLN6A
CRLF1A
DNAJB6A
DYNLT2
EIF3HB
EVI5B
FAM102BB
FAM9B
GDPD3A
GPM6AA
GRB10A
GST-35
GSTA.2
H20E11.2
HSD20B2
ISP-1
JCADA
LINC01191
LINC01252
LINC02076
MIDEASA
MIR3470B
MIR4668
MIR5100
MIR669D
MIR691
MT1JP
MYLK4B
MYLK5
MYO18AB
MYO7BB
NAMPT2
NCS1A
NRD1A
NUDT4A
NUDT4B
OR2T10
PCOLCEB
PCP-4
PDZD3B
PELATON

PRG4A
PRKAB1A
PTH3R
PTPDC1B
RAC1B
RAC3B
RGS5B
RNF144AB
RPL34-DT
RPS6KB1A
SH3GL1A
SLC2A3A
SLC2A9L1
SLC44A5B
SLC4A4B
SLC6A16A
SMARCB1A
SORT1A
SPI1A
SPSB4A
STIM1A
THBS3B
TIGD7
TJP2A
TLDC1
TLE2B
TMC3-AS1
TMEM176L.3B
TMEM26A
TNIKB
TRPC4APB
TSL
TSPAN4B
UBL7A
VCANA
VN1R5
WNK4B
ZDHHC8B
APL-1
CTR1B
CYP11B3
KRTAP31-1
MTND
MYHZ2
OLR128
OLR1398
OLR1401
OLR287
OLR569
RGD1560028
RPS26P35
SMYHC1
ATTA
BIC
CAVIN1B
CCL20B
CNP1
CYP28A5

CYP2P9
CYP313A1
CYP4E2
CYP6A23
CYT-B5-R
EB1
F13A1A.1
FKBP1C
GOLGA2P7
GSTE3
ING5A
JHE
KLK1B1
KLK5L
LINC00667
MIR1296
MIR329
MIR95
MT1P3
OLFR1151
OLR139
OLR183
PKMB
RGD1306195
RGD1307947
RGD1560633
RNASEH1-AS1
SCARNA7
SNAI1B
SVIL-AS1
TIMM23B
SCARNA9L
ASP-14
BEND3P3
BOLA3-AS1
C1GALT1C1L
C1QTNF1-AS1
CFL1P1
ELO-2
F01D5.3
ILYS-5
LINC00266-1
MIR700
MNX1-AS1
NEBL-AS1
PMF1-BGLAP
PXN-AS1
SAP30L-AS1
SMG7-AS1
SNHG20
SNORD100
SNORD16
TLR2B
CAVIN4A
DEFA-RS4
DXERTD223E
EPB41L4A-DT
FHL1A

GUCY2E
IGF1OS
ITPK1-AS1
LIM2.3
LINC01547
MIR124A-1HG
MIR690
MIRT1
MOSPD4
OLFR1109
OLFR220
RN28S1
RPS15A-PS6
SLC22A7B.1
STRA6L
TRAV3-4
VMN2R85
AANAT2
ACS-17
AF067061
AMY2-PS2
ANXA3B
APOA1BP
ARHGAP42A
ATP1B2A
BCLX
BMPR1AA
C06A1.3
C25A8.5
C30G12.2
CDH-5
CED-11
CHCHD8
CLEC-70
COL10A1A
COL-124
COL12A1A
COL-143
COL-178
COL-181
COX6B1-PS1
CPT1A2B
CSAP1
CYP2AB1
DACHC
DEFB23
DGCR11
DLX4B
DOD-23
DOD-3
ECOP
EEVS
EHMT1B
EPAS1B
ESYT1B
ETF1B
EXT1C
F21H7.2

F53B2.8
F54D1.1
F59B1.8
FAM205A
FAM225A
FAM95B1
FAM99A
FAR-3
FAT1A
FOXJ1A
FOXR2
GEM-4
GLN-6
H2AB1
HINT1-PS1
HMGB1-PS3
HRG-7
IGHG2C
ITGA11B
JAG1A
K06A5.2
KLK1B9
MAMDC2B
MIR548E
MIR725
MITFA
MRGPRA1
MSD-4
MSX2A
NDEL1B
NEWGENE_1306455
NLRP1C-PS
NNT-1
NPIPB8
NPM3-PS1
OAC-14
OLFR1045
OLFR1079
OLFR1094
OLFR1141
OLFR1188
OLFR119
OLFR1282
OLFR1283
OLFR1359
OLFR1370
OLFR1418
OLFR148
OLFR165
OLFR25
OLFR307
OLFR381
OLFR527
OLFR655
OLFR678
OLFR694
OLFR730
OLFR772

OLFR802
OLFR809
OLFR860
OLFR894
OLFR963
OLFR972
OLFR995
OLR1108
PAPPA1
PAPPAB
PLDI
PLSCR3A
PM20D1.1
PPIAL4D
PPP4R1L-PS
PTGES3P3
RAB1B-PS1
RAB-3
RBM4.1
RGD1306941
RGD1311847
RGD1562402
RGD1563354
RGD1563570
RHOX11
RIPK5
RPL12-PS1
RPL17P7
RPL4P5
RPL6-PS1
RPS10L1
RPS19L1
RT1-DOB
SIP1
SIP-1
SKINT11
SMIM31
SNORD116
SNORD14A
SNRPGP2
SPETEX-2F
SST1
T09F5.10
T22B7.7
T27A3.5
TAS2R117
TAS2R120
TAS2R121
TAS2R134
TBC1D3D
TLE3B
TNNI2A.2
TRIM55B
TWIST1A
UCKL1B
UGT1A6.S
UGT-9
UNC-64

VMN1R202
VPS26
VTRNA1-1
Y39B6A.1
Y40H7A.10
Y48E1B.8
ZFP442
ZFP457
ZFP526
ZFP966
ZK105.1
ZK1193.2
ZK381.2
AZIN1A
C11ORF58
CD300LE
DEFB103B
ELT-2
HSP26
MYHZ1.1
OLR1395
OLR1413
RS2
RSSA
TAAR4
CYP18A1
CYP6D5
DNM1P41
GPDH1
GSTD3
GSTD5
JHEH1
LAMB2P1
LSP2
MIPEPP3
MIR103-1
MIR1207
MIR126A
MIR1909
MIR19B1
MIR742
OLFR1006
POLR1HAS
PPN
RGD1310429
RGD1562699
RGD1564614
RGD1566189
RPS16P5
SPANXN3
VOM2R40
ZFP773
ABRAA
ACTL10
BPY2
CFHL2
CSNK1A1P1
CYP4F30P

DAZ3
DLGAP1-AS4
FAM187A
FOXD4L1
FRG1-DT
GJC4A
HGO
HIST1H2AH
IFNA21
INTS4P1
ISG12(B)
KCNQ1-AS1
KRTAP22-1
LIM2.2
LINC00158
LINC00589
LINC00630
LINC01841
LINC02177
LINC02245
LINC02387
LYRM4-AS1
LYZL2
MITFB
NPHP3-AS1
OR1J1
OR2AT4
OR4C16
OR4F5
OR4K15
OR4X2
OR6C2
PAX1A
PSMB9A
PTCHD1-AS
RGD1563812
RGD1565611
RHCGA
RNU5E-1
SCARNA8
SEC14L6
SEPT7A
SNORA36A
TCAM1P
TEX26-AS1
TFDP1A
TRBV5-4
TTC41P
ZFP408
ZFP952
ZNF852
SLC6A20B
ABCB11B
ACOD1LB.L
ADRB3A
ASAP2B
B3GNT5A
BACH1B

C3B.2
CAMSAP1A
CAVIN2A
CBLN13
CPEB1A
CYP2AD3
CYP3C4
EGR2B
EIF3JB
EPB41L3B
FTHL27
GOT2A
HIST1H2AC
IGK-V28
KIAA1549LA
LEPB
LIMCH1B
LOXL1-AS1
LY6E-DT
MAGEB17
MAP1LC3CL
MCAMB
MEV-1
MGAT1B
MIR1202
MIR3195
MIR696
MKRN2OS.1
MSRA-1
MST1RA
MXRA8B
MYB-AS1
NSPE-1
NT5C2A
OLFR74
P38A
PAXIP1-AS2
PCSK5B
PDHK-2
PDK2B
PKP3A
POTEJ
PPM1DA
PRKCBP1L
PROX1A
PSMC1A
PTGDSA
PTPN4B
PWAR6
RNU5B-1
SAT1B
SFTPBB
SLC19A3A
SLC7A15
SNORA13
SNORA4
SNORD116-28
SNORD59B

SOCS2-AS1
SOUL2
SPAG1A
SPINK14
STK25A
SYPL2B
TAG-234
TAS2R109
TCF7L1B
TDO2B
TLR5B
TMEM51OS1
TRIM52-AS1
TRPA1A
UGT5A5
YWHABA
YWHABB
ZNF587B
ZNF629
ZNF8
ZNRD2-DT
ZTE38
AADACL2
ABCG3L4
ACSL1A
CLDNI
EIF3J1
FAM212B
GGT2
GSTE9
ICN2
LIPM
MAPKAPK5-AS1
MTF-1
NP
PP7080
PRKAR1AA
SMP-30
TEX19.1
TEX38
TNFSF10L
ZNF321P
C19ORF81
C20ORF144
CMAHP
CRYBB2P1
CTHRC1A
FAM86C2P
GABPB1-AS1
IGH-6
LINC00862
LINC01139
MIR297
PA2G4A
PEPCK
PRDM1A
PROSC
PSMB8-AS1

SBDSP1
SNORD68
TMCC1-DT
H1-9P
TPMA
AAA1
ABI1B
ACOX-1.5
ACTR3-AS1
ADCYAP1R1A
AGXT2L1
ALS2A
AP-1SIGMA
ASAP1A
BAALC-AS2
BACH2OS
BANF1P2
BMPR2B
C11ORF40
C12ORF40
C15ORF54
C1ORF146
C1ORF185
C1ORF229
C1QTNF9B
C20ORF141
C3ORF84
C5ORF49
CACNB3B
CAMKII
CCDC26
CCNB
CCPG1OS
CECR6
CHCHD2P6
CHIAP2
CLEC18C
CLEC19A
CNTNAP5C
CT45A6
CTAGE15
CTAGE6
CYP4F35P
D6ERTD474E
DDX3
DEFB119
DOS
DSCAMB
DSCR9
DYL
ELOA-AS1
ELOVL4A
ELSPBP1
ERBB4A
F42A10.6
FAM157B
FAM175B
FAM188A

FAM188B
FAM19A1
FAM19A2
FAM74A3
FAM84B
FBXC-58
FDX1L
FOLT-2
GAGE12G
GAS6-DT
GJB10
GOLGA8DP
GPR98
GSTTP2
H2AFV
H2AFY2
H2-M10.3
HBE2
HCG22
HIST1H3G
HLA-L
HLX-AS1
HMHB1
HNF4A-AS1
HSFX2
KCNJ1A.2
KRTAP5-8
LACE1
LACRT
LCNL1
LDB1B
LGALS17A
LINC00092
LINC00336
LINC00681
LINC00893
LINC00906
LINC00934
LINC01142
LINC01166
LINC01606
LINC01697
LINC01750
LINC01798
LINC02027
LINC02091
LINC02159
LINC02166
LINC02249
LINC02402
LINC02541
LIPJ
LPHN2
LRRC37A7P
LUNAR1
MAPRE3A
MESDC1
MIATNB

MIR10D
MIR1179
MIR124-2
MIR1322
MIR135A2
MIR1471
MIR449B
MIR4506
MIR466F-2
MIR518D
MIR520G
MIR596
MIR600
MIR602
MIR609
MIR655
MIR694
MIR878
MIR891B
MIR921
MMADHC-DT
MSNB
NANOGNB
NAS-12
NBPF22P
NEP-13
NKX2.2A
NPM1P35
OLFR341
OLFR411
OLFR619
OLFR710
OLFR985
OR108-1
OR10H1
OR10J6P
OR10K2
OR11H4
OR1E2
OR1F2P
OR1J2
OR2AG1
OR2D2
OR2D3
OR2L8
OR2M4
OR2M5
OR4E2
OR4L1
OR4N5
OR51M1
OR51T1
OR52E2
OR52R1
OR5AK2
OR5B2
OR5D18
OR5I1

OR5J2
OR5M1
OR6B1
OR6B2
OR6K2
OR6N2
OR6S1
OR6W1P
OR7A5
OR7G2
OR8A1
OR8H3
OR9A2
ORAOV1
PAK2A
PANTR1
PATE3
PGP-6
PHF11C
PKN2-AS1
PPA1A
PPBPP2
PPP2R5CP
PRAMEF4
PRR25
PRSS59.1
PSG21
PSMD10P2
PSPHP1
PTPRG-AS1
PWAR4
PWARSN
RFPL1S
RGD1560015
RH50
RHCGB
RPL13AP17
RPL36AP37
RPL7AP10
RPL9P11
RPS3AP47
S100A7L2
SAT2B
SEMA3GB
SERPINA13P
SFRS18
SLC8A1B
SLC9C2
SLFN14
SNL
SNORD113-1
SNORD113-6
SNORD114-12
SNORD114-15
SNORD114-22
SNORD114-28
SNORD115-4
SNORD115-40

SNORD115-42
SNORD116-15
SNORD116-6
SNORD12B
SNORD56B
SNORD83A
SPERT
SUPT6
T16G1.6
TAS2R7
TBC1D3H
TBX2-AS1
TFR1B
TMCO7
TMEM27
TRAV13-2
TRAV8-5
TRBC2
TTTY18
TUSC5
UGT-41
UGT-8
URAHP
USP17L1
VEZF1B
VGLL4B
VIT-2
WFDC10B
WFDC9
WRB
XPD
Y51H7C.1
ZFP551
ZNF32-AS3
ZNF789
FTH1P11
HIST1H2AK
KLRA23
MIR454
MRGPRB4
OLFR586
OLFR870
TCL1B4
VMN2R121
CHMP4BL1
GPX
IMPDH1B
LIM2.4
MHC2DAB
RGD1305938
RT1-CE10
TGM2L
THOR
UPK3BL
ZNF778
BCL6AA
FCGBPL1
LINC01184

NBPF20
RGD1562107
SLX1A-SULT1A3
SNORA57
SNORA7B
SNORD117
SRGAP2B
TMPRSS11B
TRAPPC14
UGT2B9*2
ZFP133
BRD7P3
GCNT4A
HHATLB
IFNA11
MIR19B-1
MIR378B
MIR4454
MIR664-2
OLFR24
OR1L4
POU3F3A
ZFP286A
ZFP580
ZFP623
ZFP821
AMPD3B
AOX2
AOX6
AQP
ARRDC3-AS1
BAGE2
BMP1A
C11ORF97
C19ORF71
C9ORF163
CAT-4
CBLNL
CCL19A.1
CDRT15P1
CEP170P1
CLTCB
CSRNP1B
CYP1B
CYP2K22
DECAY
DKK1B
EIF1B-AS1
EIF3J-DT
F11-AS1
FAM122C
FAM229A
FOXD4L3
FOXP1B
GABARAPA
GOLGA6L6
GRN2
GS1-124K5.11

GSTT2B
HADHAB
HDGFRP2
HOATZ
IGKV1-117
IL10RB-DT
KIAA1429
KRTAP5-AS1
KRTAP6-5
LCE3F
LINC00847
LINC01515
LINC02365
MEIOSIN
MICALL2A
MIG7
MIR137HG
MIR4453HG
MIR5189
MIRT2
MRC1B
MSL3P1
MUP11
MYO15
OAZ1B
OLFR1012
OLFR1018
OLFR1176
OLFR1219
OLFR1228
OLFR1231
OLFR1259
OLFR1265
OLFR1294
OLFR130
OLFR1300-PS1
OLFR1306
OLFR1318
OLFR1386
OLFR1441
OLFR146
OLFR1463
OLFR178
OLFR193
OLFR356
OLFR390
OLFR491
OLFR493
OLFR514
OLFR571
OLFR66
OLFR670
OLFR729
OLFR994
OR4C13
OR4C46
PCDH18A
PDCD10A

PDGFBB
PLK2B
PRICKLE1A
PRL7A1
PSMD11A
PWP2H
RAB39A
RAB7L1
RGD1302996
RGD1305110
RGD1305350
RGD1306746
RGD1310935
RGD1311084
RGD1359108
RGD1560821
RSA-14-44
RUNX2A
SAT1A.1
SEC14L1P1
SEPT9A
SINHCAFL
SLCO1F2
SNORD25
SNX9B
SPDYE7P
STIMATE-MUSTN1
TAAR7D
TAS2R102
TAS2R115
THBS1B
THBS3A
TM1
TMEFF2B
V1RD19
VMN1R14
VMN1R62
VMN1R67
ZDHHC25
ZFP619
ZFP72
ZFP957
ZNF815P
NME2B.2
OLFR175
OLFR922
PRR9
RGD1564854
RTP2
ZFP507
DIRC2
DLX6OS1
F9B
GATA1A
MIR153C
MIR496A
NPIPB11
PAPL-1

PCGF5B
POU5F1P3
PPP1R3CB
RPL29P11
RPL6P27
CKBA
GSTE1
HOXC4A
LIPOGENIN
LRRC37A
MAGI2-AS3
MS4A17A.1
POMCA
PRICKLE2-AS3
PRKG1A
RNU1-4
RNY3
TERFA
LINC00520
MIBP
MPTX1
GCSHP3
H60A
IGBP1B
MIR939
OAS1I
POU5F1P4
SNORD35B
ANXA13L
C3A.6
CAMK2D1
CASP3B
CDKN1D
CHCHD6B
F44E5.4
HADHAA
HSP-16.48
LDB3A
LIFRB
LINC00545
MIR668
NIT-1
NUMR-1
PEPCK1
PSMD4B
PTGES3A
PTMAA
RDH14A
SERPINB9P1
SYN2A
THRAB
TRP53RKB
TTN-AS1
AMY1B
C15ORF65
CRY1B
FAM90A7P
GBP1P1

IFNA10
LINC00540
LINC00963
LINC01238
MIR1271
MIR499A
OLFR59
OTX2-AS1
PGP-1
PPDPFA
PROSER2-AS1
RPL10P9
RRN3P2
SAXO1
SMTNL
SNHG19
TENT5D
VIT-4
ZFP433
ZFP708
ADAM25
ADH6A
AGAP2-AS1
AMY
ARHGEF35
C22ORF31
C2ORF73
C4ORF50
C9ORF139
CCDC129
CCDC185
CCR1L1
CD300H
COL4A3BP
CPB2-AS1
D11WSU47E
DOC2GP
ERBB2IP
FAM127C
FAM132A
FAM195A
FAM196B
FAM198A
FAM19A5
FAM238B
FAM26E
FAM47B
FAM60A
FAM86B2
GAPDHP23
GRAMD2A
GREM1A
GRID2IPB
H2AFJ
H2-T9
H60B
HBAE3
HBBP1

HIST1H3D
HIST4H4
IFIT1B
ITGB1A
ITGB2-AS1
KIF26B-AS1
KMT2CA
KRBOX1
KRT8P45
KRTAP10-3
KRTAP23-1
LHFPL2A
LIM2.1
LINC00051
LINC00167
LINC00239
LINC00606
LINC00689
LINC00908
LINC01300
LINC01312
LINC01514
LINC01622
LINC02269
LINC02652
MANSC4
MCCD1
MCHR2-AS1
MESDC2
MIR1953
MIR2114
MIR3158-1
MIR3189
MIR4749
MIR4758
MIR765
MIR889
MIR891A
MRGPRG-AS1
MS4A4E
MYHAS
NBPF13P
NXF3
OLFR1342
OR10C1
OR13A1
OR13C8
OR2A14
OR2A5
OR2V2
OR2Y1
OR3A1
OR51I1
OR51L1
OR52B6
OR52E6
OR52J3
OR5B3

OR5E1P
OR5H2
OR5T3
OR7C1
OR8B2
OR8B4
OR8H2
OR8K1
OR8S1
PBP
PCAT29
PCNXL2
PIK3CD-AS2
POU2F1B
PRAMEF1
PRKACBB
PTGER4P2-CDK2AP2P2
PTOV1-AS1
PTPRDB
ROCK1P1
RPL18P13
RPS6P25
SBPL
SIGLEC17P
SIGLEC8
SIGLECL1
SLC12A10.2
SLED1
SNORA80B
SNORD116-10
SNORD116-23
SNORD116-24
SNORD116-8
SPANXN4
SPATA31A3
SPCS2P4
TAS2R3
THRAA
TLX1NB
TMEM206
TMEM220-AS1
TMEM235
TMEM239
TRIM108
USP32P1
VMN1R84
VTRNA1-2
WHSC1L1
ZNF295-AS1
ZSCAN12P1
ATP6V0E2-AS1
NORAD
RNU4ATAC
CHIA1
DEFAL1
DEFB43
GCGB
GLRP1

HIST2H2AA3
HNF1BB
IFNZ
KIF19A
MIR27E
MIR545
NCAM3
NUPR1.L
OLFR1180
OLFR1222
OLFR1324
OLFR134
OLFR1427
OLFR1450
OLFR1462
OLFR1487
OLFR1509
OLFR398
OLFR49
OLFR550
OLFR711
OLFR887
OLFR888
OLFR906
OLFR910
OLFR951
PLXNB1A
RGD1307621
RGD1309651
RGD1311899
RT1-CE2
SIRPB1A
SVS2
TAFA1
THRB.S
TNFB
VMN1R89
ZFP496
ZFP566
ACS-2
FAM47E-STBD1
KDM7A-DT
LCN5
MIR181A2HG
SMCR5
ATP6V1C1B
BCKDHBL
CTDSPLA
GJA9-MYCBP
ITGA6B
MCF2L-AS1
PRKXP1
TSPAN13A
VDRA
KRT17P3
NPIPB2
PRKAG2-AS1
RNU6-15P

CES2J
LINC00662
LINC01560
MIR704
MNDAL
MZT2
OLFR1339
PARD6G-AS1
ZFP474
A2M-AS1
ATP2B1-AS1
C19ORF53
CTSLL
DUOX
FBXL19-AS1
HIST1H2BC
KRTAP5-2
LINC00937
PGA3
PRH1-PRR4
SNORA12
TFAP2A-AS1
TSC22D1-AS1
TSSK3
ZFP59
OACYL
COPG2IT1
EIF4E1C
GSTO-1
HSP30
IGFBP2B
OLFR1415
PAX2A
RPL21P28
ANTIPROT1
DEFA24
DEFB33
DLX1A
DLX1AS
EMX3
HMGN4
KLRI1
LCE1H
MIR153
NR5A1B
OLFR357
OLFR470
OLFR642
OLFR699
OLFR875
OLFR883
RGD1308544
RPS27RT
THRB.L
TMCO5
ZFP658
ZNF316
ACVR2B-AS1

ANP32AP1
ANPEPA
AQP12B
C16ORF96
C2CD4D-AS1
C5ORF64
C6ORF163
CBY3
CCL4L1
CDASE
CECR5
CLEC2A
CLECL1
CTAGE1
CYP2X7
DEFA10P
DPH3P1
EGOT
ELTD1
ERC2-IT1
FAM133CP
FAM175A
FAM19A3
FAM238C
FAM96B
GH2
GNBP2
GRM1A
GSDMC3
H2AFY
HNRNPA1P33
KIF4B
LINC00111
LINC00887
LINC01115
LINC01361
LINC01550
LINC02381
LINC02817
LRIT3
LYPD4
MGAM2
MIR103
MIR3944
MIR506
MIR551A
MIR575
MIR641
MKL2
MPC1L
MROH4
MUM1L1
MUTED
NLGN2A
OLFR1333
OLFR1505
OLR575
OR14I1

OR1A1
OR4X1
OR5AS1
OR5B12
OR5P2
OR7C2
OR9A4
OR9Q2
ORAI1B
PCAT18
PLAC9A
PRB4
RAB9BP1
RSPH3B
SNORD115-1
STOML3B
THEGL
TMEM2
TMEM249
WEE2-AS1
ZNF578
ANP32C
CCDC18-AS1
CDKN2B-AS1
EXOC3-AS1
GSEC
IGF1RB
OFCC1
PSG29
SCARNA3
TMEM161B-AS1
TRXR-1
VMN1R47
CBWD2
KIF25-AS1
LINC00294
TMEM236
TSPY26P
ACP5B
CALM1A
HSPE1-MOB4
KDRL
MTLN
RNY4
SMIM17
SODH-1
ZNF766
SEC24B-AS1
TNNI2B.2
AKR1C12L1
ATP1A1A.3
C4
EPT1
KCTD12.2
LINC01963
LPAL2
MIR2137
MIR378C

OLFR1131
OLFR1417
OLFR1445
OLFR150
OLFR231
OLFR53
OLFR570
OLFR578
OLFR620
OLFR907
RGD1561113
TEDDM3
TPH1B
TRBC1
USP17L5
ZNF667-AS1
ARRDC3A
BLACAT1
C10ORF143
CLDN20
LINC01134
MIR291A
PGM5-AS1
SBF2-AS1
ZFP422-PS
AIM1
AKR7L
AMELY
AQP4-AS1
ARMS2
ATP2B3B
ATXN3L
CCDC144NL
CCDC149A
CXORF58
DEFB129
EZHIP
FAHD2B
FAHD2CP
FAM103A1
FAM196A
FAM84A
FGF14-AS2
FOXD2-AS1
FSTL1B
GAGE1
GNRHR3
GSTS3
HIST1H3A
IFNW1
LINC00471
LINC00632
LINC00707
LINC01141
LKAAEAR1
MIR129
MIR365-1
MIR559

MS4A12
NCAM1A
OLFR272
OLR1583
OR2T1
OR4K1
OR8J3
POLR2J4
PRR34
PVRL3
RFPL3
SLC16A1-AS1
SLC25A21-AS1
SNAR-G2
SPATA41
SVS6
TAS2R40
TAS2R41
TAS2R60
TEX45
TMEM30C
TMSB4XP8
USP27X-DT
VN1R2
ZACN
DESMA
PTH1A
PURPL
SNORA73A
TNNT3B
ADH
CYP-35A3
HIST1H2AN
ZNF780A
ADAM8A
ASAP1-IT1
HSPA4A
LINC00648
MIR1468
MIR218-1
PRP2
TMPO-AS1
LINC00842
POTEG
PTCHD3
RBM12B2
ZNF251
DPPA1
GDPD4
NLRP4E
OLFR1173
OLFR168
OLFR229
OLFR930
SPATA9
RNU1-1
ZNF503-AS2
APTR

C15ORF40
C21ORF62
CASC1
CLEC4C
DBPB
DNM1P46
DRICH1
FAM127A
FAM64A
GLTSCR1
HCG4
IQCF5
L1CAMA
LBHD2
LINC00698
LINC00886
LINC02499
NRAD1
OLFR1443
OR2C1
OR4D2
OR4N4
OR7G1
PRAMEL4
PRSS44
SARA
SDCBP2-AS1
SERF1A
SLCO4A1-AS1
SLFN10-PS
SNAR-F
STPG3
TAAR9
TMEM57
TOP1P2
WHSC1
ZFHX4-AS1
ZNF683
ZNF878
CCZ1B
RN7SK
ZNRD1ASP
OLFR1039
OLFR267
OLFR481
OLFR559
TRY5
MIR292
NCOR1P1
FNDC9
ZNF718
APELA
ALDH
ATP1A2A
C8ORF88
CPLX4
CT45A1
DUXAP10

FAM46C
GRXCR2
HMGB3P1
IQCF6
LINC00942
MYBPC2B
NPBWR1
NXF5
OCM2
PARP15
PLEKHA8P1
PRSS33
PRSS50
RAMP2-AS1
RNF133
RPL23AP7
SNORD116-1
SPACA7
ZFP651
BMS1P1
C16ORF46
DEFB37
DRAIC
FAM153B
GZMN
HSF3
IRF1B
OLFR1323
ZFP251
ZFP949
PAXIP1-DT
CRHB
TMEM191A
ALMS1-IT1
COX8H
DFNA5
FNDC3C1
FOXO1A
FRG2
KRT3
MS4A13
NLRP9
SPATA31D1D
STMND1
TAPT1-AS1
UGT2A2
ZNF284
ZNF491
VMAC
ZNF214
OLFR447
ZFP933
ANKRD20A2P
ANKRD7
C10ORF82
C8ORF37
CFAP157
FOXQ1B

FRG2B
IQCH-AS1
LINC00894
MHC
NR1D4A
NRDC
PET117
POM121L9P
RSL1
SPANXB1
TRIM73
KLRB1B
MFSD14C
SUPT7L
TCAM1
ZFP397
C12ORF56
KRTAP2-3
LINC00115
MIR485
NPS
PNLIPRP3
SCP2D1
SPINT4
ZCCHC6
ZNF625
ZFP647
C8ORF76
MIR488
MSLNL
OLFR161
SNTN
ZNF501
MIR210HG
CARMIL2
CHT10
CLRN2
ERVMER61-1
FAM46A
FAM46B
HLA-DOB
NME9
RFPL4A
TMUB2
ZNF709
AOC4P
GTSF1L
OLFR401
PRR22
RPS4Y1
USP17LA
ZNF599
ZNF880
CYP19A2
LRRC37BP1
NUP210L
PGBD2
TRAF3IP2-AS1

ZNF547
ZNF596
CCDC175
MAGEB16
ZNF418
CEP126
ZNF416
PPM1N
FBXO27
C5ORF24
