# Supplementary material for: Pharmacological Mechanisms Underlying the Hepatoprotective Effects of Ecliptae herba on Hepatocellular Carcinoma
Source: Evid Based Complement Alternat Med. 2021 Jul 16;2021:5591402. doi: 10.1155/2021/5591402 (PMC8302389; doi:10.1155/2021/5591402)
Supplement: Supplementary Materials — Supplementary File S1: a total of 48 chemical ingredients of EH were obtained from TCMSP. Supplementary File S2: detailed information of the targets of 6 active ingredients in EH was extracted from three databases, TCMSP, DGIDB, and SwissTargetPrediction. Supplementary File S3: detailed information on HCC-related targets was extracted from GeneCards and CTD. Supplementary File S4: detailed information on the PPI network of 52 potential therapeutic targets for HCC was obtained from the STRING platform. Supplementary File S5: topological parameters of nodes in the E-H network obtained from Cytoscape. Supplementary File S6: detailed information on GO enrichment analysis obtained from WebGestalt. Supplementary File S7: detailed information on the top 10 GO terms of the GO network in the TCGA RNASeq LIHC database through Network Topology-based Analysis obtained from WebGestalt. Supplementary File S8: detailed information on the top 20 KEGG enrichment pathways obtained from the WebGestalt. Supplementary File S9: detailed information on the C-T-P network obtained from Cytoscape. [file 5591402.f1.zip › 5591402.f1/Supplementary File S4.pdf]

Detailed information on the PPI network of 52 potential therapeutic targets for HCC was obtained from the STRING platform												
#node1	node2	node1_string_id	node2_string_id	neighborhood_on_chromosome	gene_fusion	phylogenetic_cooccurrence	homology	coexpression	experimentally_determined_interaction	database_annotated	automated_textmining	combined_score
ABCG2	APEX1	9606.ENSP00000237612	9606.ENSP00000216714	0	0	0	0	0.061	0	0	0.464	0.476
ABCG2	AR	9606.ENSP00000237612	9606.ENSP00000363822	0	0	0	0	0	0.076	0	0.399	0.42
ABCG2	CYP1B1	9606.ENSP00000237612	9606.ENSP00000478561	0	0	0	0	0	0.05	0	0.418	0.423
ABCG2	E2F1	9606.ENSP00000237612	9606.ENSP00000345571	0	0	0	0	0.064	0	0	0.429	0.443
ABCG2	MET	9606.ENSP00000237612	9606.ENSP00000317272	0	0	0	0	0.062	0.056	0	0.433	0.454
ABCG2	PIM1	9606.ENSP00000237612	9606.ENSP00000362608	0	0	0	0	0.063	0.379	0	0.166	0.472
ABCG2	CYP1A1	9606.ENSP00000237612	9606.ENSP00000369050	0	0	0	0	0	0.05	0	0.486	0.49
ABCG2	ESR2	9606.ENSP00000237612	9606.ENSP00000343925	0	0	0	0	0	0.076	0	0.507	0.525
ABCG2	CYP1A2	9606.ENSP00000237612	9606.ENSP00000342007	0	0	0	0	0	0.05	0	0.522	0.527
ABCG2	TOP1	9606.ENSP00000237612	9606.ENSP00000354522	0	0	0	0	0	0	0	0.588	0.588
ABCG2	NR1I2	9606.ENSP00000237612	9606.ENSP00000336528	0	0	0	0	0.055	0.076	0	0.63	0.648
ABCG2	EGFR	9606.ENSP00000237612	9606.ENSP00000275493	0	0	0	0	0.08	0	0	0.647	0.661
ABCG2	ESR1	9606.ENSP00000237612	9606.ENSP00000405330	0	0	0	0	0	0.076	0	0.673	0.685
ABCG2	AHR	9606.ENSP00000237612	9606.ENSP00000242057	0	0	0	0	0	0	0	0.697	0.697

ABCG2	CYP3A4	9606.ENSP00000237612	9606.ENSP00000337915	0	0	0	0	0	0.05	0	0.713	0.716
ABCG2	AKT1	9606.ENSP00000237612	9606.ENSP00000451828	0	0	0	0	0.062	0.056	0	0.753	0.763
ACHE	PON1	9606.ENSP00000303211	9606.ENSP00000222381	0	0	0	0	0.062	0	0	0.674	0.681
ACHE	MPO	9606.ENSP00000303211	9606.ENSP00000225275	0	0	0	0	0	0.064	0	0.41	0.424
ACHE	TYR	9606.ENSP00000303211	9606.ENSP00000263321	0	0	0	0	0	0	0	0.531	0.531
ACHE	APP	9606.ENSP00000303211	9606.ENSP00000284981	0	0	0	0	0.091	0.379	0	0.73	0.834
ACHE	F2	9606.ENSP00000303211	9606.ENSP00000308541	0	0	0	0	0.062	0.052	0	0.41	0.429
ACHE	PTGS2	9606.ENSP00000303211	9606.ENSP00000356438	0	0	0	0	0	0.064	0	0.437	0.451
ACHE	AKT1	9606.ENSP00000303211	9606.ENSP00000451828	0	0	0	0	0	0.062	0	0.448	0.46
ACHE	CYP3A4	9606.ENSP00000303211	9606.ENSP00000337915	0	0.003	0	0	0	0	0	0.462	0.462
AHR	AKT1	9606.ENSP00000242057	9606.ENSP00000451828	0	0	0	0	0	0	0	0.447	0.447
AHR	GPR35	9606.ENSP00000242057	9606.ENSP00000411788	0	0	0	0	0	0	0	0.465	0.465
AHR	ESR2	9606.ENSP00000242057	9606.ENSP00000343925	0	0	0	0	0	0.05	0	0.514	0.519
AHR	CYP19A1	9606.ENSP00000242057	9606.ENSP00000379683	0	0	0	0	0	0	0	0.534	0.534
AHR	MMP9	9606.ENSP00000242057	9606.ENSP00000361405	0	0	0	0	0	0	0	0.56	0.56
AHR	RELA	9606.ENSP00000242057	9606.ENSP00000384273	0	0	0	0	0	0.379	0	0.378	0.597

AHR	EGFR	9606.ENSP00000242057	9606.ENSP00000275493	0	0	0	0	0.061	0	0	0.635	0.643
AHR	PTGS2	9606.ENSP00000242057	9606.ENSP00000356438	0	0	0	0	0.098	0	0	0.671	0.69
AHR	AR	9606.ENSP00000242057	9606.ENSP00000363822	0	0	0	0	0	0.384	0	0.589	0.736
AHR	CYP3A4	9606.ENSP00000242057	9606.ENSP00000337915	0	0	0	0	0.061	0	0	0.783	0.788
AHR	NR1I2	9606.ENSP00000242057	9606.ENSP00000336528	0	0	0	0	0.061	0.05	0	0.792	0.798
AHR	CYP1A2	9606.ENSP00000242057	9606.ENSP00000342007	0	0	0	0	0.061	0	0	0.839	0.843
AHR	ESR1	9606.ENSP00000242057	9606.ENSP00000405330	0	0	0	0	0	0.384	0	0.826	0.888
AHR	CYP1B1	9606.ENSP00000242057	9606.ENSP00000478561	0	0	0	0	0.095	0	0	0.939	0.943
AHR	CYP1A1	9606.ENSP00000242057	9606.ENSP00000369050	0	0	0	0	0.061	0	0.9	0.975	0.997
AKR1B1	EGFR	9606.ENSP00000285930	9606.ENSP00000275493	0	0	0	0	0.231	0	0	0.285	0.426
AKR1B1	PRKCB	9606.ENSP00000285930	9606.ENSP00000305355	0	0	0	0	0	0	0	0.44	0.44
AKR1B1	PIM1	9606.ENSP00000285930	9606.ENSP00000362608	0	0	0	0	0	0	0	0.473	0.473
AKR1B1	XDH	9606.ENSP00000285930	9606.ENSP00000368727	0	0	0	0	0	0	0	0.498	0.497
AKR1B1	AKT1	9606.ENSP00000285930	9606.ENSP00000451828	0	0	0	0	0	0	0	0.535	0.535
AKR1B1	PTGS2	9606.ENSP00000285930	9606.ENSP00000356438	0	0	0	0	0.062	0	0	0.653	0.661
AKR1B1	CBR1	9606.ENSP00000285930	9606.ENSP00000290349	0.041	0	0	0	0.063	0	0	0.762	0.768

AKT1	CSNK2A1	9606.ENSP00000451828	9606.ENSP00000217244	0	0	0.223	0.581	0.057	0.406	0	0.377	0.541
AKT1	MMP2	9606.ENSP00000451828	9606.ENSP00000219070	0	0	0	0	0.061	0	0	0.833	0.836
AKT1	MPO	9606.ENSP00000451828	9606.ENSP00000225275	0	0	0	0	0	0.058	0	0.559	0.567
AKT1	HSPB1	9606.ENSP00000451828	9606.ENSP00000248553	0	0	0	0	0	0.698	0	0.864	0.957
AKT1	TYR	9606.ENSP00000451828	9606.ENSP00000263321	0	0	0	0	0	0	0	0.485	0.485
AKT1	EGFR	9606.ENSP00000451828	9606.ENSP00000275493	0	0	0	0.554	0	0.431	0	0.973	0.678
AKT1	APP	9606.ENSP00000451828	9606.ENSP00000284981	0	0	0	0	0.063	0.07	0	0.62	0.64
AKT1	BAX	9606.ENSP00000451828	9606.ENSP00000293288	0	0	0	0	0.061	0.379	0	0.581	0.734
AKT1	MMP3	9606.ENSP00000451828	9606.ENSP00000299855	0	0	0	0	0	0	0	0.585	0.585
AKT1	INSR	9606.ENSP00000451828	9606.ENSP00000303830	0	0	0	0.559	0.052	0.288	0.9	0.677	0.948
AKT1	PRKCB	9606.ENSP00000451828	9606.ENSP00000305355	0	0	0	0.868	0	0.399	0	0.5	0.437
AKT1	F2	9606.ENSP00000451828	9606.ENSP00000308541	0	0	0	0	0	0.05	0	0.662	0.665
AKT1	GSK3B	9606.ENSP00000451828	9606.ENSP00000324806	0	0	0.218	0.576	0.049	0.995	0.9	0.918	0.999
AKT1	NR1I2	9606.ENSP00000451828	9606.ENSP00000336528	0	0	0	0	0	0.085	0	0.408	0.435
AKT1	ESR2	9606.ENSP00000451828	9606.ENSP00000343925	0	0	0	0	0	0.407	0	0.722	0.828
AKT1	E2F1	9606.ENSP00000451828	9606.ENSP00000345571	0	0	0	0	0.091	0.124	0.9	0.481	0.953

AKT1	PIK3CG	9606.ENSP00000451828	9606.ENSP00000352121	0	0	0	0	0.063	0.174	0.9	0.771	0.979
AKT1	TOP1	9606.ENSP00000451828	9606.ENSP00000354522	0	0	0	0	0	0	0	0.452	0.452
AKT1	PARP1	9606.ENSP00000451828	9606.ENSP00000355759	0	0	0	0	0.061	0.057	0	0.783	0.791
AKT1	PTGS2	9606.ENSP00000451828	9606.ENSP00000356438	0	0	0	0	0.062	0.058	0	0.848	0.854
AKT1	MMP9	9606.ENSP00000451828	9606.ENSP00000361405	0	0	0	0	0.061	0	0	0.885	0.887
AKT1	ALOX5	9606.ENSP00000451828	9606.ENSP00000363512	0	0	0	0	0	0	0	0.429	0.429
AKT1	AR	9606.ENSP00000451828	9606.ENSP00000363822	0	0	0	0	0	0.407	0	0.89	0.932
AKT1	CYP19A1	9606.ENSP00000451828	9606.ENSP00000379683	0	0	0	0	0	0.055	0	0.63	0.635
AKT1	RELA	9606.ENSP00000451828	9606.ENSP00000384273	0	0	0	0	0.086	0	0	0.776	0.786
AKT1	CHEK1	9606.ENSP00000451828	9606.ENSP00000388648	0	0	0.241	0.594	0	0.436	0	0.811	0.652
AKT1	ESR1	9606.ENSP00000451828	9606.ENSP00000405330	0	0	0	0	0.055	0.407	0.9	0.867	0.991
AKT1	RXRA	9606.ENSP00000451828	9606.ENSP00000419692	0	0	0	0	0.085	0.085	0.9	0.271	0.93
AKT1	PIK3R1	9606.ENSP00000451828	9606.ENSP00000428056	0	0	0	0	0.062	0.447	0.9	0.835	0.99
AKT1	HSF1	9606.ENSP00000451828	9606.ENSP00000431512	0	0	0	0	0.086	0.098	0	0.572	0.616
AKT1	HIF1A	9606.ENSP00000451828	9606.ENSP00000437955	0	0	0	0	0	0.362	0.9	0.827	0.988
ALOX15	CYP3A4	9606.ENSP00000458832	9606.ENSP00000337915	0	0	0	0	0	0	0.9	0.087	0.904

ALOX15	CYP1A2	9606.ENSP00000458832	9606.ENSP00000342007	0	0	0	0	0	0	0.9	0.057	0.901
ALOX15	PTGS1	9606.ENSP00000458832	9606.ENSP00000354612	0	0	0	0	0.061	0	0.9	0.594	0.958
ALOX15	PTGS2	9606.ENSP00000458832	9606.ENSP00000356438	0	0	0	0	0.061	0	0.9	0.601	0.959
ALOX15	MMP9	9606.ENSP00000458832	9606.ENSP00000361405	0	0	0	0	0.061	0	0	0.561	0.57
ALOX15	ALOX5	9606.ENSP00000458832	9606.ENSP00000363512	0	0	0.298	0.905	0.062	0	0.9	0.891	0.912
ALOX5	MMP2	9606.ENSP00000363512	9606.ENSP00000219070	0	0	0	0	0	0	0	0.501	0.501
ALOX5	MPO	9606.ENSP00000363512	9606.ENSP00000225275	0	0	0	0	0.12	0	0	0.543	0.581
ALOX5	PTGS1	9606.ENSP00000363512	9606.ENSP00000354612	0	0	0	0	0.095	0	0.9	0.728	0.973
ALOX5	PTGS2	9606.ENSP00000363512	9606.ENSP00000356438	0	0	0	0	0.08	0	0.9	0.758	0.975
ALOX5	MMP9	9606.ENSP00000363512	9606.ENSP00000361405	0	0	0	0	0.159	0	0	0.423	0.494
ALOX5	RELA	9606.ENSP00000363512	9606.ENSP00000384273	0	0	0	0	0	0.379	0	0.189	0.474
APEX1	PTGS2	9606.ENSP00000216714	9606.ENSP00000356438	0	0	0	0	0	0	0	0.492	0.492
APEX1	CHEK1	9606.ENSP00000216714	9606.ENSP00000388648	0	0	0	0	0.063	0	0	0.498	0.51
APEX1	TOP2A	9606.ENSP00000216714	9606.ENSP00000411532	0.129	0	0	0	0.098	0	0	0.489	0.564
APEX1	TOP1	9606.ENSP00000216714	9606.ENSP00000354522	0	0	0	0	0.08	0	0	0.549	0.568
APEX1	HIF1A	9606.ENSP00000216714	9606.ENSP00000437955	0	0	0	0	0	0.294	0	0.617	0.718

APEX1	CSNK2A1	9606.ENSP00000216714	9606.ENSP00000217244	0	0	0	0	0.107	0.379	0	0.572	0.742
APEX1	PARP1	9606.ENSP00000216714	9606.ENSP00000355759	0	0	0	0	0.118	0	0.9	0.8	0.98
APP	PON1	9606.ENSP00000284981	9606.ENSP00000222381	0	0	0	0	0	0	0.72	0.322	0.802
APP	EGFR	9606.ENSP00000284981	9606.ENSP00000275493	0	0	0	0	0.146	0.393	0	0.503	0.72
APP	ESR1	9606.ENSP00000284981	9606.ENSP00000405330	0	0	0	0	0	0	0	0.44	0.44
APP	PRSS1	9606.ENSP00000284981	9606.ENSP00000308720	0	0	0	0	0	0.319	0	0.231	0.453
APP	MMP9	9606.ENSP00000284981	9606.ENSP00000361405	0	0	0	0	0.06	0	0	0.474	0.485
APP	PTGS2	9606.ENSP00000284981	9606.ENSP00000356438	0	0	0	0	0.061	0.379	0	0.454	0.654
APP	GSK3B	9606.ENSP00000284981	9606.ENSP00000324806	0	0	0	0	0.057	0.397	0	0.748	0.845
APP	PIK3R1	9606.ENSP00000284981	9606.ENSP00000428056	0	0	0	0	0	0	0.9	0.19	0.915
APP	RELA	9606.ENSP00000284981	9606.ENSP00000384273	0	0	0	0	0	0	0.9	0.408	0.938
APP	F2	9606.ENSP00000284981	9606.ENSP00000308541	0	0	0	0	0	0.132	0.9	0.413	0.944
AR	MMP2	9606.ENSP00000363822	9606.ENSP00000219070	0	0	0	0	0.061	0	0	0.4	0.412
AR	HSPB1	9606.ENSP00000363822	9606.ENSP00000248553	0	0	0	0	0	0	0	0.443	0.443
AR	EGFR	9606.ENSP00000363822	9606.ENSP00000275493	0	0	0	0	0.062	0.402	0	0.678	0.803
AR	INSR	9606.ENSP00000363822	9606.ENSP00000303830	0	0	0	0	0.061	0.085	0	0.522	0.553

AR	PRKCB	9606.ENSP00000363822	9606.ENSP00000305355	0	0	0	0	0	0.348	0	0.318	0.536
AR	MET	9606.ENSP00000363822	9606.ENSP00000317272	0	0	0	0	0	0.085	0	0.738	0.75
AR	GSK3B	9606.ENSP00000363822	9606.ENSP00000324806	0	0	0	0	0	0.407	0.9	0.49	0.967
AR	CYP3A4	9606.ENSP00000363822	9606.ENSP00000337915	0	0	0	0	0	0.085	0	0.433	0.459
AR	E2F1	9606.ENSP00000363822	9606.ENSP00000345571	0	0	0	0	0	0.305	0	0.58	0.696
AR	PARP1	9606.ENSP00000363822	9606.ENSP00000355759	0	0	0	0	0	0	0	0.445	0.445
AR	PTGS2	9606.ENSP00000363822	9606.ENSP00000356438	0	0	0	0	0	0.05	0	0.437	0.442
AR	MMP9	9606.ENSP00000363822	9606.ENSP00000361405	0	0	0	0	0	0	0	0.716	0.717
AR	PIM1	9606.ENSP00000363822	9606.ENSP00000362608	0	0	0	0	0	0	0	0.595	0.595
AR	CYP1A1	9606.ENSP00000363822	9606.ENSP00000369050	0	0	0	0	0	0.085	0	0.417	0.444
AR	ESR1	9606.ENSP00000363822	9606.ENSP00000405330	0	0	0	0.656	0.062	0.379	0	0.936	0.587
AR	HIF1A	9606.ENSP00000363822	9606.ENSP00000437955	0	0	0	0	0	0.413	0	0.378	0.619
AR	RELA	9606.ENSP00000363822	9606.ENSP00000384273	0	0	0	0	0	0.393	0	0.625	0.763
AR	CYP19A1	9606.ENSP00000363822	9606.ENSP00000379683	0	0	0	0	0	0.085	0	0.873	0.879
AR	PIK3R1	9606.ENSP00000363822	9606.ENSP00000428056	0	0	0	0	0	0.153	0.9	0.517	0.955
BAX	GSK3B	9606.ENSP00000293288	9606.ENSP00000324806	0	0	0	0	0.059	0.384	0	0.215	0.505

CBR1	CYP1A1	9606.ENSP00000290349	9606.ENSP00000369050	0	0	0	0	0	0.059	0.9	0.205	0.918
CBR1	CYP3A4	9606.ENSP00000290349	9606.ENSP00000337915	0	0	0	0	0	0.059	0.9	0.29	0.927
CHEK1	CSNK2A1	9606.ENSP00000388648	9606.ENSP00000217244	0	0	0	0.596	0.061	0.402	0	0.263	0.469
CHEK1	EGFR	9606.ENSP00000388648	9606.ENSP00000275493	0	0	0	0	0	0	0	0.531	0.531
CHEK1	E2F1	9606.ENSP00000388648	9606.ENSP00000345571	0	0	0	0	0.16	0	0	0.456	0.524
CHEK1	PIK3CG	9606.ENSP00000388648	9606.ENSP00000352121	0	0	0	0	0	0.262	0	0.259	0.43
CHEK1	TOP1	9606.ENSP00000388648	9606.ENSP00000354522	0	0	0	0	0.062	0.343	0	0.788	0.858
CHEK1	PARP1	9606.ENSP00000388648	9606.ENSP00000355759	0	0	0	0	0.17	0.135	0	0.786	0.833
CHEK1	MMP9	9606.ENSP00000388648	9606.ENSP00000361405	0	0	0	0	0	0	0	0.513	0.513
CHEK1	RELA	9606.ENSP00000388648	9606.ENSP00000384273	0	0	0	0	0	0.294	0	0.561	0.677
CHEK1	ESR1	9606.ENSP00000388648	9606.ENSP00000405330	0	0	0	0	0.061	0	0	0.414	0.426
CHEK1	TOP2A	9606.ENSP00000388648	9606.ENSP00000411532	0	0	0	0	0.803	0.4	0	0.606	0.949
CSNK2A1	HIF1A	9606.ENSP00000217244	9606.ENSP00000437955	0	0	0	0	0	0.379	0	0.111	0.424
CSNK2A1	HSF1	9606.ENSP00000217244	9606.ENSP00000431512	0	0	0	0	0	0.476	0	0.118	0.518
CSNK2A1	TOP1	9606.ENSP00000217244	9606.ENSP00000354522	0	0	0	0	0.064	0.379	0	0.243	0.521

CSNK2A1	RELA	9606.ENSP00000217244	9606.ENSP00000384273	0	0	0	0	0.052	0.305	0	0.511	0.65
CSNK2A1	TOP2A	9606.ENSP00000217244	9606.ENSP00000411532	0	0	0	0	0.084	0.892	0	0.463	0.942
CYP19A1	EGFR	9606.ENSP00000379683	9606.ENSP00000275493	0	0	0	0	0	0	0	0.648	0.648
CYP19A1	CYP3A4	9606.ENSP00000379683	9606.ENSP00000337915	0	0	0.419	0.577	0	0	0.9	0.609	0.937
CYP19A1	ESR2	9606.ENSP00000379683	9606.ENSP00000343925	0	0	0	0	0.062	0.085	0	0.845	0.855
CYP19A1	PTGS2	9606.ENSP00000379683	9606.ENSP00000356438	0	0	0	0	0.05	0	0	0.635	0.639
CYP19A1	CYP1A1	9606.ENSP00000379683	9606.ENSP00000369050	0	0	0.409	0.563	0	0	0.9	0.68	0.941
CYP19A1	CYP1B1	9606.ENSP00000379683	9606.ENSP00000478561	0	0	0.365	0.555	0	0	0	0.679	0.426
CYP19A1	ESR1	9606.ENSP00000379683	9606.ENSP00000405330	0	0	0	0	0	0.085	0	0.96	0.962
CYP1A1	PON1	9606.ENSP00000369050	9606.ENSP00000222381	0	0	0	0	0.055	0	0	0.428	0.436
CYP1A1	NR1I2	9606.ENSP00000369050	9606.ENSP00000336528	0	0	0	0	0.062	0.085	0	0.677	0.699
CYP1A1	CYP3A4	9606.ENSP00000369050	9606.ENSP00000337915	0	0	0.443	0.605	0.129	0	0.9	0.895	0.95

CYP 1A1	CYP1 A2	9606.ENSP0 0000369050	9606.ENSP0 0000342007	0	0	0.448	0.975	0.077	0	0.8	0.92	0.813
CYP 1A1	ESR2	9606.ENSP0 0000369050	9606.ENSP0 0000343925	0	0	0	0	0.064	0.085	0	0.52	0.553
CYP 1A1	PTGS 2	9606.ENSP0 0000369050	9606.ENSP0 0000356438	0	0	0	0	0.055	0	0	0.495	0.502
CYP 1A1	ESR1	9606.ENSP0 0000369050	9606.ENSP0 0000405330	0	0	0	0	0.061	0.085	0	0.675	0.696
CYP 1A1	RXRA	9606.ENSP0 0000369050	9606.ENSP0 0000419692	0	0	0	0	0.051	0.085	0.9	0.212	0.922
CYP 1A1	CYP1 B1	9606.ENSP0 0000369050	9606.ENSP0 0000478561	0	0	0.424	0.893	0.277	0	0.9	0.936	0.935
CYP 1A2	PON 1	9606.ENSP0 0000342007	9606.ENSP0 0000222381	0	0	0	0	0	0	0	0.428	0.428
CYP 1A2	NR1I 2	9606.ENSP0 0000342007	9606.ENSP0 0000336528	0	0	0	0	0	0.085	0	0.677	0.692
CYP 1A2	CYP3 A4	9606.ENSP0 0000342007	9606.ENSP0 0000337915	0	0	0.442	0.604	0.139	0	0.9	0.935	0.952
CYP 1A2	ESR1	9606.ENSP0 0000342007	9606.ENSP0 0000405330	0	0	0	0	0	0.085	0	0.395	0.423
CYP 1A2	CYP1 B1	9606.ENSP0 0000342007	9606.ENSP0 0000478561	0	0	0.418	0.882	0	0	0.8	0.833	0.827
CYP 1A2	XDH	9606.ENSP0 0000342007	9606.ENSP0 0000368727	0	0	0	0	0.061	0	0.9	0.096	0.907
CYP 1B1	MMP 2	9606.ENSP0 0000478561	9606.ENSP0 0000219070	0	0	0	0	0.063	0	0	0.479	0.491
CYP 1B1	NR1I 2	9606.ENSP0 0000478561	9606.ENSP0 0000336528	0	0	0	0	0.061	0.085	0	0.503	0.535
CYP 1B1	CYP3 A4	9606.ENSP0 0000478561	9606.ENSP0 0000337915	0	0	0.403	0.597	0.093	0	0	0.734	0.455
CYP 1B1	ESR2	9606.ENSP0 0000478561	9606.ENSP0 0000343925	0	0	0	0	0.063	0.085	0	0.689	0.711

CYP1B1	PTGS2	9606.ENSP00000478561	9606.ENSP00000356438	0	0	0	0	0.101	0	0	0.656	0.678
CYP1B1	ESR1	9606.ENSP00000478561	9606.ENSP00000405330	0	0	0	0	0.073	0.085	0	0.645	0.673
CYP3A4	PON1	9606.ENSP00000337915	9606.ENSP00000222381	0	0	0	0	0.097	0	0	0.421	0.454
CYP3A4	EGFR	9606.ENSP00000337915	9606.ENSP00000275493	0	0	0	0	0	0	0	0.45	0.45
CYP3A4	F2	9606.ENSP00000337915	9606.ENSP00000308541	0	0	0	0	0.087	0.05	0	0.491	0.519
CYP3A4	NR1I2	9606.ENSP00000337915	9606.ENSP00000336528	0	0	0	0	0.134	0.085	0	0.955	0.962
CYP3A4	RXRA	9606.ENSP00000337915	9606.ENSP00000419692	0	0	0	0	0	0.085	0	0.433	0.459
CYP3A4	ESR1	9606.ENSP00000337915	9606.ENSP00000405330	0	0	0	0	0.049	0.085	0	0.522	0.548
E2F1	EGFR	9606.ENSP00000345571	9606.ENSP00000275493	0	0	0	0	0.061	0	0	0.501	0.511
E2F1	GSK3B	9606.ENSP00000345571	9606.ENSP00000324806	0	0	0	0	0	0.521	0	0.22	0.611
E2F1	TOP2A	9606.ENSP00000345571	9606.ENSP00000411532	0	0	0	0	0.297	0	0	0.212	0.422
E2F1	ESR1	9606.ENSP00000345571	9606.ENSP00000405330	0	0	0	0	0.062	0.379	0	0.413	0.628
E2F1	PARP1	9606.ENSP00000345571	9606.ENSP00000355759	0	0	0	0	0.096	0.472	0	0.525	0.754
EGFR	MMP2	9606.ENSP00000275493	9606.ENSP00000219070	0	0	0	0	0.11	0	0	0.673	0.697
EGFR	HSPB1	9606.ENSP00000275493	9606.ENSP00000248553	0	0	0	0	0.089	0.692	0	0.671	0.899
EGFR	F2	9606.ENSP00000275493	9606.ENSP00000308541	0	0	0	0	0.062	0	0	0.4	0.413

EGFR	PIM1	9606.ENSP00000275493	9606.ENSP00000362608	0	0	0	0.582	0	0.393	0	0.246	0.447
EGFR	RELA	9606.ENSP00000275493	9606.ENSP00000384273	0	0	0	0	0	0	0	0.473	0.473
EGFR	TOP2A	9606.ENSP00000275493	9606.ENSP00000411532	0	0	0	0	0	0	0	0.53	0.53
EGFR	PIK3CG	9606.ENSP00000275493	9606.ENSP00000352121	0	0	0	0	0	0.08	0	0.511	0.53
EGFR	TOP1	9606.ENSP00000275493	9606.ENSP00000354522	0	0	0	0	0	0	0	0.531	0.531
EGFR	PARP1	9606.ENSP00000275493	9606.ENSP00000355759	0	0	0	0	0	0.099	0	0.546	0.573
EGFR	MET	9606.ENSP00000275493	9606.ENSP00000317272	0	0	0	0.58	0.229	0.418	0	0.933	0.715
EGFR	ESR2	9606.ENSP00000275493	9606.ENSP00000343925	0	0	0	0	0	0.077	0	0.739	0.749
EGFR	MMP9	9606.ENSP00000275493	9606.ENSP00000361405	0	0	0	0	0	0	0	0.828	0.828
EGFR	PTGS2	9606.ENSP00000275493	9606.ENSP00000356438	0	0	0	0	0.061	0	0	0.869	0.872
EGFR	ESR1	9606.ENSP00000275493	9606.ENSP00000405330	0	0	0	0	0	0.402	0	0.908	0.943
EGFR	PRKCB	9606.ENSP00000275493	9606.ENSP00000305355	0	0	0	0.553	0.049	0.403	0.9	0.304	0.945
EGFR	MMP3	9606.ENSP00000275493	9606.ENSP00000299855	0	0	0	0	0.076	0	0.9	0.543	0.954
EGFR	PRKCA	9606.ENSP00000275493	9606.ENSP00000408695	0	0	0	0.557	0.049	0.44	0.9	0.676	0.959
EGFR	HIF1A	9606.ENSP00000275493	9606.ENSP00000437955	0	0	0	0	0	0	0.9	0.743	0.973
EGFR	PIK3R1	9606.ENSP00000275493	9606.ENSP00000428056	0	0	0	0	0	0.882	0.9	0.581	0.994

ESR1	MMP2	9606.ENSP00000405330	9606.ENSP00000219070	0	0	0	0	0	0	0	0.532	0.532
ESR1	HSPB1	9606.ENSP00000405330	9606.ENSP00000248553	0	0	0	0	0	0.336	0.9	0.414	0.957
ESR1	MMP3	9606.ENSP00000405330	9606.ENSP00000299855	0	0	0	0	0	0	0	0.426	0.426
ESR1	MET	9606.ENSP00000405330	9606.ENSP00000317272	0	0	0	0	0	0.085	0	0.448	0.473
ESR1	GSK3B	9606.ENSP00000405330	9606.ENSP00000324806	0	0	0	0	0	0.085	0	0.38	0.409
ESR1	ESR2	9606.ENSP00000405330	9606.ENSP00000343925	0	0	0	0.925	0.061	0.379	0.9	0.967	0.941
ESR1	TOP1	9606.ENSP00000405330	9606.ENSP00000354522	0	0	0	0	0.061	0.043	0	0.398	0.411
ESR1	PARP1	9606.ENSP00000405330	9606.ENSP00000355759	0	0	0	0	0	0.342	0	0.652	0.761
ESR1	PTGS2	9606.ENSP00000405330	9606.ENSP00000356438	0	0	0	0	0	0.05	0	0.771	0.773
ESR1	MMP9	9606.ENSP00000405330	9606.ENSP00000361405	0	0	0	0	0	0	0	0.581	0.581
ESR1	RELA	9606.ENSP00000405330	9606.ENSP00000384273	0	0	0	0	0	0.393	0	0.473	0.666
ESR1	TOP2A	9606.ENSP00000405330	9606.ENSP00000411532	0	0	0	0	0.061	0.043	0	0.477	0.488
ESR1	HIF1A	9606.ENSP00000405330	9606.ENSP00000437955	0	0	0	0	0	0.413	0	0.695	0.813
ESR1	PIK3R1	9606.ENSP00000405330	9606.ENSP00000428056	0	0	0	0	0.063	0.404	0.9	0.418	0.963
ESR2	PTGS2	9606.ENSP00000343925	9606.ENSP00000356438	0	0	0	0	0	0.05	0	0.439	0.444
ESR2	HIF1A	9606.ENSP00000343925	9606.ENSP00000437955	0	0	0	0	0	0.095	0	0.532	0.558

ESR2	PIK3R1	9606.ENSP00000343925	9606.ENSP00000428056	0	0	0	0	0	0.081	0.9	0.222	0.922
F2	MPO	9606.ENSP00000308541	9606.ENSP00000225275	0	0	0	0	0	0.057	0	0.479	0.488
F2	TYR	9606.ENSP00000308541	9606.ENSP00000263321	0	0	0	0	0.527	0	0	0.322	0.665
F2	PARP1	9606.ENSP00000308541	9606.ENSP00000355759	0	0	0	0	0	0	0	0.477	0.477
F2	MMP9	9606.ENSP00000308541	9606.ENSP00000361405	0	0	0	0	0	0	0	0.481	0.481
F2	PTGS2	9606.ENSP00000308541	9606.ENSP00000356438	0	0	0	0	0	0.057	0	0.502	0.511
F2	PIK3R1	9606.ENSP00000308541	9606.ENSP00000428056	0	0	0	0	0	0	0.9	0.085	0.904
GSK3B	PRKCB	9606.ENSP00000324806	9606.ENSP00000305355	0	0	0	0.582	0.061	0.529	0	0.355	0.602
GSK3B	TOP2A	9606.ENSP00000324806	9606.ENSP00000411532	0	0	0	0	0.062	0.294	0	0.208	0.429
GSK3B	RELA	9606.ENSP00000324806	9606.ENSP00000384273	0	0	0	0	0	0.294	0	0.244	0.443
GSK3B	PRKCA	9606.ENSP00000324806	9606.ENSP00000408695	0	0	0	0.589	0	0.399	0	0.297	0.465
GSK3B	PTGS2	9606.ENSP00000324806	9606.ENSP00000356438	0	0	0	0	0	0.058	0	0.63	0.637
GSK3B	PIK3R1	9606.ENSP00000324806	9606.ENSP00000428056	0	0	0	0	0.062	0.085	0.8	0.303	0.864
GSK3B	HSF1	9606.ENSP00000324806	9606.ENSP00000431512	0	0	0	0	0.061	0.098	0.9	0.469	0.949
HIF1A	MMP2	9606.ENSP00000437955	9606.ENSP00000219070	0	0	0	0	0	0	0	0.72	0.721
HIF1A	MMP3	9606.ENSP00000437955	9606.ENSP00000299855	0	0	0	0	0	0	0	0.488	0.488

HIF1A	MET	9606.ENSP00000437955	9606.ENSP00000317272	0	0	0	0	0	0.086	0	0.394	0.422
HIF1A	PARP1	9606.ENSP00000437955	9606.ENSP00000355759	0	0	0	0	0	0.379	0	0.519	0.689
HIF1A	PTGS2	9606.ENSP00000437955	9606.ENSP00000356438	0	0	0	0	0.077	0	0	0.533	0.55
HIF1A	MMP9	9606.ENSP00000437955	9606.ENSP00000361405	0	0	0	0	0	0	0	0.55	0.55
HIF1A	RELA	9606.ENSP00000437955	9606.ENSP00000384273	0	0	0	0	0	0.379	0	0.378	0.597
HIF1A	HSF1	9606.ENSP00000437955	9606.ENSP00000431512	0	0	0	0	0	0.063	0	0.439	0.452
HSF1	HSPB1	9606.ENSP00000431512	9606.ENSP00000248553	0	0	0	0	0.09	0.294	0	0.755	0.828
HSF1	INSR	9606.ENSP00000431512	9606.ENSP00000303830	0	0	0	0	0	0.098	0	0.404	0.439
HSF1	PARP1	9606.ENSP00000431512	9606.ENSP00000355759	0	0	0	0	0	0	0	0.579	0.579
HSPB1	MMP2	9606.ENSP00000248553	9606.ENSP00000219070	0	0	0	0	0.086	0	0	0.615	0.633
HSPB1	MMP9	9606.ENSP00000248553	9606.ENSP00000361405	0	0	0	0	0	0	0	0.6	0.6
INSR	PRKCA	9606.ENSP00000303830	9606.ENSP00000408695	0	0	0	0.549	0	0.399	0	0.207	0.446
INSR	MET	9606.ENSP00000303830	9606.ENSP00000317272	0	0	0	0.576	0.061	0.389	0	0.312	0.473
INSR	PIK3CG	9606.ENSP00000303830	9606.ENSP00000352121	0	0	0	0	0.062	0.124	0	0.429	0.49
INSR	PRKCB	9606.ENSP00000303830	9606.ENSP00000305355	0	0	0	0.55	0	0.073	0.8	0.254	0.826
INSR	PIK3R1	9606.ENSP00000303830	9606.ENSP00000428056	0	0	0	0	0.061	0.472	0.9	0.492	0.971

MET	MMP2	9606.ENSP00000317272	9606.ENSP00000219070	0	0	0	0	0.065	0	0	0.511	0.523
MET	PTGS2	9606.ENSP00000317272	9606.ENSP00000356438	0	0	0	0	0.061	0.058	0	0.4	0.422
MET	MMP9	9606.ENSP00000317272	9606.ENSP00000361405	0	0	0	0	0	0	0	0.546	0.546
MET	PARP1	9606.ENSP00000317272	9606.ENSP00000355759	0	0	0	0	0.086	0.057	0	0.555	0.583
MET	PIK3R1	9606.ENSP00000317272	9606.ENSP00000428056	0	0	0	0	0	0.407	0.9	0.262	0.952
MMP2	MPO	9606.ENSP00000219070	9606.ENSP00000225275	0	0	0	0	0	0	0	0.511	0.511
MMP2	RELA	9606.ENSP00000219070	9606.ENSP00000384273	0	0	0	0	0	0.085	0	0.57	0.59
MMP2	PTGS2	9606.ENSP00000219070	9606.ENSP00000356438	0	0	0	0	0.066	0	0	0.696	0.704
MMP2	MMP9	9606.ENSP00000219070	9606.ENSP00000361405	0	0	0	0.929	0.061	0	0.9	0.958	0.908
MMP2	MMP3	9606.ENSP00000219070	9606.ENSP00000299855	0	0	0	0.88	0.181	0	0.9	0.892	0.923
MMP3	MPO	9606.ENSP00000299855	9606.ENSP00000225275	0	0	0	0	0	0	0	0.456	0.456
MMP3	PTGS2	9606.ENSP00000299855	9606.ENSP00000356438	0	0	0	0	0.095	0	0	0.774	0.787
MMP3	PRSS1	9606.ENSP00000299855	9606.ENSP00000308720	0	0	0	0	0	0	0.9	0.063	0.902
MMP3	PRKCA	9606.ENSP00000299855	9606.ENSP00000408695	0	0	0	0	0	0	0.9	0.152	0.911
MMP3	MMP9	9606.ENSP00000299855	9606.ENSP00000361405	0	0	0	0.791	0.518	0	0.9	0.911	0.959
MMP9	MPO	9606.ENSP00000361405	9606.ENSP00000225275	0	0	0	0	0.076	0	0	0.704	0.714

MMP9	PRSS1	9606.ENSP00000361405	9606.ENSP00000308720	0	0	0	0	0	0	0.9	0.2	0.916
MMP9	PTGS2	9606.ENSP00000361405	9606.ENSP00000356438	0	0	0	0	0.097	0	0	0.725	0.741
MMP9	RELA	9606.ENSP00000361405	9606.ENSP00000384273	0	0	0	0	0	0.344	0	0.683	0.783
MPO	PON1	9606.ENSP00000225275	9606.ENSP00000222381	0	0	0	0	0	0	0	0.575	0.575
MPO	XDH	9606.ENSP00000225275	9606.ENSP00000368727	0	0	0	0	0	0	0	0.563	0.563
MPO	PTGS2	9606.ENSP00000225275	9606.ENSP00000356438	0	0	0	0	0.065	0	0	0.691	0.699
MPO	PTGS1	9606.ENSP00000225275	9606.ENSP00000354612	0	0	0	0	0.076	0	0.9	0.408	0.94
NR1I2	RXRA	9606.ENSP00000336528	9606.ENSP00000419692	0	0	0	0.631	0.061	0.89	0	0.639	0.917
PARP1	TOP1	9606.ENSP00000355759	9606.ENSP00000354522	0	0	0	0	0.062	0.379	0	0.81	0.88
PARP1	TOP2A	9606.ENSP00000355759	9606.ENSP00000411532	0	0	0	0	0.175	0	0	0.326	0.42
PARP1	RELA	9606.ENSP00000355759	9606.ENSP00000384273	0	0	0	0	0	0.379	0	0.616	0.751
PIK3CG	PRKCB	9606.ENSP00000352121	9606.ENSP00000305355	0	0	0	0	0.116	0.379	0	0.625	0.776
PIK3CG	PIK3R1	9606.ENSP00000352121	9606.ENSP00000428056	0	0	0	0	0.061	0.446	0.9	0.691	0.981
PIK3R1	RELA	9606.ENSP00000428056	9606.ENSP00000384273	0	0	0	0	0	0	0.9	0.236	0.92
PIK3R1	RXRA	9606.ENSP00000428056	9606.ENSP00000419692	0	0	0	0	0	0.404	0	0.181	0.492
PRKCA	PRKCB	9606.ENSP00000408695	9606.ENSP00000305355	0	0	0	0.981	0	0.39	0.9	0.9	0.937

PRKCA	RELA	9606.ENSP00000408695	9606.ENSP00000384273	0	0	0	0	0	0	0.9	0.214	0.918
PRKCA	TOP2A	9606.ENSP00000408695	9606.ENSP00000411532	0	0	0	0	0	0.563	0	0.088	0.584
PRKCA	RXRA	9606.ENSP00000408695	9606.ENSP00000419692	0	0	0	0	0	0	0.9	0.123	0.908
PRKCB	TYR	9606.ENSP00000305355	9606.ENSP00000263321	0	0	0	0	0.063	0	0.8	0.246	0.846
PRKCB	TOP2A	9606.ENSP00000305355	9606.ENSP00000411532	0	0	0	0	0	0.563	0	0	0.563
PRKCB	RELA	9606.ENSP00000305355	9606.ENSP00000384273	0	0	0	0	0	0	0.9	0.222	0.918
PTGS1	TYR	9606.ENSP00000354612	9606.ENSP00000263321	0	0	0	0	0.051	0	0	0.4	0.406
PTGS1	PTGS2	9606.ENSP00000354612	9606.ENSP00000356438	0	0	0.432	0.967	0.14	0.379	0.8	0.956	0.889
PTGS2	RELA	9606.ENSP00000356438	9606.ENSP00000384273	0	0	0	0	0	0	0	0.558	0.558
RELA	RXRA	9606.ENSP00000384273	9606.ENSP00000419692	0	0	0	0	0.09	0.524	0	0.21	0.628
TOP1	TOP2A	9606.ENSP00000354522	9606.ENSP00000411532	0	0	0	0	0.112	0.925	0.54	0.918	0.997
