# Supplementary material for: Pharmacological Mechanisms Underlying the Hepatoprotective Effects of Ecliptae herba on Hepatocellular Carcinoma
Source: Evid Based Complement Alternat Med. 2021 Jul 16;2021:5591402. doi: 10.1155/2021/5591402 (PMC8302389; doi:10.1155/2021/5591402)
Supplement: Supplementary Materials — Supplementary File S1: a total of 48 chemical ingredients of EH were obtained from TCMSP. Supplementary File S2: detailed information of the targets of 6 active ingredients in EH was extracted from three databases, TCMSP, DGIDB, and SwissTargetPrediction. Supplementary File S3: detailed information on HCC-related targets was extracted from GeneCards and CTD. Supplementary File S4: detailed information on the PPI network of 52 potential therapeutic targets for HCC was obtained from the STRING platform. Supplementary File S5: topological parameters of nodes in the E-H network obtained from Cytoscape. Supplementary File S6: detailed information on GO enrichment analysis obtained from WebGestalt. Supplementary File S7: detailed information on the top 10 GO terms of the GO network in the TCGA RNASeq LIHC database through Network Topology-based Analysis obtained from WebGestalt. Supplementary File S8: detailed information on the top 20 KEGG enrichment pathways obtained from the WebGestalt. Supplementary File S9: detailed information on the C-T-P network obtained from Cytoscape. [file 5591402.f1.zip › 5591402.f1/Supplementary File S5.pdf]

Topological parameters of nodes in E-H network obtained from cytoscape

SUID	AverageShortestPathLength	BetweennessCentrality	ClosenessCentrality	ClusteringCoefficient	Degree	degree.layout	Eccentricity	IsSingleNode	name	NeighborhoodConnectivity	NumberOfDirectedEdges	NumberOfUndirectedEdges	PartnerOfMultiEdgedNodePairs	Radiality	selected	SelfLoops	shared name	Stress	TopologicalCoefficient
131	1.313725	0.206016	0.761194	0.258824	35	35	2	FALSE	AKT1	12.25714	35	0	0	0.921569	FALSE	0	AKT1	2000	0.240336
123	1.431373	0.089246	0.69863	0.337438	29	29	2	FALSE	EGFR	13.7931	29	0	0	0.892157	FALSE	0	EGFR	1148	0.270453
125	1.470588	0.067784	0.68	0.39886	27	27	2	FALSE	ESR1	14.92593	27	0	0	0.882353	FALSE	0	ESR1	1006	0.292665
144	1.509804	0.085964	0.662338	0.346667	25	25	2	FALSE	PTGS2	14.08	25	0	0	0.872549	FALSE	0	PTGS2	1076	0.276078
103	1.588235	0.041309	0.62963	0.424242	22	22	3	FALSE	AR	15.68182	22	0	0	0.852941	FALSE	0	AR	638	0.313636
154	1.647059	0.040523	0.607143	0.45614	19	19	3	FALSE	MMP9	15.42105	19	0	0	0.838235	FALSE	0	MMP9	604	0.308421
156	1.666667	0.027075	0.6	0.450292	19	19	3	FALSE	RELA	16.21053	19	0	0	0.833333	FALSE	0	RELA	404	0.330827

100	1.72549	0.028045	0.579545	0.433333	16	16	3	FALSE	ABCG2	15	16	0	0	0.818627	FALSE	0	ABCG2	382	0.306122
127	1.686275	0.048945	0.593023	0.55	16	16	2	FALSE	AHR	17.0625	16	0	0	0.828431	FALSE	0	AHR	484	0.34
129	1.764706	0.038392	0.566667	0.358333	16	16	3	FALSE	CYP3A4	12.75	16	0	0	0.808824	FALSE	0	CYP3A4	528	0.271277
222	1.784314	0.013269	0.56044	0.552381	15	15	3	FALSE	HIF1A	17.26667	15	0	0	0.803922	FALSE	0	HIF1A	236	0.367376
113	1.843137	0.021508	0.542553	0.494505	14	14	3	FALSE	CYP1A1	13.42857	14	0	0	0.789216	FALSE	0	CYP1A1	312	0.298413
180	1.764706	0.008547	0.566667	0.615385	14	14	3	FALSE	MMP2	17.78571	14	0	0	0.808824	FALSE	0	MMP2	160	0.362974
204	1.862745	0.009831	0.536842	0.494505	14	14	3	FALSE	PARP1	16.07143	14	0	0	0.784314	FALSE	0	PARP1	174	0.36526
196	1.803922	0.016387	0.554348	0.423077	13	13	3	FALSE	GSK3B	15.46154	13	0	0	0.799902	FALSE	0	GSK3B	226	0.322115
21	1.862745	0.01009	0.536842	0.461538	13	13	3	FALSE	PIK3R	16.23077	13	0	0	0.784	FALS	0	PIK3R1	13	0.360684

8									1					314	E			0	
140	1.823529	0.022474	0.548387	0.515152	12	12	3	FALSE	APP	17.33333	12	0	0	0.794118	FALSE	0	APP	358	0.361111
213	1.862745	0.007994	0.536842	0.560606	12	12	3	FALSE	CHEK1	16.16667	12	0	0	0.784314	FALSE	0	CHEK1	146	0.351449
105	1.960784	0.003688	0.51	0.654545	11	11	3	FALSE	CYP1B1	15.18182	11	0	0	0.759804	FALSE	0	CYP1B1	54	0.361472
109	1.901961	0.004546	0.525773	0.6	11	11	3	FALSE	MET	18.36364	11	0	0	0.77451	FALSE	0	MET	98	0.408081
115	1.823529	0.004015	0.548387	0.672727	11	11	3	FALSE	ESR2	19.18182	11	0	0	0.794118	FALSE	0	ESR2	104	0.391466
136	1.862745	0.025752	0.536842	0.418182	11	11	3	FALSE	MPO	13.09091	11	0	0	0.784314	FALSE	0	MPO	274	0.282609
142	1.803922	0.009729	0.554348	0.472727	11	11	3	FALSE	F2	17	11	0	0	0.79902	FALSE	0	F2	178	0.34
169	1.901961	0.012596	0.525773	0.436364	11	11	3	FALSE	PRKCB	14.81818	11	0	0	0.77451	FALSE	0	PRKCB	136	0.329293
24	2	0.007562	0.5	0.490909	11	11	3	FALSE	TOP2	13.45455	11	0	0	0.75	FALS	0	TOP2A	96	0.336364

0									A						E				
117	1.980392	0.016896	0.504951	0.511111	10	10	3	FALSE	CYP1A2	12.3	10	0	0	0.754902	FALSE	0	CYP1A2	172	0.297561
152	1.843137	0.001691	0.542553	0.8	10	10	3	FALSE	CYP19A1	20.6	10	0	0	0.789216	FALSE	0	CYP19A1	60	0.420408
190	1.843137	0.012356	0.542553	0.6	10	10	3	FALSE	MMP3	18.6	10	0	0	0.789216	FALSE	0	MMP3	204	0.379592
107	1.960784	0.001425	0.51	0.722222	9	9	3	FALSE	E2F1	19.88889	9	0	0	0.759804	FALSE	0	E2F1	30	0.45202
119	1.960784	0.002443	0.51	0.694444	9	9	3	FALSE	TOP1	17.77778	9	0	0	0.759804	FALSE	0	TOP1	74	0.40404
101	2.058824	0.003333	0.485714	0.464286	8	8	3	FALSE	APEX1	13.75	8	0	0	0.735294	FALSE	0	APEX1	72	0.34375
121	1.901961	0.003125	0.525773	0.714286	8	8	3	FALSE	NR1I2	15.625	8	0	0	0.77451	FALSE	0	NR1I2	72	0.325521
133	1.921569	0.006362	0.520408	0.535714	8	8	3	FALSE	ACHE	15.125	8	0	0	0.769608	FALSE	0	ACHE	104	0.321809
1	2.078431	0.00274	0.4811	0.5357	8	8	3	FALS	CS	14.5	8	0	0	0.7	FA	0	CS	5	0.37179

78		4	32	14				E	NK2A1					30392	LSE		NK2A1	6	5
192	2.039216	0.00398	0.490385	0.428571	8	8	3	FALSE	INSR	14.125	8	0	0	0.740196	FALSE	0	INSR	48	0.344512
208	1.921569	0.005385	0.520408	0.678571	8	8	3	FALSE	ALOX5	16.75	8	0	0	0.769608	FALSE	0	ALOX5	98	0.356383
349	2.019608	0.004841	0.495146	0.392857	8	8	3	FALSE	PRKCA	13.5	8	0	0	0.745098	FALSE	0	PRKCA	54	0.321429
167	1.921569	0.026153	0.520408	0.285714	7	7	3	FALSE	AKR1B1	15.71429	7	0	0	0.769608	FALSE	0	AKR1B1	306	0.335404
183	1.980392	0.001729	0.504951	0.761905	7	7	3	FALSE	HSPB1	21.85714	7	0	0	0.754902	FALSE	0	HSPB1	34	0.485714
216	1.980392	0.005485	0.504951	0.380952	7	7	3	FALSE	RXRA	16.14286	7	0	0	0.754902	FALSE	0	RXRA	86	0.35873
220	2.098039	0.002754	0.476636	0.380952	7	7	3	FALSE	HSF1	14.28571	7	0	0	0.72549	FALSE	0	HSF1	38	0.3663
134	2.254902	0.003598	0.443478	0.4	6	6	3	FALSE	PON1	11.83333	6	0	0	0.68627	FALSE	0	PON1	44	0.369792

														5					
201	2.078431	9.86E-04	0.481132	0.666667	6	6	3	FALSE	PIK3CG	18	6	0	0	0.730392	FALSE	0	PIK3CG	14	0.439024
224	2.156863	0.005398	0.463636	0.4	6	6	3	FALSE	ALOX15	13.83333	6	0	0	0.710784	FALSE	0	ALOX15	48	0.373874
138	2.098039	0.004273	0.476636	0.4	5	5	3	FALSE	TYR	14	5	0	0	0.72549	FALSE	0	TYR	60	0.345
227	2.294118	0.002258	0.435897	0.5	5	5	3	FALSE	PTGS1	11	5	0	0	0.676471	FALSE	0	PTGS1	24	0.36
111	2.176471	4.74E-04	0.459459	0.666667	4	4	3	FALSE	PIM1	18.5	4	0	0	0.705882	FALSE	0	PIM1	6	0.486842
172	2.45098	0.001756	0.408	0	3	3	3	FALSE	XDH	9.333333	3	0	0	0.637255	FALSE	0	XDH	16	0.378788
176	2.509804	9.05E-04	0.398438	0.333333	3	3	4	FALSE	CBR1	12.33333	3	0	0	0.622549	FALSE	0	CBR1	8	0.545455
249	2.490196	1.31E-04	0.401575	0.666667	3	3	4	FALSE	PRSS1	13.66667	3	0	0	0.627451	FALSE	0	PRSS1	2	0.569444
1	2.235294	0	0.4473	1	2	2	3	FALS	BA	24	2	0	0	0.6	FA	0	BAX	0	0.64864

88			68					E	X					91176	LSE				9
149	2.666667	0	0.375	0	1	1	3	FALSE	GPR35	16	1	0	0	0.583333	FALSE	0	GPR35	0	0
