# Supplementary material for: Pharmacological Mechanisms Underlying the Hepatoprotective Effects of Ecliptae herba on Hepatocellular Carcinoma
Source: Evid Based Complement Alternat Med. 2021 Jul 16;2021:5591402. doi: 10.1155/2021/5591402 (PMC8302389; doi:10.1155/2021/5591402)
Supplement: Supplementary Materials — Supplementary File S1: a total of 48 chemical ingredients of EH were obtained from TCMSP. Supplementary File S2: detailed information of the targets of 6 active ingredients in EH was extracted from three databases, TCMSP, DGIDB, and SwissTargetPrediction. Supplementary File S3: detailed information on HCC-related targets was extracted from GeneCards and CTD. Supplementary File S4: detailed information on the PPI network of 52 potential therapeutic targets for HCC was obtained from the STRING platform. Supplementary File S5: topological parameters of nodes in the E-H network obtained from Cytoscape. Supplementary File S6: detailed information on GO enrichment analysis obtained from WebGestalt. Supplementary File S7: detailed information on the top 10 GO terms of the GO network in the TCGA RNASeq LIHC database through Network Topology-based Analysis obtained from WebGestalt. Supplementary File S8: detailed information on the top 20 KEGG enrichment pathways obtained from the WebGestalt. Supplementary File S9: detailed information on the C-T-P network obtained from Cytoscape. [file 5591402.f1.zip › 5591402.f1/Supplementary File S6.pdf]

Top 10 GO_BP										
geneSet	GO:1901700	GO:1901701	GO:0006915	GO:0010035	GO:0010941	GO:0042981	GO:0043067	GO:0010647	GO:0023056	GO:0008283
description	response to oxygen-containing compound	cellular response to oxygen-containing compound	apoptotic process	response to inorganic substance	regulation of cell death	regulation of apoptotic process	regulation of programmed cell death	positive regulation of cell communication	positive regulation of signaling	cell proliferation
size	1556	1074	1912	536	1649	1517	1531	1733	1739	1986
overlap	32	27	31	19	28	27	27	28	28	29
expect	4.854914197	3.351014041	5.965678627	1.6723869	5.145085803	4.73322933	4.776911076	5.407176287	5.425897036	6.19656786
enrichmentRatio	6.59125964	8.05726257	5.196391213	11.3610075	5.442086113	5.70435069	5.652188112	5.17830352	5.160437033	4.68001007
pValue	0	0	1.11E-16	8.88E-16	2.44E-15	3.22E-15	4.00E-15	8.66E-15	9.33E-15	2.98E-14
FDR	0	0	3.36E-13	2.02E-12	4.44E-12	4.88E-12	5.19E-12	9.42E-12	9.42E-12	2.62E-11
gene symbol	MMP2	MMP2	XDH	MET	XDH	XDH	XDH	XDH	XDH	MMP2
	MET	MET	MET	CYP1A1	MET	MET	MET	MET	MET	XDH
	PRKCB	PRKCB	PRKCB	MMP3	GSK3B	GSK3B	GSK3B	PRKCB	PRKCB	MET
	CYP1A1	AHR	AHR	EGFR	MMP3	EGFR	EGFR	CYP19A1	CYP19A1	CYP1A1
	AHR	GSK3B	GSK3B	PON1	EGFR	PIK3R1	PIK3R1	GSK3B	GSK3B	AHR
	GSK3B	MMP3	EGFR	ALOX15	PIK3R1	CSNK2A1	CSNK2A1	EGFR	EGFR	EGFR
	MMP3	EGFR	PIK3R1	MPO	CSNK2A1	MPO	MPO	PIK3R1	PIK3R1	TYR
	EGFR	PIK3R1	CSNK2A1	CYP1A2	MPO	PIK3CG	PIK3CG	CSNK2A1	CSNK2A1	CSNK2A1
	TYR	MPO	CHEK1	RELA	PIK3CG	TOP2A	TOP2A	ALOX15	ALOX15	RXRA
	PIK3R1	PIK3CG	MPO	HIF1A	TOP2A	RELA	RELA	PIK3CG	PIK3CG	ACHE
	RXRA	RELA	PIK3CG	AKT1	RELA	HIF1A	HIF1A	RELA	RELA	PIK3CG
	PON1	HIF1A	TOP2A	APP	HIF1A	INSR	INSR	HIF1A	HIF1A	RELA
	MPO	INSR	RELA	PTGS2	INSR	AKT1	AKT1	INSR	INSR	HIF1A
	CYP1A2	AKT1	HIF1A	APEX1	AKT1	AR	AR	AKT1	AKT1	INSR
	PIK3CG	AR	INSR	AKR1B1	AR	HSPB1	HSPB1	AR	AR	AKT1
	RELA	APP	AKT1	CYP1B1	HSPB1	PTGS2	PTGS2	APP	APP	AR

	HIF1A	PTGS2	AR	PARP1	PTGS2	ESR1	ESR1	F2	F2	APP
	INSR	ESR1	APP	HSF1	ESR1	APEX1	APEX1	PTGS2	PTGS2	F2
	AKT1	APEX1	HSPB1	MMP9	APEX1	AKR1B1	AKR1B1	ESR1	ESR1	PTGS2
	AR	AKR1B1	PTGS2		AKR1B1	CYP1B1	CYP1B1	GPR35	GPR35	ESR1
	APP	CYP1B1	ESR1		CYP1B1	E2F1	E2F1	AKR1B1	AKR1B1	AKR1B1
	PTGS2	E2F1	APEX1		E2F1	PIM1	PIM1	CYP1B1	CYP1B1	CYP1B1
	ESR1	PIM1	AKR1B1		PIM1	PARP1	PARP1	E2F1	E2F1	E2F1
	APEX1	PARP1	CYP1B1		PARP1	HSF1	HSF1	PARP1	PARP1	PIM1
	AKR1B1	HSF1	E2F1		HSF1	BAX	BAX	HSF1	HSF1	HSF1
	CYP1B1	PRKCA	PIM1		BAX	PRKCA	PRKCA	BAX	BAX	PTGS1
	E2F1	MMP9	PARP1		PRKCA	MMP9	MMP9	PRKCA	PRKCA	BAX
	PIM1		HSF1		MMP9			MMP9	MMP9	PRKCA
	PARP1		BAX							MMP9
	HSF1		PRKCA							
	PRKCA		MMP9							
	MMP9									
Top 10 GO_CC										
geneSet	GO:0000228	GO:0005667	GO:0044454	GO:0005694	GO:0044427	GO:0097651	GO:0000785	GO:0048471	GO:0043235	GO:0005783
description	nuclear chromosome	transcription factor complex	nuclear chromosome part	chromosome	chromosomal part	phosphatidylinositol 3-kinase complex, class I	chromatin	perinuclear region of cytoplasm	receptor complex	endoplasmic reticulum
size	573	355	535	1014	886	5	509	684	396	1861
overlap	11	8	9	12	11	2	8	9	7	15
expect	1.660775176	1.028927029	1.550636508	2.9389634	2.567969993	0.01449193	1.475278472	1.982496022	1.147760855	5.39389634
enrichmentRatio	6.623413065	7.775089754	5.804068169	4.08307228	4.283539149	138.007843	5.422705035	4.539731682	6.098831452	2.78092107
pValue	5.79E-07	7.58E-06	2.02E-05	2.46E-05	3.70E-05	8.19E-05	9.89E-05	1.35E-04	1.37E-04	1.74E-04

FDR	6.80E-04	0.004452046	0.007240347	0.00724035	0.008698873	0.01603994	0.016599301	0.017848066	0.017848066	0.02039898
gene symbol	CSNK2A1	AHR	CSNK2A1	CSNK2A1	CSNK2A1	PIK3R1	CSNK2A1	EGFR	MET	XDH
	RXRA	RXRA	RXRA	RXRA	RXRA	PIK3CG	RXRA	TYR	AHR	CYP1A1
	CHEK1	RELA	RELA	CHEK1	CHEK1		CHEK1	PIK3R1	EGFR	CYP19A1
	TOP2A	HIF1A	AR	TOP2A	RELA		RELA	ACHE	RXRA	EGFR
	RELA	NR1I2	ESR1	RELA	AR		AR	APP	ABCG2	PIK3R1
	AR	APEX1	APEX1	AR	ESR1		ESR1	APEX1	INSR	CYP1A2
	ESR1	E2F1	E2F1	ESR1	APEX1		E2F1	AKR1B1	APP	CYP3A4
	APEX1	PARP1	PARP1	APEX1	E2F1		HSF1	HSF1		APP
	E2F1		TOP1	E2F1	PARP1			PRKCA		F2
	PARP1			PARP1	HSF1					PTGS2
	TOP1			HSF1	TOP1					APEX1
				TOP1						CYP1B1
										PTGS1
										BAX
										PRKCA
Top 10 GO_MF										
geneSet	GO:0046914	GO:0042802	GO:0046983	GO:0016491	GO:0020037	GO:0008134	GO:0004879	GO:0098531	GO:0046906	GO:0005506
description	transition metal ion binding	identical protein binding	protein dimerization activity	oxidoreductase activity	heme binding	transcription factor binding	nuclear receptor activity	transcription factor activity, direct ligand regulated sequence-specific DNA binding	tetrapyrrole binding	iron ion binding
size	1058	1696	1270	731	128	638	48	48	138	148
overlap	22	22	19	15	8	14	6	6	8	8
expect	3.300101973	5.29014456	3.96137004	2.28012717	0.39925619	1.99004259	0.14972107	0.149721073	0.43044808	0.4616399

		2	4		3		3		3	7
enrichmentRa<br>tio	6.666460666	4.15867652<br>4	4.79632041<br>2	6.57858045	20.0372596<br>2	7.03502532	40.0745192<br>3	40.07451923	18.5852842<br>8	17.329521<br>8
pValue	1.58E-13	1.69E-09	3.35E-09	3.64E-09	5.49E-09	5.78E-09	7.59E-09	7.59E-09	9.95E-09	1.73E-08
FDR	2.97E-10	1.59E-06	1.71E-06	1.71E-06	1.78E-06	1.78E-06	1.78E-06	1.78E-06	2.08E-06	3.24E-06
gene symbol	MMP2	XDH	XDH	XDH	CYP1A1	PRKCB	AHR	AHR	CYP1A1	XDH
	XDH	MET	AHR	CYP1A1	CYP19A1	AHR	RXRA	RXRA	CYP19A1	CYP1A1
	PRKCB	AHR	EGFR	CYP19A1	MPO	GSK3B	NR1I2	NR1I2	MPO	CYP19A1
	CYP1A1	EGFR	TYR	TYR	CYP1A2	PIK3R1	AR	AR	CYP1A2	ALOX15
	CYP19A1	TYR	PIK3R1	ALOX15	CYP3A4	RXRA	ESR1	ESR1	CYP3A4	CYP1A2
	MMP3	CSNK2A1	RXRA	MPO	PTGS2	RELA	ESR2	ESR2	PTGS2	CYP3A4
	TYR	PON1	PON1	CYP1A2	CYP1B1	HIF1A			CYP1B1	CYP1B1
	RXRA	ACHE	ACHE	CYP3A4	PTGS1	NR1I2			PTGS1	ALOX5
	ALOX15	ABCG2	ABCG2	CBR1		AR				
	CYP1A2	PIK3CG	TOP2A	PTGS2		ESR1				
	NR1I2	TOP2A	RELA	APEX1		APEX1				
	AR	RELA	HIF1A	AKR1B1		E2F1				
	CYP3A4	AKT1	AKT1	CYP1B1		PIM1				
	APP	APP	AR	ALOX5		PARP1				
	ESR1	HSPB1	HSPB1	PTGS1						
	ESR2	PTGS2	PTGS2							
	CYP1B1	ESR1	E2F1							
	ALOX5	ESR2	HSF1							
	PIM1	PARP1	BAX							
	PARP1	HSF1								
	PRKCA	BAX								
	MMP9	MMP9								
