# Supplementary material for: Pharmacological Mechanisms Underlying the Hepatoprotective Effects of Ecliptae herba on Hepatocellular Carcinoma
Source: Evid Based Complement Alternat Med. 2021 Jul 16;2021:5591402. doi: 10.1155/2021/5591402 (PMC8302389; doi:10.1155/2021/5591402)
Supplement: Supplementary Materials — Supplementary File S1: a total of 48 chemical ingredients of EH were obtained from TCMSP. Supplementary File S2: detailed information of the targets of 6 active ingredients in EH was extracted from three databases, TCMSP, DGIDB, and SwissTargetPrediction. Supplementary File S3: detailed information on HCC-related targets was extracted from GeneCards and CTD. Supplementary File S4: detailed information on the PPI network of 52 potential therapeutic targets for HCC was obtained from the STRING platform. Supplementary File S5: topological parameters of nodes in the E-H network obtained from Cytoscape. Supplementary File S6: detailed information on GO enrichment analysis obtained from WebGestalt. Supplementary File S7: detailed information on the top 10 GO terms of the GO network in the TCGA RNASeq LIHC database through Network Topology-based Analysis obtained from WebGestalt. Supplementary File S8: detailed information on the top 20 KEGG enrichment pathways obtained from the WebGestalt. Supplementary File S9: detailed information on the C-T-P network obtained from Cytoscape. [file 5591402.f1.zip › 5591402.f1/Supplementary File S7.pdf]

Detailed information of top 10 GO terms of GO network in the TCGA RNASeq LIHC database through Network Topology-based Analysis								
**GO ID**	**description**	**size**	**overlap**	**interestGene**	**expect**	**enrichmentRatio**	**pValue**	**FDR**
GO:0042759	long-chain fatty acid biosynthetic process	21	5	ALOX5|1;ALOX15|1;CYP1A1|1;CYP1A2|1;CYP3A4|1	0.055301	90.41408	1.57E-09	2.28E-05
GO:0001676	long-chain fatty acid metabolic process	84	6	ALOX5|1;ALOX15|1;CYP1A1|1;CYP1A2|1;CYP3A4|1;PTGS1|1	0.221204	27.12422	5.85E-08	2.84E-04
GO:0006629	lipid metabolic process	901	13	ACHE|1;ALOX5|1;ALOX15|1;CYP1A1|1;CYP1A2|1;CYP3A4|1;CYP19A1|1;MET|1;PTGS1|1;HSD17B11|0;GAL3ST4|0;CYP27C1|0;TNFAIP8L3|0	2.372681	5.479033	5.89E-08	2.84E-04
GO:0008610	lipid biosynthetic process	457	10	ACHE|1;ALOX5|1;ALOX15|1;CYP1A1|1;CYP1A2|1;CYP3A4|1;CYP19A1|1;PTGS1|1;HSD17B11|0;GAL3ST4|0	1.203458	8.30939	8.63E-08	3.12E-04
GO:0006706	steroid catabolic process	19	4	CYP1A2|1;CYP3A4|1;CYP19A1|1;HSD17B11|0	0.050034	79.94508	1.38E-07	4.00E-04
GO:0006633	fatty acid biosynthetic process	110	6	ALOX5|1;ALOX15|1;CYP1A1|1;CYP1A2|1;CYP3A4|1;PTGS1|1	0.289673	20.71304	2.95E-07	7.12E-04
GO:0044255	cellular lipid metabolic process	684	11	ACHE|1;ALOX5|1;ALOX15|1;CYP1A1|1;CYP1A2|1;CYP3A4|1;MET|1;PTGS1|1;GAL3ST4|0;CYP27C1|0;TNFAIP8L3|0	1.801237	6.106916	3.55E-07	7.33E-04
GO:0042445	hormone metabolic process	137	6	ACHE|1;CYP1A1|1;CYP3A4|1;CYP19A1|1;HSD17B11|0;CYP27C1|0	0.360774	16.63091	1.08E-06	0.001958794
GO:0006690	icosanoid metabolic process	77	5	ALOX5|1;ALOX15|1;CYP1A1|1;CYP1A2|1;PTGS1|1	0.202771	24.65839	1.39E-06	0.00223392
GO:0034754	cellular hormone metabolic process	84	5	CYP1A1|1;CYP3A4|1;CYP19A1|1;HSD17B11|0;CYP27C1|0	0.221204	22.60352	2.14E-06	0.003000318
