# Supplementary material for: Pharmacological Mechanisms Underlying the Hepatoprotective Effects of Ecliptae herba on Hepatocellular Carcinoma
Source: Evid Based Complement Alternat Med. 2021 Jul 16;2021:5591402. doi: 10.1155/2021/5591402 (PMC8302389; doi:10.1155/2021/5591402)
Supplement: Supplementary Materials — Supplementary File S1: a total of 48 chemical ingredients of EH were obtained from TCMSP. Supplementary File S2: detailed information of the targets of 6 active ingredients in EH was extracted from three databases, TCMSP, DGIDB, and SwissTargetPrediction. Supplementary File S3: detailed information on HCC-related targets was extracted from GeneCards and CTD. Supplementary File S4: detailed information on the PPI network of 52 potential therapeutic targets for HCC was obtained from the STRING platform. Supplementary File S5: topological parameters of nodes in the E-H network obtained from Cytoscape. Supplementary File S6: detailed information on GO enrichment analysis obtained from WebGestalt. Supplementary File S7: detailed information on the top 10 GO terms of the GO network in the TCGA RNASeq LIHC database through Network Topology-based Analysis obtained from WebGestalt. Supplementary File S8: detailed information on the top 20 KEGG enrichment pathways obtained from the WebGestalt. Supplementary File S9: detailed information on the C-T-P network obtained from Cytoscape. [file 5591402.f1.zip › 5591402.f1/Supplementary File S8.pdf]

Top 20 KEGG enrichment pathways

geneSet	hsa05200	hsa05223	hsa05215	hsa01522	hsa01521	hsa04933	hsa04066	hsa05214	hsa05205	hsa04913
description	Pathways in cancer	Non-small cell lung cancer	Prostate cancer	Endocrine resistance	EGFR tyrosine kinase inhibitor resistance	AGE-RAGE signaling pathway in diabetic complications	HIF-1 signaling pathway	Glioma	Proteoglycans in cancer	Ovarian steroidogenesis
size	526	66	97	98	79	99	100	71	201	49
overlap	19	8	9	9	8	8	8	7	10	6
expect	3.4507966	0.432989691	0.6363636	0.64292409	0.518275539	0.649484536	0.656044986	0.465792	1.31865042	0.321462043
enrichmentRatio	5.505975	18.47619048	14.142857	13.9985423	15.4358047	12.31746032	12.19428571	15.02817	7.58351102	18.66472303
pValue	2.39E-10	8.14E-09	9.66E-09	1.06E-08	3.47E-08	2.06E-07	2.23E-07	3.25E-07	5.24E-07	6.57E-07
FDR	7.80E-08	8.62E-07	8.62E-07	8.62E-07	2.26E-06	1.04E-05	1.04E-05	1.32E-05	1.90E-05	2.09E-05
Gene symbol	MMP2	PRKCB	GSK3B	MMP2	MET	MMP2	PRKCB	PRKCB	MMP2	CYP1A1
	MET	EGFR	MMP3	EGFR	PRKCB	PRKCB	EGFR	EGFR	MET	CYP19A1
	PRKCB	PIK3R1	EGFR	PIK3R1	GSK3B	PIK3R1	PIK3R1	PIK3R1	PRKCB	INSR
	GSK3B	RXRA	PIK3R1	AKT1	EGFR	RELA	RELA	AKT1	EGFR	PTGS2
	EGFR	AKT1	RELA	ESR1	PIK3R1	AKT1	HIF1A	E2F1	PIK3R1	CYP1B1
	PIK3R1	E2F1	AKT1	ESR2	AKT1	PIM1	INSR	BAX	HIF1A	ALOX5
	RXRA	BAX	AR	E2F1	BAX	BAX	AKT1	PRKCA	AKT1	
	RELA	PRKCA	E2F1	BAX	PRKCA	PRKCA	PRKCA		ESR1	
	HIF1A		MMP9	MMP9					PRKCA	
	AKT1								MMP9	
	AR									
	PTGS2									
	ESR1									
	ESR2									
	E2F1									

	PIM1									
	BAX									
	PRKCA									
	MMP9									
geneSet	hsa04919	hsa05225	hsa05163	hsa04370	hsa05222	hsa05167	hsa05161	hsa05224	hsa05226	hsa04917
description	Thyroid hormone signaling pathway	Hepatocellular carcinoma	Human cytomegalovirus infection	VEGF signaling pathway	Small cell lung cancer	Kaposi sarcoma-associated herpesvirus infection	Hepatitis B	Breast cancer	Gastric cancer	Prolactin signaling pathway
size	116	168	225	59	93	186	144	147	149	70
overlap	8	9	10	6	7	9	8	8	8	6
expect	0.7610122	1.102155576	1.4761012	0.3870665 4	0.61012183 7	1.220243674	0.94470478	0.964386	0.97750703	0.45923149
enrichmentRatio	10.512315	8.165816327	6.7746032	15.5012107	11.47311828	7.375576037	8.468253968	8.295432	8.18408437	13.06530612
pValue	7.04E-07	1.13E-06	1.48E-06	2.01E-06	2.07E-06	2.64E-06	3.63E-06	4.24E-06	4.69E-06	5.55E-06
FDR	2.09E-05	3.07E-05	3.71E-05	4.51E-05	4.51E-05	5.38E-05	6.97E-05	7.68E-05	8.05E-05	9.05E-05
Gene symbol	PRKCB	MET	PRKCB	PRKCB	PIK3R1	GSK3B	PRKCB	GSK3B	MET	GSK3B
	GSK3B	PRKCB	GSK3B	PIK3R1	RXRA	PIK3R1	PIK3R1	EGFR	GSK3B	PIK3R1
	PIK3R1	GSK3B	EGFR	AKT1	RELA	PIK3CG	RELA	PIK3R1	EGFR	RELA
	RXRA	EGFR	PIK3R1	HSPB1	AKT1	RELA	AKT1	AKT1	PIK3R1	AKT1
	HIF1A	PIK3R1	RELA	PTGS2	PTGS2	HIF1A	E2F1	ESR1	RXRA	ESR1
	AKT1	AKT1	AKT1	PRKCA	E2F1	AKT1	BAX	ESR2	AKT1	ESR2
	ESR1	E2F1	PTGS2		BAX	PTGS2	PRKCA	E2F1	E2F1	
	PRKCA	BAX	E2F1			E2F1	MMP9	BAX	BAX	
		PRKCA	BAX			BAX				
			PRKCA							
