# Supplementary material for: Pharmacological Mechanisms Underlying the Hepatoprotective Effects of Ecliptae herba on Hepatocellular Carcinoma
Source: Evid Based Complement Alternat Med. 2021 Jul 16;2021:5591402. doi: 10.1155/2021/5591402 (PMC8302389; doi:10.1155/2021/5591402)
Supplement: Supplementary Materials — Supplementary File S1: a total of 48 chemical ingredients of EH were obtained from TCMSP. Supplementary File S2: detailed information of the targets of 6 active ingredients in EH was extracted from three databases, TCMSP, DGIDB, and SwissTargetPrediction. Supplementary File S3: detailed information on HCC-related targets was extracted from GeneCards and CTD. Supplementary File S4: detailed information on the PPI network of 52 potential therapeutic targets for HCC was obtained from the STRING platform. Supplementary File S5: topological parameters of nodes in the E-H network obtained from Cytoscape. Supplementary File S6: detailed information on GO enrichment analysis obtained from WebGestalt. Supplementary File S7: detailed information on the top 10 GO terms of the GO network in the TCGA RNASeq LIHC database through Network Topology-based Analysis obtained from WebGestalt. Supplementary File S8: detailed information on the top 20 KEGG enrichment pathways obtained from the WebGestalt. Supplementary File S9: detailed information on the C-T-P network obtained from Cytoscape. [file 5591402.f1.zip › 5591402.f1/Supplementary File S9.pdf]

Detailed information of C-T-P network obtained from Cytoscape

SUID	AverageShortestPathLength	BetweennessCentrality	ClosenessCentrality	ClusteringCoefficient	Degree	Eccentricity	IsSingleNode	name	NeighborhoodConnectivity	NumberOfDirectedEdges	NumberOfUndirectedEdges	PartnerOfMultiEdgedNodePairs	Radiality	selected	SelfLoops	shared name	Stress	TopologicalCoefficient	Type
309	1.644737	0.568954	0.608	0	40	4	FALSE	quercetin	4.725	40	0	0	0.8925544	FALSE	0	quercetin	29718	0.15520833	compound
91	1.868421	0.088409	0.535211	0	21	4	FALSE	AKT1	10.38095	21	0	0	0.8552263	FALSE	0	AKT1	105882	0.19959473	gene
83	1.947368	0.068371	0.513514	0	20	4	FALSE	PIK3R1	10.25	20	0	0	0.8421105	FALSE	0	PIK3R1	8488	0.20555556	gene
72	2.171053	0.091785	0.460606	0	19	3	FALSE	Pathways in cancer	10.10526	19	0	0	0.8048825	FALSE	0	Pathways in cancer	13002	0.36421053	pathway
81	2.052632	0.04523	0.487179	0	14	4	FALSE	EGFR	11.85714	14	0	0	0.8245561	FALSE	0	EGFR	5646	0.23100304	gene
105	2.105263	0.035486	0.475	0	14	4	FALSE	BAX	11.28571	14	0	0	0.8157789	FALSE	0	BAX	4510	0.22857143	gene
7	2.131579	0.0313	0.469	0	13	4	FAL	PRKCB	11.4615	13	0	0	0.8	FA	0	PRKCB	3	0.2324	gen

7		01	136				SE		4				11404	LSE			848	7863	e
101	2.131579	0.032655	0.469136	0	13	4	FALSE	E2F1	11.61538	13	0	0	0.811404	FALSE	0	E2F1	41886	0.23589744	gene
107	2.131579	0.031301	0.469136	0	13	4	FALSE	PRKCA	11.46154	13	0	0	0.811404	FALSE	0	PRKCA	38448	0.23247863	gene
290	2.486842	0.079007	0.402116	0	13	5	FALSE	luteolin	6.538462	13	0	0	0.752193	FALSE	0	luteolin	7012	0.25174825	compound
79	2.789474	0.031272	0.358491	0	12	4	FALSE	GSK3B	8.416667	12	0	0	0.701754	FALSE	0	GSK3B	4146	0.3531746	gene
87	2.131579	0.033731	0.469136	0	11	4	FALSE	RELA	12.45455	11	0	0	0.811404	FALSE	0	RELA	3888	0.24371373	gene
177	2.460526	0.015774	0.406417	0	10	4	FALSE	Proteoglycans in cancer	11.6	10	0	0	0.756579	FALSE	0	Proteoglycans in cancer	2822	0.46086957	pathway
218	2.513158	0.010061	0.397906	0	10	5	FALSE	Human cytomegalovirus infection	13.8	10	0	0	0.747807	FALSE	0	Human cytomegalovirus infection	2306	0.55652174	pathway
12	2.565789	0.015458	0.389744	0	9	5	FALSE	Prostate cancer	11.55556	9	0	0	0.739	FALS	0	Prostate cancer	22	0.47979798	pathwa

0													035	E			02		y
131	2.486842	0.014027	0.402116	0	9	4	FALSE	Endocrine resistance	12.44444	9	0	0	0.752193	FALSE	0	Endocrine resistance	2684	0.49758454	pathway
208	2.565789	0.007568	0.389744	0	9	5	FALSE	Hepatocellular carcinoma	14	9	0	0	0.739035	FALSE	0	Hepatocellular carcinoma	1838	0.59090909	pathway
245	2.539474	0.013816	0.393782	0	9	5	FALSE	Kaposi sarcoma-associated herpesvirus infection	11.77778	9	0	0	0.743421	FALSE	0	Kaposi sarcoma-associated herpesvirus infection	2154	0.46859903	pathway
97	2.894737	0.031179	0.345455	0	8	4	FALSE	ESR1	8.5	8	0	0	0.684211	FALSE	0	ESR1	4454	0.35714286	gene
109	2.210526	0.026591	0.452381	0	8	4	FALSE	MMP9	13.875	8	0	0	0.798246	FALSE	0	MMP9	2862	0.27393617	gene
111	2.618421	0.009511	0.38191	0	8	5	FALSE	Non-small cell lung cancer	14.25	8	0	0	0.730263	FALSE	0	Non-small cell lung cancer	1754	0.63095238	pathway
141	2.592105	0.006269	0.385787	0	8	5	FALSE	EGFR tyrosine kinase	14.125	8	0	0	0.73464	FALSE	0	EGFR tyrosine kinase	153	0.59659091	pathway

								inhibitor resistance					9			inhibitor resistance	8		
150	2.644737	0.00534	0.378109	0	8	5	FALSE	AGE-RAGE signaling pathway in diabetic complications	12.75	8	0	0	0.725877	FALSE	0	AGE-RAGE signaling pathway in diabetic complications	714	0.55952381	pathway
159	2.644737	0.007912	0.378109	0	8	5	FALSE	HIF-1 signaling pathway	12.625	8	0	0	0.725877	FALSE	0	HIF-1 signaling pathway	1114	0.55357143	pathway
199	2.486842	0.015452	0.402116	0	8	4	FALSE	Thyroid hormone signaling pathway	12.375	8	0	0	0.752193	FALSE	0	Thyroid hormone signaling pathway	2842	0.47395833	pathway
256	2.644737	0.003552	0.378109	0	8	5	FALSE	Hepatitis B	14.125	8	0	0	0.725877	FALSE	0	Hepatitis B	690	0.625	pathway
265	2.513158	0.011267	0.397906	0	8	4	FALSE	Breast cancer	13.625	8	0	0	0.747807	FALSE	0	Breast cancer	2618	0.54891304	pathway
274	2.539474	0.012158	0.393782	0	8	5	FALSE	Gastric cancer	13.25	8	0	0	0.743421	FALSE	0	Gastric cancer	2364	0.5326087	pathway
73	2.236842	0.023106	0.447059	0	7	4	FALSE	MMP2	14.57143	7	0	0	0.793	FALS	0	MMP2	24	0.2887538	gene

													86	E			16		
95	2.684211	0.017926	0.372549	0	7	4	FALSE	PTGS2	10	7	0	0	0.719298	FALSE	0	PTGS2	1942	0.3	gene
99	2.210526	0.083675	0.452381	0	7	4	FALSE	ESR2	12.85714	7	0	0	0.798246	FALSE	0	ESR2	5520	0.24702381	gene
169	2.697368	0.001694	0.370732	0	7	5	FALSE	Glioma	15.42857	7	0	0	0.717105	FALSE	0	Glioma	386	0.72142857	pathway
237	2.618421	0.010011	0.38191	0	7	5	FALSE	Small cell lung cancer	13.14286	7	0	0	0.730263	FALSE	0	Small cell lung cancer	1720	0.55194805	pathway
75	2.894737	0.005177	0.345455	0	6	4	FALSE	MET	11.16667	6	0	0	0.684211	FALSE	0	MET	758	0.44202899	gene
85	3	0.030918	0.333333	0	6	4	FALSE	RXRA	8.666667	6	0	0	0.666667	FALSE	0	RXRA	4280	0.40350877	gene
89	2.315789	0.011311	0.431818	0	6	4	FALSE	HIF1A	15.66667	6	0	0	0.780702	FALSE	0	HIF1A	1512	0.32592593	gene
188	3.039474	0.006331	0.329004	0	6	5	FALSE	Ovarian steroidogenesis	3	6	0	0	0.660608	FALSE	0	Ovarian steroidogenesis	420	0.25	pathway

													8					
229	2.697368	0.006411	0.370732	0	6	5	FALSE	VEGF signaling pathway	12.66667	6	0	0	0.717105	FALSE	0	VEGF signaling pathway	872	0.55555556	pathway
283	2.565789	0.007768	0.389744	0	6	4	FALSE	Prolactin signaling pathway	13.16667	6	0	0	0.739035	FALSE	0	Prolactin signaling pathway	1778	0.52898551	pathway
366	2.881579	0.027598	0.347032	0	4	5	FALSE	wedelolactone	7	4	0	0	0.686404	FALSE	0	wedelolactone	2586	0.4	compound
380	3.039474	0.048028	0.329004	0	4	5	FALSE	3'-O-Methylorobol	4.5	4	0	0	0.660088	FALSE	0	3'-O-Methylorobol	3958	0.38888889	compound
93	2.947368	0.001693	0.339286	0	3	4	FALSE	AR	13.66667	3	0	0	0.675439	FALSE	0	AR	306	0.52777778	gene
103	2.394737	0.003552	0.417582	0	3	4	FALSE	PIM1	22.33333	3	0	0	0.767544	FALSE	0	PIM1	550	0.47407407	gene
165	2.552632	0.005189	0.391753	0	3	5	FALSE	INSR	18	3	0	0	0.741228	FALSE	0	INSR	514	0.425	gene
372	2.855263	0.001149	0.35023	0	3	5	FALSE	demethylwedelolactone	9	3	0	0	0.690789	FALSE	0	demethylwedelolactone	226	0.47058824	compound

122	2.552632	0.001756	0.391753	0	2	5	FALSE	MMP3	24.5	2	0	0	0.741228	FALSE	0	MMP3	184	0.57317073	gene
189	2.578947	0.002931	0.387755	0	2	5	FALSE	CYP1A1	23	2	0	0	0.736842	FALSE	0	CYP1A1	294	0.55	gene
191	2.578947	0.002931	0.387755	0	2	5	FALSE	CYP19A1	23	2	0	0	0.736842	FALSE	0	CYP19A1	294	0.55	gene
195	2.578947	0.002931	0.387755	0	2	5	FALSE	CYP1B1	23	2	0	0	0.736842	FALSE	0	CYP1B1	294	0.55	gene
197	2.578947	0.002931	0.387755	0	2	5	FALSE	ALOX5	23	2	0	0	0.736842	FALSE	0	ALOX5	294	0.55	gene
233	2.578947	0.001996	0.387755	0	2	5	FALSE	HSPB1	23	2	0	0	0.736842	FALSE	0	HSPB1	122	0.55	gene
248	2.552632	0.001552	0.391753	0	2	5	FALSE	PIK3CG	24.5	2	0	0	0.741228	FALSE	0	PIK3CG	162	0.57317073	gene
294	2.473684	0.003982	0.404255	0	2	5	FALSE	ALOX15	26.5	2	0	0	0.754386	FALSE	0	ALOX15	362	0.57954545	gene
2	2.473684	0.0039	0.404	0	2	5	FAL	GPR35	26.5	2	0	0	0.7	FA	0	GPR35	3	0.5795	gen

98		82	255				SE						543866	LSE			62	4545	e
300	2.473684	0.003982	0.404255	0	2	5	FALSE	TOP1	26.5	2	0	0	0.754386	FALSE	0	TOP1	362	0.57954545	gene
376	3.907895	0.005203	0.255892	0	2	5	FALSE	butin	4	2	0	0	0.515351	FALSE	0	butin	756	0.5	compound
377	3.894737	0.001727	0.256757	0	2	6	FALSE	PTGS1	3	2	0	0	0.517544	FALSE	0	PTGS1	102	0.5	gene
303	3.473684	0	0.287879	0	1	6	FALSE	TYR	13	1	0	0	0.587719	FALSE	0	TYR	0	0	gene
306	3.473684	0	0.287879	0	1	6	FALSE	APP	13	1	0	0	0.587719	FALSE	0	APP	0	0	gene
319	2.631579	0	0.38	0	1	5	FALSE	XDH	40	1	0	0	0.72807	FALSE	0	XDH	0	0	gene
323	2.631579	0	0.38	0	1	5	FALSE	AHR	40	1	0	0	0.72807	FALSE	0	AHR	0	0	gene
328	2.631579	0	0.38	0	1	5	FALSE	CSNK2A1	40	1	0	0	0.72807	FALSE	0	CSNK2A1	0	0	gene
3	2.631579	0	0.38	0	1	5	FAL	PON1	40	1	0	0	0.7	FA	0	PON1	0	0	gen

30							SE						2807	LSE					e
332	2.631579	0	0.38	0	1	5	FALSE	MPO	40	1	0	0	0.72807	FALSE	0	MPO	0	0	gene
334	2.631579	0	0.38	0	1	5	FALSE	ACHE	40	1	0	0	0.72807	FALSE	0	ACHE	0	0	gene
336	2.631579	0	0.38	0	1	5	FALSE	ABCG2	40	1	0	0	0.72807	FALSE	0	ABCG2	0	0	gene
338	2.631579	0	0.38	0	1	5	FALSE	CYP1A2	40	1	0	0	0.72807	FALSE	0	CYP1A2	0	0	gene
341	2.631579	0	0.38	0	1	5	FALSE	TOP2A	40	1	0	0	0.72807	FALSE	0	TOP2A	0	0	gene
345	2.631579	0	0.38	0	1	5	FALSE	NR1I2	40	1	0	0	0.72807	FALSE	0	NR1I2	0	0	gene
347	2.631579	0	0.38	0	1	5	FALSE	CYP3A4	40	1	0	0	0.72807	FALSE	0	CYP3A4	0	0	gene
350	2.631579	0	0.38	0	1	5	FALSE	F2	40	1	0	0	0.72807	FALSE	0	F2	0	0	gene
352	2.631579	0	0.38	0	1	5	FALSE	APEX1	40	1	0	0	0.72807	FALSE	0	APEX1	0	0	gene
354	2.631579	0	0.38	0	1	5	FALSE	AKR1B1	40	1	0	0	0.72807	FALSE	0	AKR1B1	0	0	gene
3	2.631579	0	0.38	0	1	5	FAL	PARP1	40	1	0	0	0.7	FA	0	PARP1	0	0	gen

60							SE						2807	LSE					e
362	2.631579	0	0.38	0	1	5	FALSE	HSF1	40	1	0	0	0.728807	FALSE	0	HSF1	0	0	gene
370	3.868421	0	0.258503	0	1	6	FALSE	CBR1	4	1	0	0	0.522193	FALSE	0	CBR1	0	0	gene
384	4.026316	0	0.248366	0	1	6	FALSE	CHEK1	4	1	0	0	0.495614	FALSE	0	CHEK1	0	0	gene
