# Supplementary figures and images for: Pharmacological Mechanisms Underlying the Hepatoprotective Effects of Ecliptae herba on Hepatocellular Carcinoma
Source: Evid Based Complement Alternat Med. 2021 Jul 16;2021:5591402. doi: 10.1155/2021/5591402 (PMC8302389; doi:10.1155/2021/5591402)

## Akt & beta-Actin

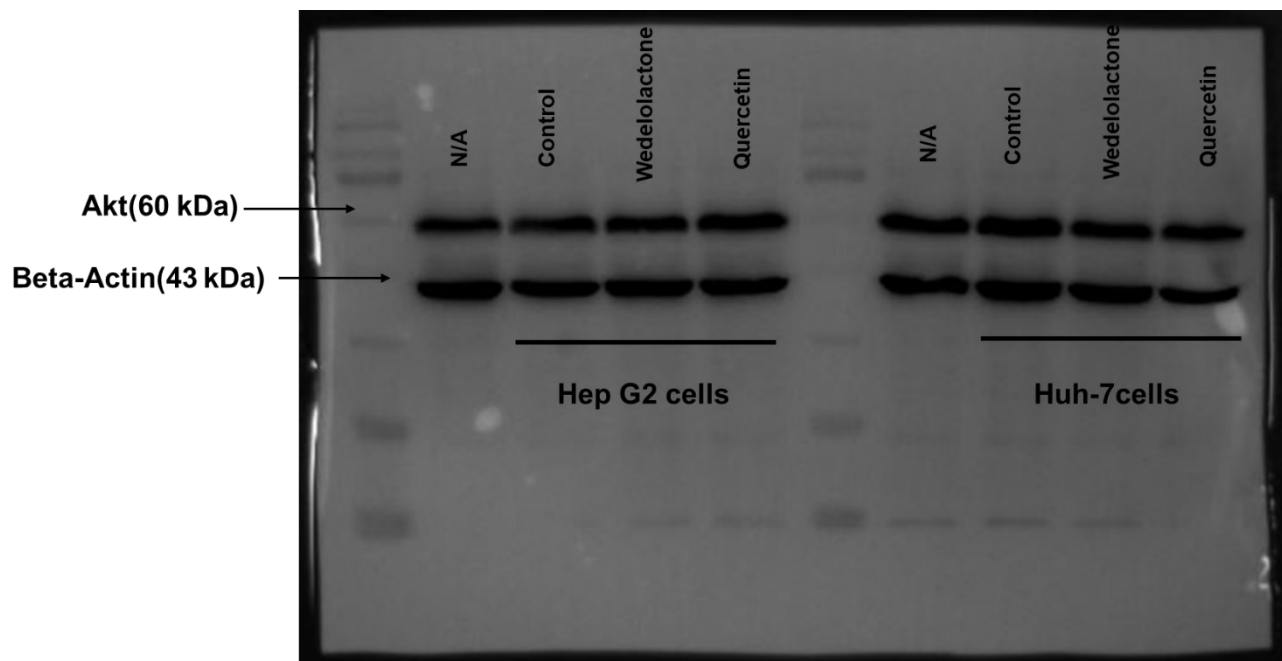

## p-Akt (Ser473) & beta Actin

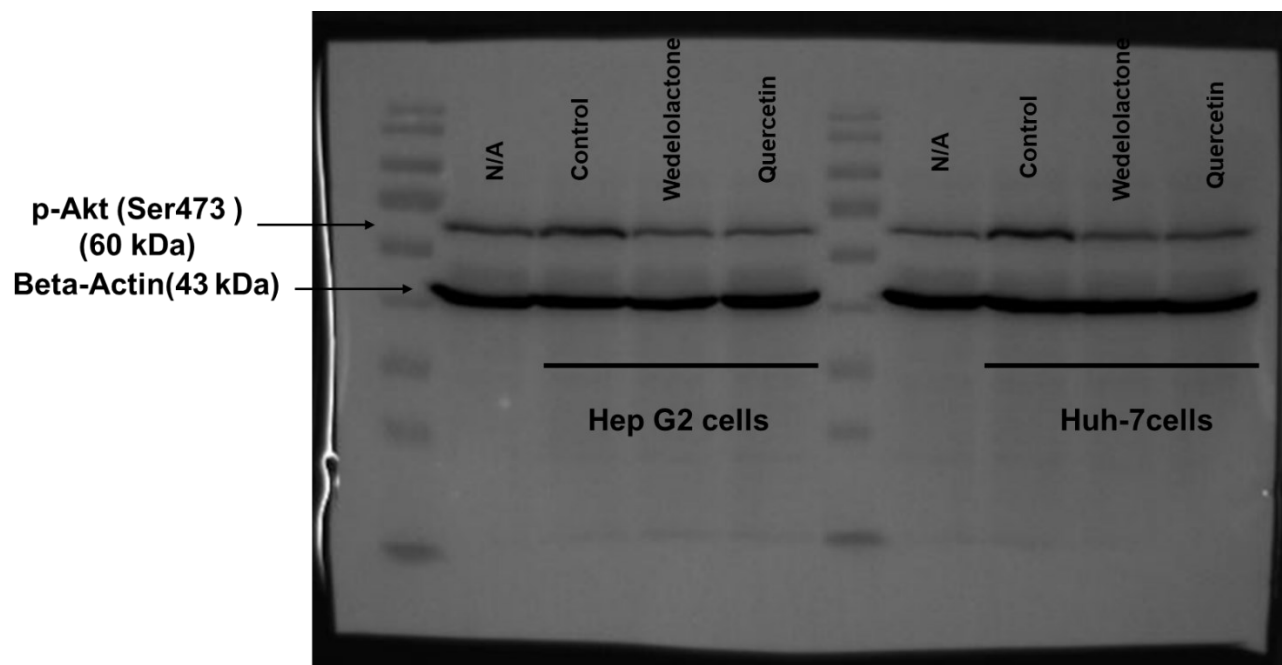

## PI3K p110 $\alpha$ & beta-Actin

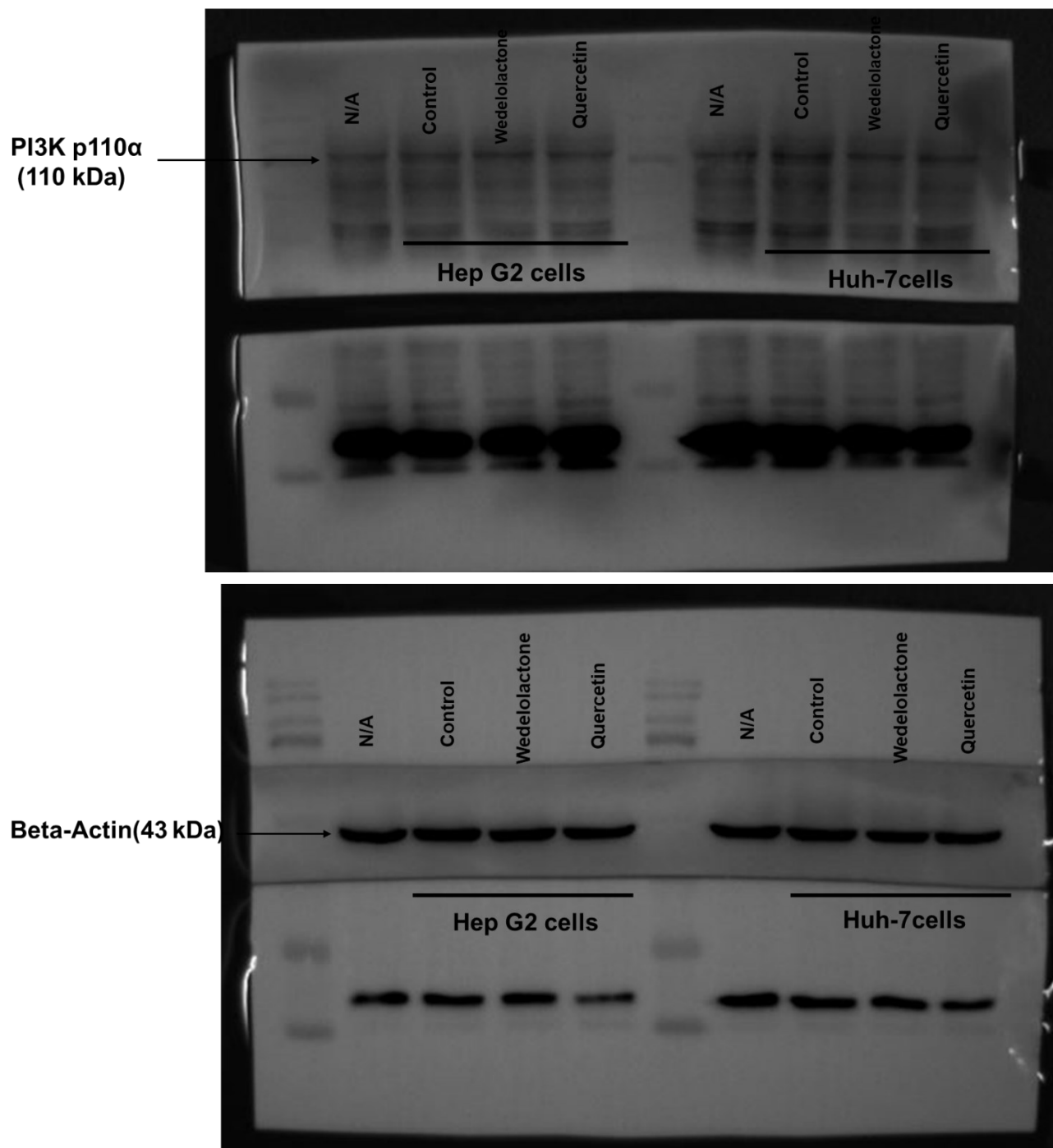

## p-PI3K & beta-Actin

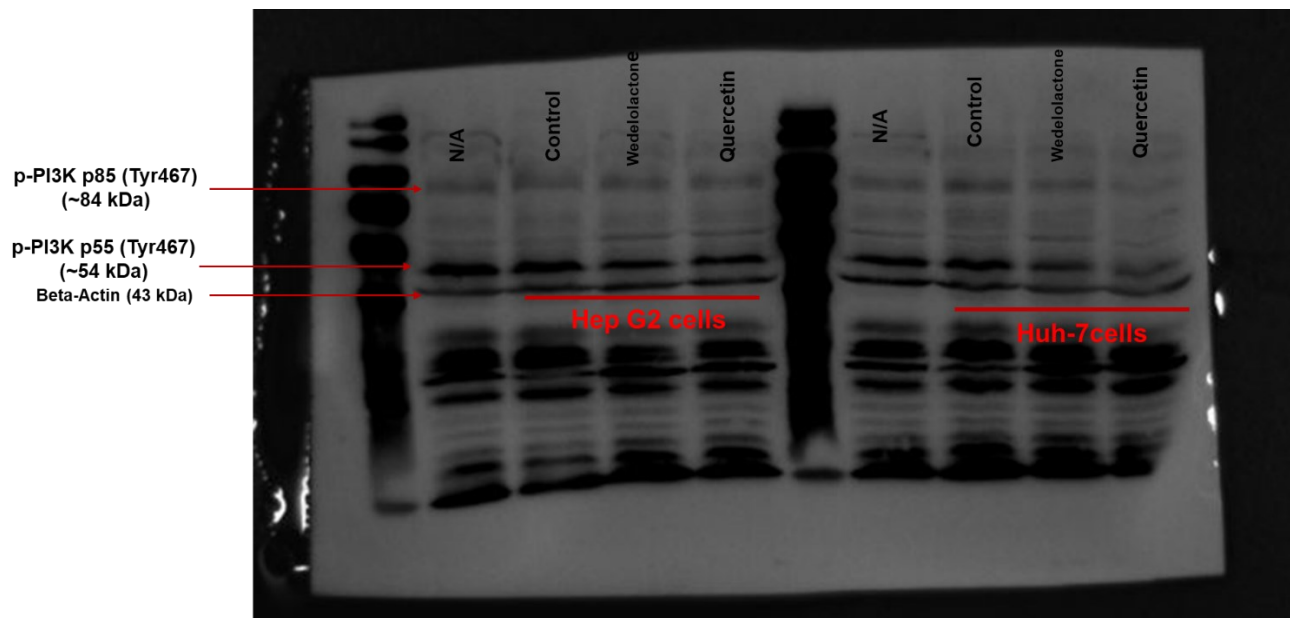

Supplement: Supplementary Materials — Supplementary File S1: a total of 48 chemical ingredients of EH were obtained from TCMSP. Supplementary File S2: detailed information of the targets of 6 active ingredients in EH was extracted from three databases, TCMSP, DGIDB, and SwissTargetPrediction. Supplementary File S3: detailed information on HCC-related targets was extracted from GeneCards and CTD. Supplementary File S4: detailed information on the PPI network of 52 potential therapeutic targets for HCC was obtained from the STRING platform. Supplementary File S5: topological parameters of nodes in the E-H network obtained from Cytoscape. Supplementary File S6: detailed information on GO enrichment analysis obtained from WebGestalt. Supplementary File S7: detailed information on the top 10 GO terms of the GO network in the TCGA RNASeq LIHC database through Network Topology-based Analysis obtained from WebGestalt. Supplementary File S8: detailed information on the top 20 KEGG enrichment pathways obtained from the WebGestalt. Supplementary File S9: detailed information on the C-T-P network obtained from Cytoscape. [file 5591402.f1.zip › 5591402.f1/Summary of the original picture of western blot assay.pdf]
